# Lysine methyltransferase SMYD2 inhibits antiviral innate immunity by promoting IRF3 dephosphorylation

**DOI:** 10.1038/s41419-023-06118-y

**Published:** 2023-09-06

**Authors:** Jiacheng Wu, Ye Hu, Jiaying Song, Jia Xu, Qian Zhang, Yangyang Chai, Xin Wang, Bingjing Wang, Yong Zhao, Xuetao Cao, Xiaoqing Xu

**Affiliations:** 1 Department of Immunology, Center for Immunotherapy, Institute of Basic Medical Sciences, Peking Union Medical College, Chinese Academy of Medical Sciences, Beijing, 100005 China; 2 College of Life Sciences, Nankai University, Tianjin, 300071 China; 3 National Key Laboratory of Medical Immunology, Institute of Immunology, Second Military Medical University, Shanghai, 200433 China; 4 Fuwai Central China Cardiovascular Hospital, Heart Center of Henan Provincial People’s Hospital, Zhengzhou, 450046 China

**Keywords:** Innate immunity, Infection

## Abstract

Phosphorylation of IRF3 is critical to induce type I interferon (IFN-I) production in antiviral innate response. Here we report that lysine methyltransferase SMYD2 inhibits the expressions of IFN-I and proinflammatory cytokines in macrophages upon viral infections. The *Smyd2*-deficient mice are more resistant to viral infection by producing more IFN-I and proinflammatory cytokines. Mechanistically, SMYD2 inhibits IRF3 phosphorylation in macrophages in response to viral infection independent of its methyltransferase activity. We found that SMYD2 interacts with the DNA-binding domain (DBD) and IRF association domain (IAD) domains of IRF3 by its insertion SET domain (SETi) and could recruit phosphatase PP1α to enhance its interaction with IRF3, which leads to decreased phosphorylation of IRF3 in the antiviral innate response. Our study identifies SMYD2 as a negative regulator of IFN-I production against virus infection. The new way of regulating IRF3 phosphorylation will provide insight into the understanding of IFN-I production in the innate response and possible intervention of the related immune disorders.

## Introduction

Innate immune response is the first line that protects the host from pathogen infection. Pattern recognition receptors (PRRs), such as RIG-I, MDA5, and cGAS, expressed in both innate immune cells and other type of cells recognize pathogen-associated molecular patterns (PAMPs) to trigger signaling pathways, such as MAVS-TBK1-IRF3 pathway, and phosphorylated IRF3 (p-IRF3) translocates into nucleus, which induces the production of type I interferon (IFN-I) to eliminate the invading pathogens including viruses [1]. NF-κB is also crucial for production of IFN-I and proinflammatory cytokines after sensing viral RNA or DNA [2, 3]. IRF3 has an N-terminal DNA-binding domain (DBD) with characteristic five-tryptophan repeats, and C-terminal containing a conserved IRF association domain (IAD) and phosphoacceptor sites-rich sequence [4]. IRF3 is constitutively expressed and localized in the cytoplasm as an inactive monomer which is maintained by auto-inhibitory domains flanking the IAD. The phosphorylation of IRF3 by TBK1/IKKε is crucial to induce IFN-I in response to virus infection [5]. The regulation of IRF3 phosphorylation in the innate response needs to be fully understood.

The previous studies have shown that phosphorylation of C-terminal region of IRF3 results in release of IAD to form dimers, which leads to its nucleus translocation for triggering the transcription of IFNs [6]. Previous studies showed that proteasome-mediated degradation, SUMOylation, and dephosphorylation are three mechanisms to decrease transcriptional activity of the activated IRF3 [7]. Peptidylprolyl cis-trans isomerase, NIMA-interacting 1 (Pin1) [8], Cullin-based ubiquitin ligases [9] and RBCC protein interacting with PKC1 (RBCK1) [10] are involved in proteasome-mediated degradation of IRF3. SUMOylation at K152 of IRF3 leads to inactivation of IRF3 [11]. Caspase-8 [12], PTEN [13], phosphatase protein 2 A (PP2A) [7], and PP1cc [14] are reported in regulation of dephosphorylation of IRF3. Dysregulation of activation and inactivation of IRF3 is closely associated with infectious, inflammatory, and autoimmune diseases [15, 16]. We wondered whether there are previously unidentified regulators of IRF3 phosphorylation and dephosphorylation in antiviral innate immune response, which needs to be further investigated.

Lysine methyltransferases modify histone and no-histone protein by methylation, which has a critical function in a series of biological and immunological processes by regulation of chromatin remodeling and protein conformation [17, 18]. SMYD proteins are a class of protein lysine methyltransferases, which methylate histones and non-histone targets, with five identified members SMYD 1–5 so far. SMYD, exception of SMYD5, is comprised with s-sequence, MYND (Myeloid-Nervy-DEAF1) domain, insertion SET domain (SETi), ET, post-ET and CTD domain, and the structure of this family is special because the SET (Suppressor of variegation, Enhancer of Zeste, Trithorax) domain is separated by an MYND domain [18]. The SET domain is a conserved catalytic unit for lysine methylation found in nearly all histone methyltransferases, and the MYND domain is zinc finger motif which mainly mediates the protein-protein interaction [17]. SMYD proteins have been reported to play important roles in tumorigenesis, myogenesis, and cardiomyocyte differentiation [19]. However, the function of SMYD2 in antiviral innate immune response remains unclear.

Here we demonstrated that SMYD2 suppresses the production of antiviral IFN-I by inhibition of IRF3 phosphorylation which is independent of its methyltransferase activity. Mechanistically, SMYD2 interacted with IRF3 via binding IAD domain of IRF3, and phosphatase PP1α was recruited by SMYD2 to IRF3 to decrease phosphorylation of IRF3. Overall, SMYD2 is a negative regulator of antiviral interferon in innate response.

## Results

### SMYD2 is decreased in macrophages in response to viral infection

By analyzing the microarray data to identify differential expressions of lysine methyltransferases in macrophages infected with vesicular stomatitis virus (VSV) [20], we found the expressions of 10 lysine methyltransferases including *Prdm6*, *Prdm9*, *Setd1a*, *Setmar*, *Smyd1*, *Smyd4*, *Smyd5*, *Suv39h1*, *Suv39h2,* and *Whsc1/1*, remained almost unchanged, while the expressions of lysine methyltransferases including *Smyd2*, *Smyd3*, *Nsd1*, *Prdm2*, *Setd1b*, *Setd2*, *Setd3*, *Setd7*, *Setd8*, *Setdb1*, *Suv420h1*, *Suv420h2,* and *Whsc1* were decreased, and the expression of *Setdb2* was increased in macrophages upon VSV infection (Supplementary Fig. 1A). Intriguingly, we found that mRNA expressions of *Smyd2* and *Smyd3* but not *Smyd1, Smyd4,* and *Smyd5* were remarkably decreased in macrophages upon VSV infection (Supplementary Fig. 1A). We then confirmed the decreased mRNA expression of *Smyd2*, *Smyd3*, *Nsd1*, *Suv420h1-1*, *Setd1b*, *Setdb1* and *Setdb7* in macrophages infected with VSV (Supplementary Fig. 1B).

To clarify whether the decreased lysine methyltransferases were involved in innate immune response, we knocked down the expression of *Smyd2*, *Smyd3*, *Setdb1,* and *Setdb7* (Supplementary Fig. 2A), and found that IFN-β expression was significantly increased in macrophages once silencing of *Smyd2* (Supplementary Fig. 2B). *Smyd2* was expressed higher in macrophages than that in other immune cells including CD4^+^ T cells, CD8^+^ T cells, B cells, NK cells and dendritic cells (Supplementary Fig. 2C) and was also expressed in immune organs thymus and spleen, with the highest level in the heart (Supplementary Fig. 2D). Flag-SMYD2 was mainly located in cytoplasm of HEK293T cells, and lower expression of this protein was found in the nucleus of the cells (Supplementary Fig. 2E). Endogenous SMYD2 was further confirmed in both cytoplasm and nucleus of macrophages, and its expression was decreased in response to VSV stimulation (Supplementary Fig. 2F–H). We further validated the reduction of both mRNA and protein expression of *Smyd2* in macrophages infected with virus, LPS, and Listeria monocytogenes (Supplementary Fig. 2I, J). Also, the expression of SMYD2 protein was decreased upon IFN-α and IFN-β but not IL-6 or TNF-α stimulation (Supplementary Fig. 2K). These data indicated that SMYD2 might be involved in innate immune response.

### SMYD2 inhibits innate cytokine production in macrophages in response to viral infection

**Fig. 1:**
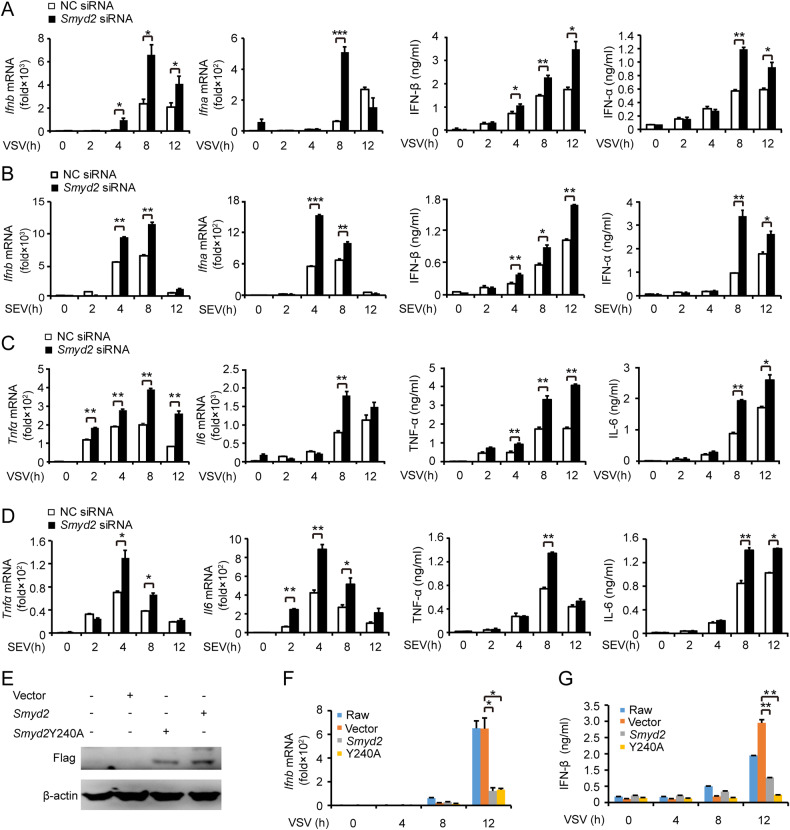
Increased expression of type I interferon and proinflammatory cytokines in macrophages after silencing of *Smyd2*. **A, B** Production of IFN-β and IFN-α by PMs silenced of *Smyd2* in response to VSV (**A**) or SEV (**B**) are analyzed with qRT-PCR and ELISA. **C**, **D** Production of IL-6 and TNF-α by PMs treated as (**A, B**) are analyzed with qRT-PCR and ELISA. **E** Detection of Flag-SMYD2 and Flag-SMYD2Y240A by western blot. **F**, **G** qRT-PCR (**F**) and ELISA (**G**) analysis of IFN-β production by RAW 264.7 overexpressed of SYMD2 or SYMD2Y240A. Vector used as control. Error bars represent s.d. Student’s *t* test. *p < 0.05, **p < 0.01, ***p < 0.001. All data are representative of three independent experiments. (**A**–**D**, **F**, **G**; mean ± s.d.).

We then examined IFN-β, IFN-α, IL-6, and TNF-α expressions in macrophages infected with RNA virus VSV and Sendai virus (SEV) after silencing of *Smyd2*. We found that both mRNA and protein expression of IFN-β and IFN-α were significantly elevated (Fig. 1A, B), and the production of IL-6 and TNF-α were also increased (Fig. 1C, D). Therefore, SMYD2 may inhibit the expressions of IFN-I and proinflammatory cytokines in macrophages in response to RNA virus infection.

In addition, overexpression of wild-type SMYD2 and lysine methyltransferases-dead mutant SMYD2Y240A [21] inhibited mRNA and protein expression of IFN-β in RAW 264.7 cells in response to VSV stimulation (Fig. 1E–G). The inhibitory function of SMYD2Y240A mutant on innate immune response indicated that the suppression of IFN-I by SMYD2 might be independent of its methyltransferase activity.

### *Smyd2*-deficient macrophages exhibit a more potent antiviral innate response

We generated *Smyd2*^−/−^ mouse by deletion of Exon 7 flanked with floxp, which leads to frameshift mutation when crossed with 129 S/Sv-Tg (Prm-cre) 58Og/J (Supplementary Fig. 3A). We confirmed the deletion efficiency of *Smyd2* by PCR and Western blot (Supplementary Fig. 3B, C).

**Fig. 2:**
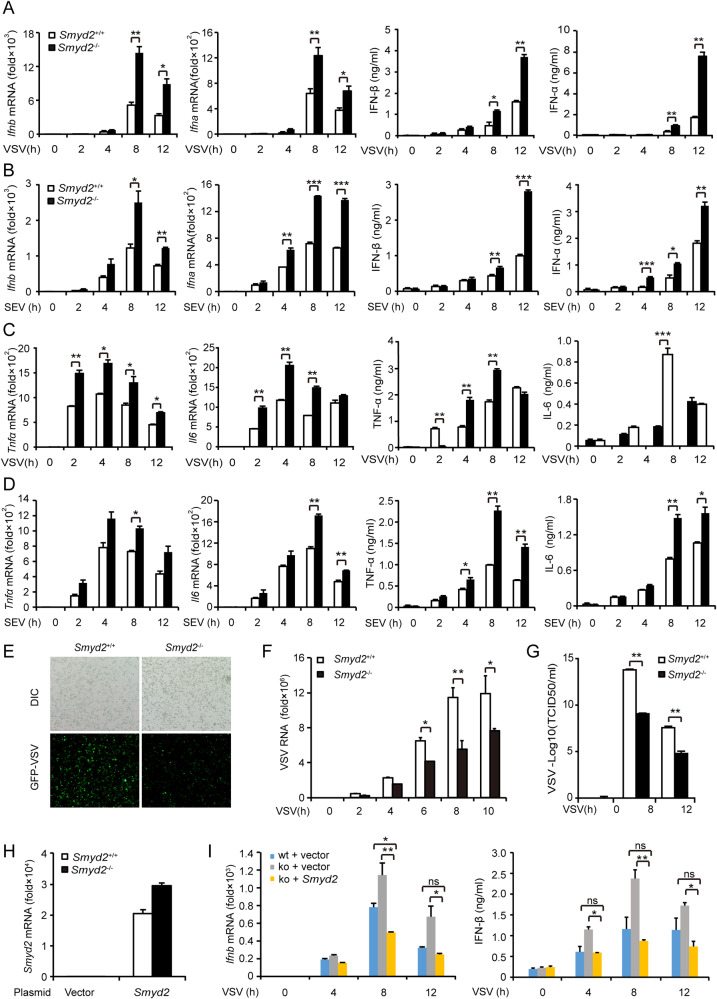
Increased production of type I interferon and proinflammatory cytokines in *Smyd2*-deficient macrophages. **A, B** Production of IFN-β and IFN-α4 by *Smyd2*^−/−^ PMs in response to VSV (**A**) or SEV (**B**) as indicated time is analyzed with qRT-PCR and ELISA. **C**, **D** Production of IL-6 and TNF-α by *Smyd2*^−/−^ PMs treated as (**A, B**) are analyzed with qRT-PCR and ELISA. **E** Observation of GFP-VSV replication in virus-infected *Smyd2*^−/−^ and *Smyd2*^+/+^ PMs for 8 hours under microscope. **F** VSV mRNA in *Smyd2*^−/−^ and *Smyd2*^+/+^ PMs infected with VSV as indicated time is analyzed by qRT-PCR. **G** Analyzation of VSV titer in supernatants of *Smyd2*^−/−^ and *Smyd2*^+/+^ PMs infected with VSV as indicated time by TICD_50_ assay. **H** qRT-PCR examination of *Smyd2* mRNA overexpressed in *Smyd2*^−/−^ and *Smyd2*^+/+^ PMs. **I** Production of IFN-β by *Smyd2*^−/−^ and *Smyd2*^+/+^ PMs overexpressed of *Smyd2* or vector in response to VSV infection as indicated time by qRT-PCR and ELISA. Vector used as control. Error bars represent s.d. Student’s *t* test. *p < 0.05, **p < 0.01, ***p < 0.001. All data are representative of three independent experiments. (**A**–**D**, **F**–**I**; mean ± s.d.).

We found that both mRNA and protein expression of IFN-β, IFN-α, IL-6, and TNF-α were substantially increased in *Smyd2*^−/−^ peritoneal macrophages (PMs) compared with control cells infected with VSV and SeV (Fig. 2A–D). We also observed that the load of GFP-VSV in *Smyd2*^−/−^ PMs was obviously decreased compared with control (Fig. 2E). We further confirmed that VSV replication and VSV titer were significantly reduced in *Smyd2*^−/−^ PMs (Fig. 2F, G). Significantly elevated expression of *Ifnb* mRNA was also found in *Smyd2*^−/−^ dendritic cells (DCs) infected with VSV and SeV (Supplementary Fig. 4A). We further identified the increased production of IFN-I in *Smyd2*^−/−^ PMs in response to DNA virus herpes simplex virus (HSV) stimulation (Supplementary Fig. 4B). Moreover, both mRNA and protein expressions of IFN-β were significantly decreased in *Smyd2*^−/−^ PMs in response to VSV infection when *Smyd2* expression was rescued (Fig. 2H, I). Therefore, SMYD2 inhibits the production of IFN-I and proinflammatory cytokines IL-6 and TNF-α in immune cells upon viral infection.

### Deficiency of *Smyd2* protects mice from infection with RNA virus

To clarify whether SMYD2 affects the development of immune cells, we examined the ratios of immune cells in the spleen of *Smyd2*^−/−^ and *Smyd2*^+/+^ mice by FACS analysis, and the results showed that the ratios of B cells, CD4^+^ T cells, CD8^+^ T cells, dendritic cells, macrophages, NK cells and neutrophils were comparable between *Smyd2*^−/−^ and *Smyd2*^+/+^ spleens (Supplementary Fig. 5A, B).

**Fig. 3:**
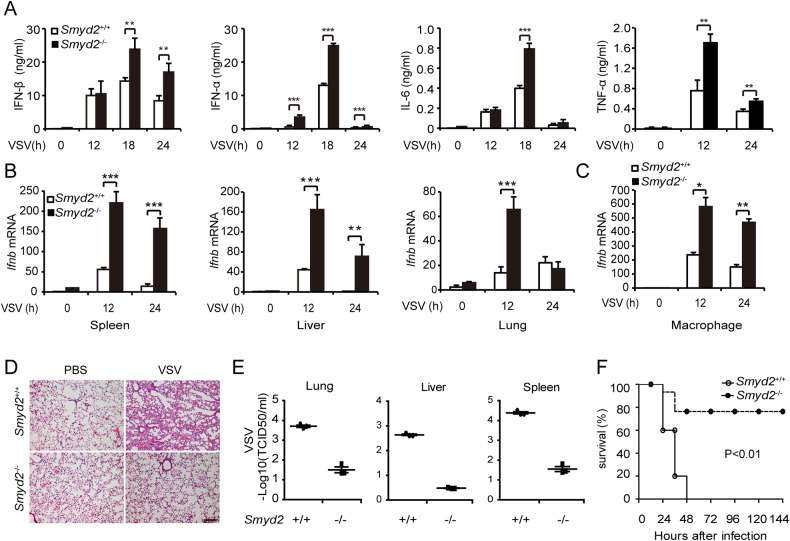
*Smyd2-*deficient mice are more resistant to viral infection by producing more type I interferon and proinflammatory cytokines. **A** Analysis of IFN-β, IFN-α4, IL-6, and TNF-α concentration in the sera of *Smyd2*^−/−^ and *Smyd2*^+/+^ mice infected with VSV as indicated time (*n* = 3 per group). **B** qRT-PCR analysis of *Ifnb* mRNA in the organs from *Smyd2*^−/−^ and *Smyd2*^+/+^ mice infected with VSV as indicated time (*n* = 3 per group). **C** qRT-PCR analysis of *Ifnb* mRNA in the PMs from *Smyd2*^−/−^ and *Smyd2*^+/+^ mice treated as (**B**). **D** Hematoxylin and eosin staining of lung sections from *Smyd2*^−/−^ and *Smyd2*^+/+^ mice to observe inflammation in response to VSV infection for 18 hours (scale bar: 100μm). **E** Examination of VSV loads in organs from *Smyd2*^−/−^ and *Smyd2*^+/+^ mice (*n* = 6 per group) intraperitoneally injected with VSV for 24 h by TCID50 assay. **F** Survival analysis of *Smyd2*^−/−^ and *Smyd2*^+/+^ mice with intraperitoneal injection of VSV (1 × 10^8^ pfu/g) (*n* = 15 per group). Error bars represent s.d. Student’s *t* test. *p < 0.05, **p < 0.01, ***p < 0.001. Data are from three independent experiments (**A**–**C**; mean±s.d. of technical triplicates) or are representative of three independent experiments (**E**, **F**; mean ± s.d.).

To investigate the function of SMYD2 in host antiviral innate response in vivo, we challenged *Smyd2*^−/−^ and *Smyd2*^+/+^ mice with VSV. Higher concentrations of IFN-β, IFN-α, IL-6, and TNF-α were detected in sera of *Smyd2*^−/−^ mice in response to VSV infection compared with control (Fig. 3A). Our results showed the significantly elevated expression of *Ifnb* mRNA in spleen, liver, lung and peritoneal macrophages of *Smyd2*^−/−^ mice in contrast with wild-type mice (Fig. 3B, C). Less infiltration of inflammatory cells was observed in lungs of *Smyd2*^−/−^ mice after infection with VSV (Fig. 3D). VSV titer in organs from *Smyd2*^−/−^ mice was significantly decreased compared with control (Fig. 3E). Moreover, *Smyd2*^−/−^ mice were more resistant to VSV infection compared with wild-type mice (Fig. 3F). Therefore, *Smyd2*-deficient mice have more potent resistance against viral infection by production of more IFN-I and proinflammatory cytokines.

### Deletion of *Smyd2* promotes expression of a serial of type I cytokines and ISGs

**Fig. 4:**
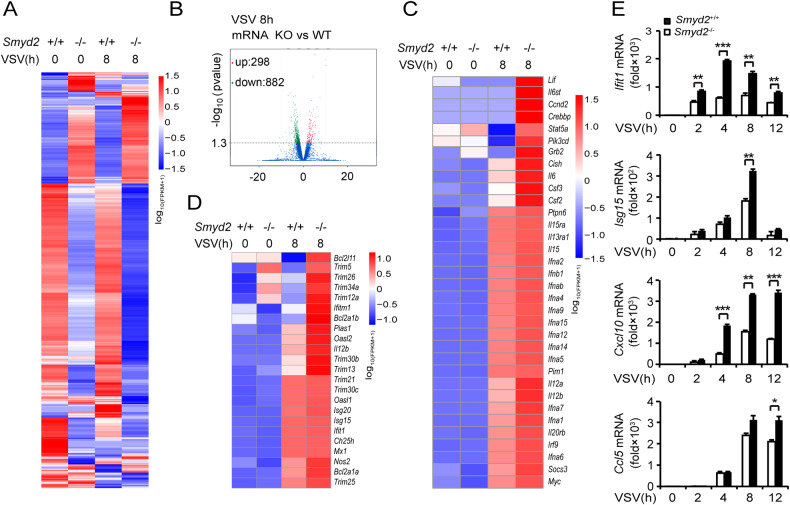
Increased antiviral innate immune responses in *Smyd2-*deficient macrophages. **A** The cluster heat map of RNA-seq data shows the differential genes (*P* < 0.05) in *Smyd2*^−/−^ and *Smyd2*^+/+^ PMs without or with VSV infection for 8 h. **B** Increased-regulation genes or decreased-regulation genes between *Smyd2*^*−/−*^ and *Smyd2*^*+/+*^ PMs infected with VSV for 8 hours are shown in the form of volcano plot. **C** The cluster heat map shows increased genes, such as type I interferon, inflammatory cytokines, and transcriptional factors, in the *Smyd2*^−/−^ PMs with VSV infection for 8 h (*P* < 0.05). **D** The cluster heat map shows increased ISGs in the *Smyd2*^−/−^ PMs with VSV infection for 8 h (*P* < 0.05). **E** qRT-PCR analysis of ISGs in *Smyd2*^−/−^ and *Smyd2*^+/+^ PMs infected with VSV as indicated time. Error bars represent s.d. Student’s *t* test. *p < 0.05, **p < 0.01, ***p < 0.001. Data are representative of three independent experiments (**E**; mean ± s.d.).

Transcriptome sequencing showed that there were 298 increased and 882 decreased genes in *Smyd2*^−/−^ PMs in response to VSV infection compared with control (Fig. 4A, B). KEGG analysis showed that the differentially expressed genes in *Smyd2*^−/−^ PMs without VSV stimulation were enriched in the signaling pathways including ubiquitin-mediated proteolysis, regulation of actin cytoskeleton, lysosome, herpes simplex infection, Rap1 signaling pathway and chemokine signaling pathway (Supplementary Fig. 6A). The differentially expressed genes in *Smyd2*^−/−^ PMs infected with VSV were enriched in the pathways, such as TNF signaling pathway, PI3K-Akt signaling pathway, focal adhesion and ECM-receptor interaction (Supplementary Fig. 6B). We further analyzed the sequencing data to confirm the increased expression of genes involved in immune response in *Smyd2*^−/−^ PMs infected with VSV. We found that mRNA expression of a serial of cytokines, especially IFN-I, and transcriptional factor *Stat5a* were significantly increased in *Smyd2*^−/−^ PMs compared with control (Fig. 4C). Moreover, a number of interferon-stimulated genes (ISGs), such as *Bcl2l11* and *Trim* family, were increased in *Smyd2*^−/−^ PMs in response to VSV infection (Fig. 4D). Several ISGs, such as *Ifit1*, *Isg15*, *Cxcl10* and *Ccl5*, were confirmed to be significantly increased in *Smyd2*^−/−^ PMs infected with VSV (Fig. 4E). Taken together, our data of transcriptome sequencing further confirm that SMYD2 inhibits antiviral innate immune response by targeting IFN-I production.

### Increased phosphorylation of IRF3 in *Smyd2-*deficient macrophages in response to virus infection

**Fig. 5:**
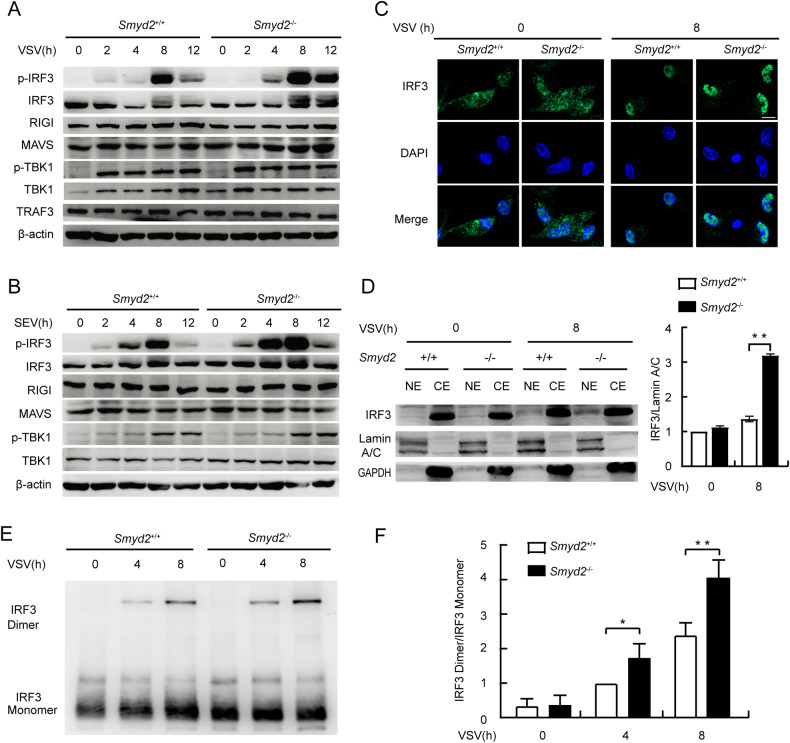
Increased IRF3 phosphorylation in *Smyd2-*deficient macrophages in response to VSV infection. **A**, **B** IB analysis of phosphorylation of critical molecules involved in RIGI signaling pathway in the lysates of *Smyd2*^−/−^ and *Smyd2*^+/+^ PMs infected with VSV (**A**) or SEV (**B**) as indicated time. **C** Examination of IRF3 translocation to nucleus of *Smyd2*^−/−^ and *Smyd2*^+/+^ PMs infected without or with VSV infection for 8 h by immunofluorescence (scale bar: 10μm). **D** IB analysis of IRF3 protein in nucleus and cytoplasm isolated from *Smyd2*^−/−^ and *Smyd2*^+/+^ PMs infected with VSV as indicated time and quantification of IRF3 in nucleus by ImageJ (right). **E** Detection of dimerized and monomeric IRF3 in *Smyd2*^−/−^ and *Smyd2*^+/+^ PMs infected with VSV as indicated time by native PAGE. **F** Quantification of dimerized IRF3/ monomeric IRF3 ratio by ImageJ (right). Error bars represent s.d. Student’s *t* test. *p < 0.05, **p < 0.01, ***p < 0.001. Data are representative of three independent experiments (**D**, **F**; mean ± s.d.).

To clarify the molecular mechanisms for SMYD2 in regulating antiviral innate immune response, we examined the innate signaling pathways and found that phosphorylation of IRF3 was obviously increased in the *Smyd2*^−/−^ PMs infected with both VSV and SEV (Fig. 5A, B). Deletion of *Smyd2* had no effect on phosphorylation of TBK1, and the expression levels of RIG-I and MAVS were comparable between *Smyd2*^−/−^ and *Smyd2*^+/+^ PMs (Fig. 5A, B). We also confirmed that IRF3 protein located in the nucleus was significantly increased in the *Smyd2*^−/−^ PMs infected with VSV by immunofluorescence and Westen blot (Fig. 5C, D). We further confirmed that the deletion of *Smyd2* remarkably promoted IRF3 dimerization in the PMs infected with VSV (Fig. 5E, F). Our results also showed the increased p-IKKα/β and p-P65 of NF-κB signaling pathway in *Smyd2*-deficient PMs infected with VSV (Supplementary Fig. 7A). Taken together, deficiency of *Smyd2* promotes phosphorylation of IRF3 in macrophages in response to virus infection, which leads to increased translocation of dimerized IRF3 to the nucleus to promote IFN-I production.

### SMYD2 inhibits IFN-I production and IRF3 phosphorylation independent of its methyltransferase activity

**Fig. 6:**
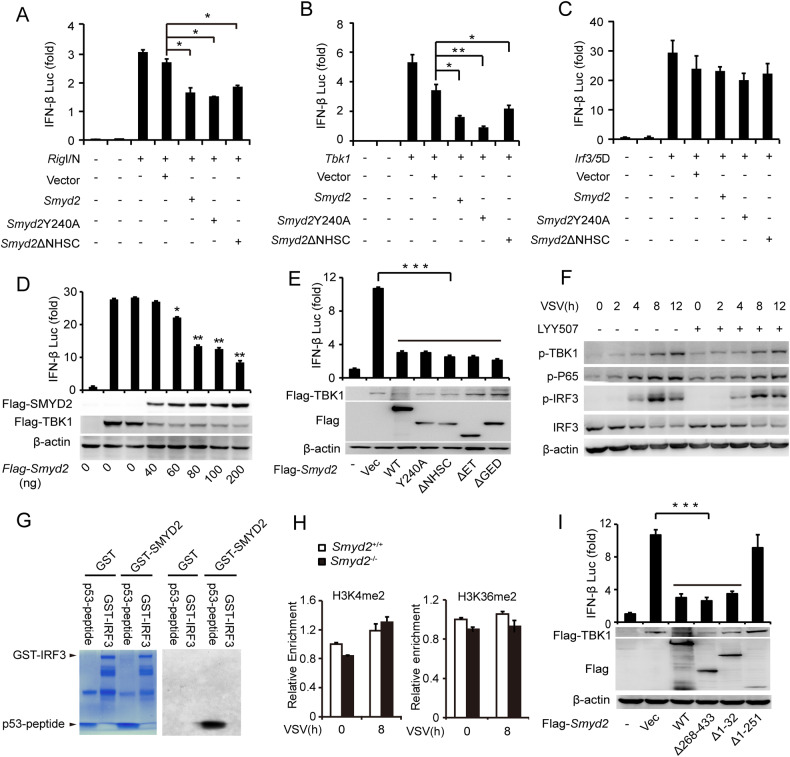
SMYD2 inhibits antiviral IFN-β production independent of its methyltransferase activity. **A**–**C** Examination of IFN-β production in HEK293T cells co-transfected with wild-type *Smyd2* or its mutants and *Rig*I/N (**A**), *Tbk1* (**B**), or *Irf3*/5D (**C**) by Luciferase activity assay. **D** Luciferase activity assay in lysates of HEK293T cells co-transfected with gradient doses of *Smyd2* plasmid with or without *Tbk1* plasmid (up panel); IB analysis of SMYD2 and TKB1 overexpressed in HEK293T cells (down panel). **E** Luciferase activity assay in lysates of HEK293T cells co-transfected with *Tbk1* plasmid and wild-type and mutant plasmids of *Smyd2* (up panel); IB analysis of SMYD2 and TKB1 overexpressed in HEK293T cells (down panel). **F** IB analysis of phosphorylation of TBK1, IRF3, and P65 in PMs pretreated without or with different doses of LYY507, a specific methyltransferase inhibitor of SMYD2, in response to VSV infection. **G** Autoradiogram of HMTase assay with recombinant GST, GST-SMYD2, GST-IRF3, and synthesized p53 peptide 362-380, left shows Coomassie staining of purified recombinant proteins and synthesized p53 peptide, and right shows autoradiogram with S-Adenosyl-L-[methyl-^3^H]. **H** CHIP assay of H3K4me2 and H3K36me2 modification at *Ifnb* promotor in *Smyd2*^−/−^ and *Smyd2*^+/+^ PMs infected without or with VSV for the indicated time. **I** Luciferase activity assay in lysates of HEK293T cells co-transfected with *Tbk1* plasmid and wild-type and truncated mutant plasmids of *Smyd2* (up panel); IB analysis of SMYD2 and TKB1 overexpressed in HEK293T cells (down panel). Error bars represent s.d. Student’s *t* test. *p < 0.05, **p < 0.01, ***p < 0.001. Data are representative of three independent experiments (**A**–**E**, **H**, **I**; mean ± s.d.).

SMYD2 is a lysine methyltransferase, which methylate histones and non-histone proteins. We further clarify whether SMYD2 regulates the antiviral innate response by its methyltransferase activity. The luciferase activity assay showed that SMYD2 significantly inhibited RIGI-N (N-terminal of RIGI) and TBK1-driven *Ifnb* activation but not transcription activity of IRF3 (5D) (Fig. 6A–C), which indicated that SMYD2 inhibited antiviral innate response by attenuating the activation of IRF3. These results were also consistent with results of increased phosphorylation of IRF3 in *Smyd2*^−/−^ PMs (Fig. 5A, C). We further confirmed that SMYD2 inhibited TBK1-driven *Ifnb* activation in a dose-dependent manner (Fig. 6D). In addition, we found that *Smyd2* mutants *Smyd2*Y240A, *Smyd2*ΔNHSC, *Smyd2*ΔET and *Smyd2*ΔGED, which lack methyltransferase activity in publications [18, 19, 21], had comparable inhibitory effect on *Ifnb* expression as well as wild-type *Smyd2* (Fig. 6A, B, E). VSV infection-induced IFN-β production was also remarkably inhibited in RAW 264.7 cells overexpressing *Smyd2*Y240A mutant (Fig. 1E, F). We then confirmed that phosphorylation of IRF3 and TBK1 were not influenced by LYY507, a specific inhibitor of SMYD2 methyltransferase activity, in the macrophages in response to VSV infection (Fig. 6F). The activity of LYY507 was confirmed by examination of *P21* mRNA in macrophages treated with this inhibitor (Supplementary Fig. 7B).

To clarify whether SMYD2 directly methylates IRF3, we performed an Autoradiogram of HMTase assay. The results showed that purified GST-SMYD2 couldn’t methylate the purified GST-IRF3 positively controlled with synthesized p53 peptide SRAHSSHLKSKKGQSTSRH, which includes the K370 methylated by SMYD2 (Fig. 6G, Supplementary Fig. 7C). Published study showed that SMYD2 di-methylates H3K4 and H3K36 to control expressions of target genes [22]. To identify whether SMYD2 attenuates the expression of *Ifnb* by methylation of histone, we then examined the status of H3K4me2 and H3K36me2 by CHIP assay in the promoter region of *Ifnb* after deletion of *Smyd2*. Our results showed that the abundance of H3K4me2 and H3K36me2 wasn’t influenced after deletion of *Smyd2* in response to VSV infection (Fig. 6H).

We further investigated which domain of SMYD2 was involved in the inhibition of IFN-I expression, truncated *Smyd2*s, such as *Smyd2*Δ1-31aa, *Smyd2*Δ1-251aa, and *Smyd2*Δ268-433aa, were generated. Our luciferase activity assay showed that SMYD2 deleting S domain (1-31aa) and CTD domain (268-433aa) still inhibited the transcriptional activity of *Ifnb*, while truncated mutant *Smyd2*Δ1-251aa including S domain, MYND domain, SETi domain, and ET domain, abrogated its inhibition of *Ifnb* expression (Fig. 6I).

Taken together, we demonstrated that inhibition of antiviral IFN-β production by SMYD2 was independent of its methyltransferase activity, and 1-251aa of SMYD2 was necessary for its function in the negative regulation of antiviral innate response.

### SMYD2 interacts with the DBD and IAD domains of IRF3 by its SETi domain

**Fig. 7:**
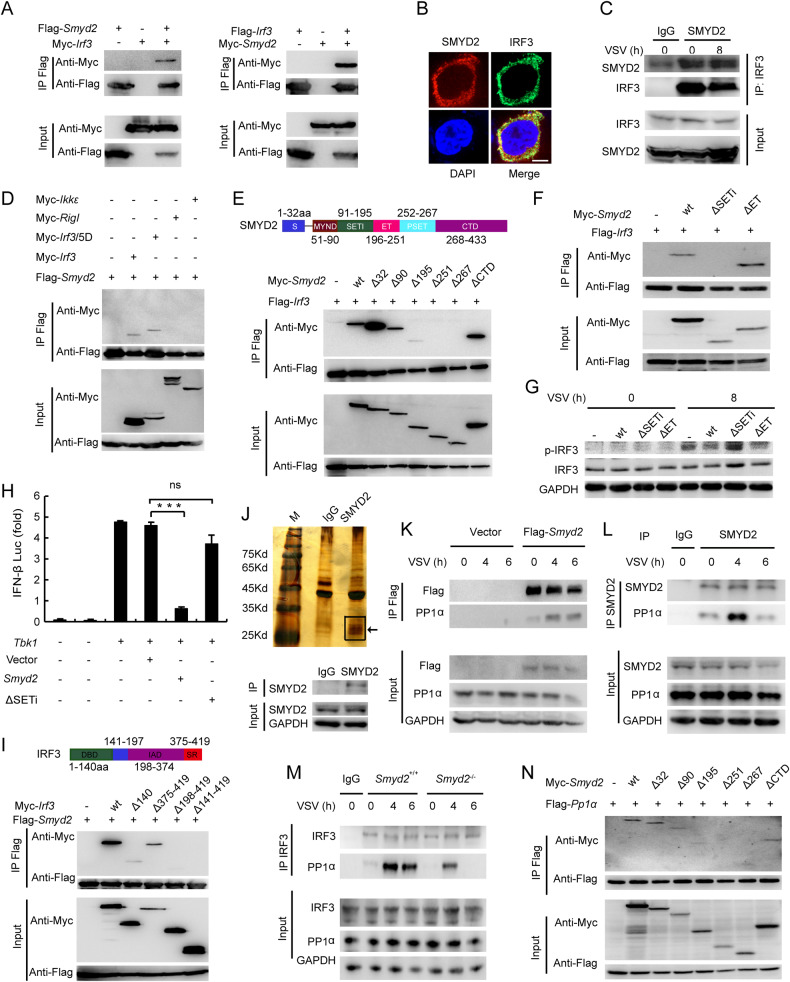
SMYD2 inhibits interaction between IRF3 and PP1. **A** Immunoblot analysis of interaction between tagged SMYD2 and IRF3 in HEK293T cells lysis immunoprecipitated using Flag antibody. **B** Identification of co-localization between SMYD2 and IRF3 in HEK293T by immunofluorescence (scale bar: 5 μm). **C** Immunoblot analysis of endogenous SMYD2 in the cell lysates of PMs immunoprecipitated by IRF3 antibody. **D** Immunoblot analysis of interaction between tagged SMYD2 and IRF3, IRF3/5D, RIGI or IKKε in HEK293T cells lysis immunoprecipitated using Flag antibody. **E** Immunoblot analysis of interaction between tagged-IRF3 with truncated mutants of SMYD2 by immunoprecipitation. **F** Immunoblot analysis of interaction between tagged-IRF3 with WT and mutants of SMYD2 by immunoprecipitation. **G** Immunoblot analysis of p-IRF3 in *Smyd2*^−/−^ PMs overexpressed with indicated plasmids treated with VSV as indicated time. **H** Luciferase activity assay in lysates of HEK293T cells co-transfected with *Tbk1* plasmid and wild-type and indicated mutants of *Smyd2*. **I** Immunoblot analysis of interaction between tagged SMYD2 with truncated mutants of IRF3 by immunoprecipitation. **J** Silver staining of proteins immunoprecipitated with SMYD2 antibody and control IgG in lysis of peritoneal macrophages, and bands indicated with arrow were analyzed by mass spectrometry (up panel); down panel showed immunoblot analysis of endogenous SMYD2 immunoprecipitated from up panel. **K** Immunoblot analysis of interaction between Flag-tagged SMYD2 and endogenous PP1α in lysis of RAW 264.7 by immunoprecipitation with Flag antibody. **L** Immunoblot analysis of interaction between endogenous SMYD2 and PP1α in lysis of peritoneal macrophages by immunoprecipitation with SMYD2 antibody. **M** Immunoblot analysis of interaction between endogenous IRF3 and PP1α in lysis of *Smyd2*^−/−^ and *Smyd2*^+/+^ PMs by immunoprecipitation with IRF3 antibody. **N** Immunoblot analysis of interaction between tagged PP1α with truncated mutants of SMYD2 by immunoprecipitation. Error bars represent s.d. Student’s *t* test. *p < 0.05, **p < 0.01, ***p < 0.001. Data are representative of three independent experiments (**H**; mean ± s.d.).

We wondered whether SMYD2 inhibited the phosphorylation of IRF3 by interaction with IRF3. Therefore, we performed immunoprecipitation in the lysates of HEK293T cells transfected with tagged-*Irf3* and *Smyd2* by Flag antibody. The results showed the interaction between exogenous SMYD2 and IRF3 in HEK293T cells (Fig. 7A). The co-localization of SMYD2 and IRF3 was also confirmed in the above HEK293T cells (Fig. 7B). The interaction between endogenous SMYD2 and IRF3 was further identified in PMs with VSV infection (Fig. 7C). Co-IP assay showed that exogenous SMYD2 interacted with IRF3 (5D) but not RIGI or IKK-ε (Fig. 7D).

To identify the domains mediating the interaction between SMYD2 and IRF3, we performed immunoprecipitation in the lysates of HEK293T cells transfected with truncated *Smyd2* and wild-type *Irf3* plasmids. SMYD2Δ1-195aa interaction with IRF3 was significantly decreased, and SMYD2Δ1-251aa did not interact with IRF3 (Fig. 7E). These results were consistent with the data which showed that SMYD2Δ1-251aa had no influence on the transcriptional activity of IFN-β in luciferase activity assay (Fig. 6I). We further confirmed that deletion of SETi but not ET domain abolished the interaction between SMYD2 and IRF3 (Fig. 7F). Phosphorylated IRF3 was not inhibited in *Smyd2*^−/−^ PMs overexpressed with SETi-deleted mutant in comparison to wild-type and ET-deleted mutant (Fig. 7G, Supplementary Fig. 7D). Our luciferase activity assay also showed that SETi-deleted mutant of *Smyd2* had no effect on inhibiting TBK1-driven *Ifnb* activation (Fig. 7H). We then performed the same examination in HEK293T transfected with truncated *Irf3* and wild-type *Smyd2* plasmids, and we found that deletion of DBD (DNA-binding domain) (1-140aa) significantly decreased the interaction with SMYD2, and deletion of IAD domain abrogated the interaction between SMYD2 and IRF3 (Fig. 7I). Taken together, we demonstrated that the SETi domain of SMYD2 is responsible for its interaction with DBD and IAD domains of IRF3.

### SMYD2 inhibits phosphorylation of IRF3 by recruiting PP1

Our data showed that the activation of TBK1, the crucial kinase of IRF3, is not regulated by SMYD2, and IRF3 is not methylated by SMYD2 in PMs in response to VSV infection. The inhibition of IRF3 phosphorylation by SMYD2 is independent of upstream kinase activity or methylation of IRF3. What mechanism is involved in this process? Whether SMYD2 regulates the dephosphorylation of IRF3 by phosphatase? Phosphatases, SHP2, SHP1, PP1, and PP2A, have been reported in the regulation of antiviral IFN-I production. Moreover, PP1 and PP2A are reported to interact with IRF3 to inhibit IRF3 activation by dephosphorylation [7, 14, 23, 24]. Moreover, the interaction between SMYD2 and IRF3 was confirmed (Fig. 7A–I), so we wondered whether SMYD2 may promote the dephosphorylation of IRF3 by these phosphatases.

In order to identify the proteins interacting with SMYD2, mass spectrometry was performed after immunoprecipitation by SMYD2 antibody in lysate of peritoneal macrophages, and we did find that two catalytic subunits of PP1, Ppp1ca, and Ppp1cc, were precipitated by SMYD2 antibody (Fig. 7J, Supplementary Fig. 7E), which suggested that SMYD2 protein might promote dephosphorylation of IRF3 by interaction with phosphatase PP1. We then confirmed interaction between Flag-tagged SMYD2 and endogenous PP1α in RAW 264.7 cells (Fig. 7K), and found that the interaction between these two proteins was increased in response to VSV stimulation. Moreover, we performed co-IP analysis to confirm the interaction between endogenous SMYD2 and PP1α, and interaction between endogenous SMYD2 and PP1α was increased in response to VSV stimulation (Fig. 7L). We also found that expressions of PP1α and PP2A were similar between *Smyd2*^−/−^ and *Smyd2*^+/+^ PMs with or without VSV infection (Supplementary Fig. 7F). We speculated that SMYD2 protein promotes interaction between IRF3 and PP1, which leads to PP1-mediated dephosphorylation of IRF3. Therefore, we further performed co-IP analysis to clarify whether SMYD2 influences the interaction between IRF3 and PP1. The results showed that interaction between endogenous PP1α and IRF3 in macrophages infected with VSV was significantly inhibited after deletion of *Smyd2* (Fig. 7M). We further confirmed that SMYD2Δ1-195aa interaction with PP1 was significantly decreased, and SMYD2Δ1-251aa did not interact with PP1 (Fig. 7N), which suggested that SETi domain of SMYD2 mediated the interaction between SMYD2 and PP1. Taken together, our data showed that SMYD2 acted as scaffold to promote the inactivation of IRF3 by PP1.

Taken together, we have demonstrated that SMYD2 inhibits the production of antiviral IFN-I expression by inhibiting IRF3 phosphorylation through interaction with PP1, and the inhibitory process by SMYD2 does not depend on its methyltransferase activity (Supplementary Fig. 7G).

## Discussion

Innate immune response is effectively activated to protect the host from viral infection. The IRF3 activation is critical for host cells to effectively eliminate the virus by inducing IFN-I production [25]. However, the antiviral innate response is tightly regulated, for example, the IRF3 activation is timely and negatively regulated to prevent IFN-I overproduction so as to avoid immune injury to host [13, 26, 27]. In this study, we report that SMYD2 inhibits IRF3 phosphorylation by recruitment of PP1α, independent of its methyltransferase activity, to suppress the production of IFN-I in the antiviral innate immune response (Supplementary Fig. 7G). Our study identifies a new negative regulator of IRF3 activation, which might have potential significance in the control of infection and inflammation. In addition, the viral infection-induced reduction of SMYD2 in the early stage of innate response may provide new insight into the effective activation of antiviral innate immune response by relieving the suppressor.

SMYD3 is reported to methylate MAP3K2 at lysine 260 to activate Ras/Raf/MEK/ERK in driving tumorigenesis [28]. SMYD2, which is expressed in both cytoplasm and nucleus in cancer cells, is also investigated in its function on dimethylation of H3K4 or H3K36 to suppress cell proliferation [24]. SMYD2 also methylates non-histone targets, for example, SMYD2 mono-methylates K370 in p53 to reduce the DNA-binding efficiency of p53 [21], and this protein also methylates K810 of RB1 or K313 of PTEN to enhance cell cycle progression or inactivate tumor suppression function of PTEN, respectively [29, 30]. Our data showed that SMYD2 had crucial function in the inhibition of IFN-I production. In addition to the inhibition of SMYD2 by viral infection, we also found that SMYD2 expression was decreased by stimulation of both RNA and DNA virus, which suggested the broad significance of SMYD2 in the inhibition of innate immune response. We further confirmed that SMYD2 protein was decreased by IFN-β but not IL-6 and TNF-α stimulation, which indicates that inhibition of SMYD2 expression when the host cells need full activation to eliminate pathogens by relieving the suppression by suppressor.

Post-translational modifications (PTMs), such as methylation, play critical roles in multiple biological processes, including innate immune response [31]. Our original purpose of this study is to screen the protein methyltransferases, which have a function in the regulation of antiviral innate immune response by methylation of proteins. SMYD2 is confirmed as methyltransferase in multiple studies, which can methylate histone and non-histone proteins to regulate the function of these proteins [21, 22, 29, 30]. Abnormal function of SMYD2 is closely associated with cancer [32–34] and kidney disease [35]. Little is known about the function of SMYD2 in antiviral innate immune response. This is the first time to confirm that SMYD2 inhibits the production of IFN-I-independent of its methyltransferase activity, which is mediated with PP1α recruited by SMYD2 to dephosphorylate IRF3. Recent studies also clarify that the function of G9A in gastric cancer metastasis or the function of EZH2 in hematopoiesis is independent of their methyltransferase activity [36, 37]. Our data showed that SETi domain of SMYD2 was crucial for its negative function in innate immune response, while the methyltransferase activity is not necessary for this process. Therefore, we discovered the non-canonical function of SMYD2 in the inhibition of IFN-I production in macrophages in response to viral infection.

A previous study showed that the expressions of inflammatory cytokines IL-6 and TNF-α in bone marrow-derived macrophages are inhibited by SMYD2 in response to LPS stimulation, and SMYD2 is reported to modify the histone and inhibit NF-κB and ERK signaling pathways to regulate this process [38]. While this study did not clarify how SMYD2 inhibits the activation of these two signaling pathways. In our study, we also found the increased production IL-6 and TNF-α in peritoneal macrophages after deletion of *Smyd2*, and the activity of NF-κB signaling pathway was increased in *Smyd2*^*−/−*^ peritoneal macrophages infected with VSV. Maybe the mechanisms involved in negative regulation of IL-6 and TNF-α production by SMYD2 in macrophages are similar between LPS stimulation and VSV infection. Further work needs to be performed to clarify the detailed molecular mechanisms involved in these innate processes.

## Materials and methods

### Reagents

Anti-Rabbit SMYD2 (#9734), Anti-Mouse Lamin A/C (#4777), Anti-Rabbit RIG-I (#3743), Anti-Rabbit Phospho-IRF-3 (Ser396) (#4764), Anti-Rabbit TBK1 (#3013), Anti-Rabbit Phospho-TBK1/NAK (Ser172) (#5483), Anti-Rabbit MAVS (#4983), Anti-Rabbit NF-kappa-B p65 (#4764), Anti-Rabbit Phospho-NF-kappa-B p65 (Ser536) (#3033), Anti-Mouse Phospho-I-kappa-B-alpha (Ser32) (#2859), Anti-Rabbit IKK-alpha (#2682), Anti-Rabbit IKK-beta (#2684), Phospho-IKK-alpha (Ser176)/IKK-beta (Ser177) (#2078) and Anti-rabbit IgG-488 (#4412) were from Cell Signaling Technology. Anti-PP1-alpha (sc-7482) was from Santa Cruz. APC anti-mouse CD11c (117310) was from Biolegend. Anti-Rabbit PP2-alpha (610555), Percp-Rat Anti-Mouse CD4 (553052), FITC-Rat Anti-Mouse CD49b (553857) and PE Rat Anti-CD11b (553311) were from BD PharMingen. PE anti-mouse CD8a Antibody (100708), APC anti-mouse CD11c Antibody (117310), PerCP/Cy5.5 anti-mouse CD19 Antibody (115534), FITC anti-mouse Ly-6G (127606), FITC anti-mouse CD3 (100204), anti-mouse F/480 FITC (123107) and PE anti-mouse I-A/I-E (107608) were from Biolegend. Recombinant Mouse GM-CSF (415-ML-050) and Recombinant Mouse IL-4 Protein (404-ML-01M) were from R&D Systems. Murine M-CSF (315-02-1000) was from PeproTech. LYY507 was donated by the University of Oxford. Camptothecin (#13637) was from Cell Signaling Technology. ^3^H SAM (NET155H001MC) was from PerkinElmer.

### Mice

BAC recombineering was used to generate floxp-flanked *Smyd2* mice, and two floxps are located at the upstream and downstream intron of the 7^th^ extron respectively, which leads to frameshift mutation after crossing with mice carrying *Cre* gene. *Neo* gene flanked with Frt was used to screen out the recombined ES clones. Targeted ES cells were identified by Southern blot and were injected into C57BL/6 blastocysts to generate high-percentage chimeras. *Smyd2*-floxed allele mice (*Smyd2*^*f/+*^) was obtained after deletion of *Neo* gene by crossing between *Smyd2* chimeras with B6; SJL-Tg (ACTFLPe) 9205Dym/J mice (003800, the Jackson Laboratory) [14]. To delete *Smyd2* gene in all tissues of mice (*Smyd2*^*−/−*^*)*, *Smyd2*-floxed allele (*Smyd2*^*f/+*^) mice were crossed with129S/Sv-Tg (Prm-cre) 58Og/J mice (003328, the Jackson Laboratory), which has efficient recombination of a Cre target transgene in the male germ line, but not in other tissues. *Smyd2*^*f/f*^ littermate mice were used as controls. The genotyping primers were: deletion of *Neo* F: 5′ -AGGGTCCTTCTTGCTCTT-3′, deletion of *Neo* R: 5′ -CCTTGTCTATTTGGCTTCC-3′; *Smyd2*^*−/−*^F: 5′-GAATGTCATTGTGACCTACAA-3′, *Smyd2*^*−/−*^R: 5′-CCTTATCCTTGGTTGTACACT-3′. Mice were bred in pathogen-free conditions.

### Culture of cell lines

Mouse peritoneal macrophages (PMs) were recruited by Thioglycolate and cultured in endotoxin-free DMEM (GIBICO) medium with 10% (vol/vol) FCS (Invitrogen). BM-derived macrophages or dendritic cells from C57BL/6 mice were generated by culturing BM cells with M-CSF (50 ng/ml) or GM-CSF (10 ng/ml) plus IL-4 (1 ng/ml) for 5 days (Peprotech). The HEK293T and RAW 264.7 cell lines were from the American Type Culture Collection. The cells were cultured in endotoxin-free DMEM (GIBICO) supplemented with 10% FCS.

### Pathogens

VSV (Indiana Strain), SEV (Sendai virus), and HSV-1 virus were propagated and amplified as described previously [20]. *L. monocytogenes* (LM) was provided by Dr. H. Shen. (University of Pennsylvania School of Medicine, USA).

### In vivo animal experiments

Age- and gender-matched mice (6–8 weeks old) with the indicated genotype were randomly selected to establish animal models in vivo. Mice were intravenously injected with VSV (5 × 10^7^ pfu/g). At the indicated time, the serum, spleen, liver, lung, and PM cells of the mice were collected for further detection. The lungs of mice were excised, fixed with 4% paraformaldehyde, and embedded. Sections were then stained with hematoxylin and eosin. The titers of VSV in the liver, lung, and spleen were evaluated by TCID50 assay. Mice were intravenously injected with VSV (1 × 10^8^ pfu/g) for the mouse survival assay.

### Quantitative real-time PCR assay

Quantitative PCR analysis was performed by LightCycler (Roche) using a SYBR RT-PCR kit (Takara). Data were normalized to β-actin expression. Sequences of the primers for quantitative real-time RT-PCR are in Supplementary Table 1.

### RNA interference

SiRNAs (final concentration of 20 nM, Genepharma) were transfected in peritoneal macrophages using RNAi MAX reagent (Invitrogen) following the manufacturer’s instructions. qRT-PCR or western blot was performed to examine the efficiency of interference.

### Immunofluorescence

HEK293T cells transfected with constructs and peritoneal macrophages infected with or without VSV were plated on the glass coverslips in 24-well plates, and labeled with antibodies against Flag-tag, Myc-tag, SMYD2 or IRF3. Cells were subjected to microscopy analysis with the Olympus FV100 or Zeiss LSM780 confocal laser microscope.

### Nucleus and cytoplasm fraction isolation

Minute^TM^ cytoplasmic and nuclear extraction kit (SC003, Invent biotechnologies) was used according to the manufacturer’s instructions. Briefly, Cells lysed in cytoplasmic extraction buffer were placed on ice for 5 mins and then centrifuged. Transfer the supernatant (cytosol fraction) to a new tube. Add nuclear extraction buffer to the pellet, then immediately transfer the nuclear extract to the filter cartridge with collection tube, centrifuge 14,000–16,000 rpm for 30 seconds, and the liquid in the collection tube was nuclear extract.

### Plasmid constructs

Wild-type CDS or mutants of *Smyd2*, *Irf3,* or *Pp1a* amplified from cDNA of mouse macrophages were subcloned into pcDNA3.1 eukaryotic expression vectors, and then mutants of *Smyd2* and *Irf3* were generated. All constructs were confirmed by DNA sequencing.

### Transfection of cells

HEK293T cells (American Type Culture Collection) were transfected with JetPEI reagents (PolyPlus), and peritoneal macrophages or RAW 264.7 were transfected with FuGENE HD (Promega) according to the manufacturer’s instruction.

### Western blot and Immunoprecipitation

Cells were lysed with cell lysis buffer (CST) supplemented with a protease inhibitor cocktail (Millipore). The protein concentration of the extracts was measured with BCA assay (Pierce). Immunoblot and Immunoprecipitation assays were performed as described previously [20].

### ELISA

The concentration of IFN-β (42400-1, PBL), IFN-α (BMS6027-five, ebioscience), IL-6 (431308, Biolegend), and TNF-α (430908, Biolegend) in culture medium or the sera were measured according to the manufacturer’s instruction.

### Flow cytometry

Splenic cells were re-suspended at 1 × 10^6^/ml in 5% rat serum containing PBS, and surface staining with antibodies was performed. The cells were analyzed by flow cytometer (BD LSRFortessa^TM^).

### RNA-seq and analysis

Total RNA was isolated with Trizol from *Smyd2*^*+/+*^ and *Smyd2*^−/−^ peritoneal macrophages infected without or with VSV, and then RNA quantification and qualification were confirmed. A total amount of 3 μg RNA per sample was used as input material for the RNA sample preparations. Ribosomal RNA and rRNA-free residue were removed. Subsequently, sequencing libraries were generated using the rRNA-depleted RNA by NEBNext® Ultra™ Directional RNA Library Prep Kit for Illumina® (NEB, USA) following manufacturer’s recommendations. The libraries were sequenced on an Illumina Hiseq 4000 platform. Protein-coding and non-coding sequences independent of known annotations were effectively distinguished by CNCI (Coding-Non-Coding-Index) (v2) profiles adjoining nucleotide triplets. Cuffdiff (v2.1.1) was used to calculate FPKMs of both lncRNAs and coding genes in each sample. Differential expression analysis or GO and KEGG enrichment analysis were performed.

### Luciferase reporter assay

HEK293T cells plated in 96-well plate (4 × 10^4^ /well) were co-transfected with IFN-β reporter plasmid pRL-TK-Renilla luciferase plasmid and other plasmids according to experiment design. The luciferase activity in the cells was measured with a Dual-Luciferase Reporter Assay system according to the manufacturer’s instructions (Promega) by varioskan flash (Thermo). Renilla luciferase activity was used to normalize the firefly luciferase.

### CHIP

Peritoneal macrophages were treated with ChIP Assay kit (Beyotime P2078) according to the described protocol. After incubation with control IgG, H3K4me2, and H3K36me2 antibodies, Antibody–chromatin complexes were pulled down by magnetic protein G beads (Invitrogen). After cross-link reversal and proteinase K treatment, immunoprecipitated DNA extracted with phenol-chloroform and ethanol-precipitated was used to perform qRT-PCR. Primers used are in Supplementary Table 1.

### In vitro methyltransferase assay

Purified recombinant GST-SMYD2 protein was incubated with purified recombinant GST-IRF3 and 2μCi S-adenosyl-L-[methyl-^3^H] methionine (^3^H SAM) in SAM buffer (50 mM Tris-HCl at PH 8.8, 10 mM DTT, and 10 mM MgCl_2_) for 1 hour at 30 °C. GST protein was negative control, and incubation of GST-SMYD2 with p53 peptide and 2μCi S-adenosyl-L-[methyl-^3^H] methionine (^3^H SAM, PerkinElmer) was used as positive control. Then all samples were separated by sodium dodecyl sulfate-polyacrylamide gel electrophoresis (SDS-PAGE), visualized by Coomassie staining. After de-staining, the gel was submerged with EN^3^HANCE (PerkinElmer) for 1.5 h, and then exposed to Kodak XAR film after 30 days at −80 °C.

### Statistical analysis

Statistical significance between groups was determined by two-tailed Student’s *t* test. *P* < 0.05 were considered to be significant (**P < 0.01, ***P < 0.001). For mouse survival study, Kaplan–Meier survival curves were generated and analyzed for statistical significance with GraphPad Prism 4.0.

### Reporting summary

Further information on research design is available in the Nature Research Reporting Summary linked to this article.

## Supplementary information

Supplementary Information

Unprocessed gel and images

Reporting Summary

## Data Availability

All data needed to evaluate the conclusions in the paper are present in the paper or the Supplementary Materials. Further information and requests for resources and reagents should be directed to and will be fulfilled by the Lead Contact, Xuetao Cao (caoxt@immunol.org). The RNA high throughput sequencing data of this study are deposited in the Genome Sequence Archive at the National Genomics Data Center, China National Center for Bioinformation / Beijing Institute of Genomics, Chinese Academy of Sciences (GSA: CRA010740).
