# Supplementary material for: Lysine methyltransferase SMYD2 inhibits antiviral innate immunity by promoting IRF3 dephosphorylation
Source: Cell Death Dis. 2023 Sep 6;14(9):592. doi: 10.1038/s41419-023-06118-y (PMC10482964; doi:10.1038/s41419-023-06118-y)
Supplement: Supplementary file 1 — Supplementary Information [file 41419_2023_6118_MOESM1_ESM.docx]

**Supplementary Information**


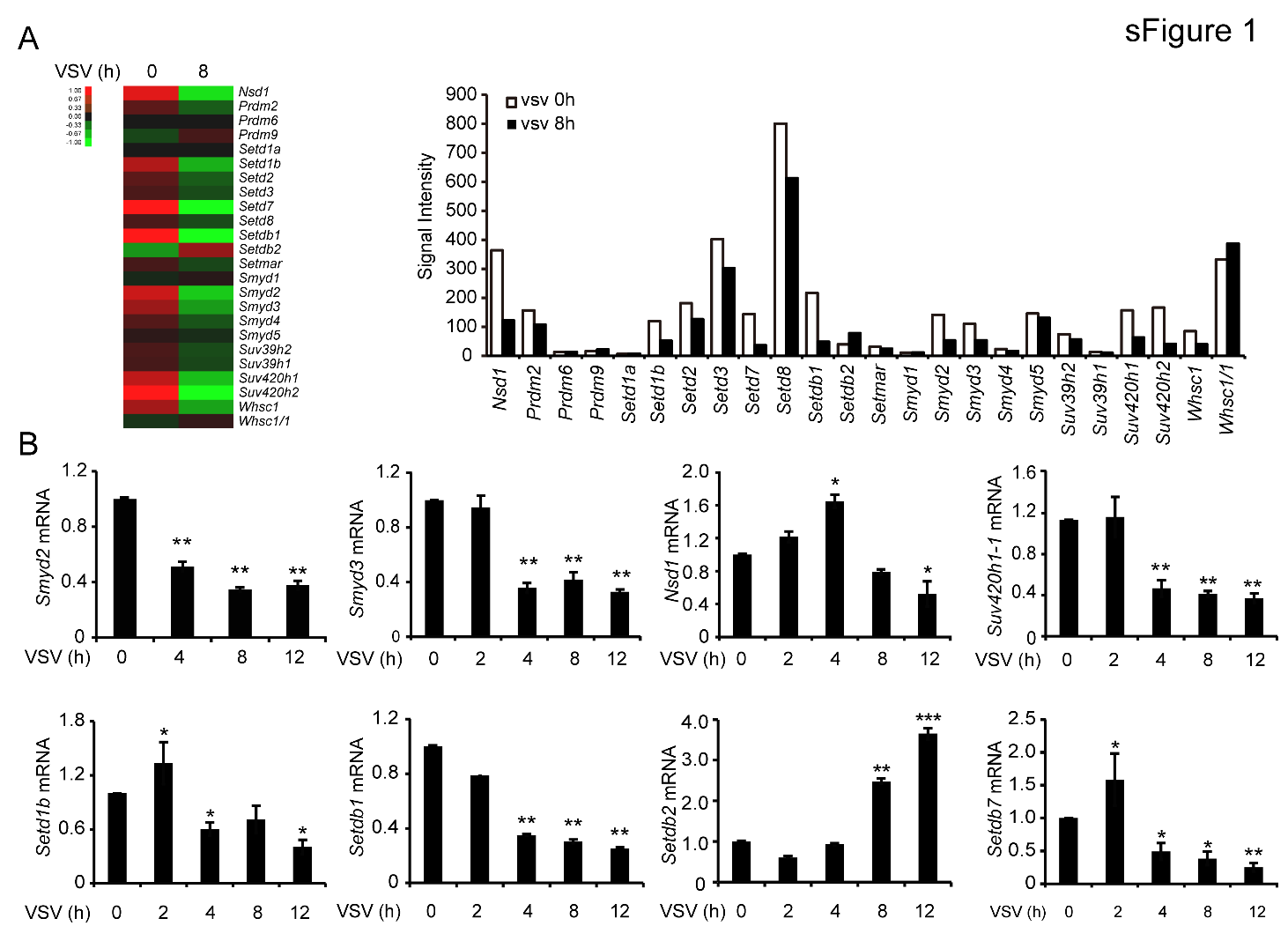


**Supplementary Figure 1.** ***Smyd2* expression is inhibited by VSV infection and inhibits IFN-β production. A** Differential mRNA expressions of protein lysine methyltransferases in peritoneal macrophages in response to VSV infection are analyzed by microarray, differential genes are shown in the heatmap (left) or histogram (right). **B** Increased-regulation or decreased-regulation of genes identified in microarray are confirmed in PMs infected for indicated time with VSV by qRT-PCR. Error bars represent s.d. Student’s t test. *p<0.05, **p<0.01, ***p<0.001. Data are representative of three independent experiments (**B**; mean ± s.d.).


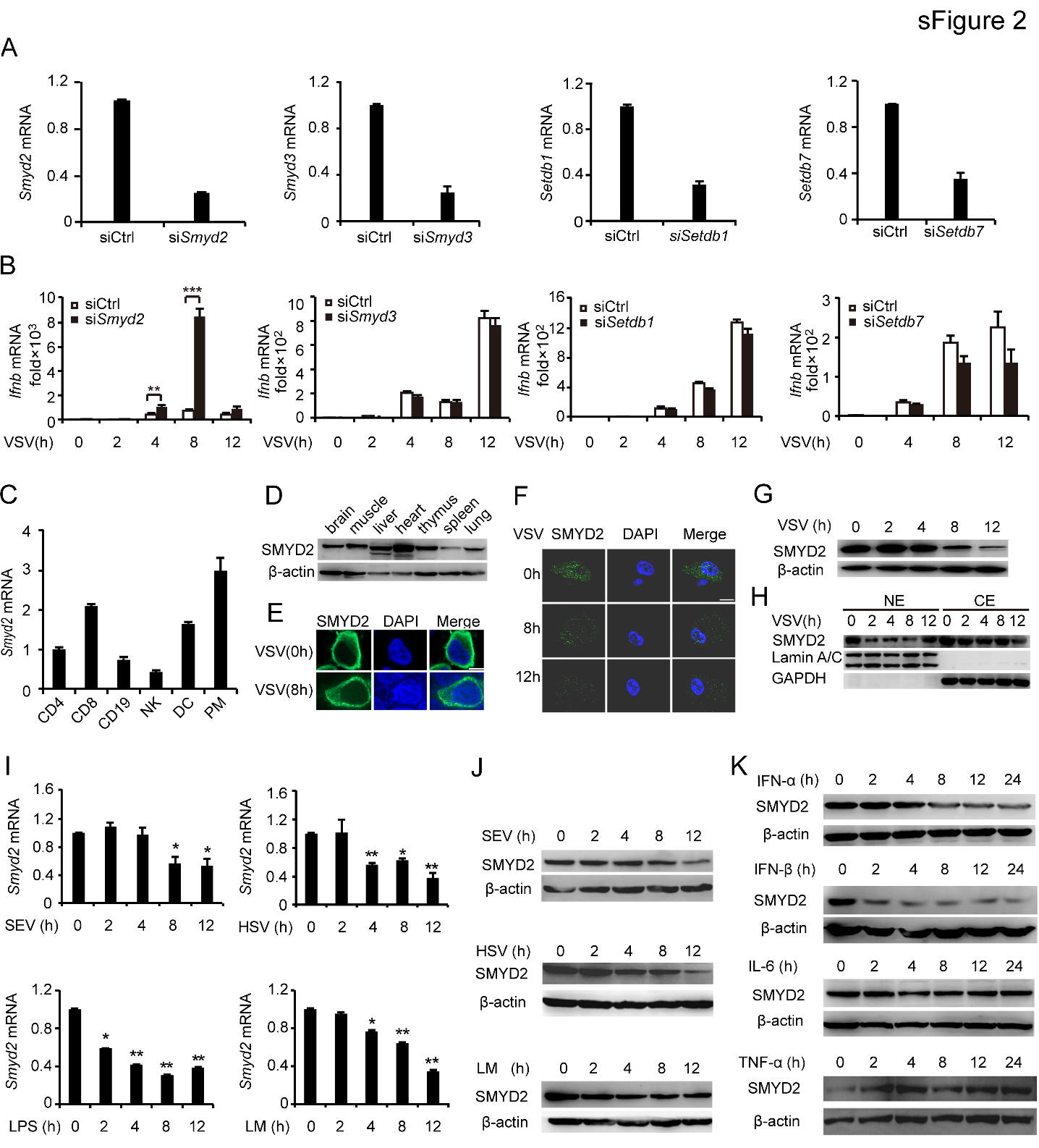


**Supplementary Figure 2. The efficiency of silence by specific siRNAs and decreased SMYD2 expression in macrophages in response to stimulation. A** qRT-PCR analysis of *Smyd2*, *Smyd3*, *Setdb1* and *Setdb7* expressions in peritoneal macrophages treated with according specific siRNAs compared with control. **B** IFN-β production is analyzed in the PMs silenced of *Smyd2*, *Smyd3*, *Setdb1* and *Setdb7* in response of VSV infection as indicated time. **C** *Smyd2* mRNA is analyzed in CD4^+^ T cell, CD8^+^ T cell, CD19^+^ B cell, NK, DC and PM by qRT-PCR. **D** SMYD2 protein is analyzed in organs by Western blot. **E** Analysis of FLAG**-**SMYD2 location in transfected 293T by immunofluorescence, DAPI for staining nucleus (scale bar: 5μm). **F** Location of endogenous SMYD2 in PMs is examined by immunofluorescence, DAPI for staining nucleus (scale bar: 10μm). **G** IB analysis SMYD2 protein in PMs infected with VSV as indicated time. **H** IB analysis SMYD2 protein in nucleus and cytoplasm isolated from PMs infected with VSV as indicated time. **I** qRT-PCR analysis of *Smyd2* mRNA in peritoneal macrophages stimulated with SEV, HSV, LPS or LM as indicated time. **J** Western blot analysis of SMYD2 protein in peritoneal macrophages stimulated with VSV as indicated time. **K** Western blot analysis of SMYD2 protein in peritoneal macrophages stimulated with IFN-α (300pg/ml), IFN-β (300pg/ml), IL-6 (100ng/ml) and TNF-α (100ng/ml) as indicated time. Error bars represent s.d. Student’s t test. *p<0.05, **p<0.01. All data are representative of three independent experiments. (**A, B, C, I**; mean ± s.d.).


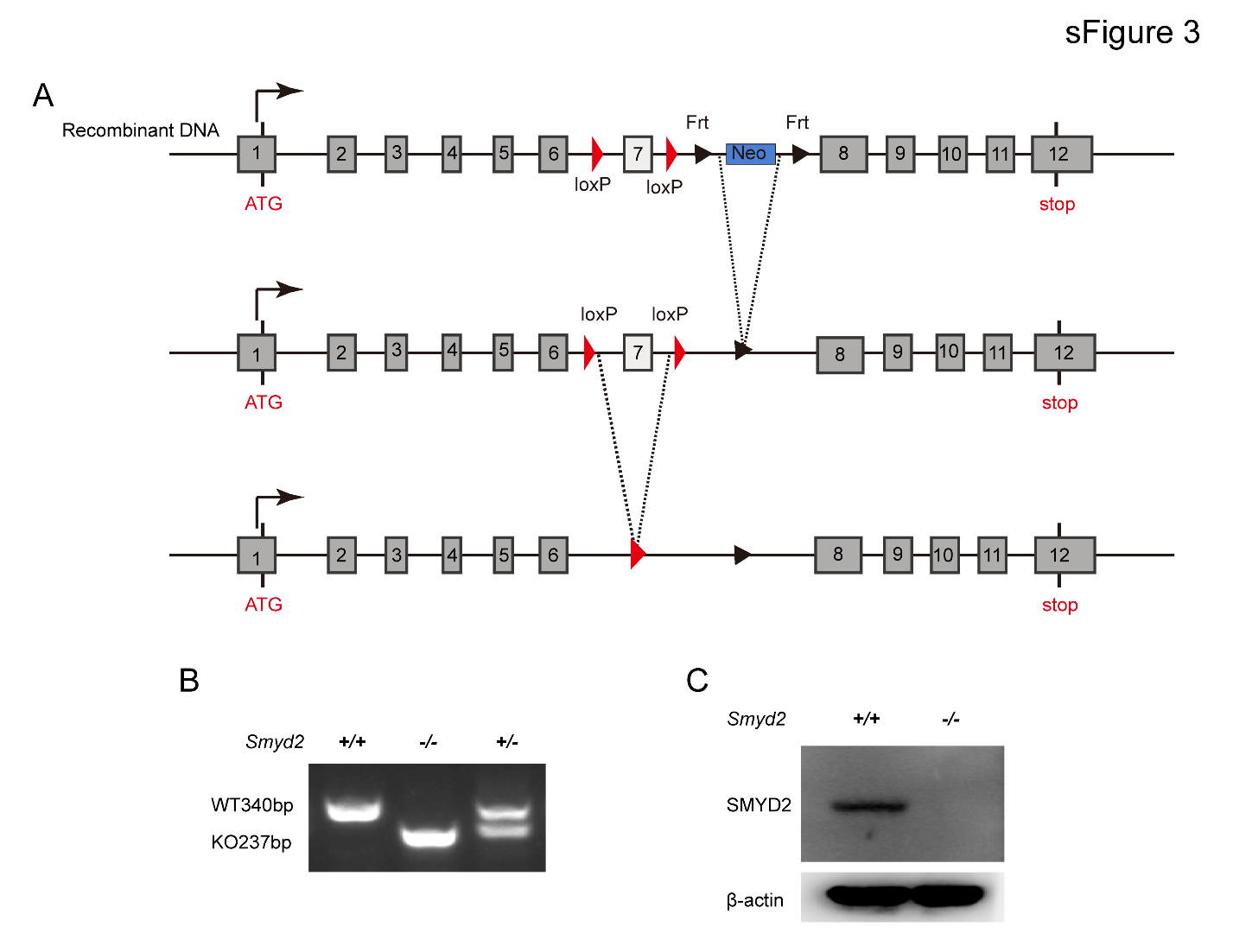


**Supplementary Figure 3. Generation of *Smyd2*-deficient mice. A** Schematic illustrating the generation of *Smyd2* knockout mice by deletion of 7^th^ exon. **B** The genotype of the mice analyzed by PCR; the size of the DNA fragments is showed in bp on the left side of the gel images. **C** Western blot analysis of SMYD2 expression in *Smyd2^+/+^* and *Smyd2^-/-^* mice.


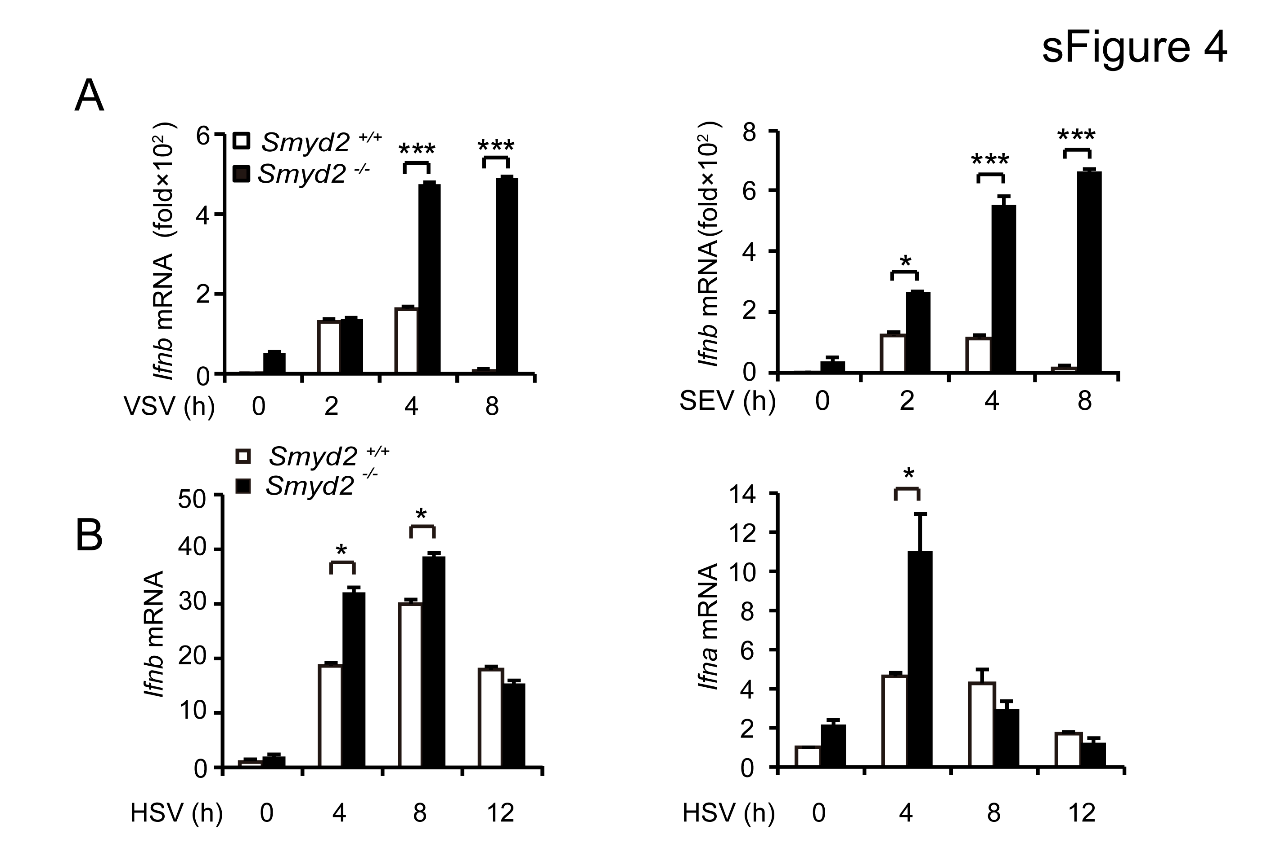


**Supplementary Figure 4. Increased expression of type Ⅰ interferon in *Smyd2^-/-^* dendritic cells.** **A, B** qRT-PCR analysis of *Ifnb* or *Ifna* mRNA in *Smyd2^-/-^* and *Smyd2^+/+^* dendritic cells infected with VSV, SEV (**A**) and peritoneal macrophages infected with HSV (**B**) as indicated time. Error bars represent s.d. Student’s t test. *p<0.05, **p<0.01, ***p<0.01. Data are representative of three independent experiments (**A, B**; mean ± s.d.).


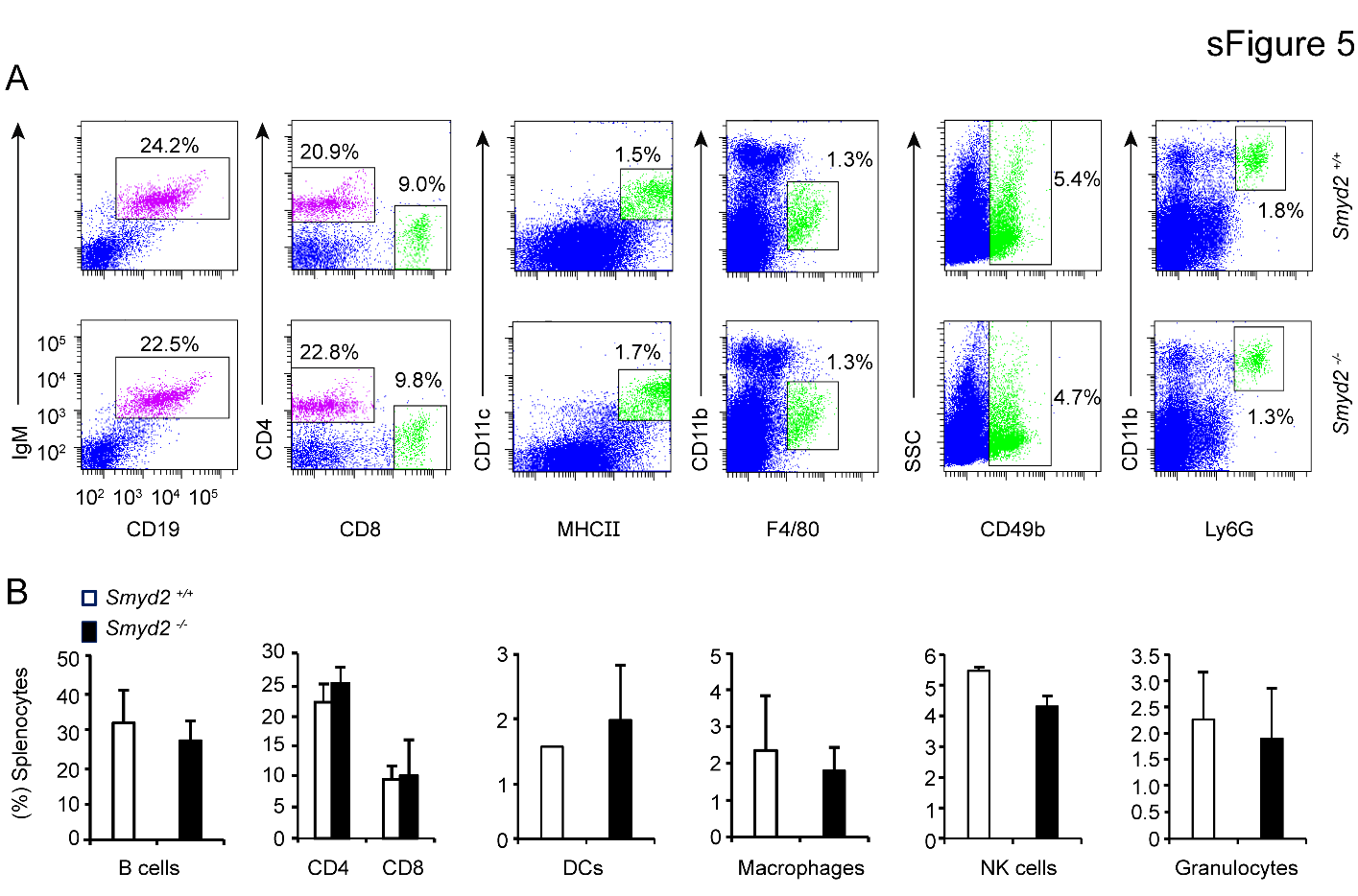


**Supplementary Figure 5. Deletion of *Smyd2* has no effect on the development of immune cells of spleen. A, B** Flow cytometry analysis of the ratio of immune cells, such as CD19^+^ B cells, CD4^+^ or CD8^+^ T cells, dendritic cells, macrophages, NK cells and neutrophil cells, in spleen of *Smyd2^-/-^* and *Smyd2^+/+^* mice (**A**). Statistical analysis (**B**) of the ratio of immune cells in (**A**). Error bars represent s.d. Student’s t test. Data are from three independent experiments (**B**; mean ± s.d.).


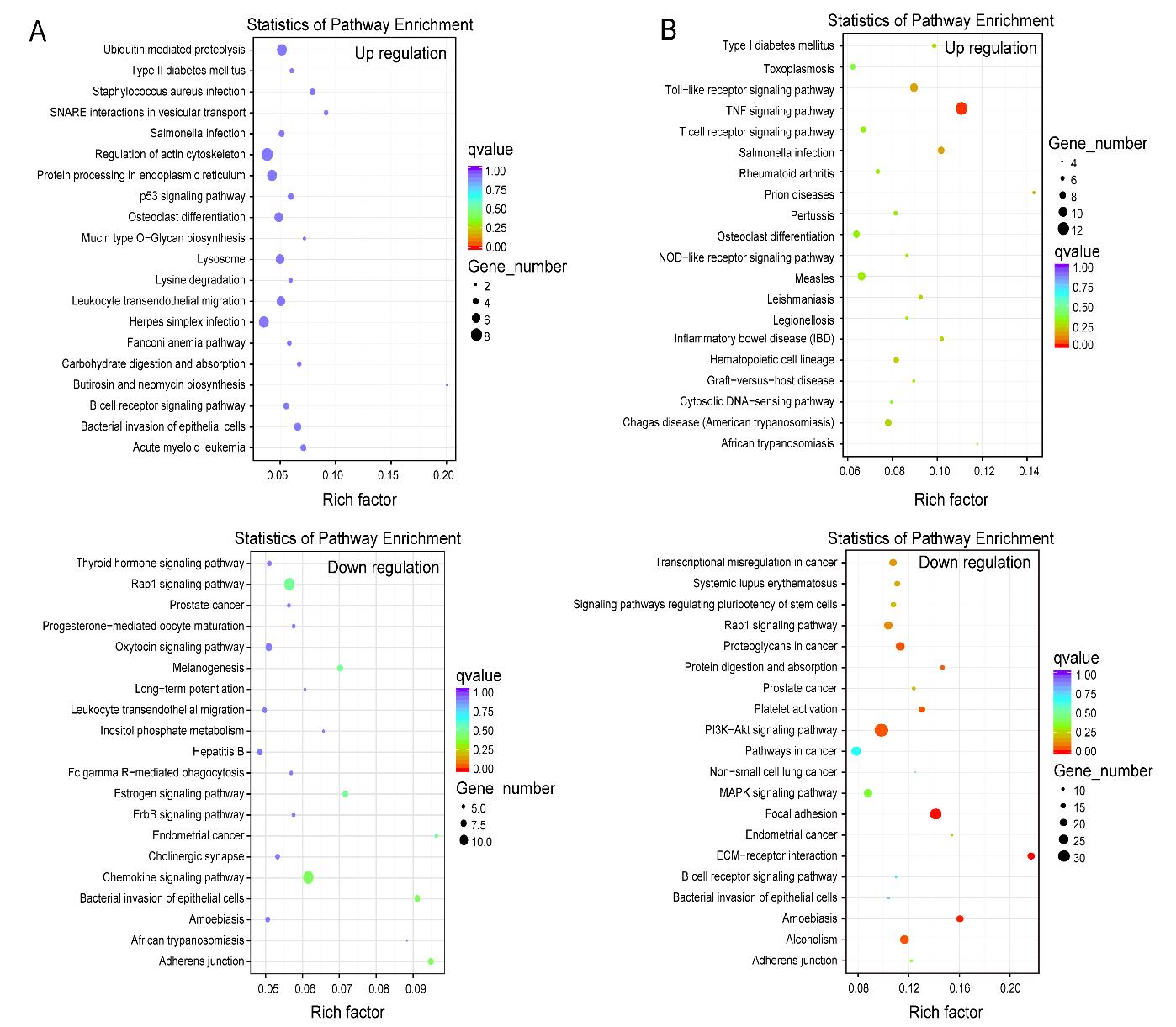


**Supplementary Figure 6. Screening of differentially expressed genes between *Smyd2^-/-^* and *Smyd2^+/+^* PMs infected without or with VSV by transcriptome sequencing**. **A** Enriched- GO (Gene Ontology) analysis of differential genes of transcriptome sequencing classified to biological processes. **B** KEGG (Kyoto Encyclopedia of Genes and Genomes) analysis of differential genes of transcriptome sequencing involved in signaling pathways.


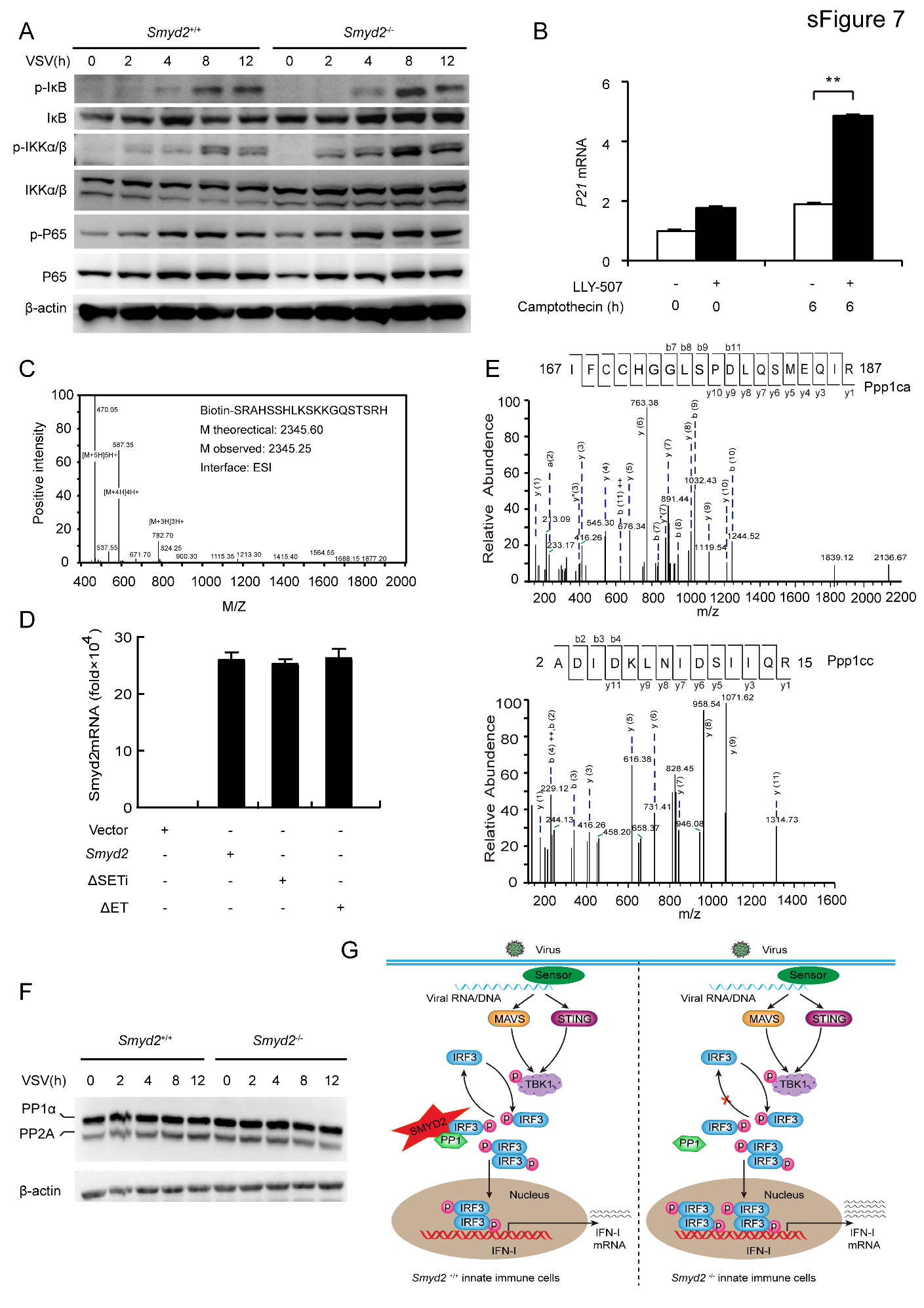


**Supplementary Figure 7. Increased Phosphorylation of NF-κB signaling pathway in *Smyd2-*deficient PMs in response to VSV infection.** **A** IB analysis of phosphorylation of critical molecules involved in NF-κB signaling pathway in the lysates of cells *Smyd2*^-/-^ and *Smyd2*^+/+^ PMs infected with VSV as indicated time. **B** qRT-PCR analysis of *P21* mRNA in Hela cells, which was pretreated without or with LYY-507 for 2 hours and then stimulated without or with camptothecin as indicated time. **C** Synthesis of p53 peptide Biotin-SRAHSSHLKSKKGQSTSRH, which includes the K370 methylated by SMYD2. **D** qRT-PCR analysis of *Smyd2* mRNA in *Smyd2*^-/-^ PMs overexpressed with wild type and mutants of *Smyd2* without VSV. **E** Peptides of Ppp1ca and Ppp1cc identified by mass spectrometry from bands indicated with arrow from **Figure 7J**. **F** IB analysis of PP1α and PP2A expression in the lysates of cells *Smyd2*^-/-^ and *Smyd2*^+/+^ PMs infected with VSV as indicated time. **G** Model of the function of SMYD2 on inhibiting IRF3 phosphorylation by recruiting phosphatase PP1. Error bars represent s.d. Student’s t test. **p<0.01. Data are representative of three independent experiments (**B, D**; mean ± s.d.).

**Supplementary Table 1. Sequence of primers used for qRT-PCR or CHIP.**

| **Gene Symbol** | **Primers (5’-3’)** | |
| --- | --- | --- |
|  | **Forward** | **Reverse** |
| *Smyd2* | AACAGAAGACAGGAACGACCG | CGATGACATTGCGTGCGTAT |
| ΔSETi | CTGCCTGTACATGCAGGACT | TTCCCAGCTTCAGCCACATG |
| ΔET | CTGCCTGTACATGCAGGACT | TTCCCAGCTTCAGCCACATG |
| *Smyd3* | TGCTCCATCGTATTCAACGG | ACCTCCTTCCATATTTGCTC |
| *Nsd1* | GTGAGACACAGAAGTGGTCG | GTGACAATGGGTTGATTCTT |
| *Suv420h1-1* | GGCTTAAAAAGTTAGGTGACA | TGGGTGAGGTAGAGTTGGAA |
| *Setd1b* | TGAGGGAGAAACGGTATGAG | GTTGATGTGCTGCTTAGAGT |
| *Setdb1* | GCTACCTCAATCACAGTTGC | CCACTTCGTAGTTGTAGTCC |
| *Setdb2* | AGGCTCATACCTACTGGCAT | AATGTCCCTTTGTCAATGTC |
| *Setdb7* | GGCGTTTACACCTATGAG | TCCTCTGGTCAGGGTACACA |
| *Ifnb* | ATGAGTGGTGGTTGCAGGC | TGACCTTTCAAATGCAGTAGATTCA |
| *Ifna* | ATCCCAGACACAAGCAGAGAG | GGCTGTGGGTTTGAGTCTTCT |
| *Il6* | TAGTCCTTCCTACCCCAATTTCC | TTGGTCCTTAGCCACTCCTTC |
| *Tnfα* | AAGCCTGTAGCCCACGTCGTA | GGCACCACTAGTTGGTTGTCTTTG |
| VSV | ACGGCGTACTTCCAGATGG | CTCGGTTCAAGATCCAGGT |
| *Ifit1* | CTGAGATGTCACTTCACATGGAA | GTGCATCCCCAATGGGTTCT |
| *Isg15* | GGTGTCCGTGACTAACTCCAT | TGGAAAGGGTAAGACCGTCCT |
| *Cxcl10* | CCAAGTGCTGCCGTCATTTTC | GGCTCGCAGGGATGATTTCAA |
| *Ccl5* | TGCAGAGGACTCTGAGACAGC | GAGTGGTGTCCGAGCCATA |
| *Ifnb* (CHIP) | TAACCCAGTACATAGCATATA | AGTGAGAATGATCTTCCTTCAT |
| *β-actin* | AGTGTGACGTTGACATCCGT | GCAGCTCAGTAACAGTCCGC |
