# Supplementary material for: Lysine methyltransferase SMYD2 inhibits antiviral innate immunity by promoting IRF3 dephosphorylation
Source: Cell Death Dis. 2023 Sep 6;14(9):592. doi: 10.1038/s41419-023-06118-y (PMC10482964; doi:10.1038/s41419-023-06118-y)

Fig. 1

E

β-actin

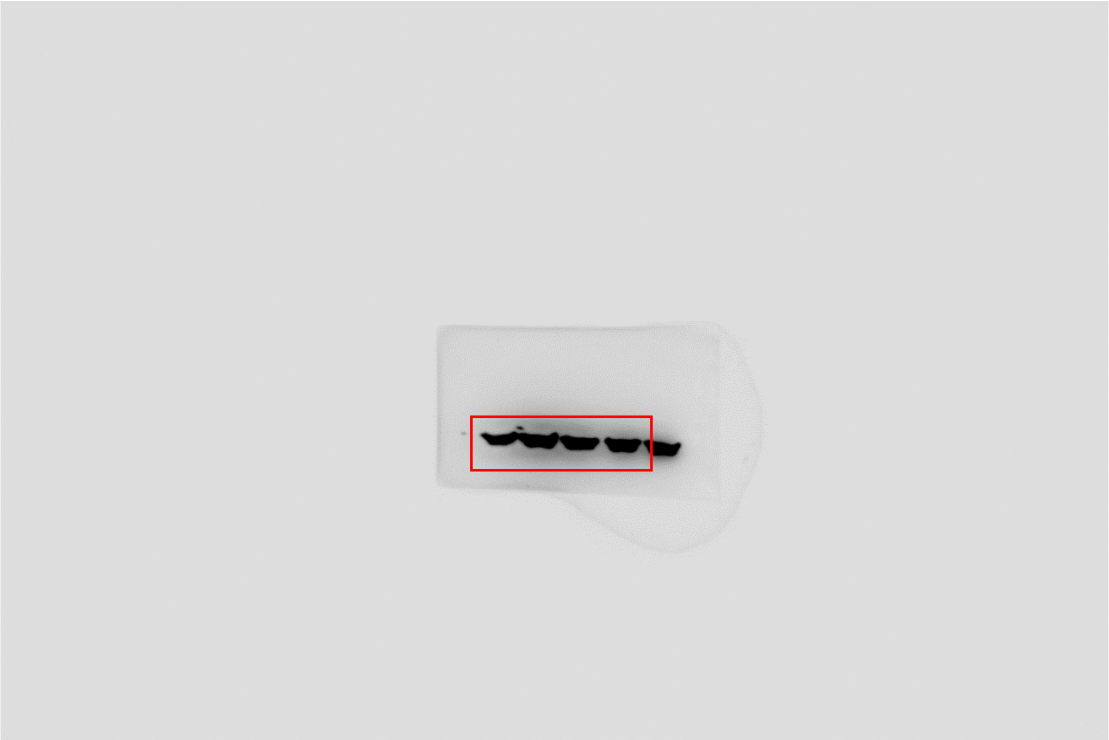

Flag

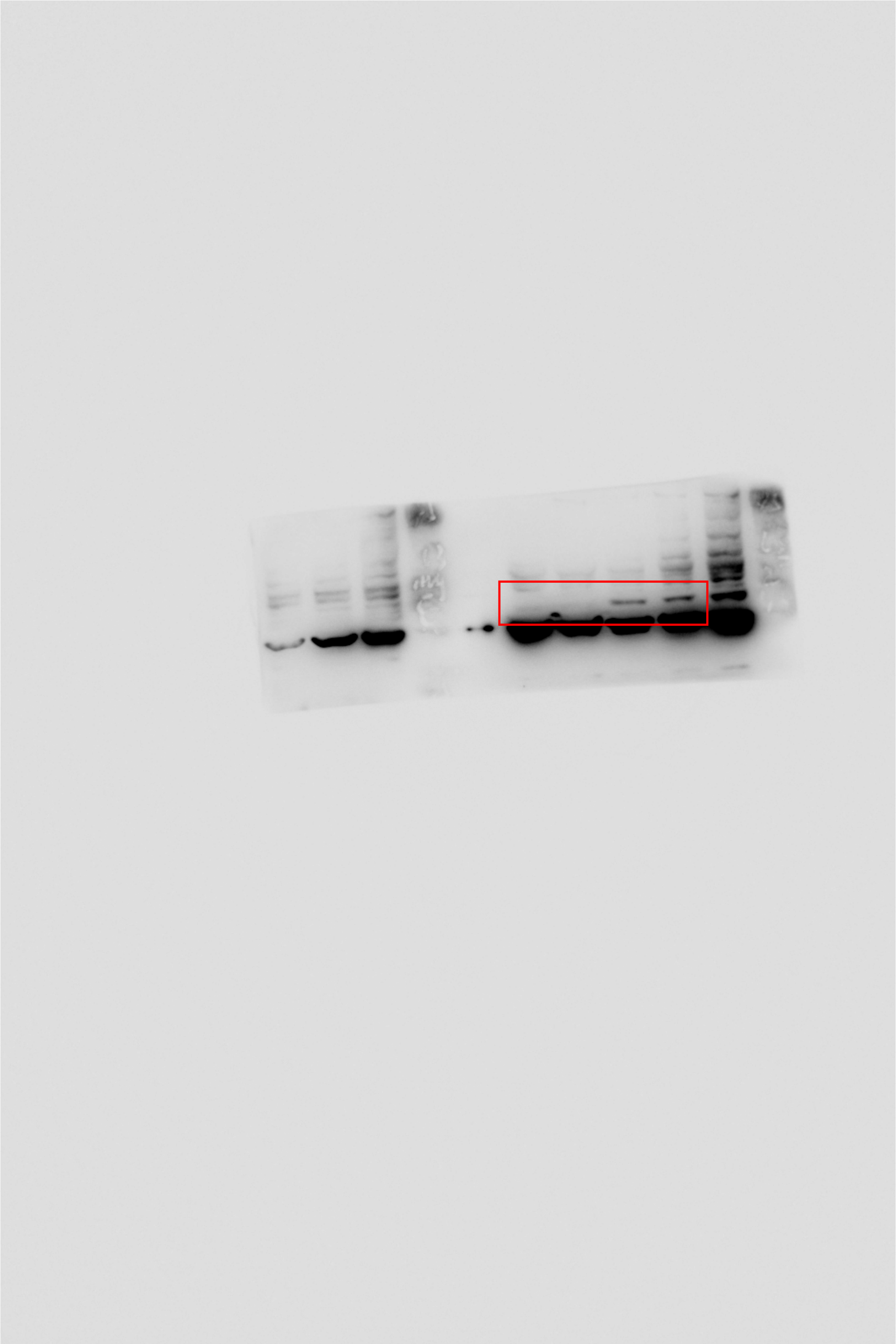

Fig. 2

E

Smyd2$^{-/-}$

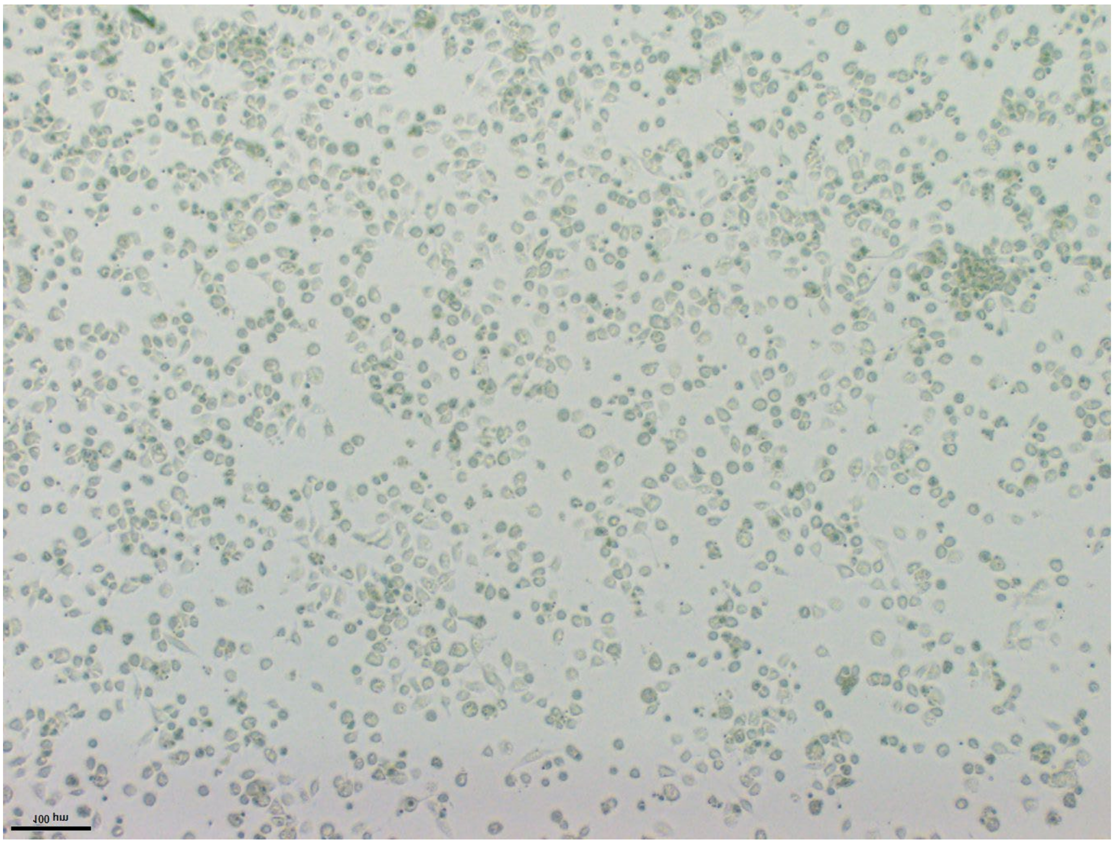

Smyd2$^{-/-}$

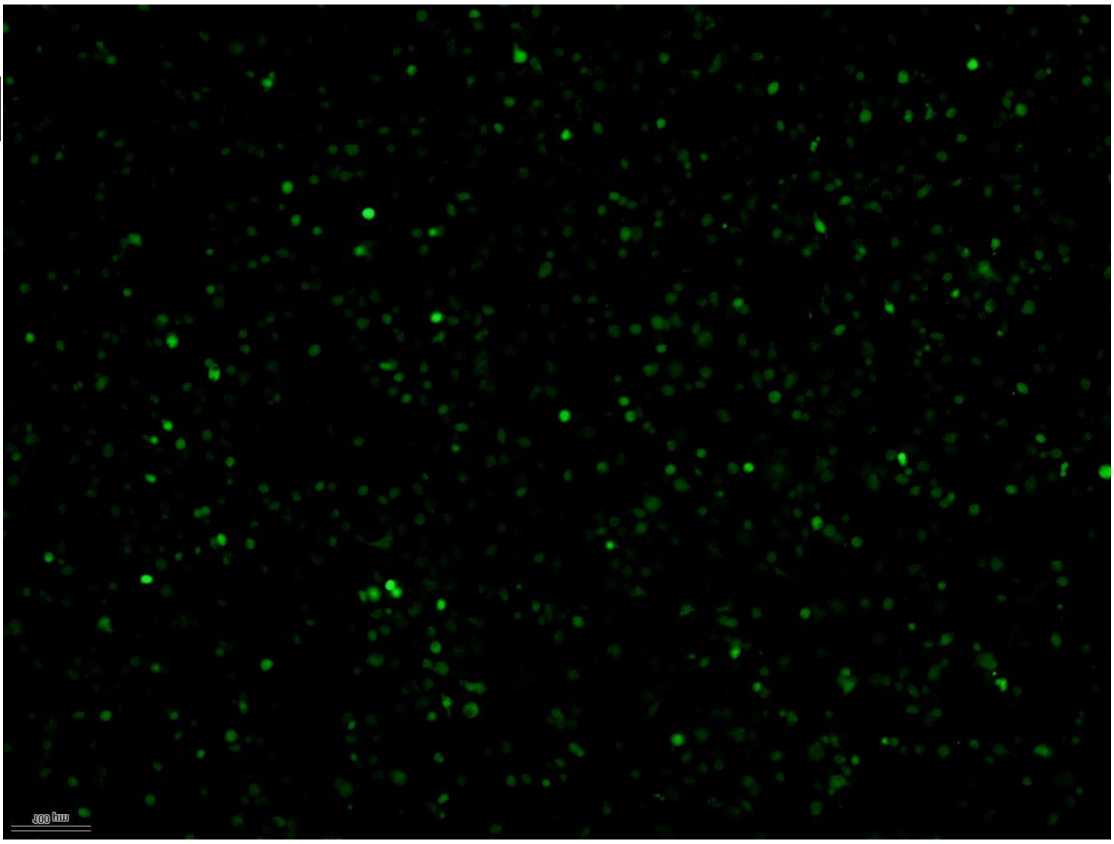

Smyd2$^{+/+}$

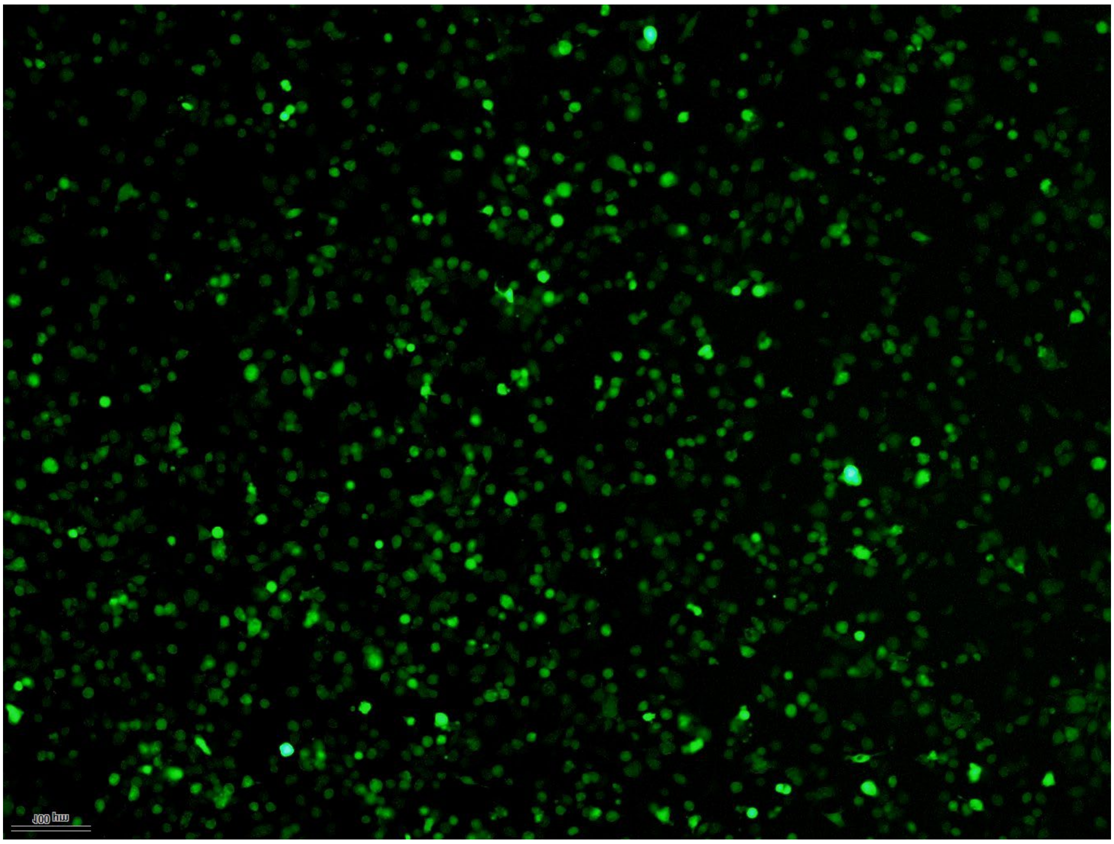

Smyd2$^{+/+}$

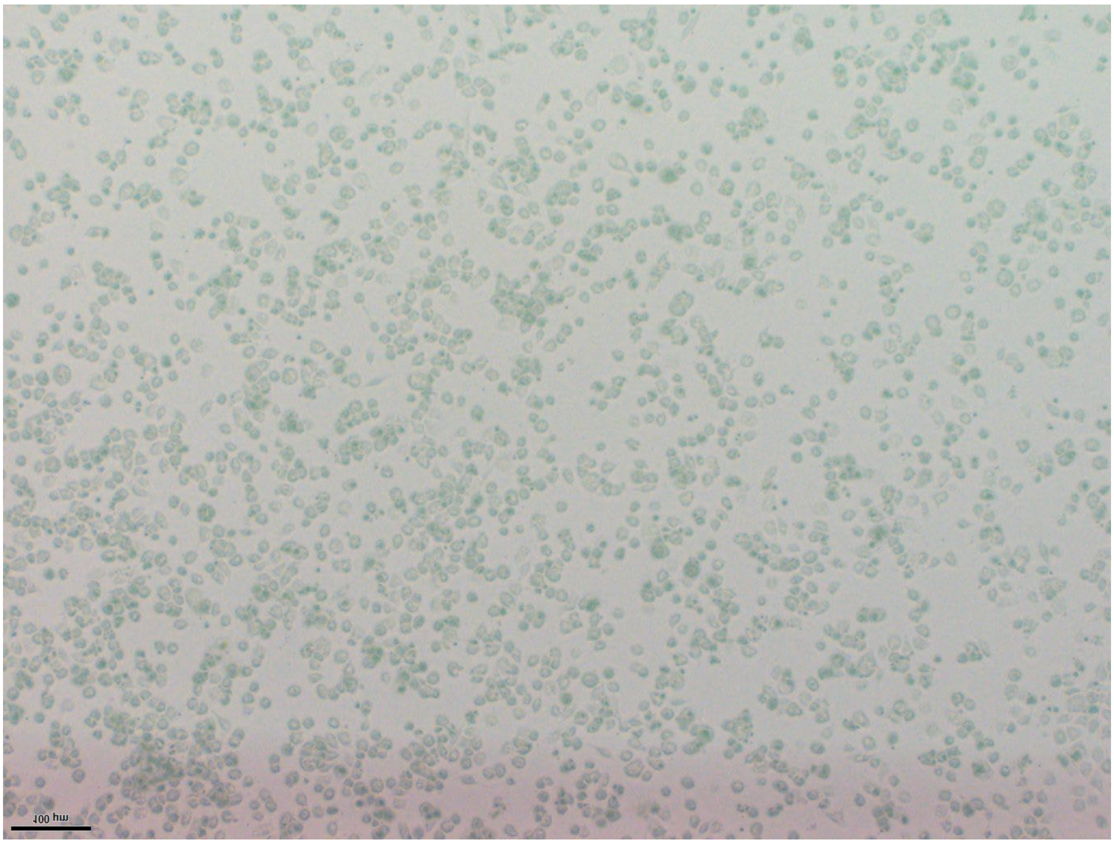

Fig. 3

D

Smyd2$^{+/+}$
PBS

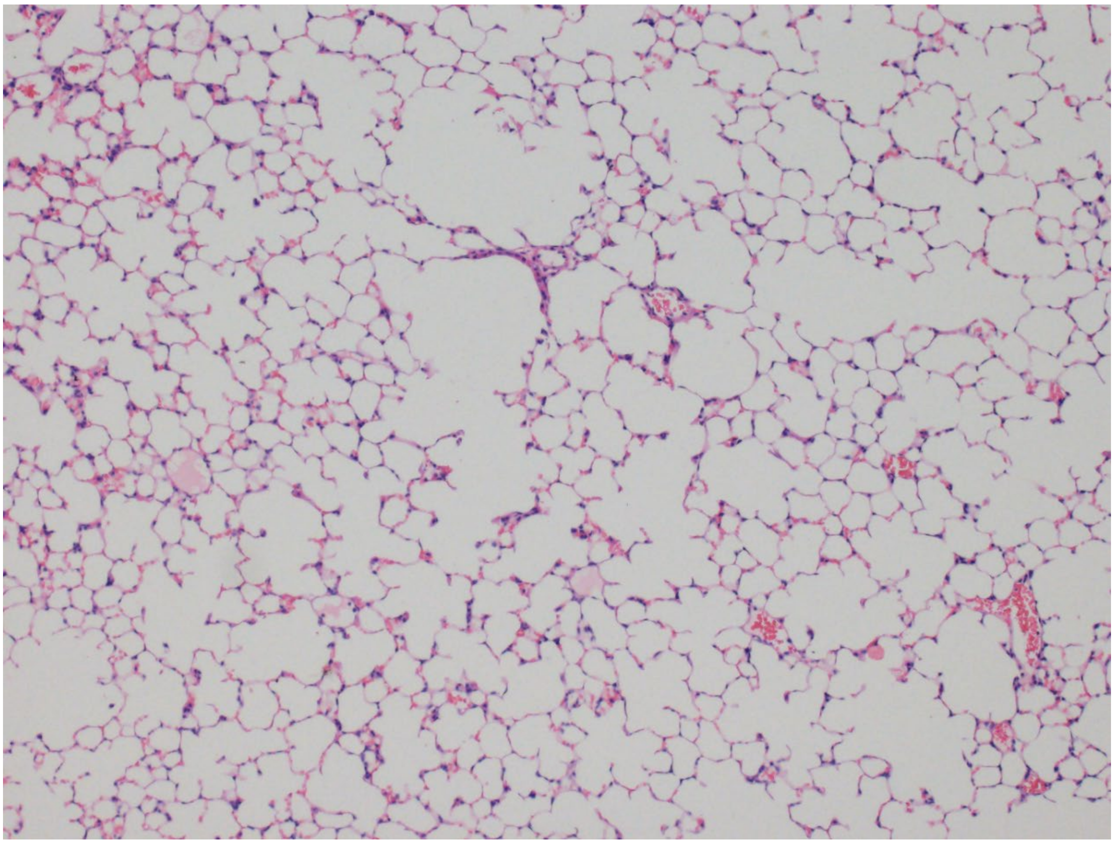

Smyd2$^{+/+}$
VSV

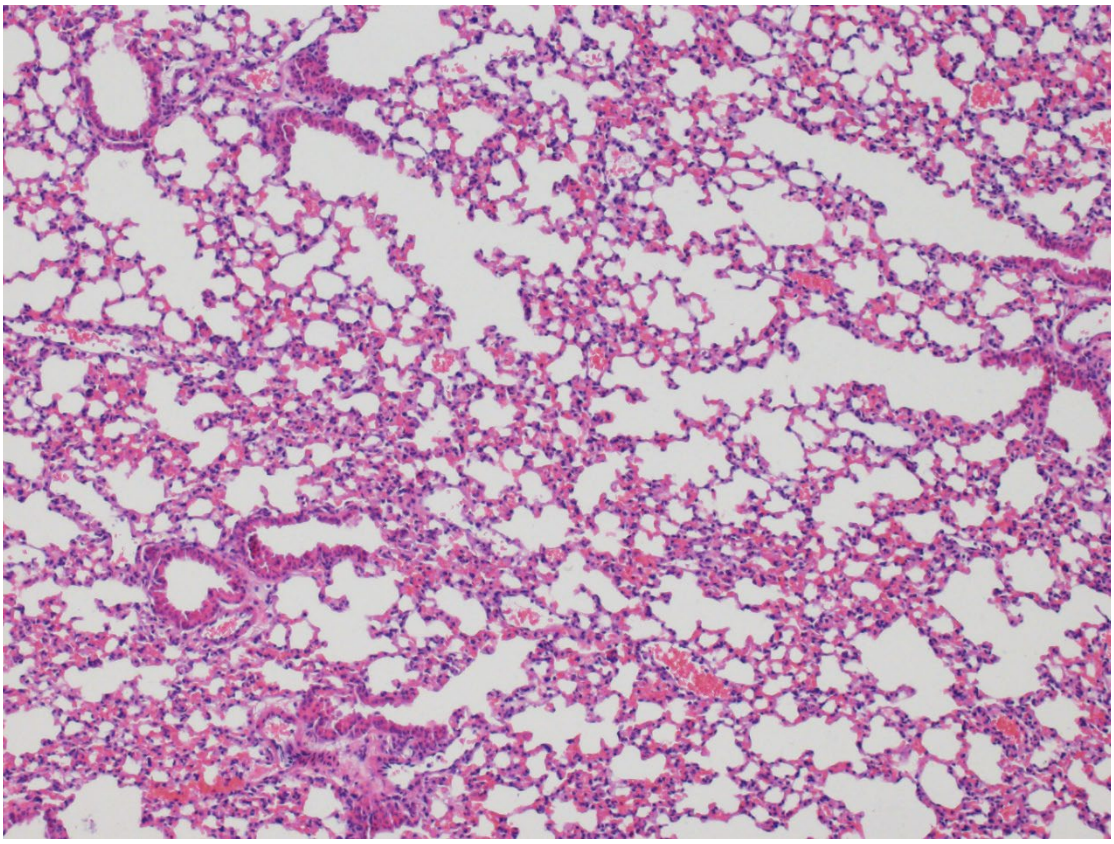

Smyd2$^{-/-}$
PBS

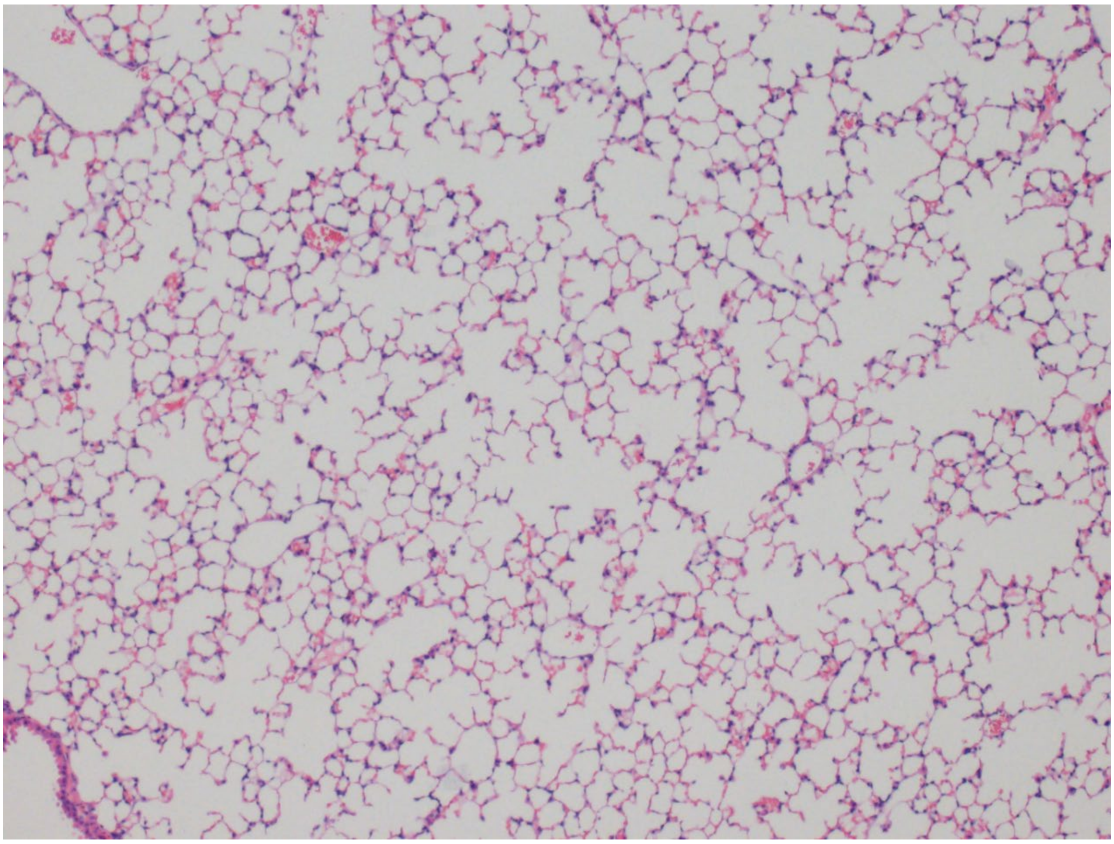

Smyd2$^{-/-}$
VSV

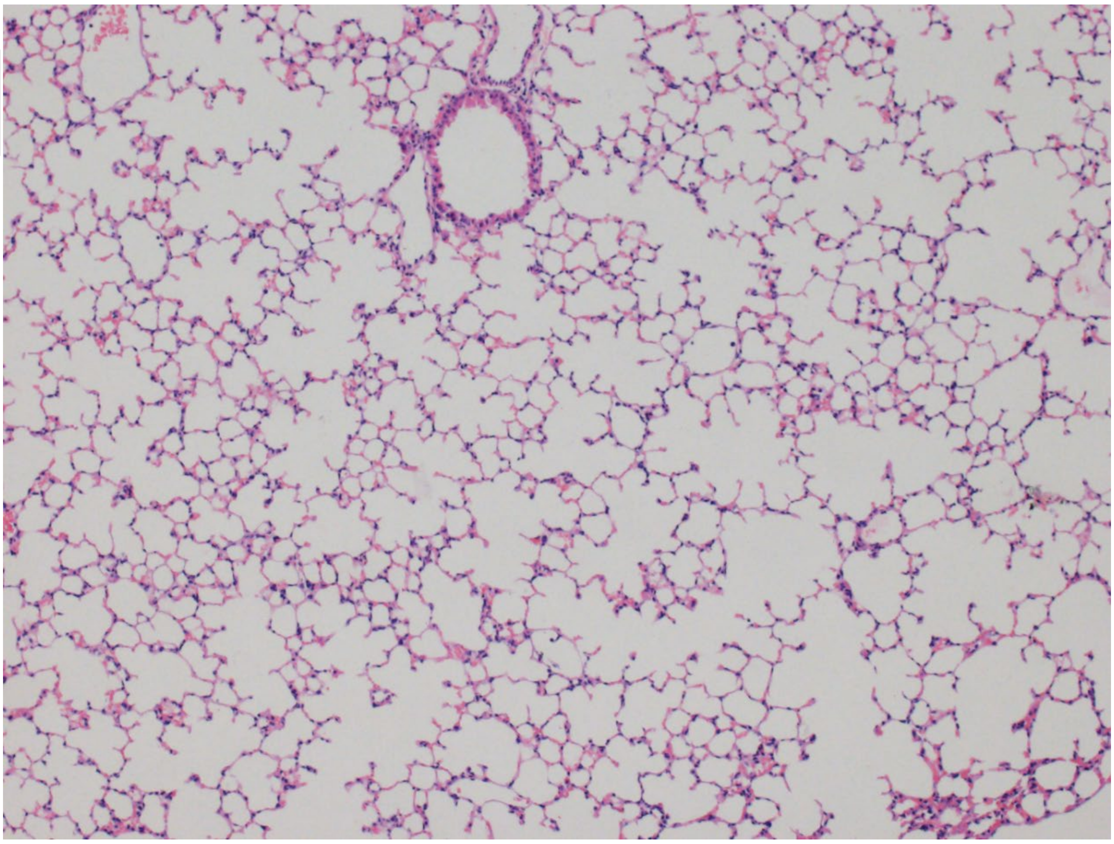

Fig. 5

A

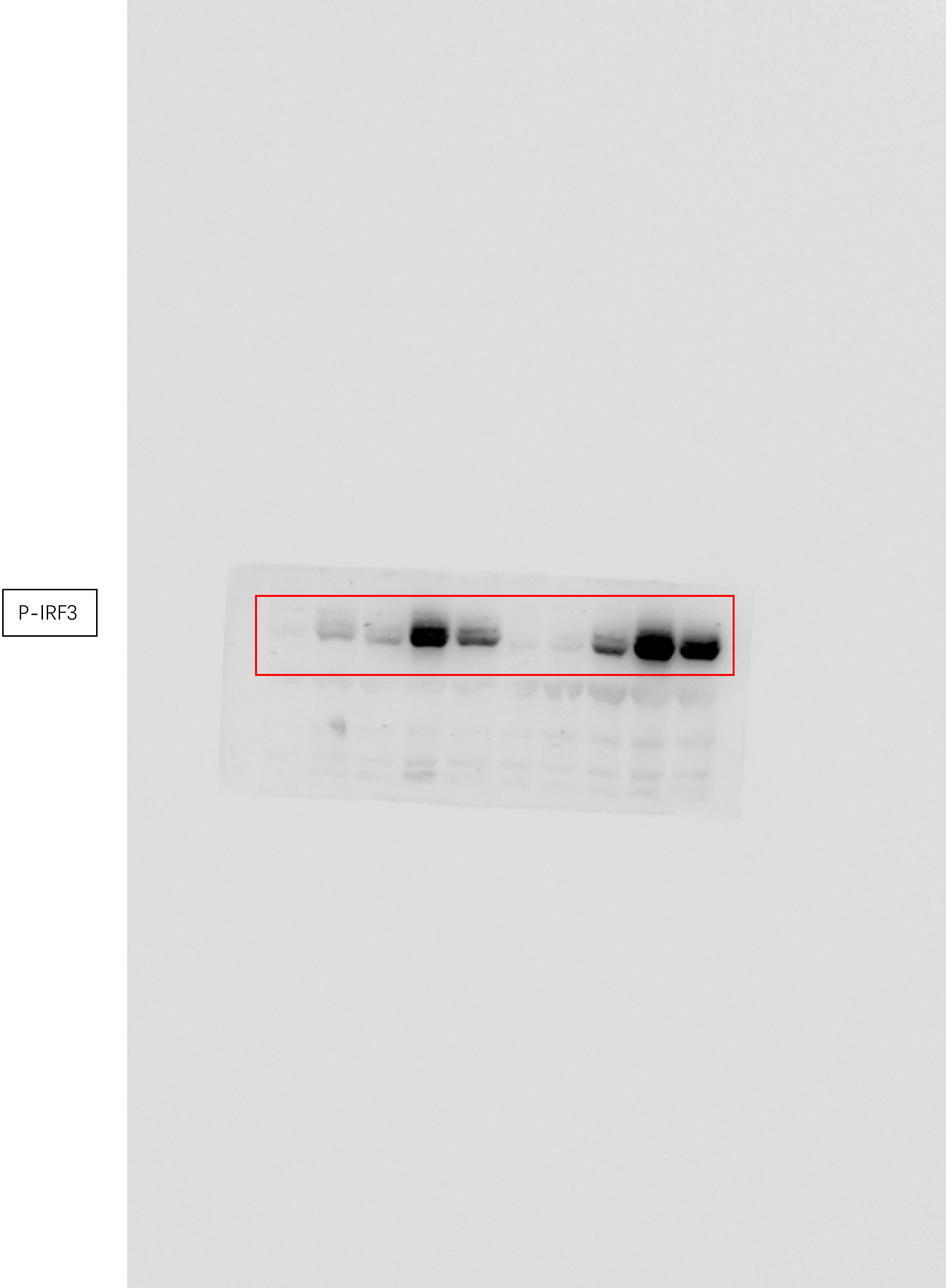

RIGI

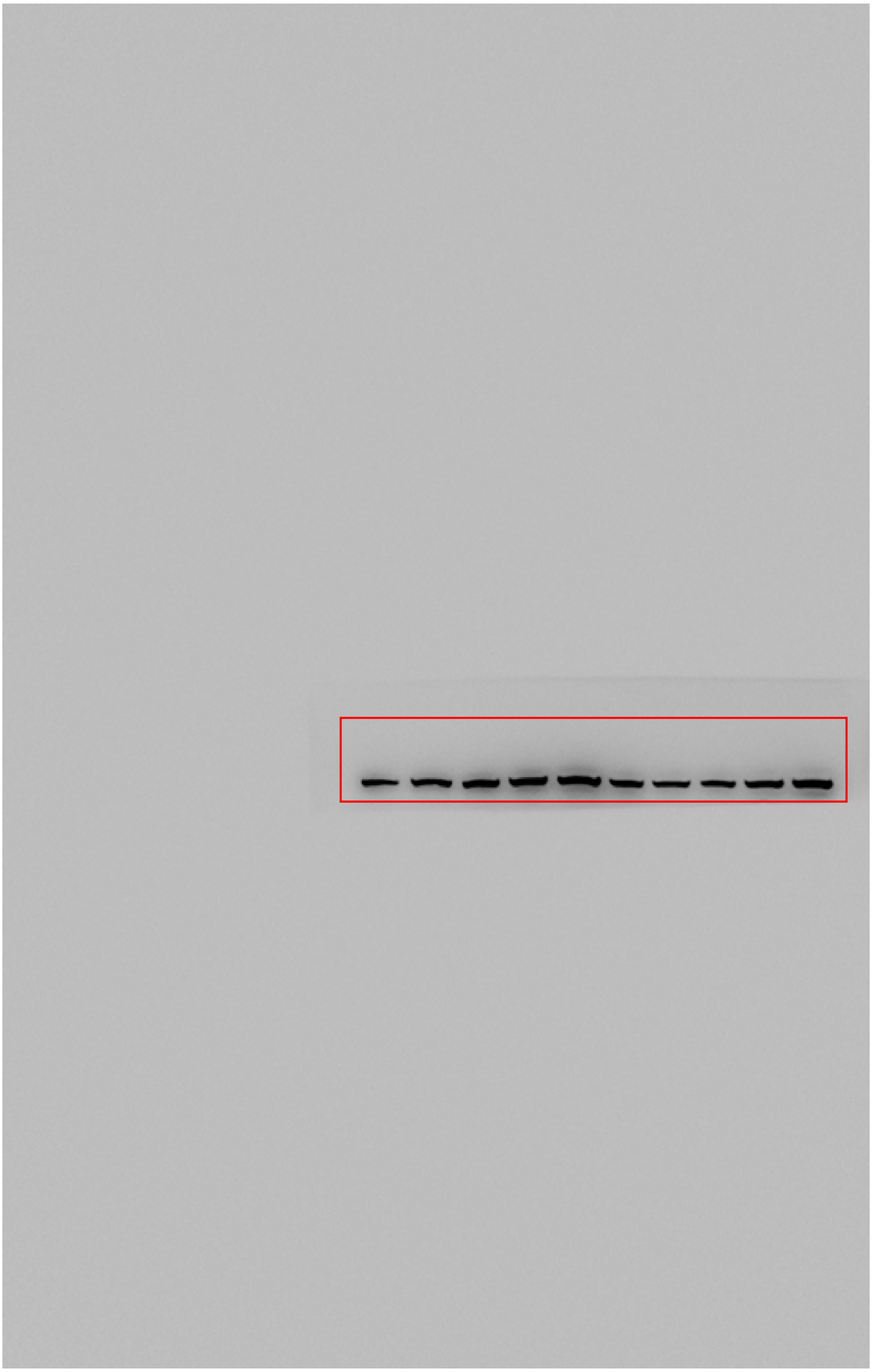

IRF3

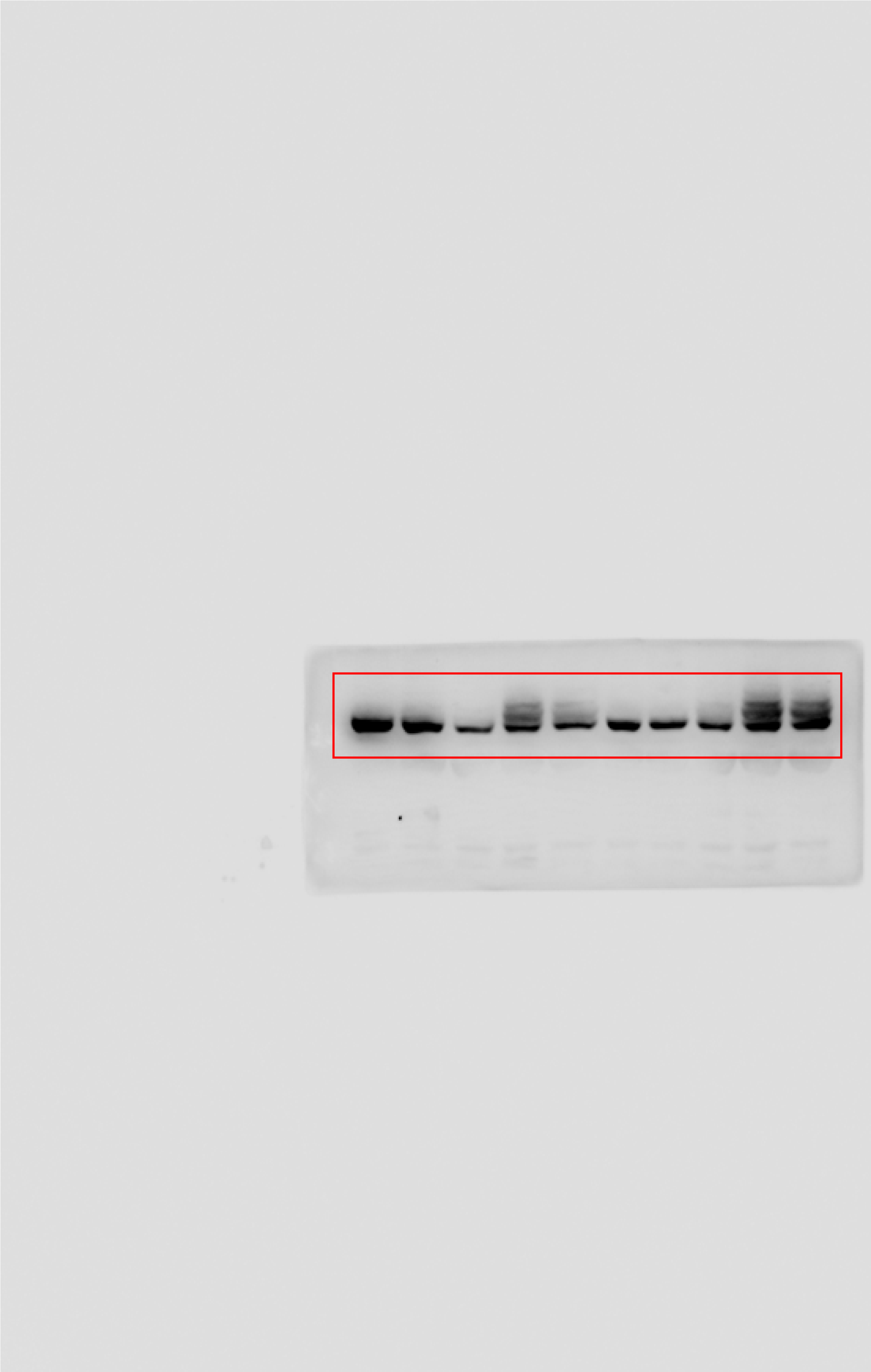

MAVS

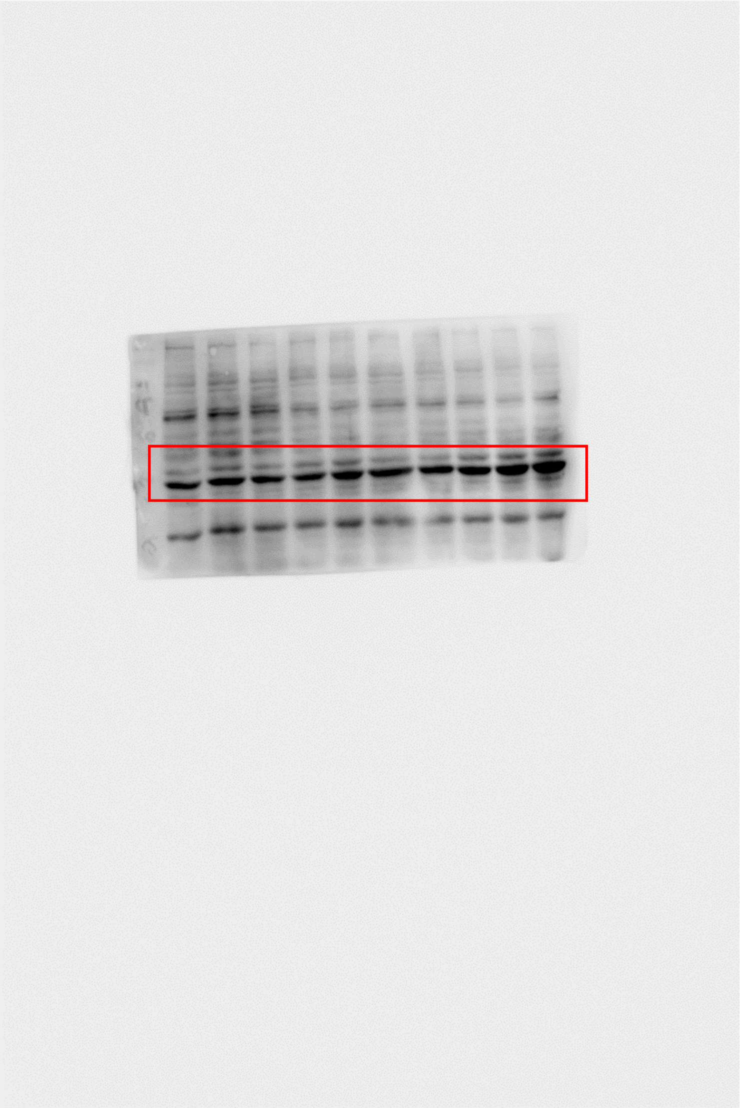

P-TBK1

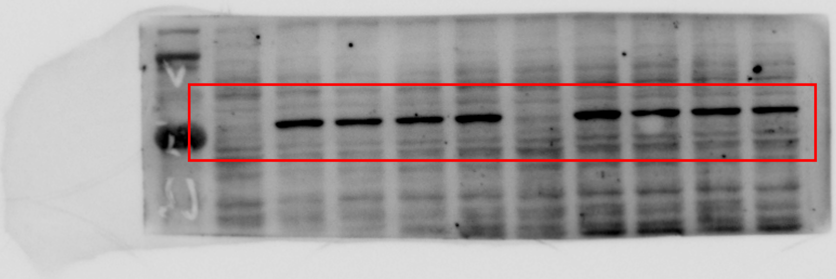

TBK1

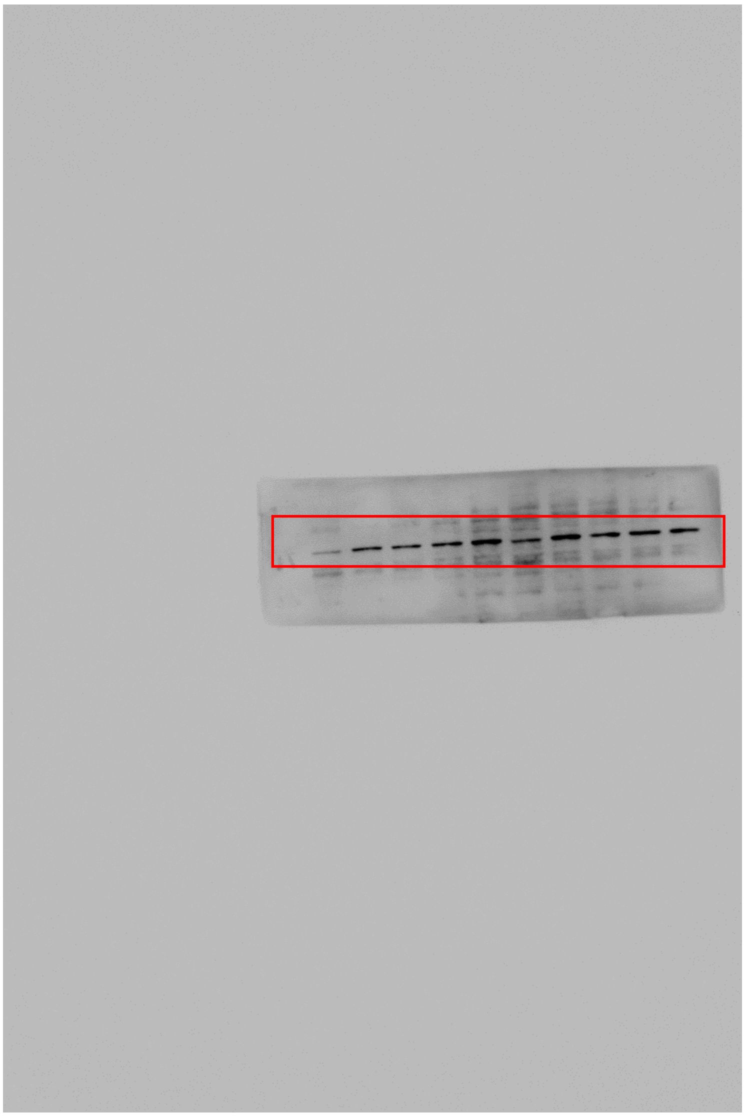

TRAF3

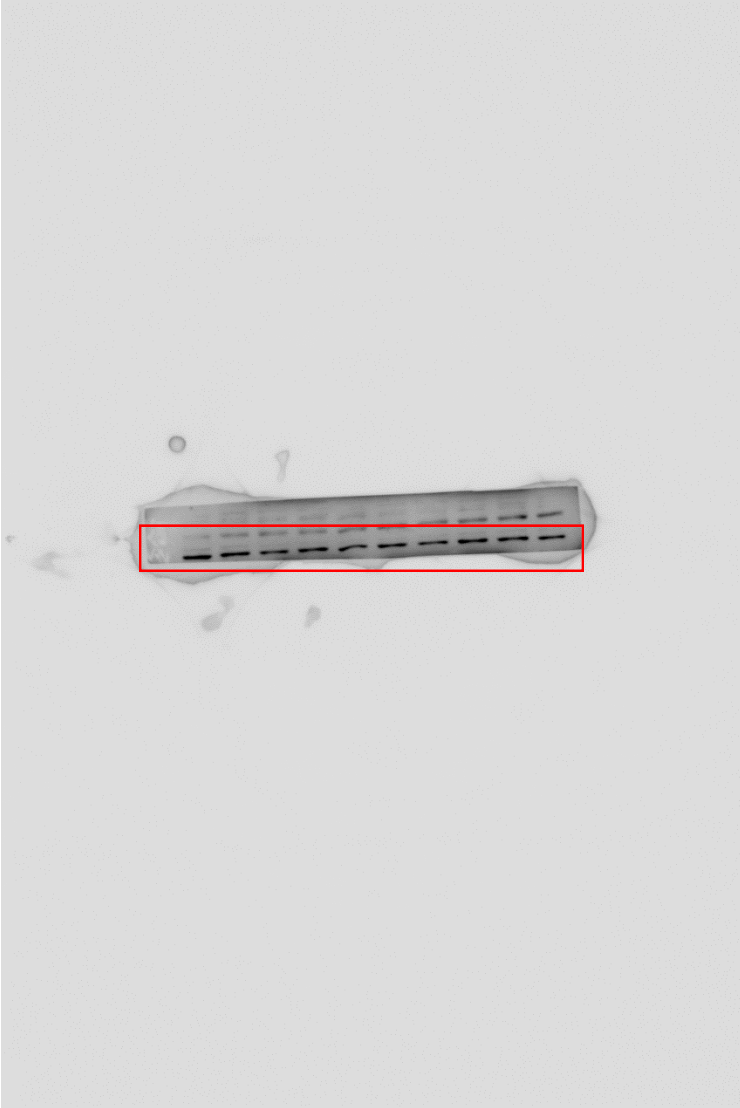

β-actin

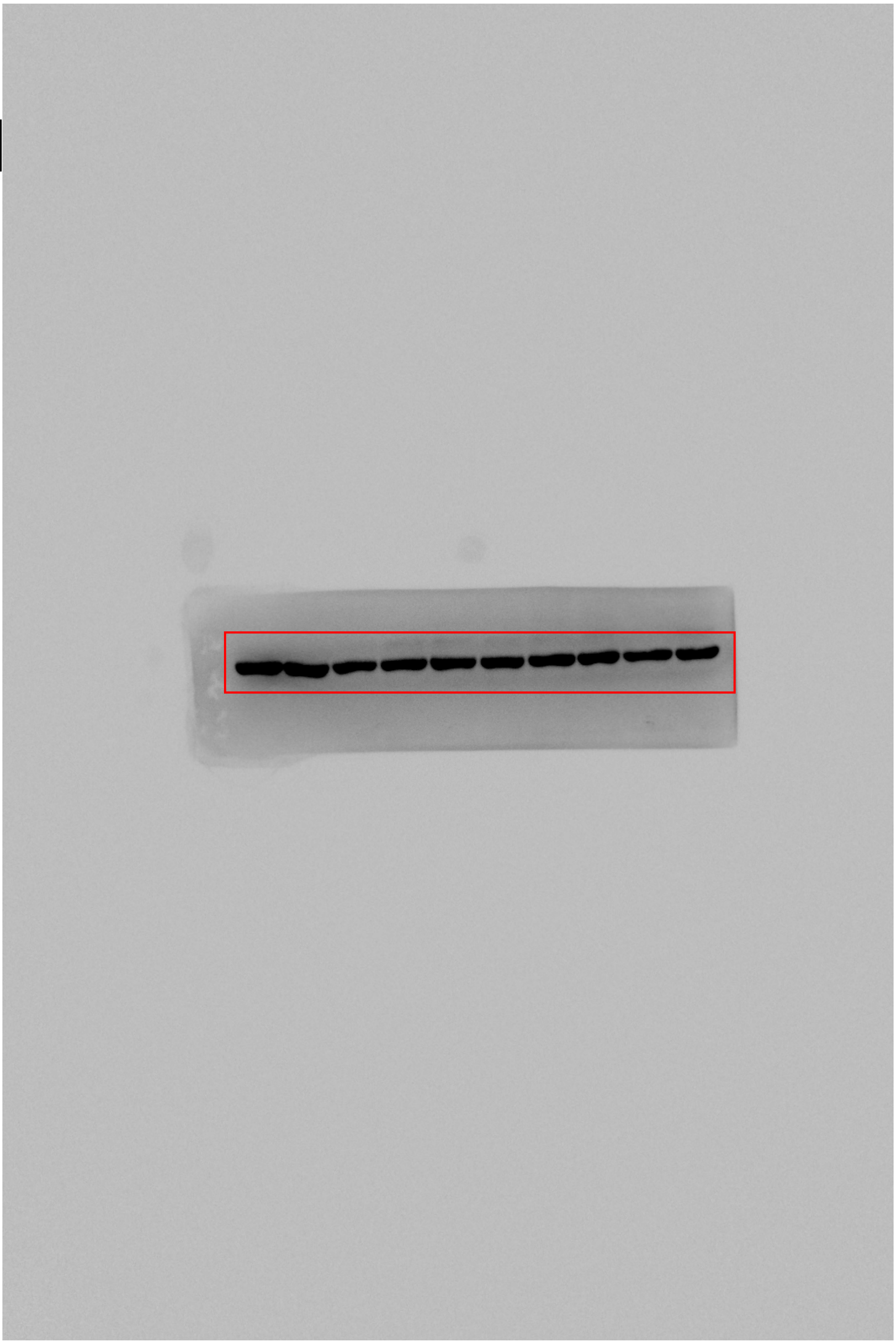

B

p-IRF3

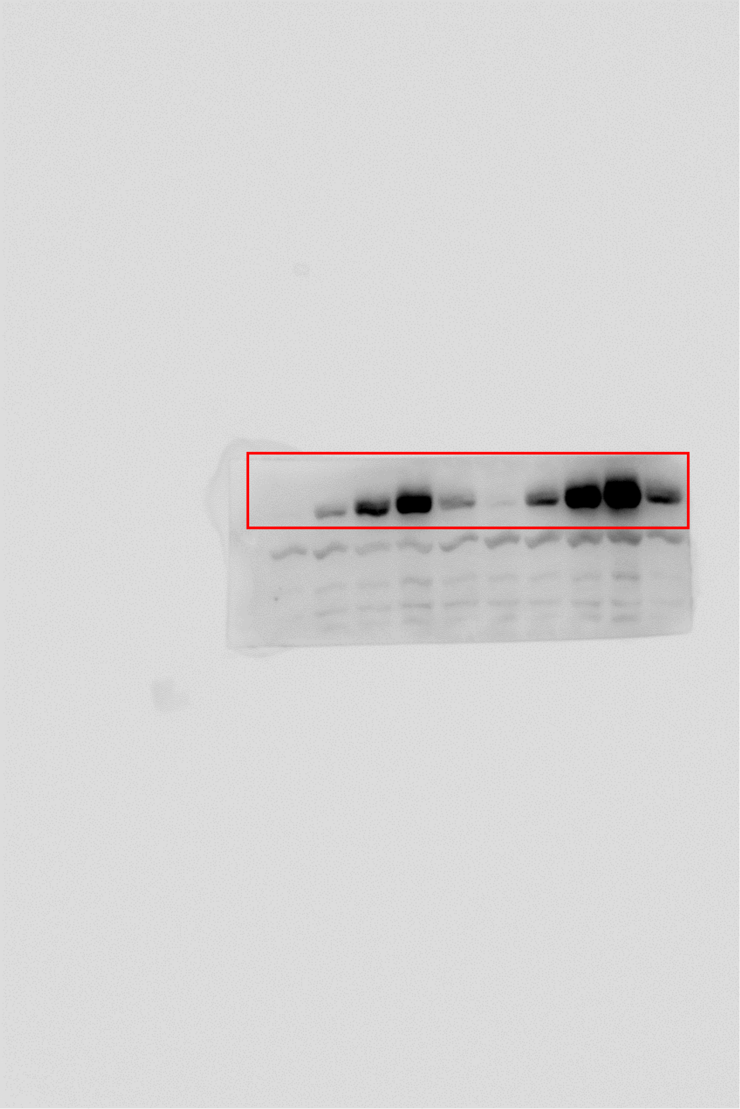

IRF3

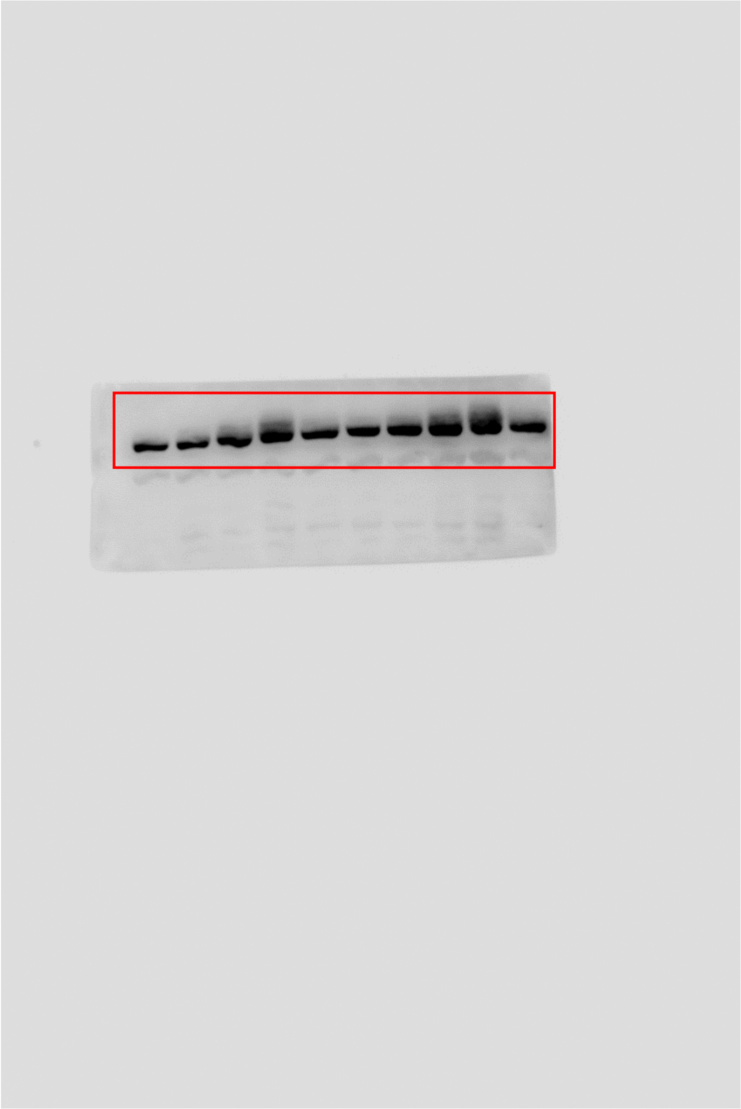

RIGI

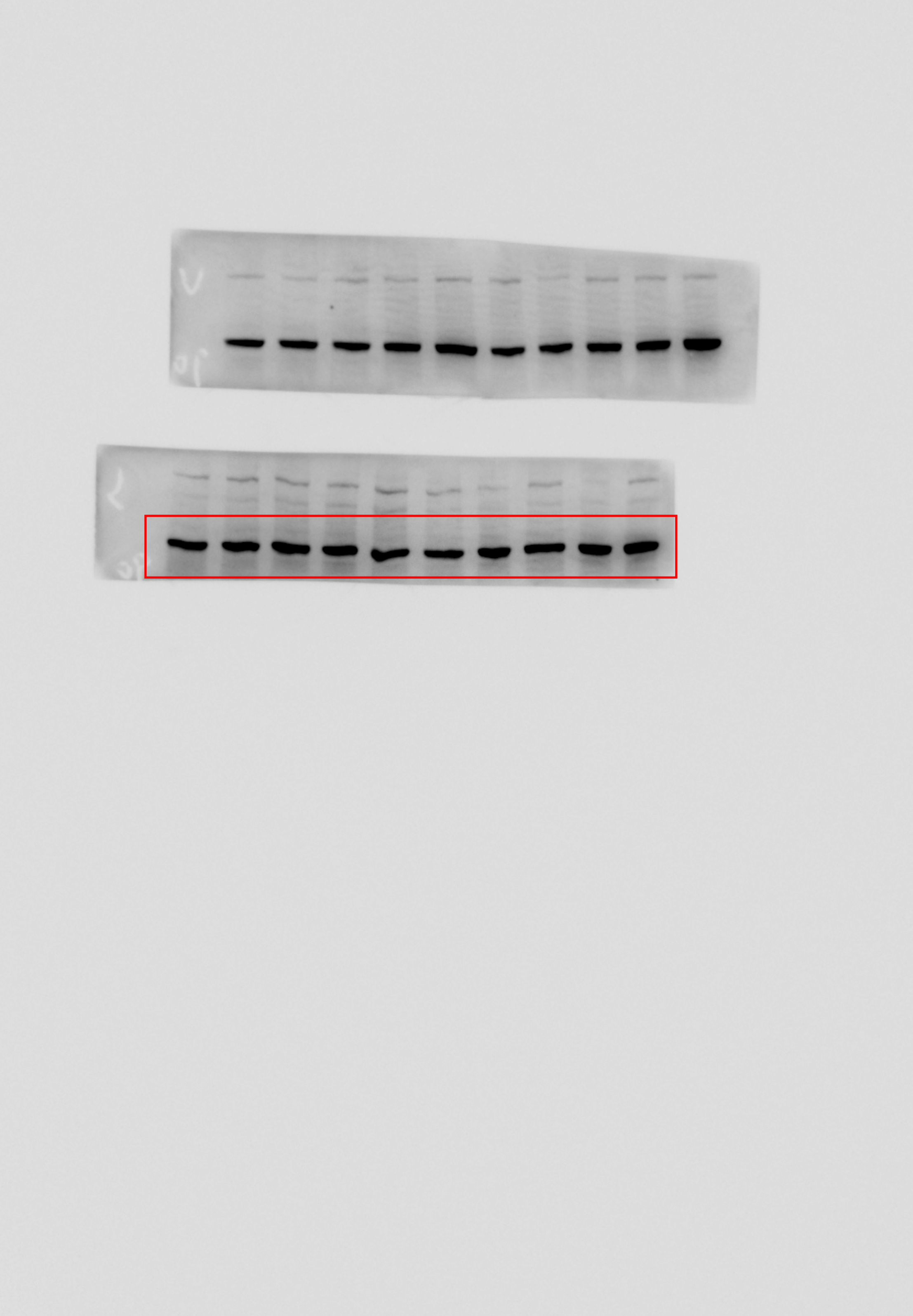

MAVS

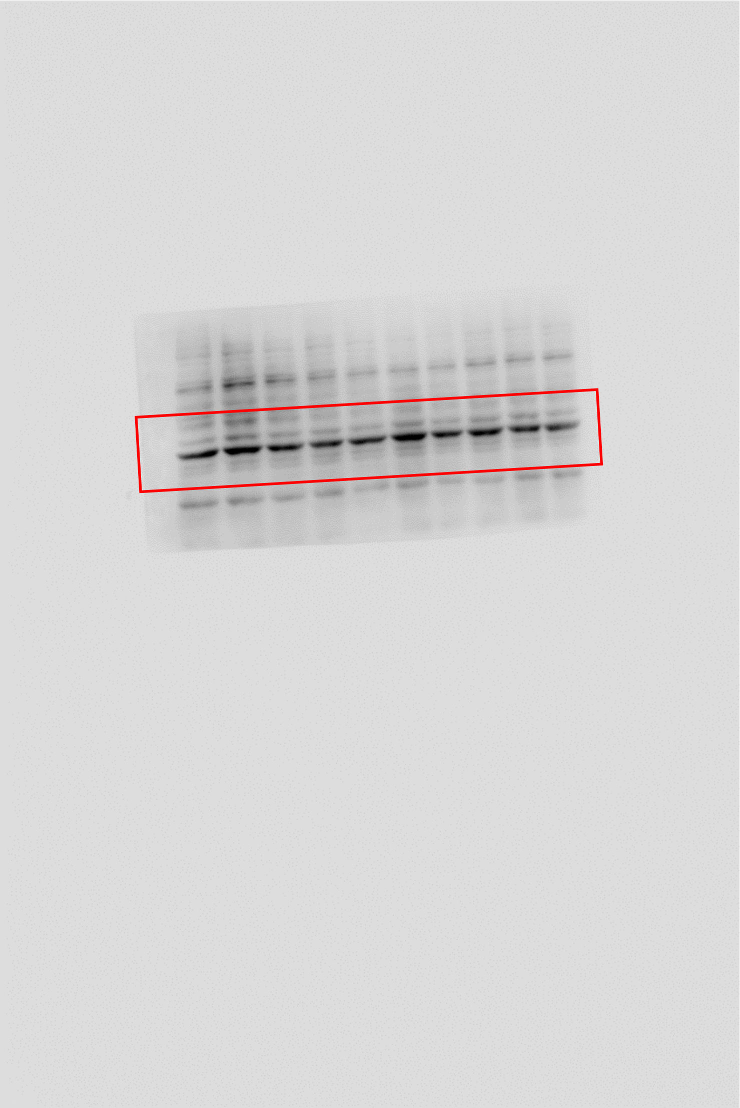

p-TBK1

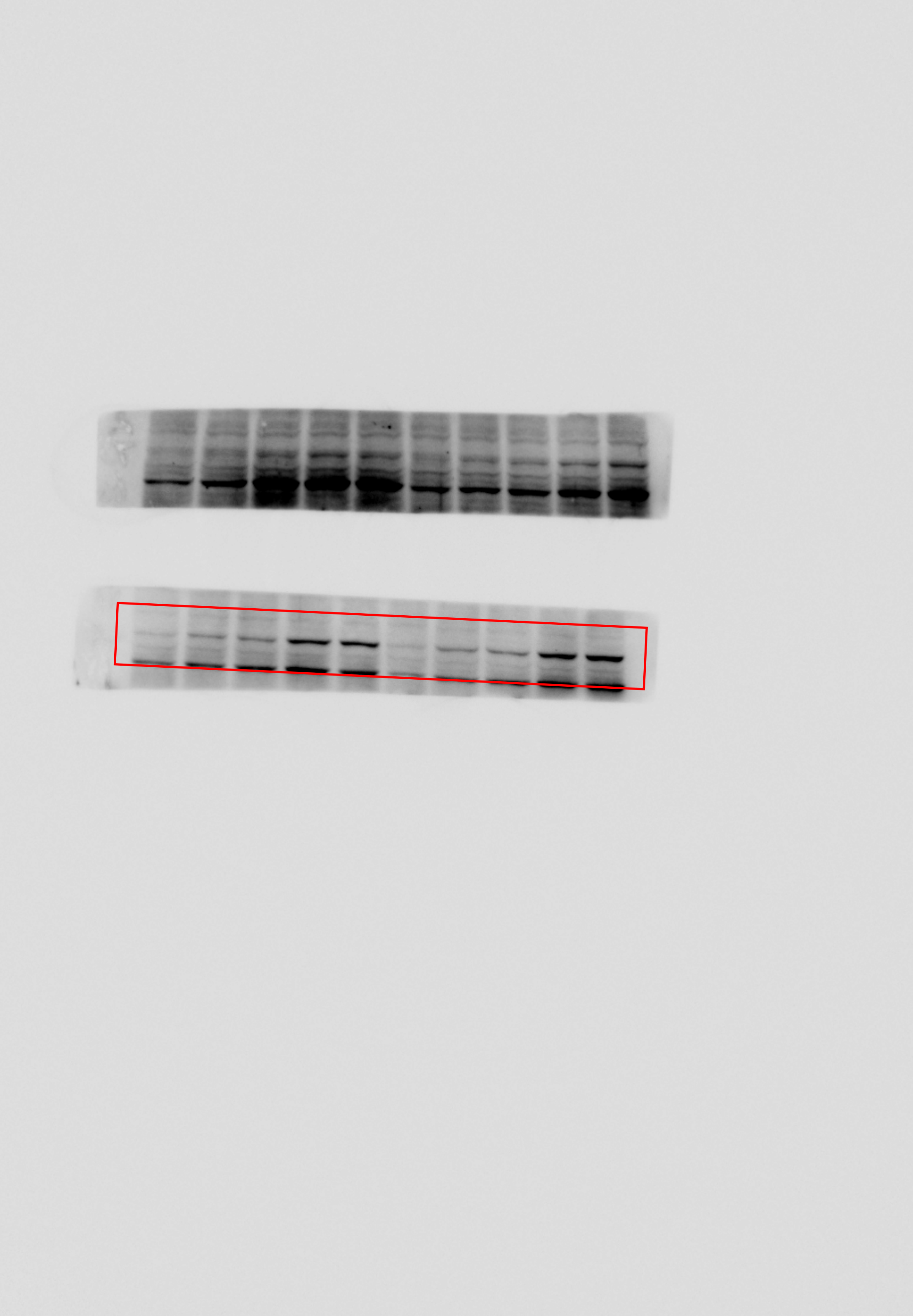

TBK1

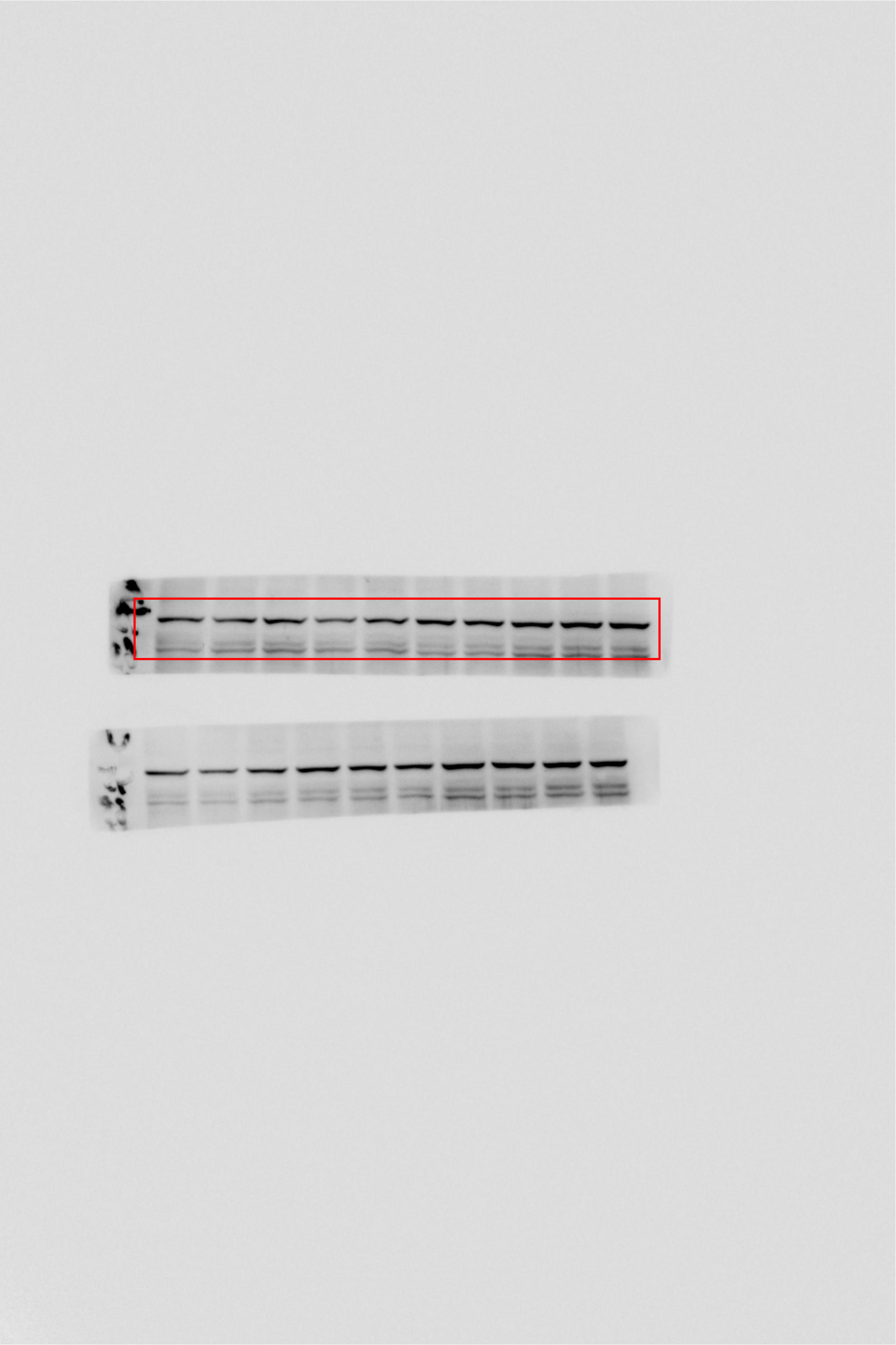

β-actin

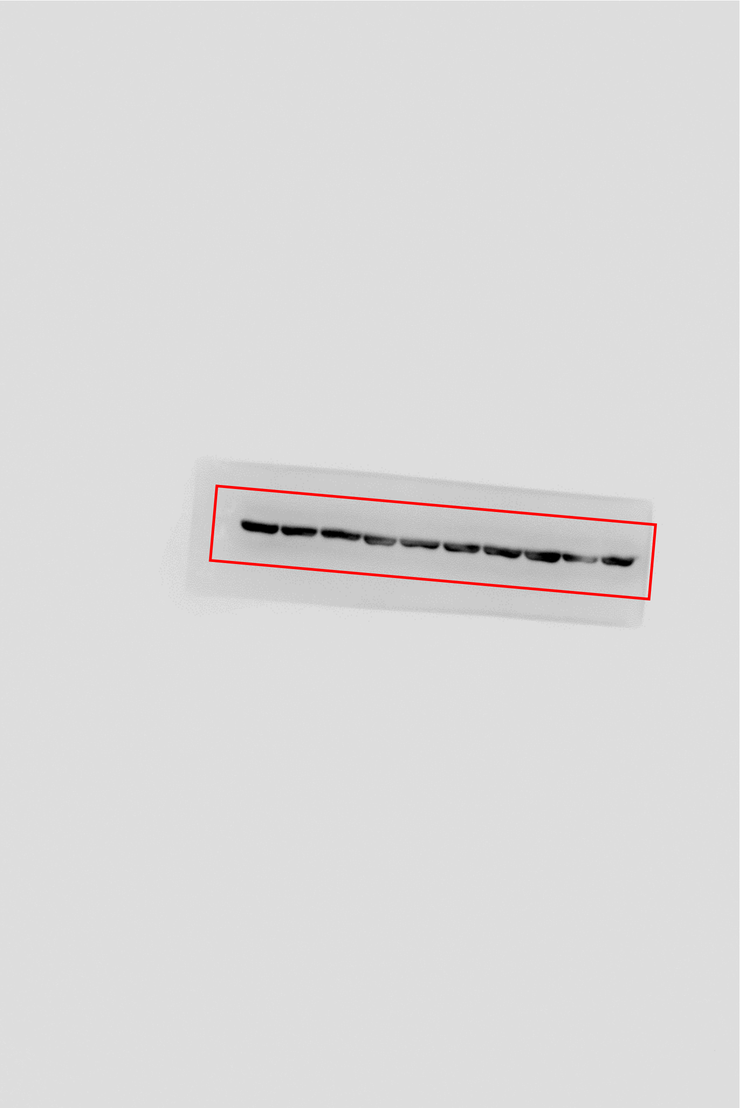

C

smyd2 $^{+/+}$ vsv 0h

IRF3

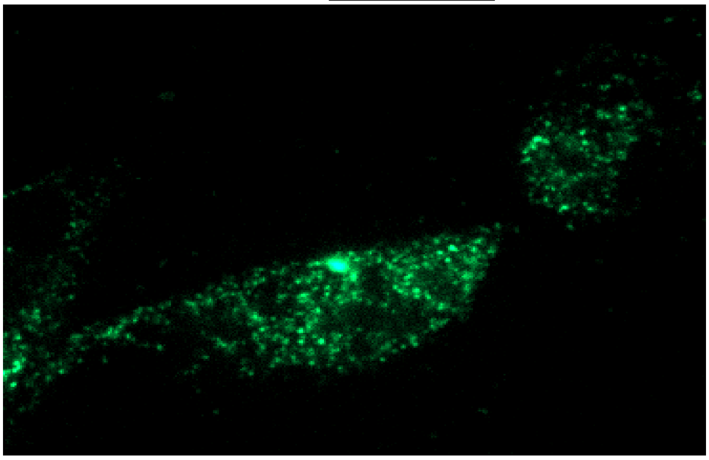

DAPI

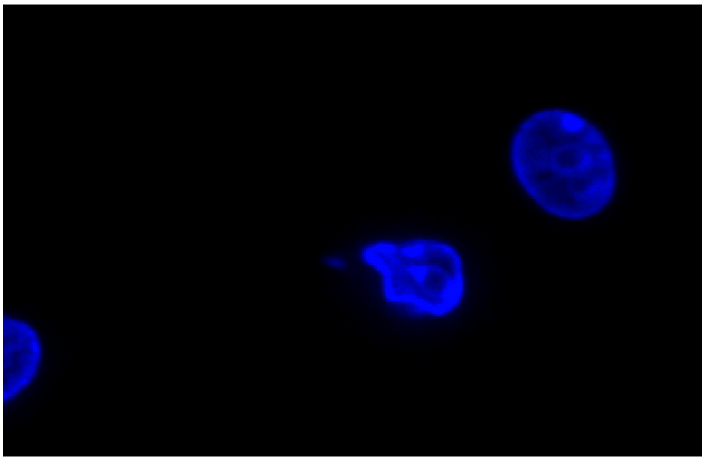

Merge

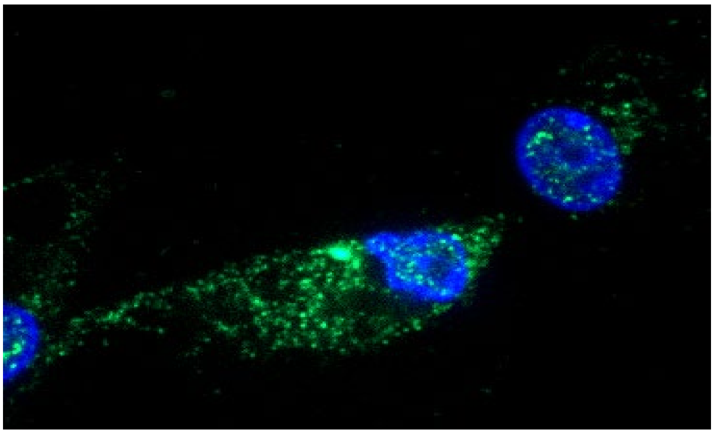

smyd2 $^{-/-}$ vsv 0h

IRF3

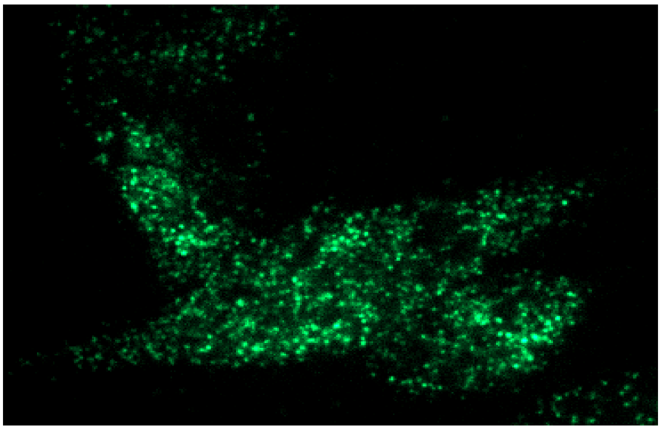

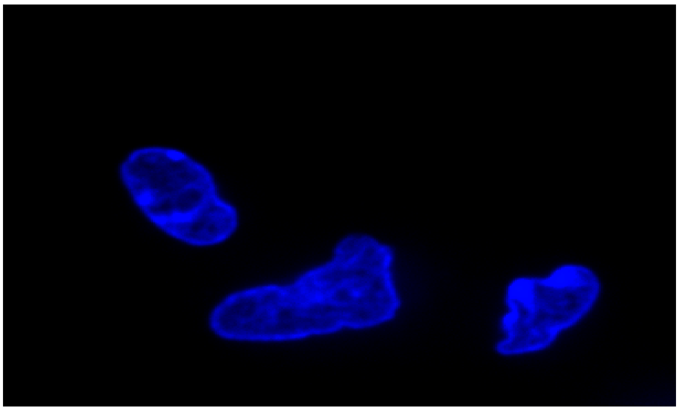

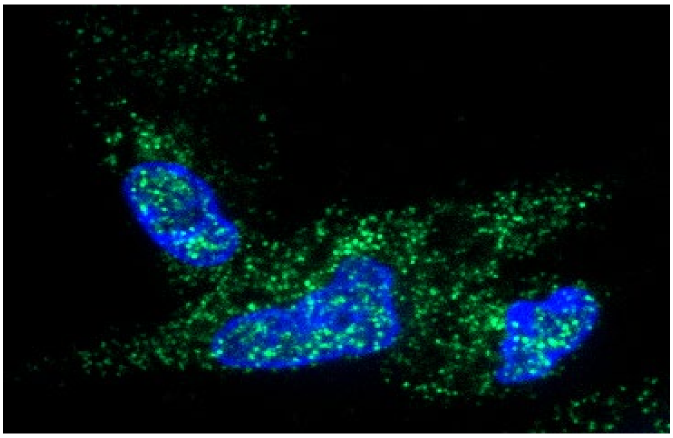

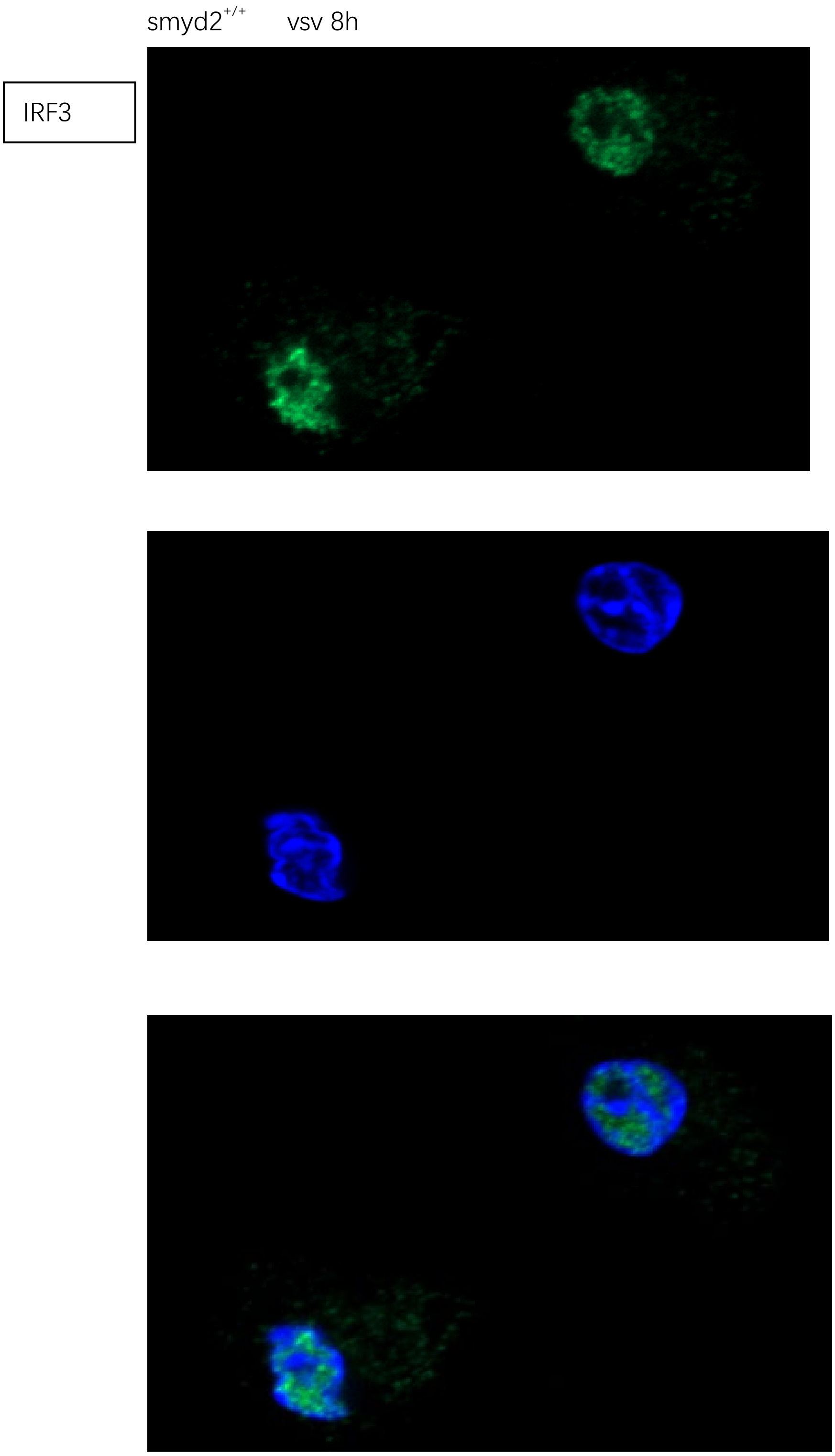

smyd2$^{+/+}$ vsv 8h
IRF3

smyd2 $^{-/-}$ vsv 8h

IRF3

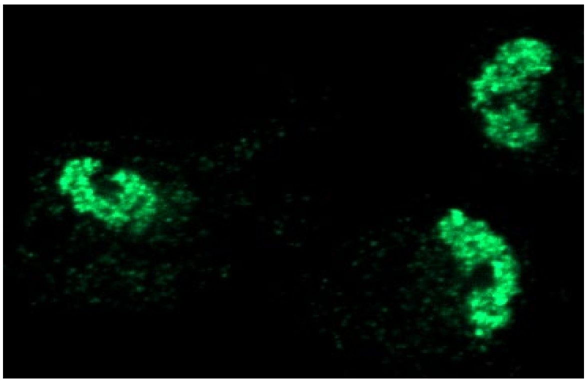

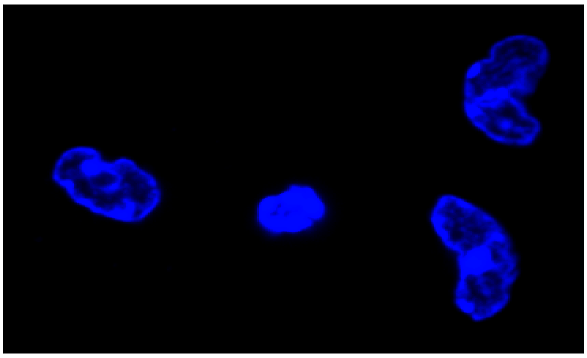

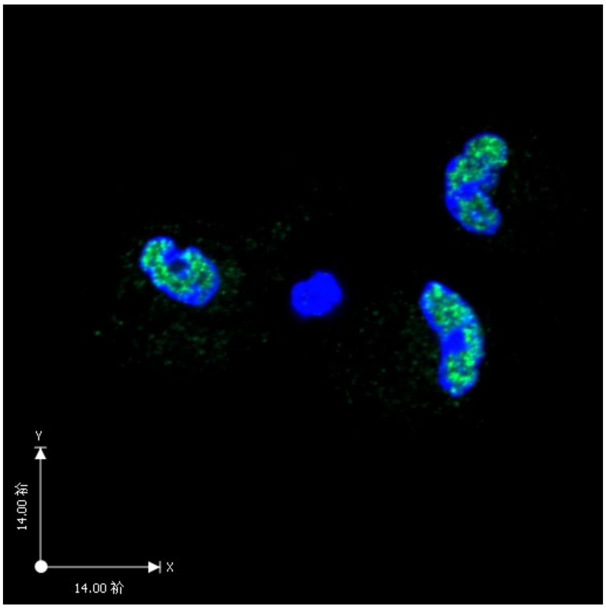

D

IRF3

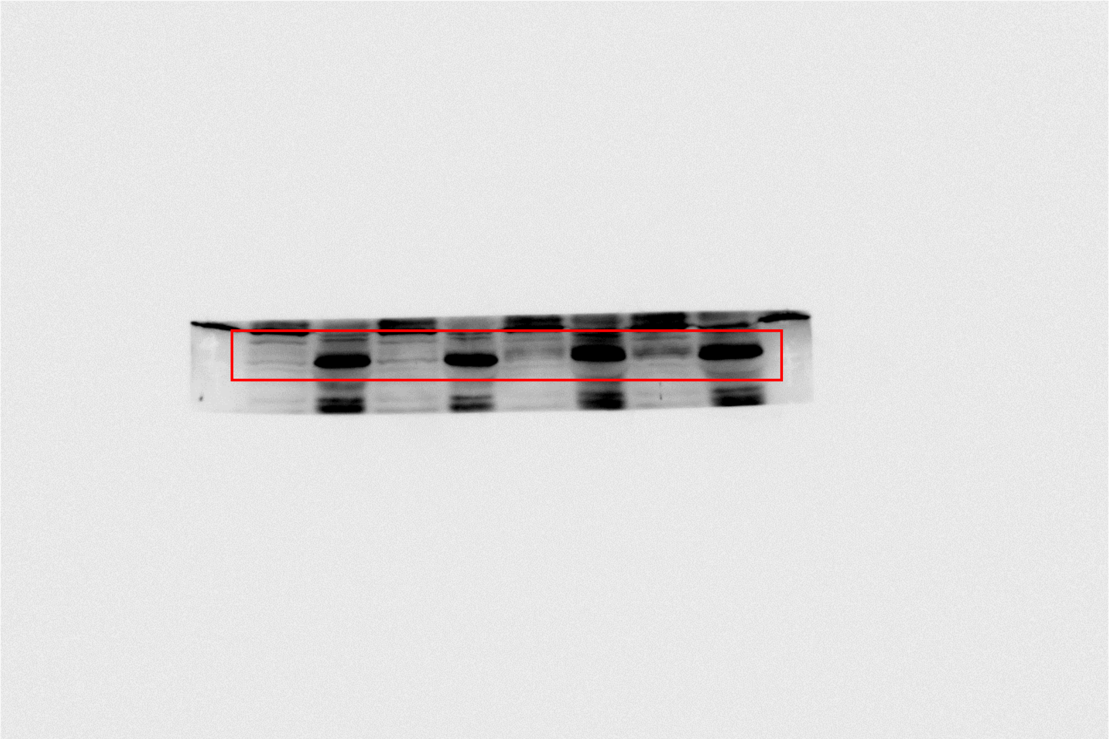

Lamin A/C

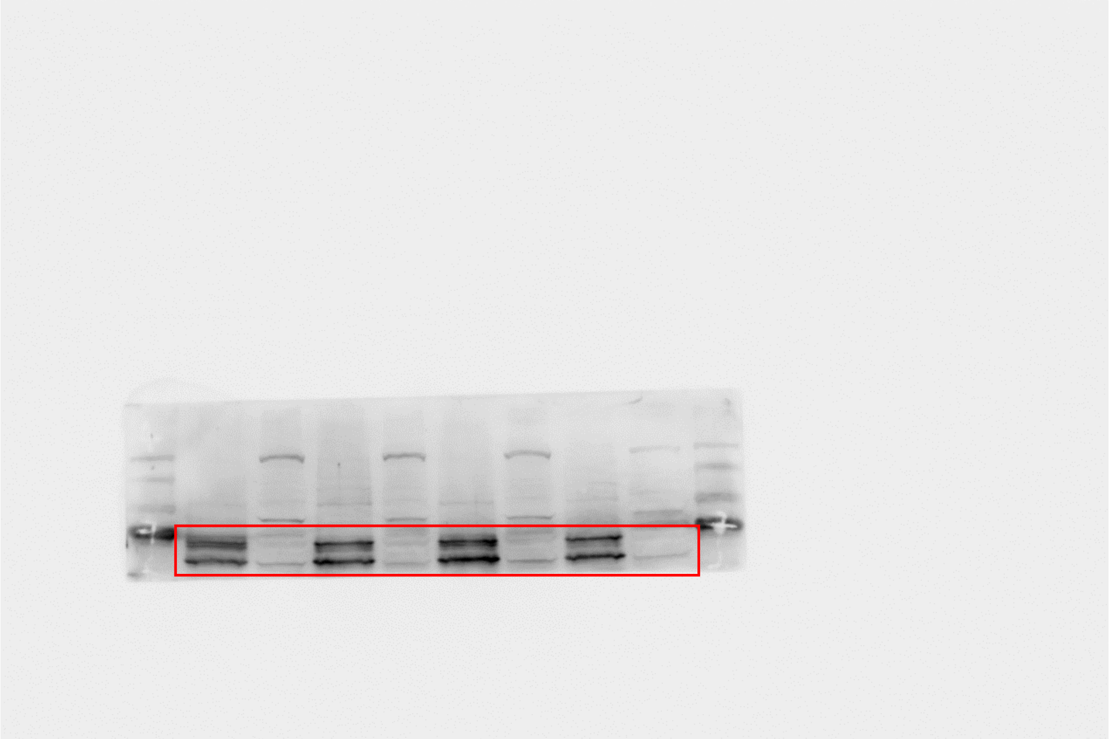

GAPDH

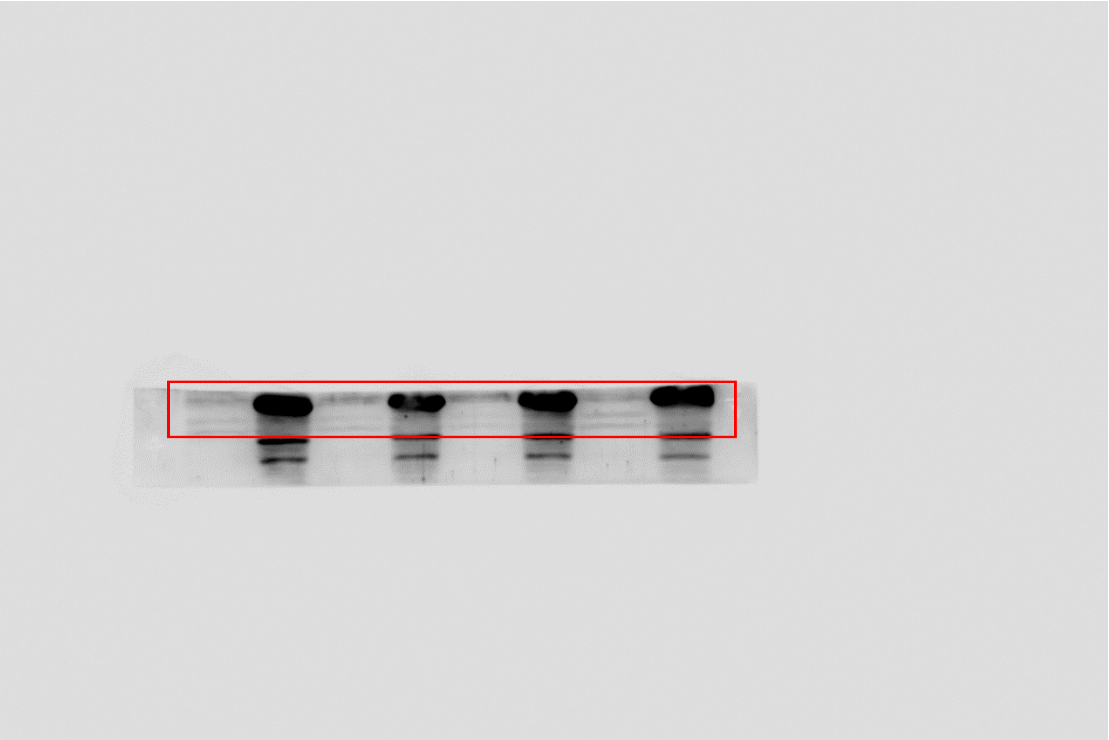

E

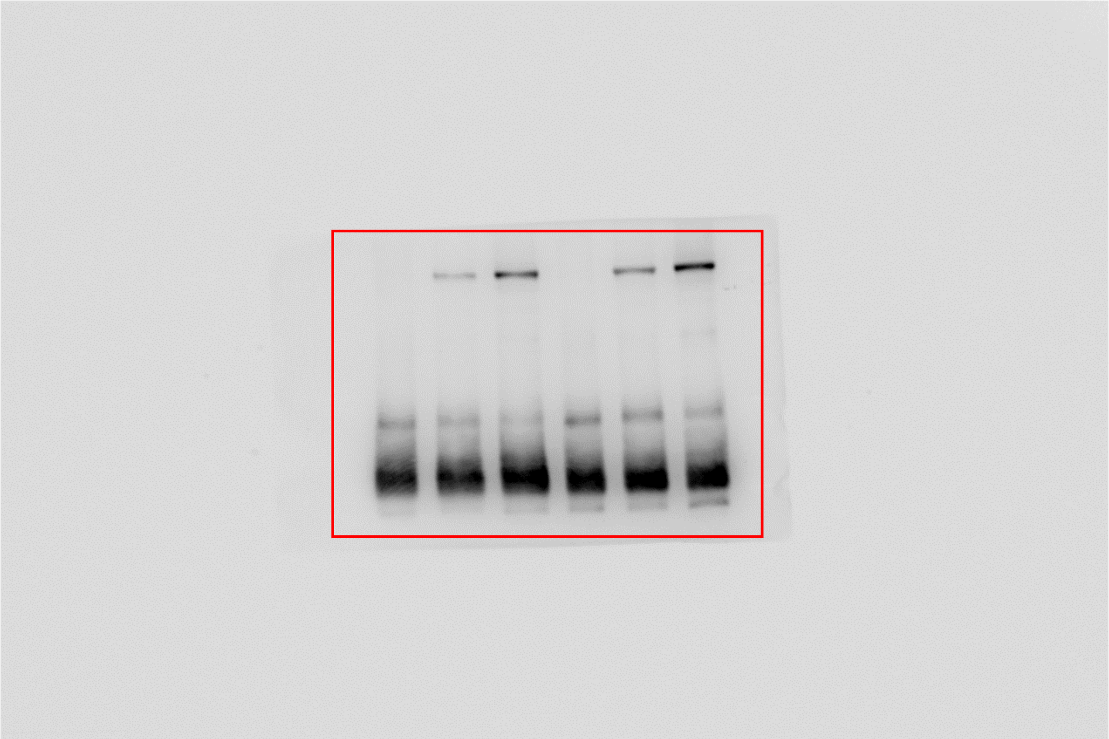

Fig. 6

D

Flag-SMYD2

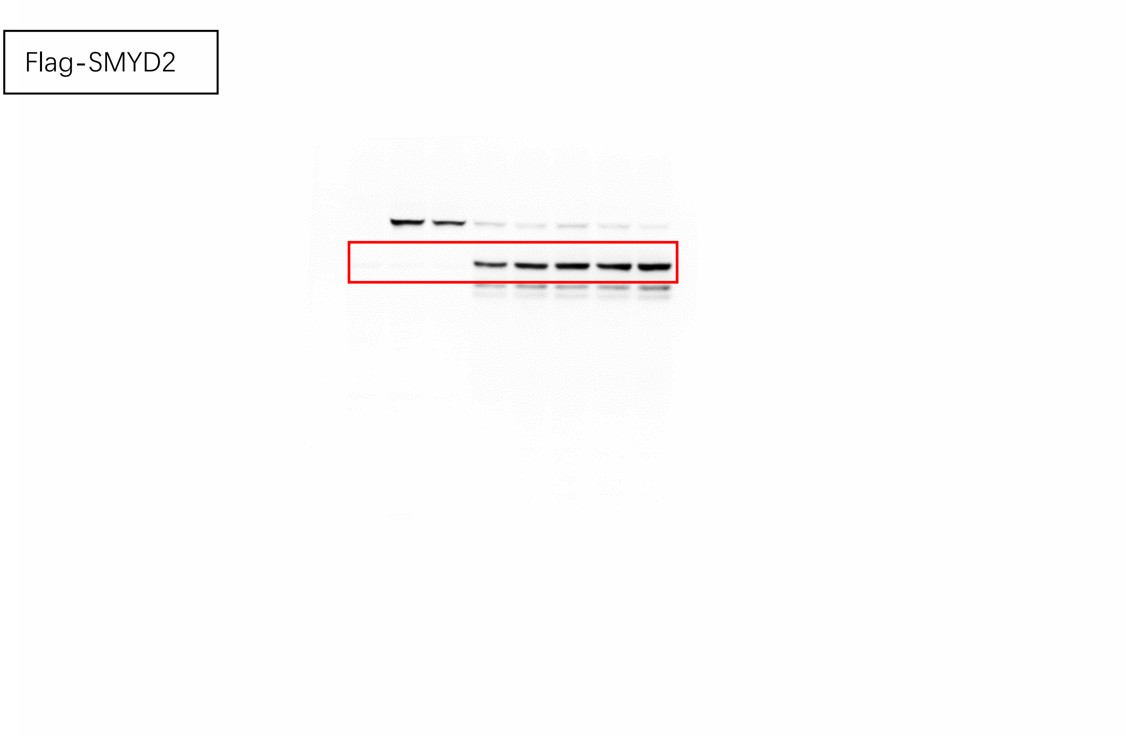

Flag-TBK1

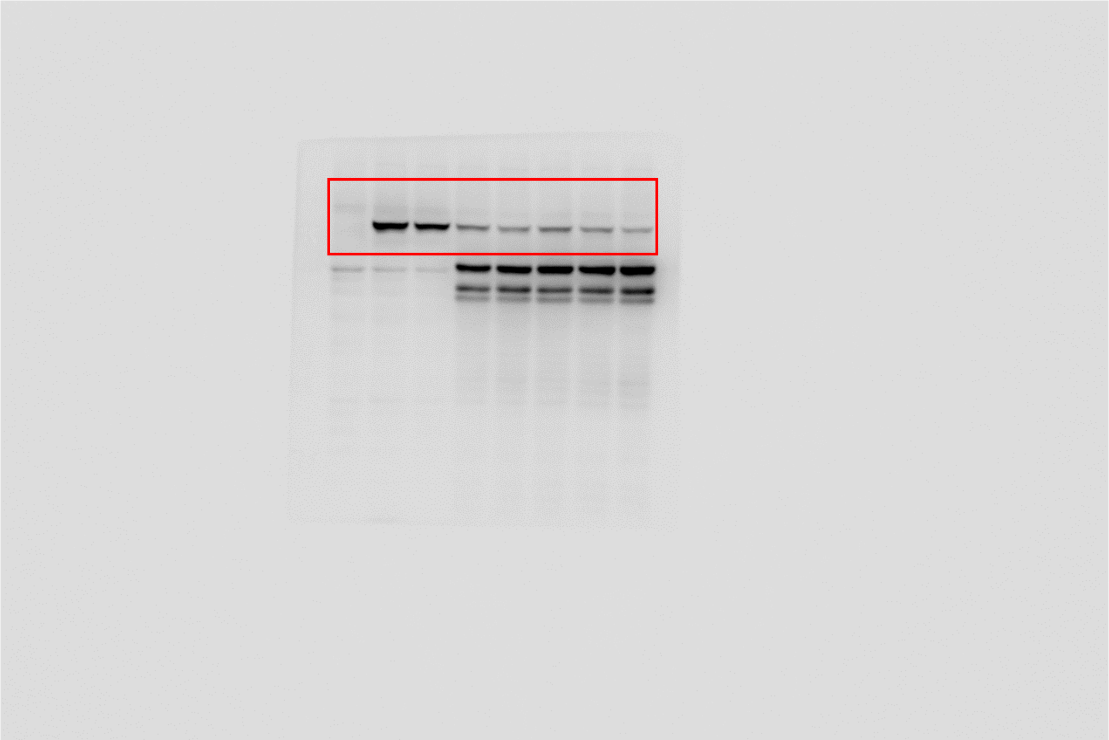

β-actin

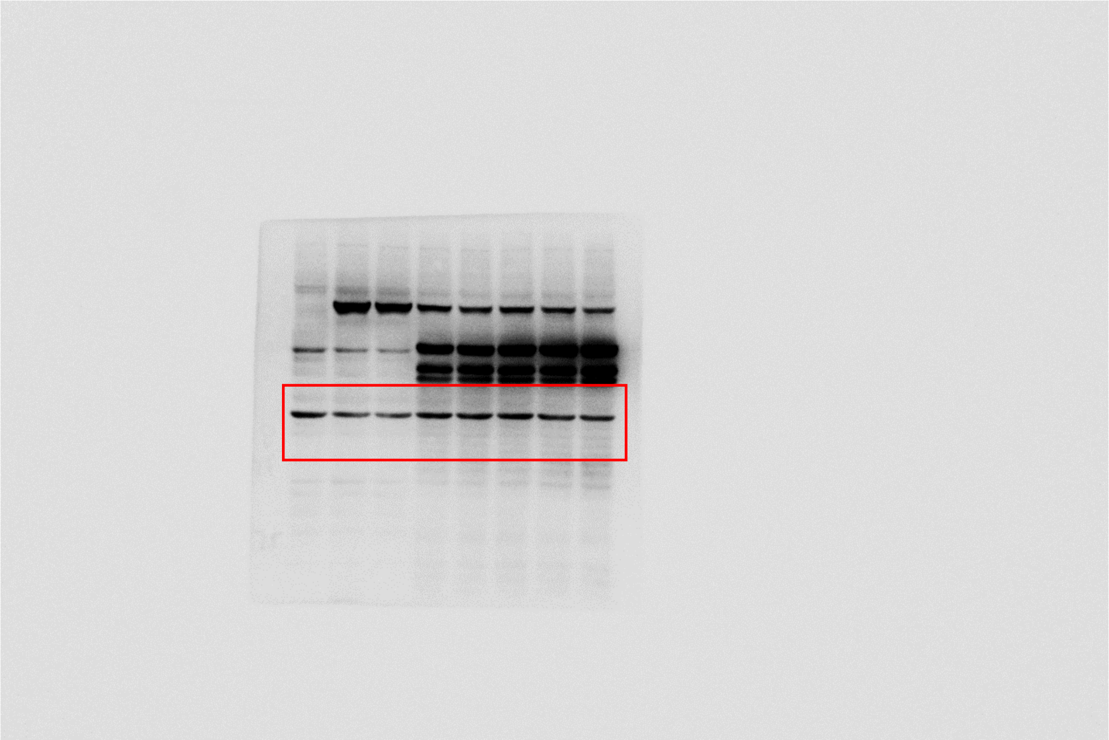

E

flag-TBK1

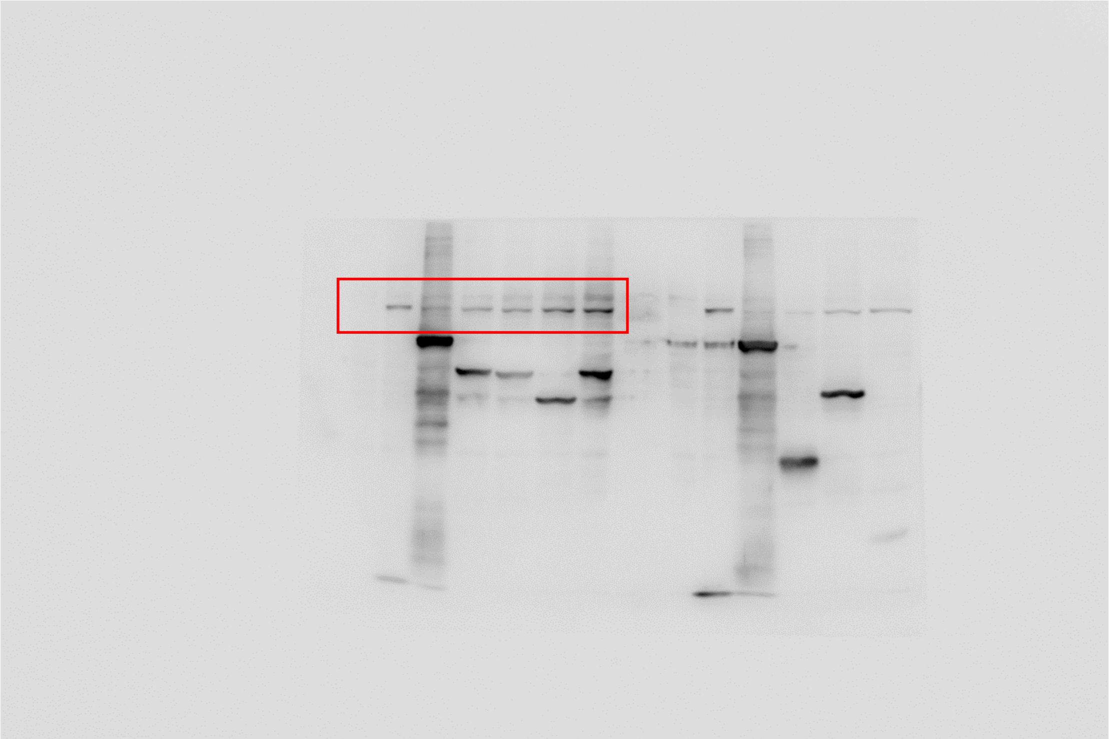

Flag

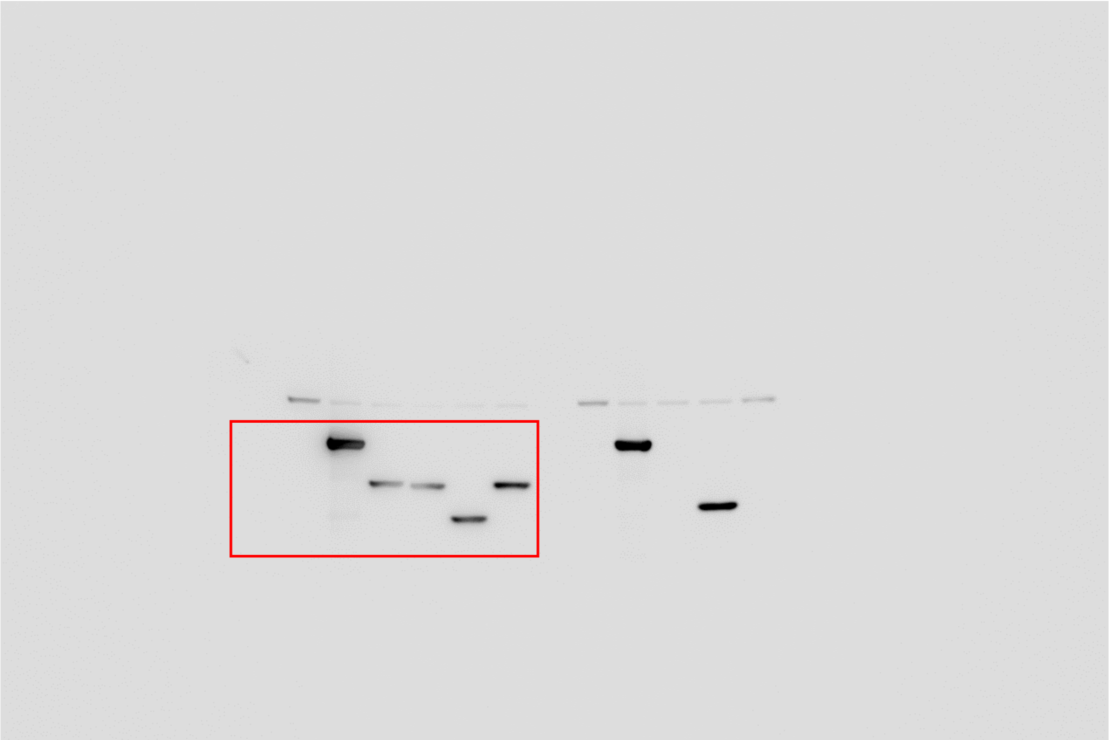

β-actin

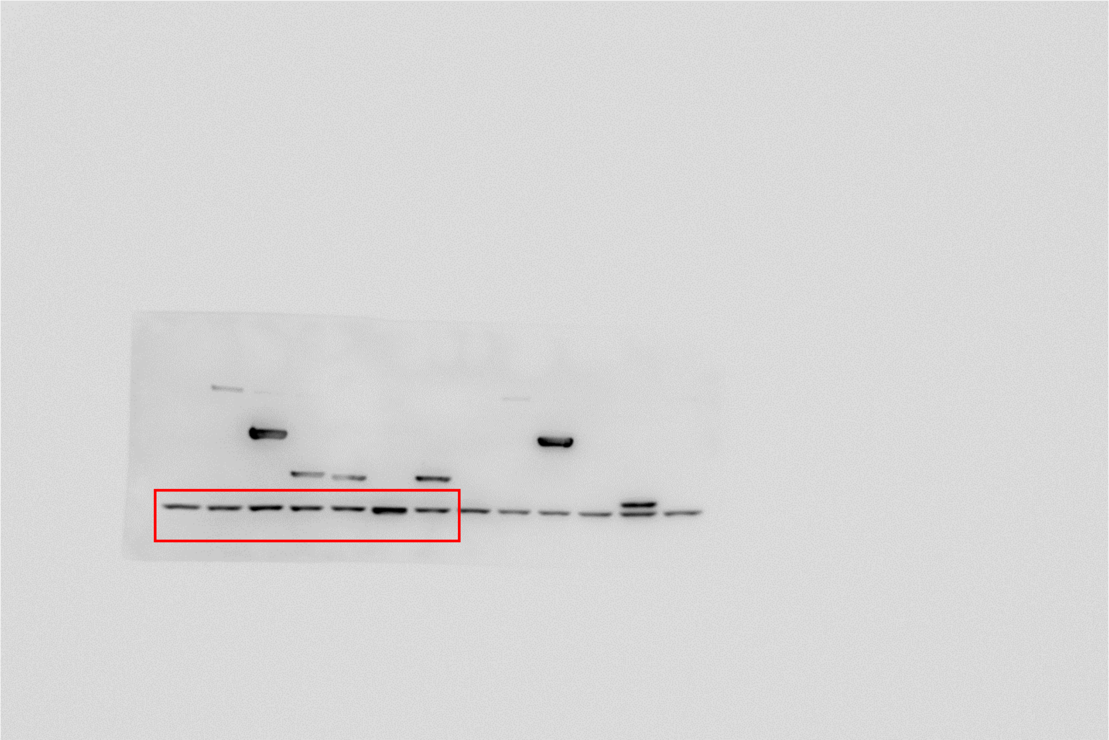

F

p-TBK1

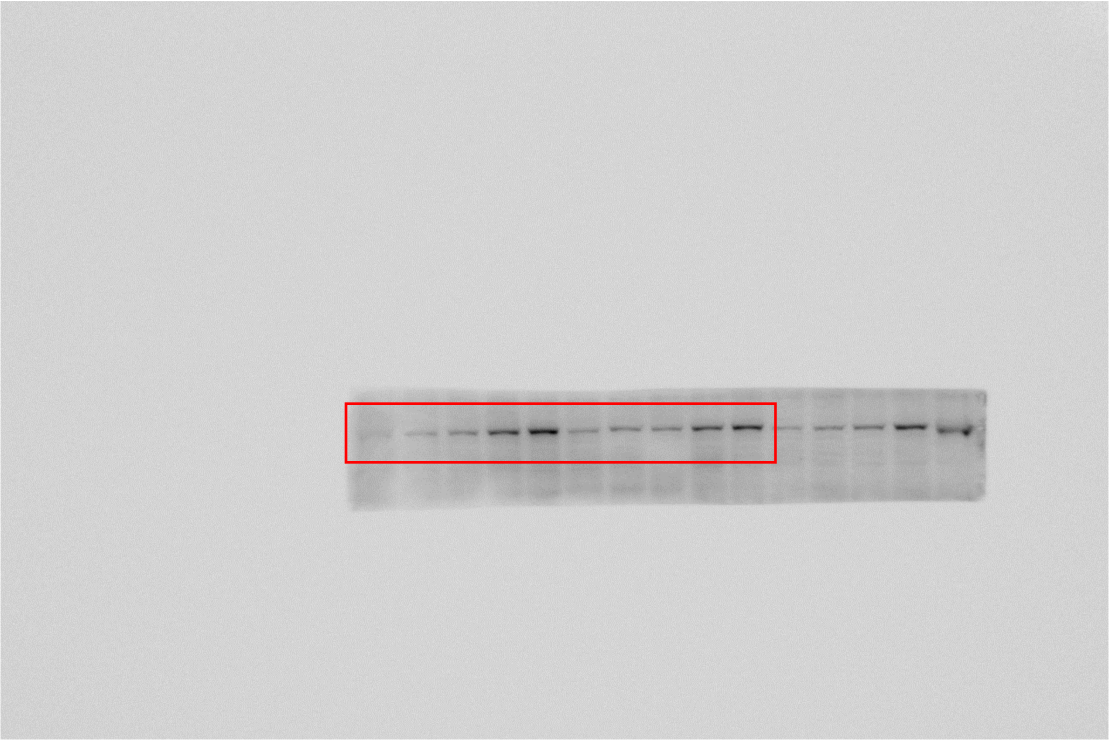

p-P65

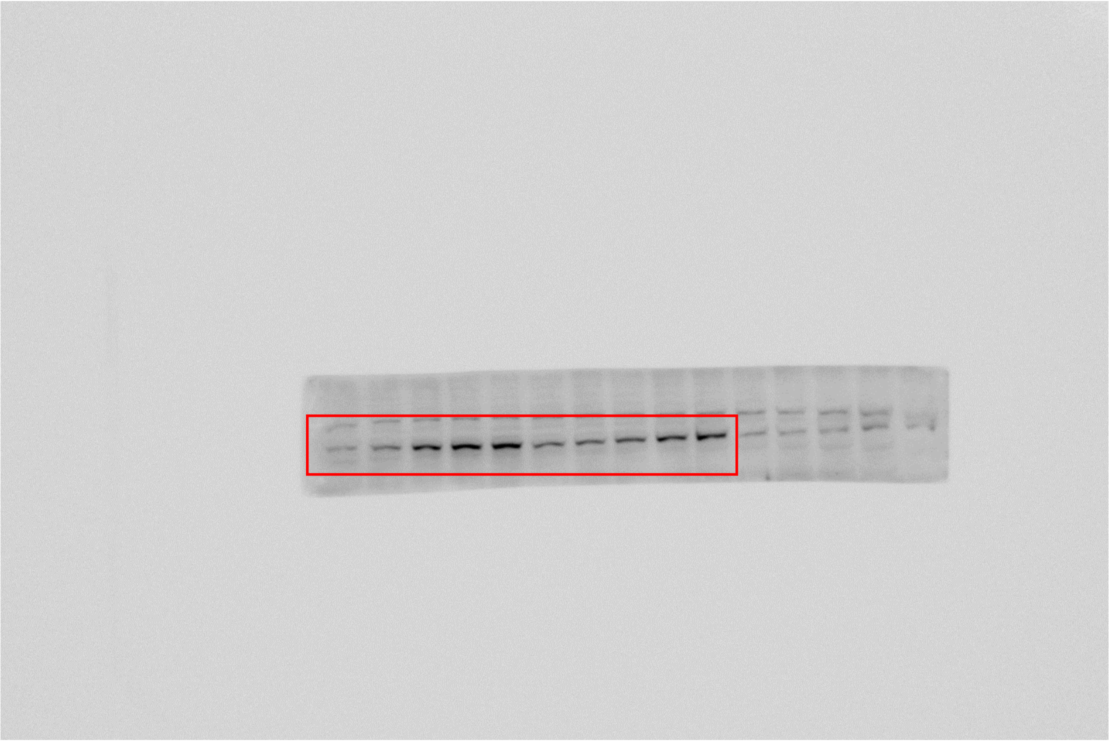

p-IRF3

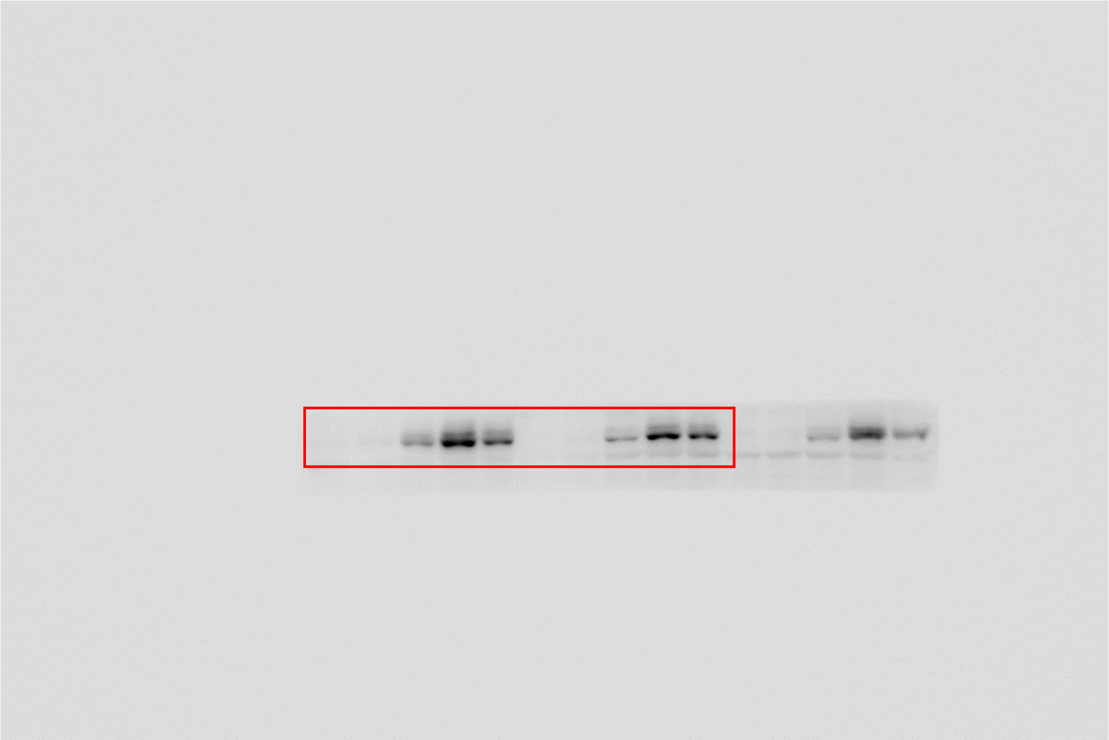

IRF3

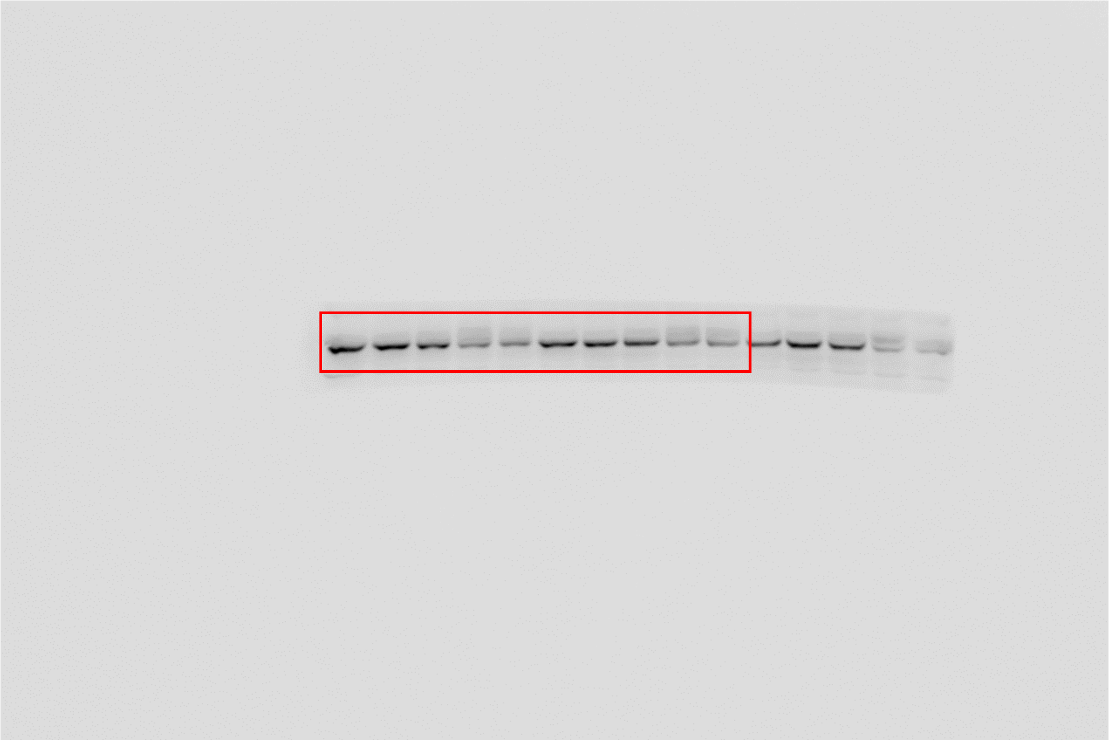

β-actin

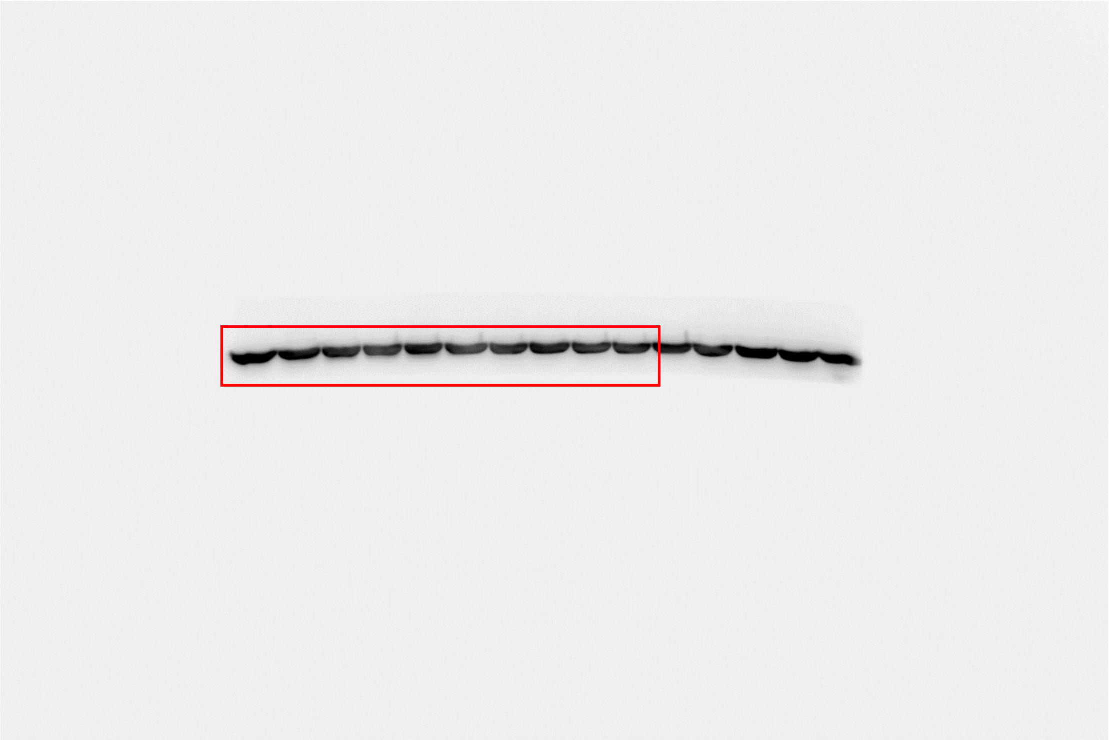

G

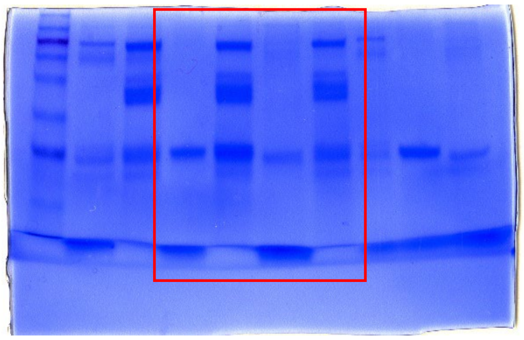

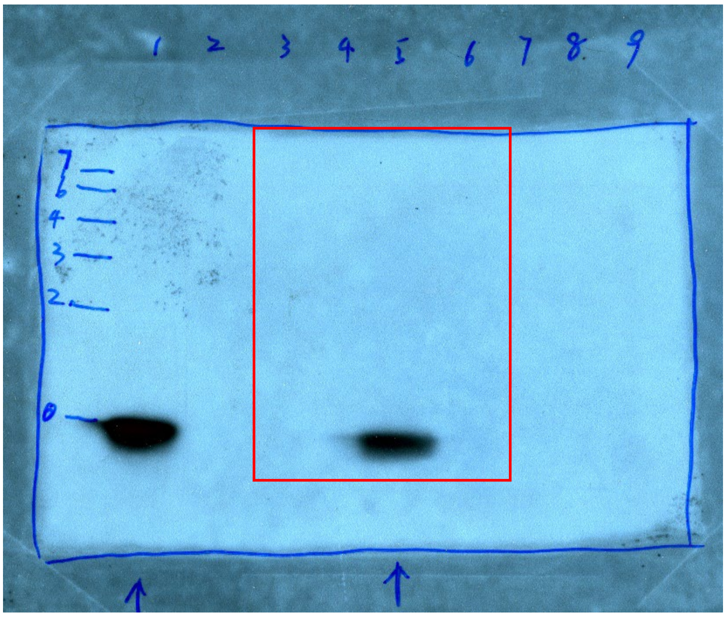

I

Flag

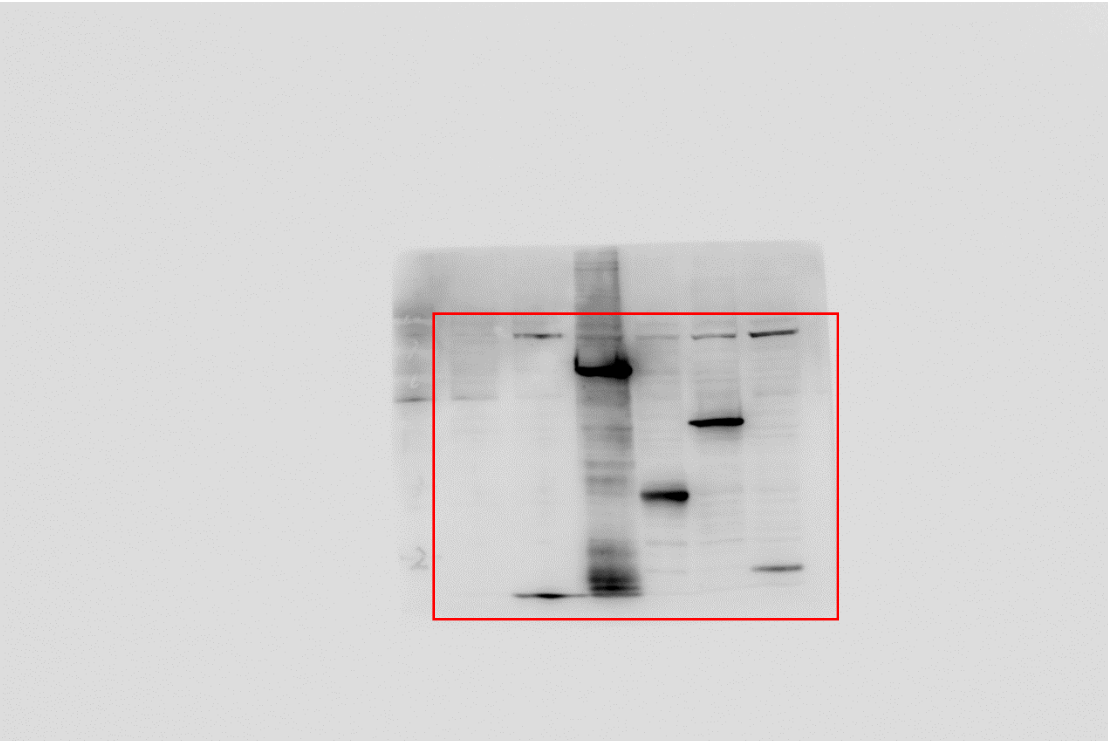

β-actin

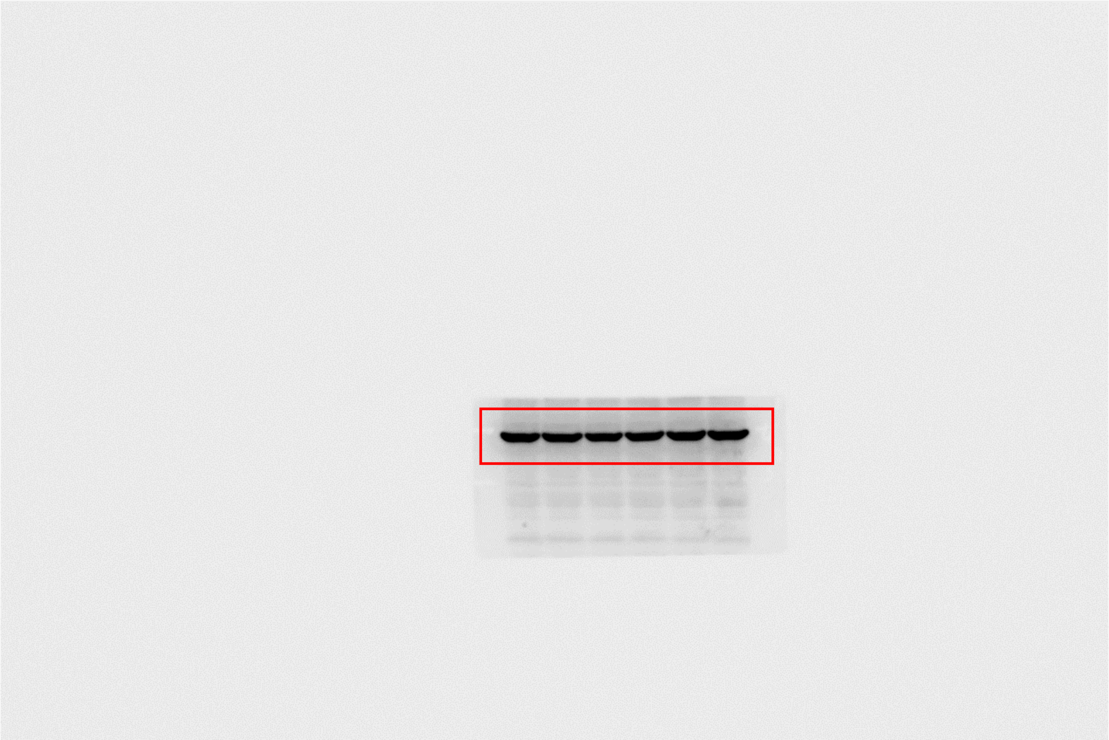

Fig. 7

A

Flag-Smyd2
IP Flag
Anti-Flag

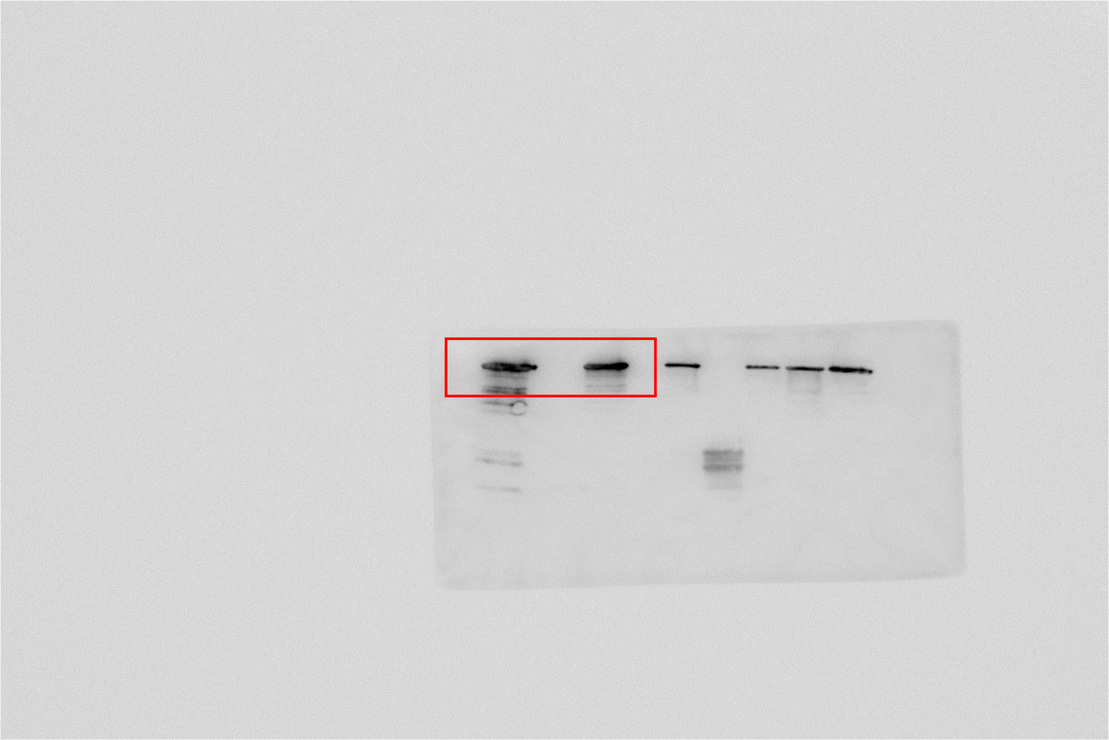

Flag-Smyd2
IP Flag
Anti-Myc

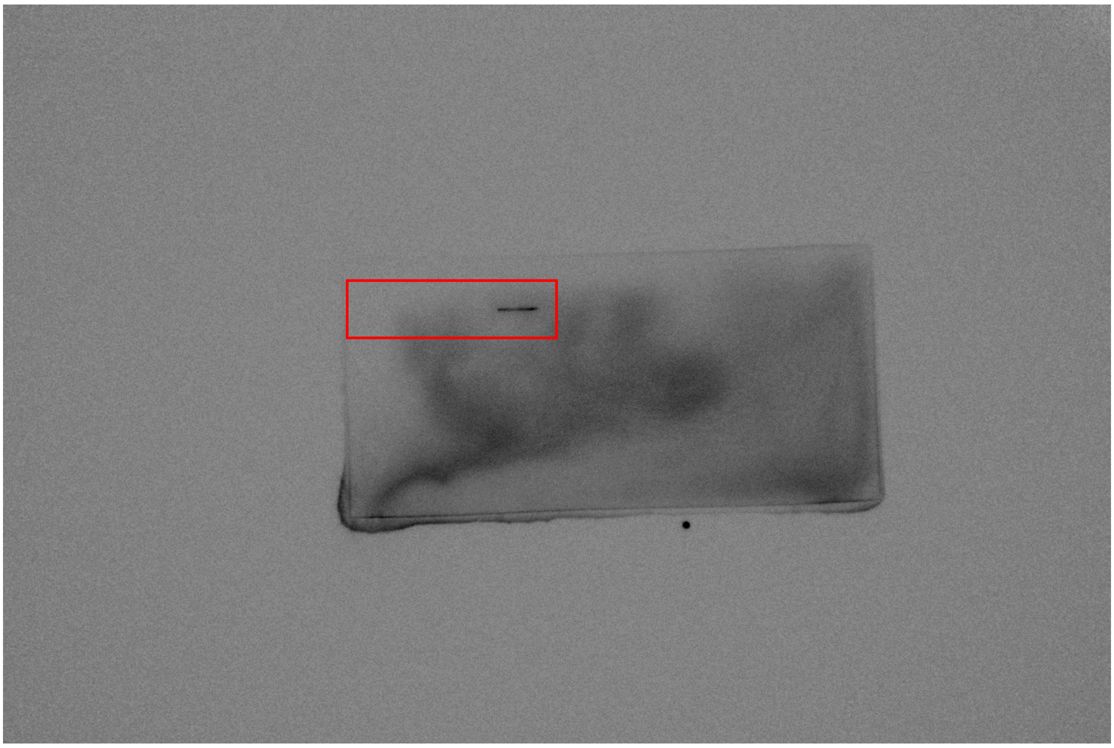

Flag-IRF3
IP Flag
Anti-Myc

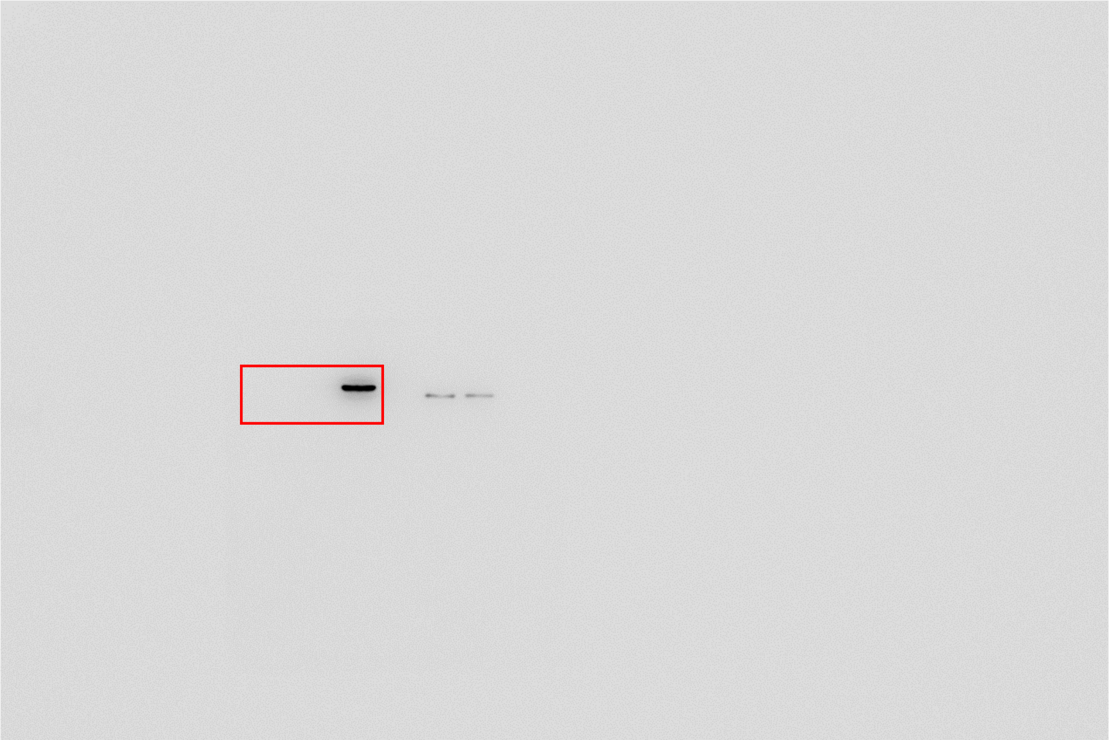

Flag-IRF3
IP Flag
Anti-Flag

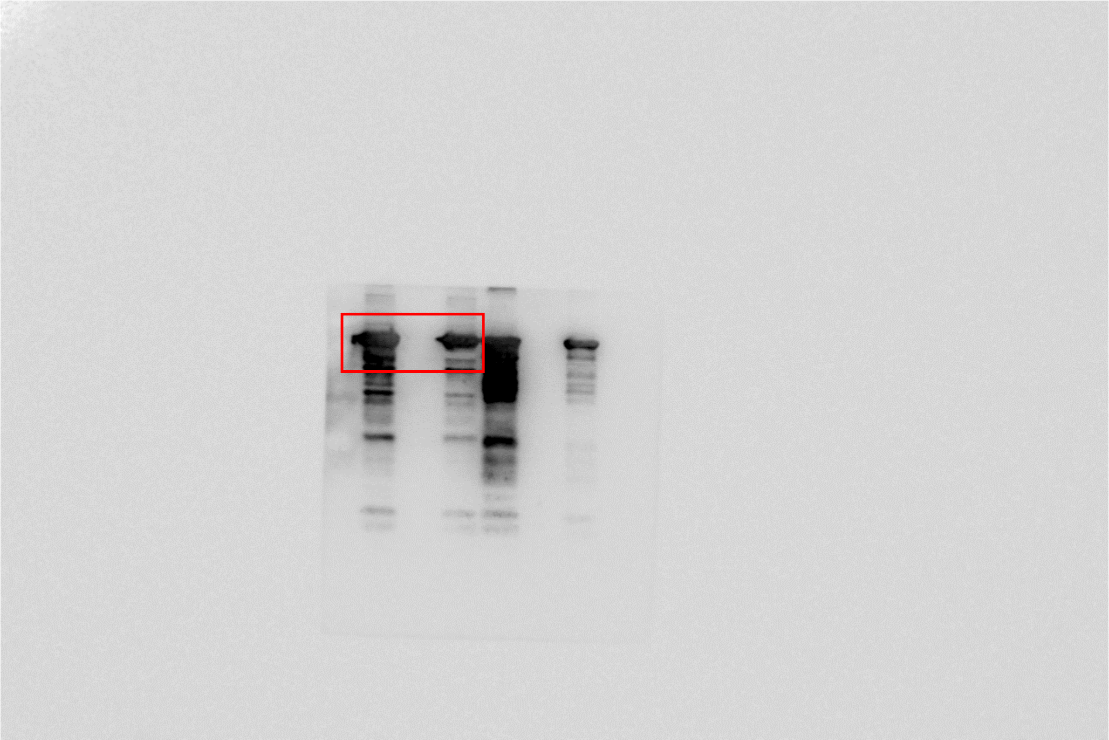

Flag-IRF3
input
Anti-Myc

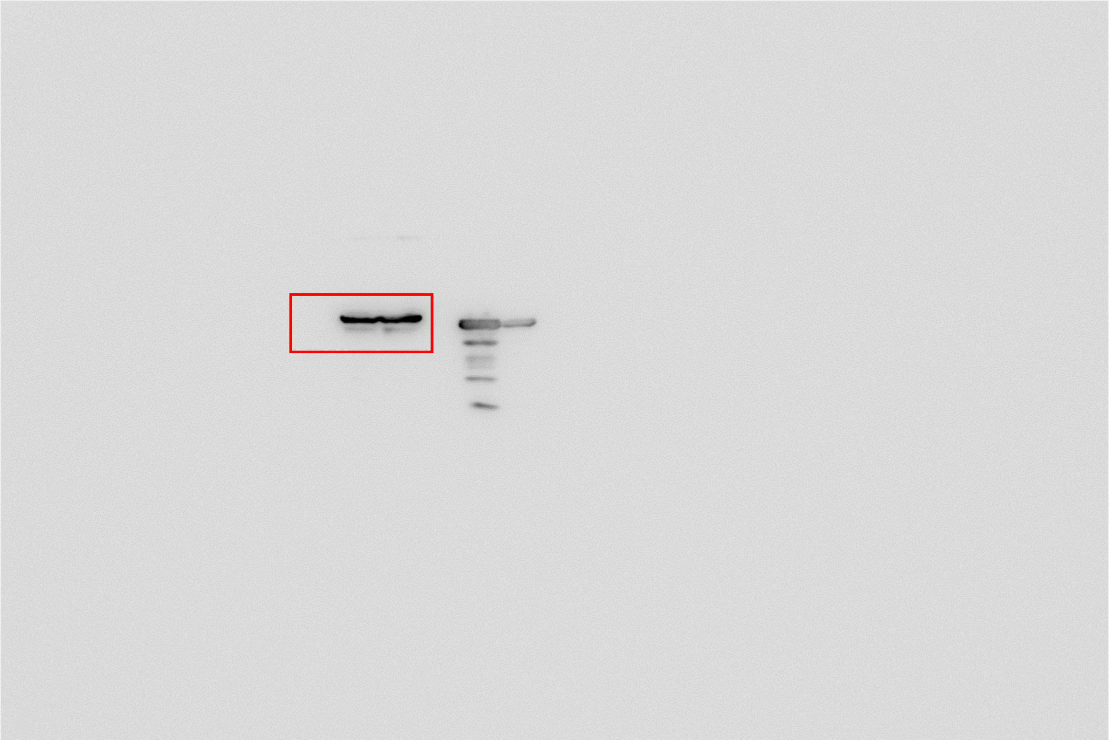

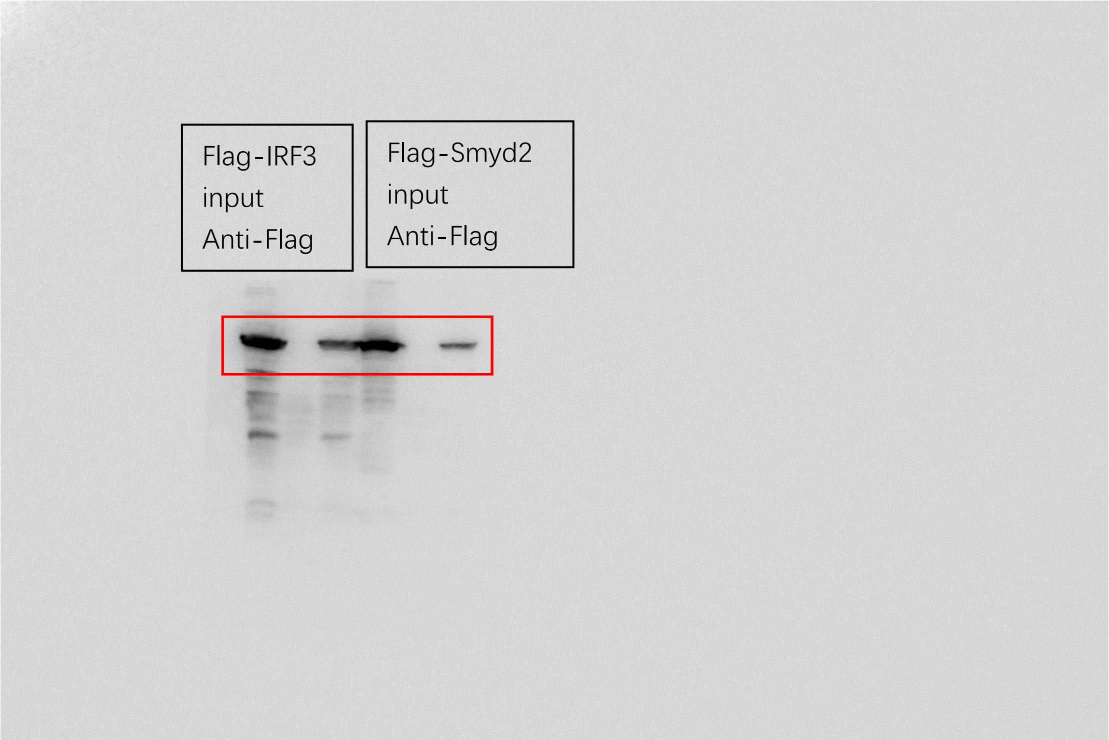

Flag-Smyd2
input
Anti-Myc

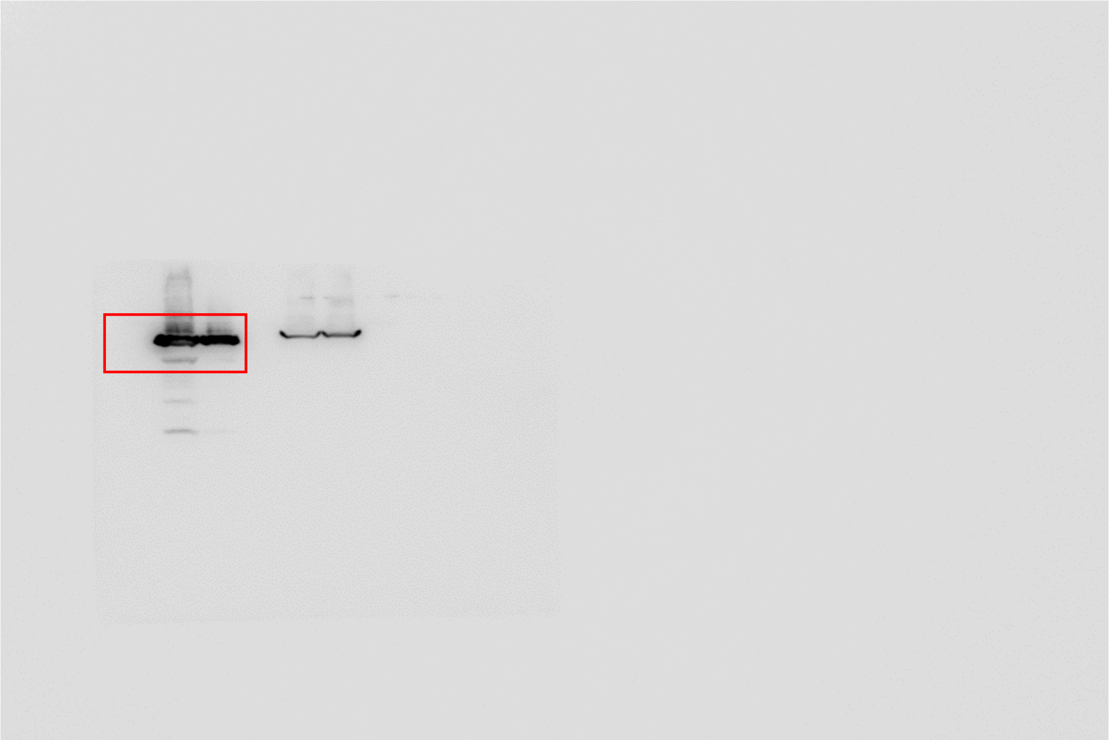

B

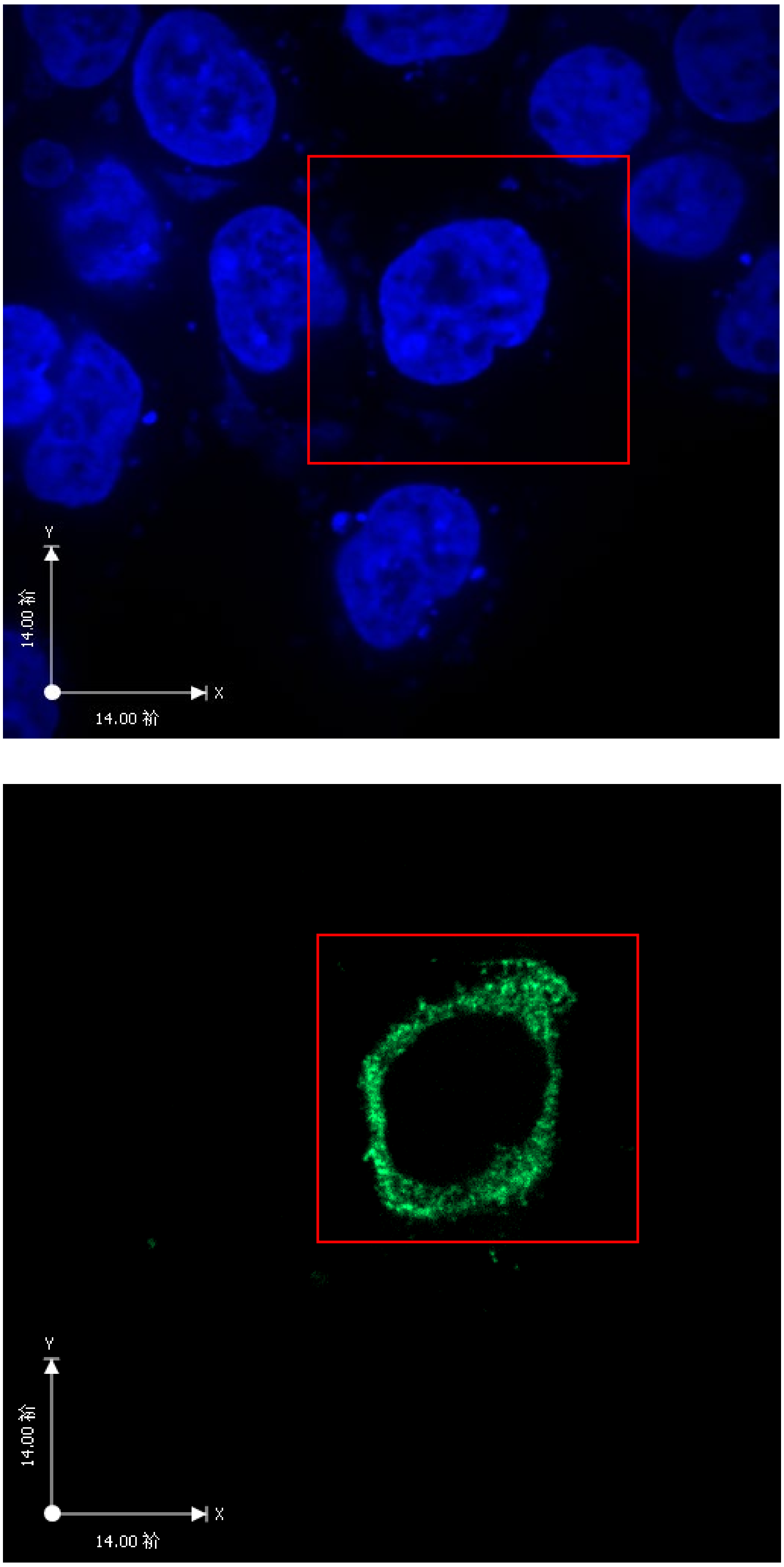

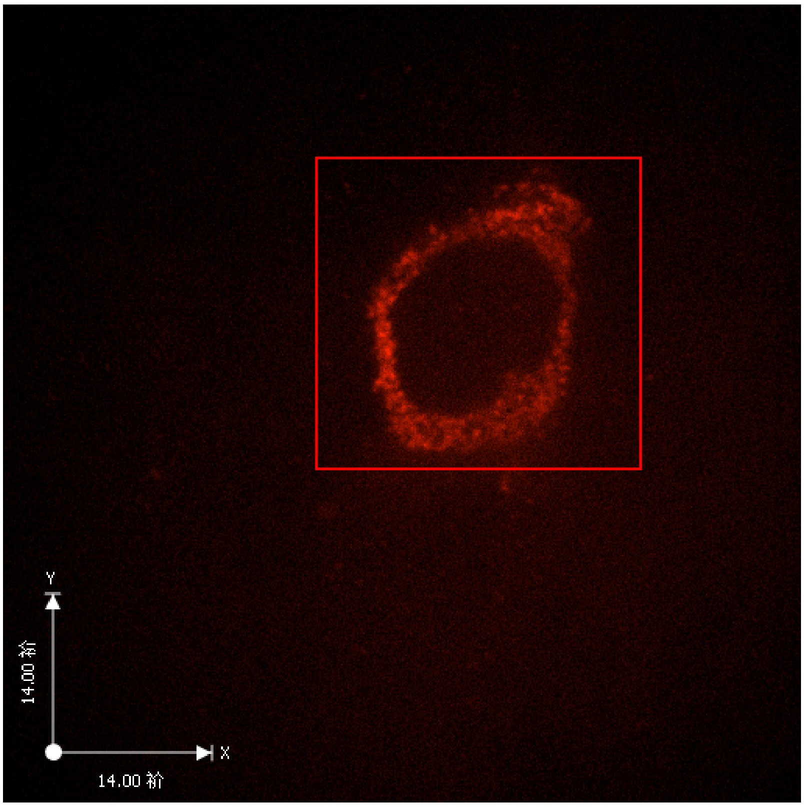
Y
14.00 祄
X
14.00 祄

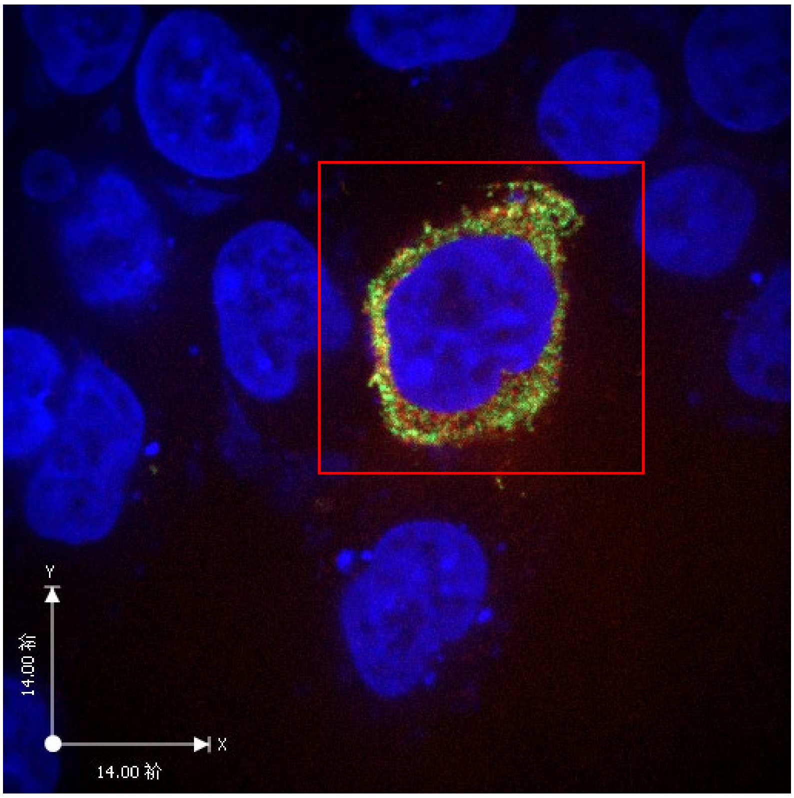
Y
14.00 祄
X
14.00 祄

c

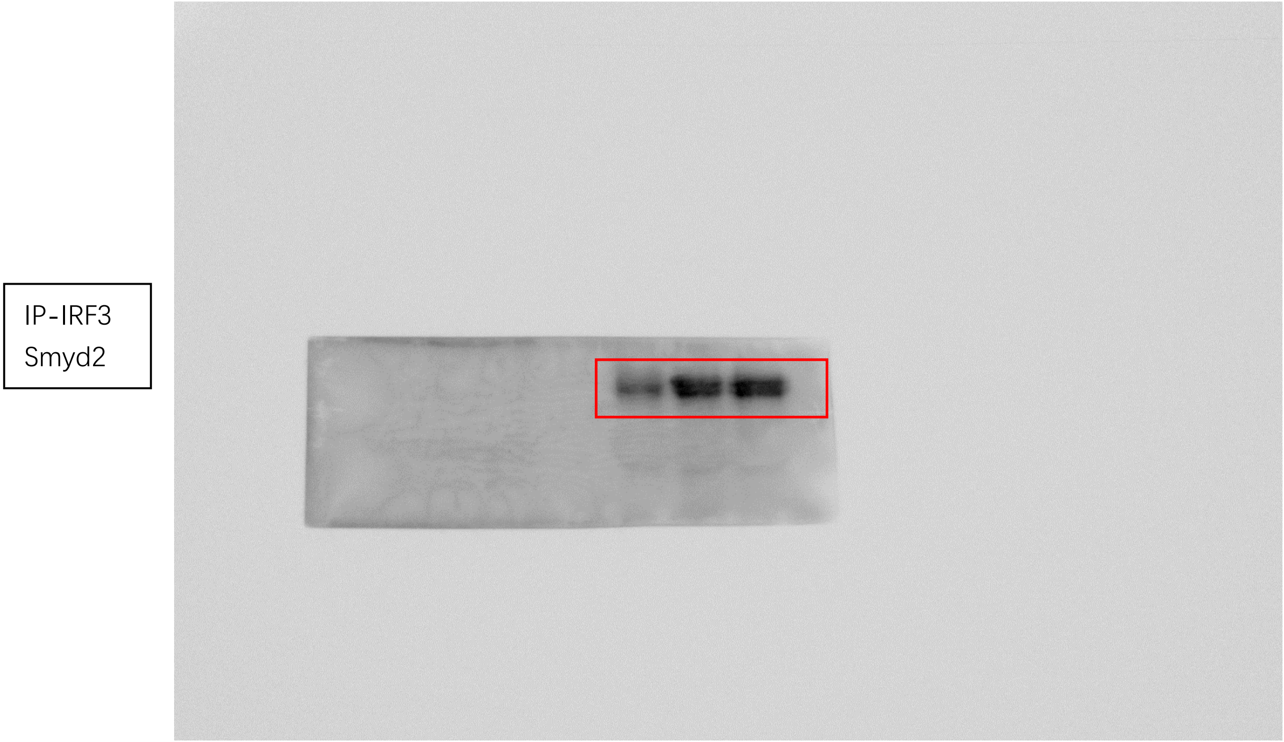

input
Smyd2

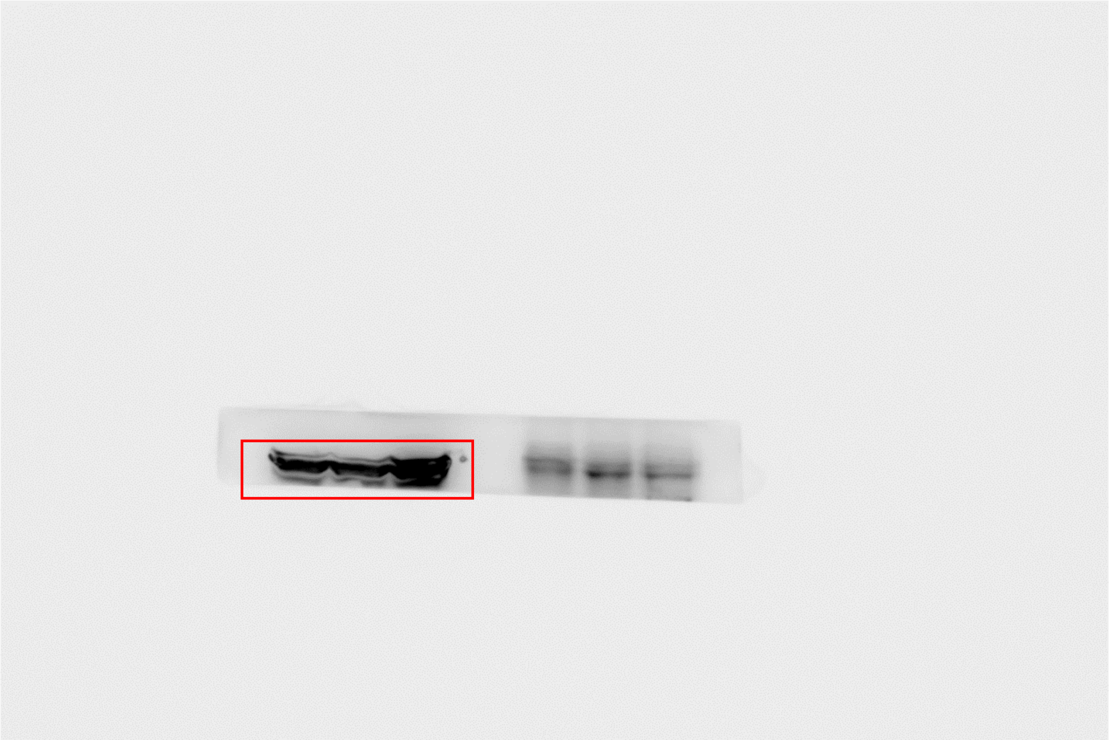

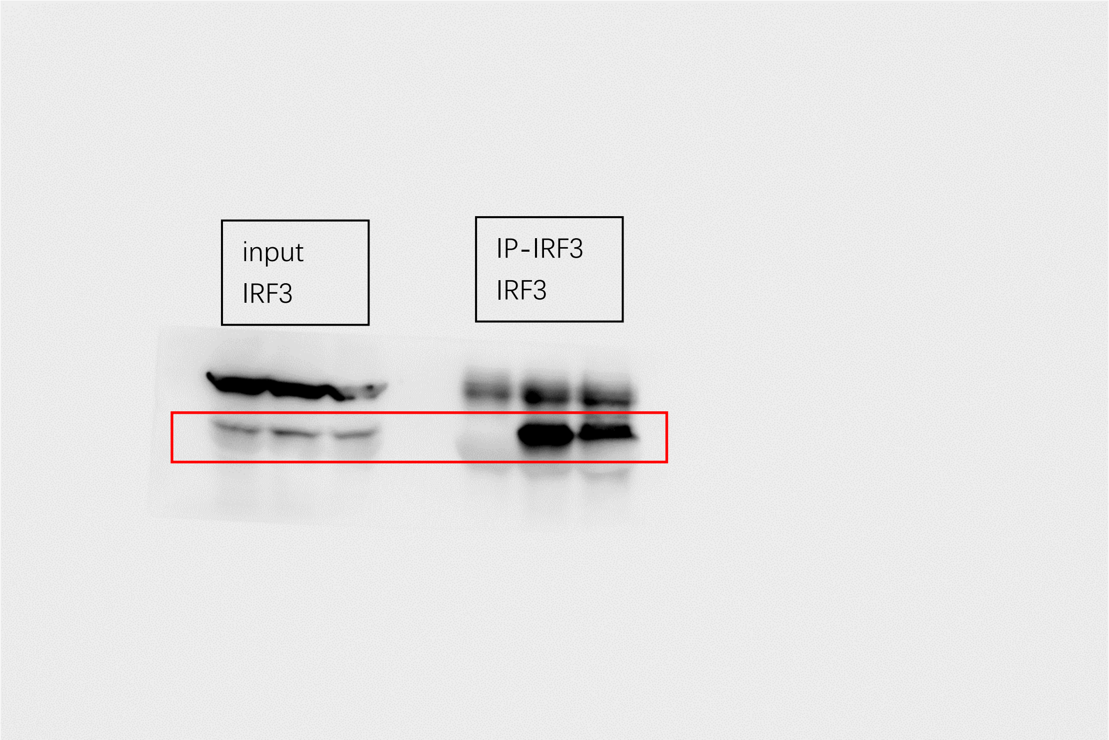

D

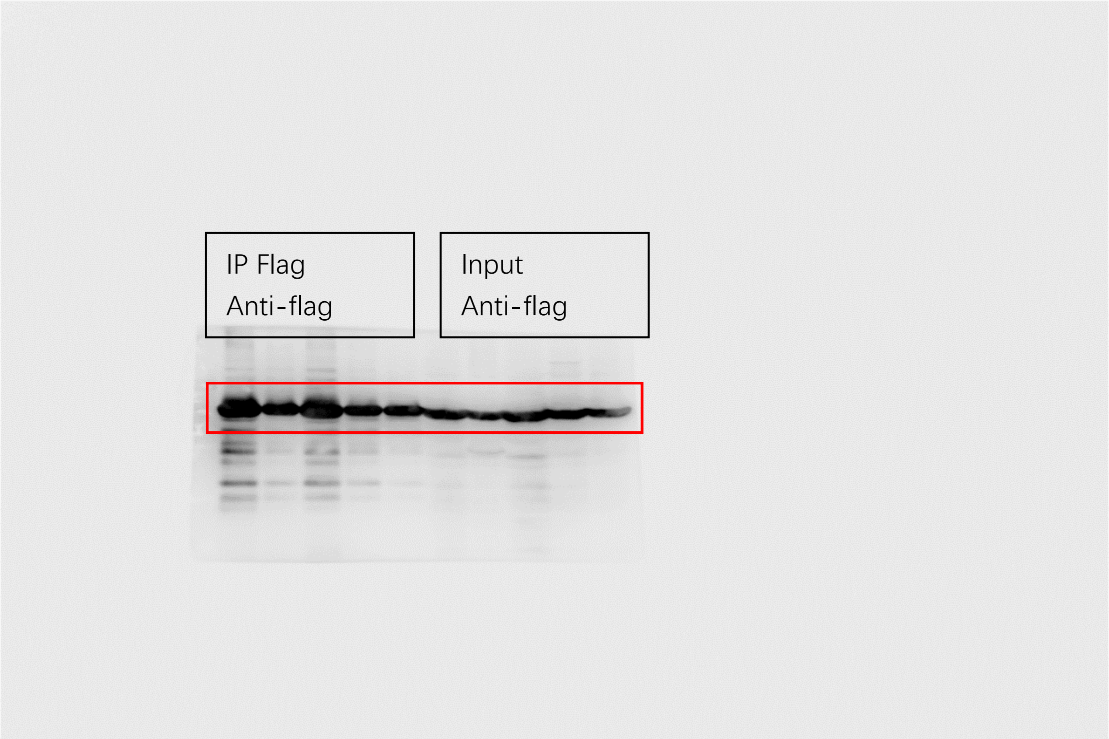
IP Flag
Anti-flag
Input
Anti-flag

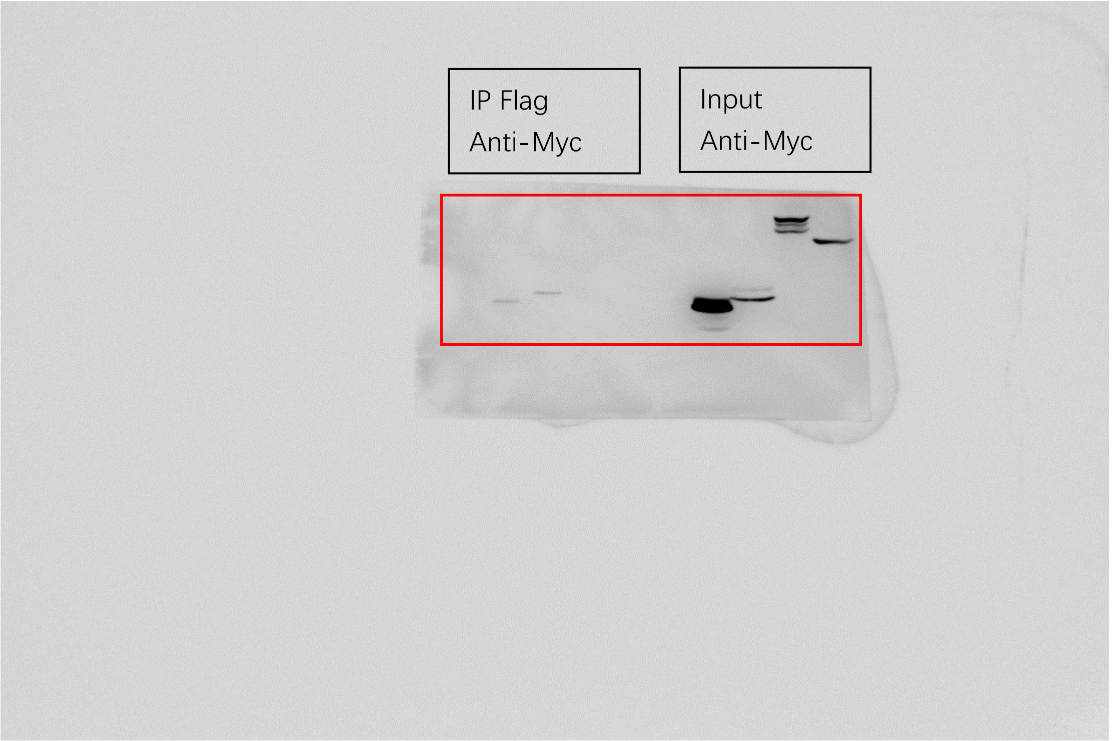
IP Flag
Anti-Myc
Input
Anti-Myc

E

Input
Anti-Myc

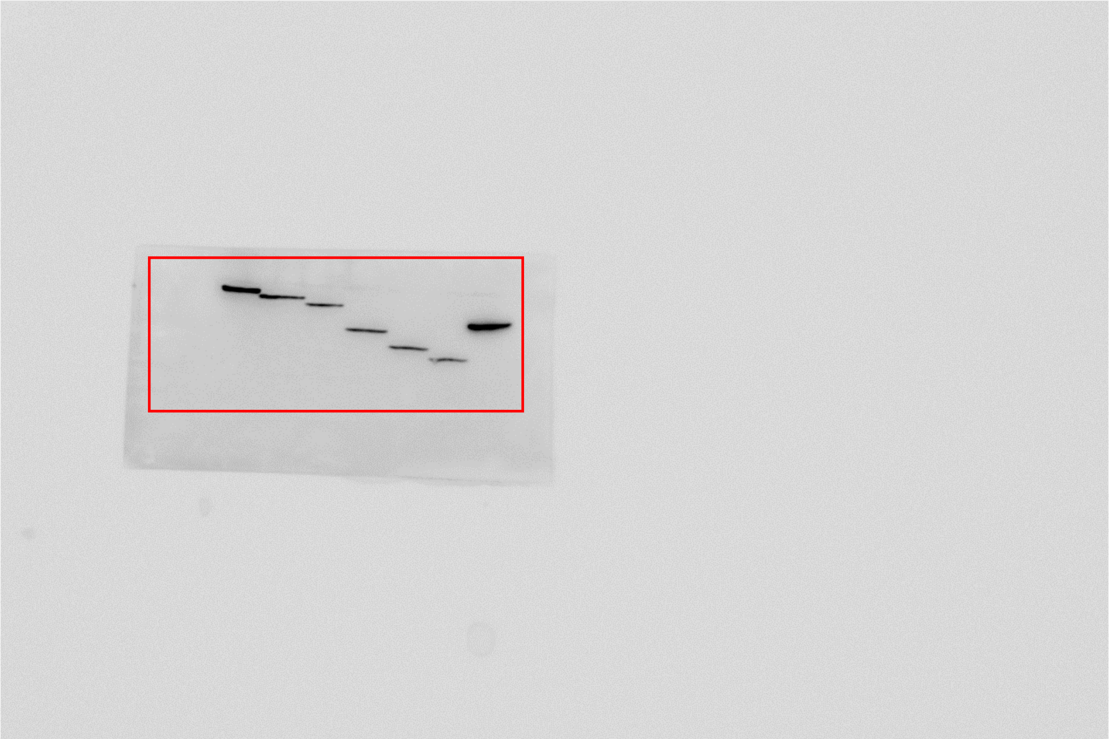

IP Flag
Anti-Myc

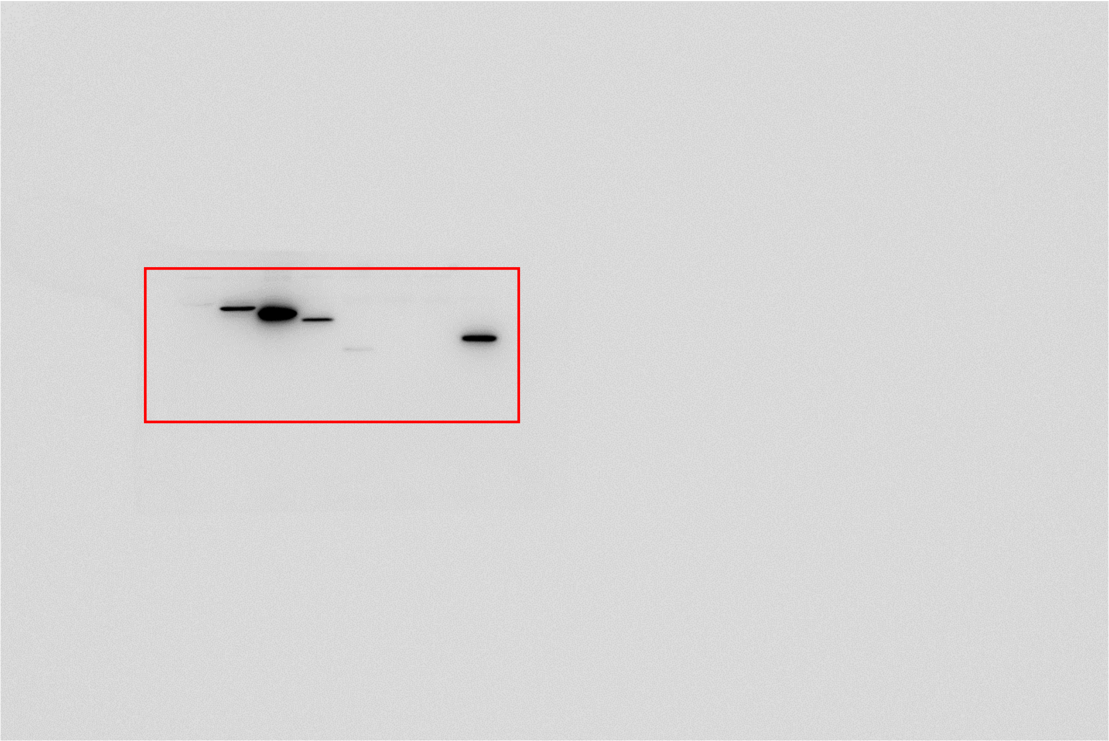

Input
Anti-Flag

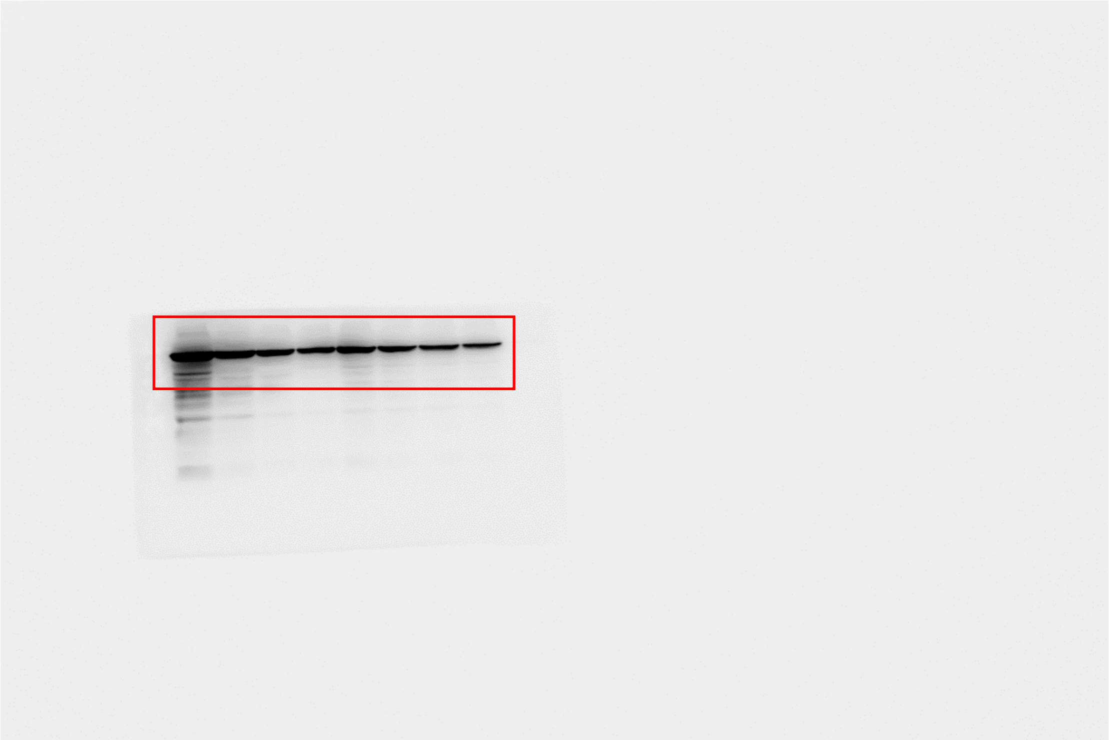

IP Flag
Anti-Flag

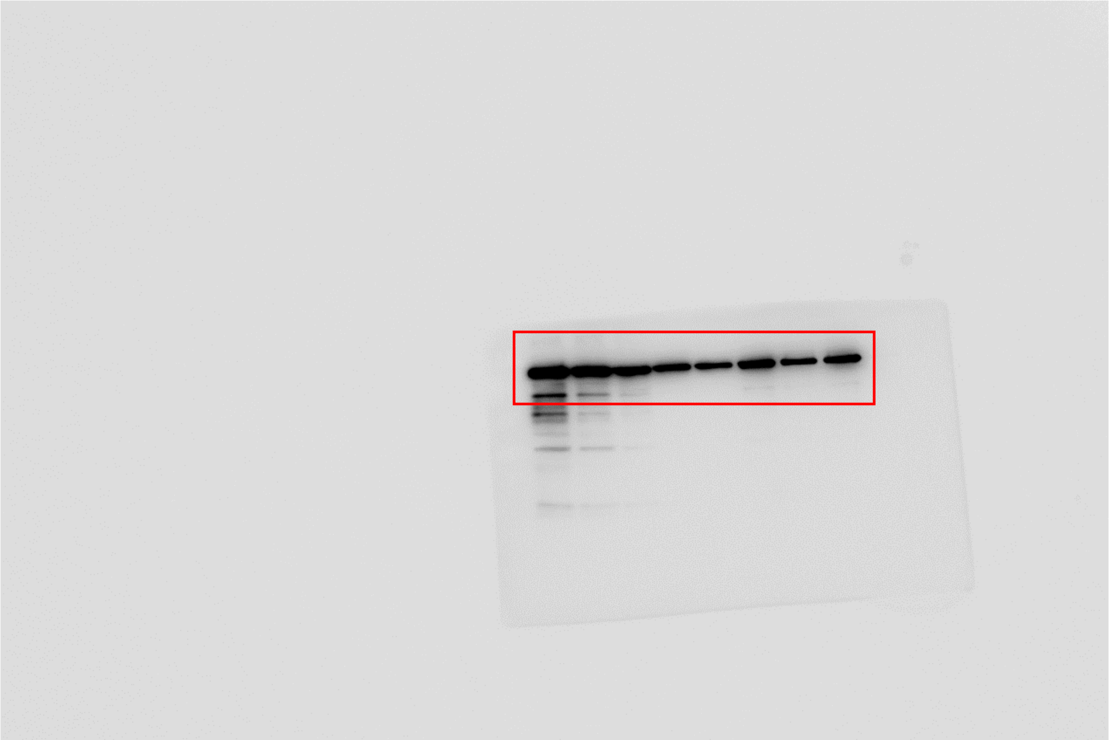

F

IP Flag
Anti-Myc

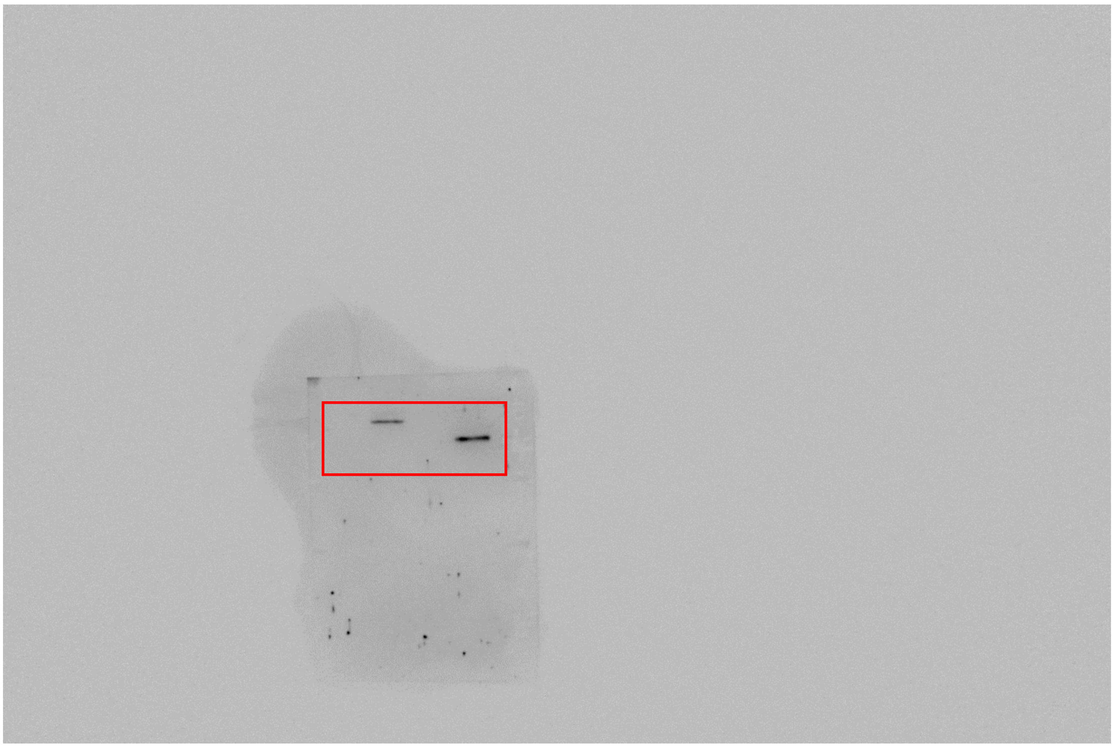

Input
Anti-Myc

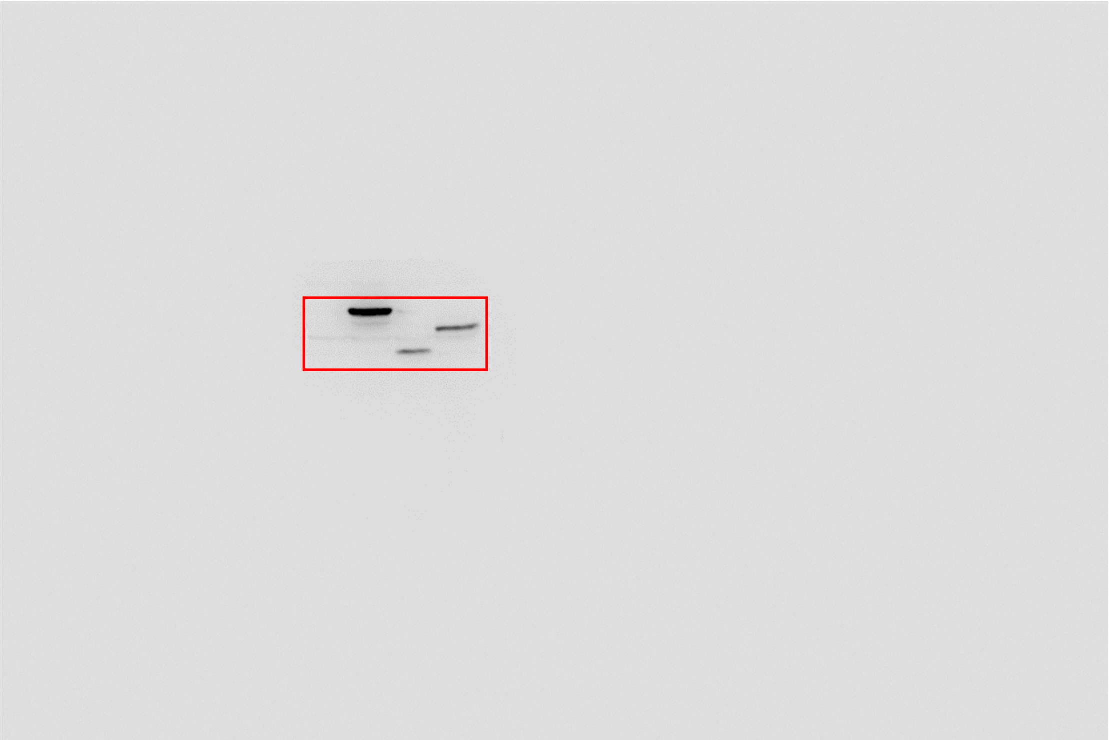

IP Flag
Anti-Flag

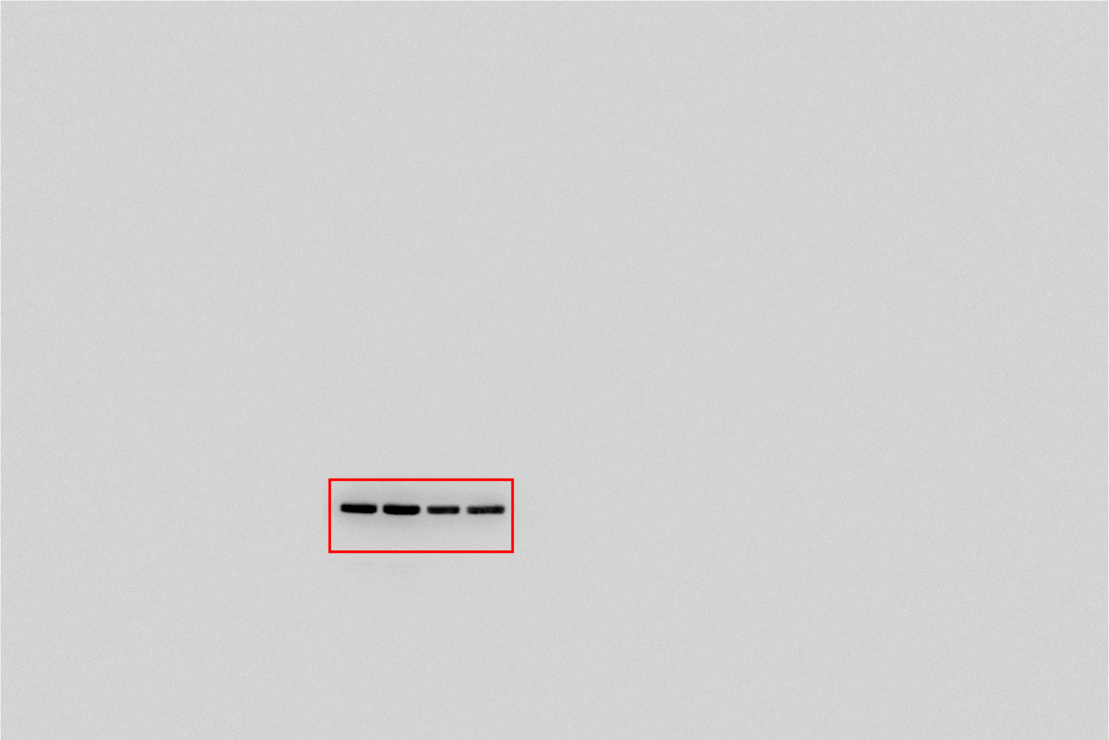

Input
Anti-Flag

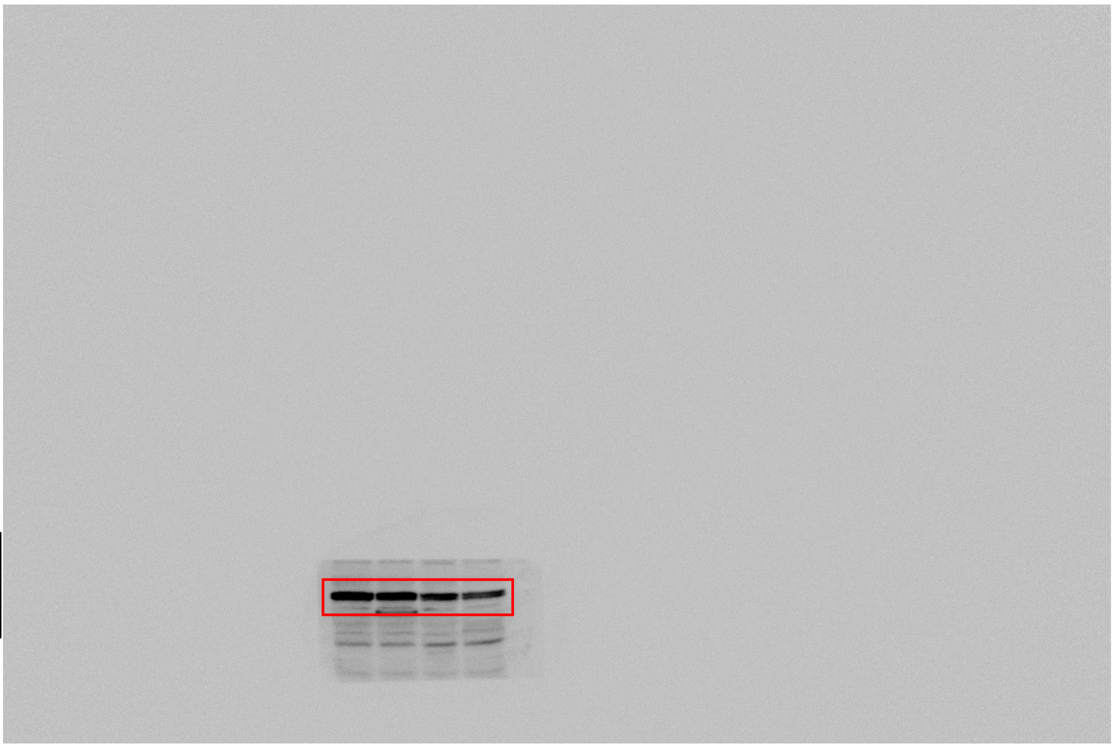

G

p-IRF3

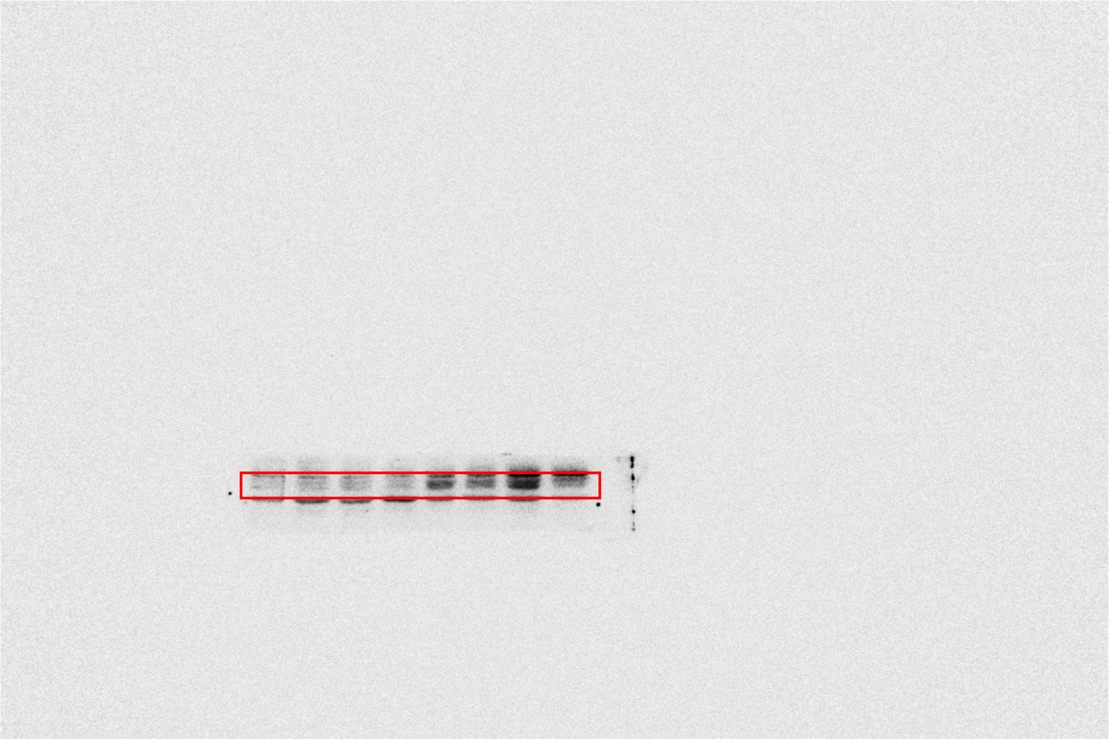

IRF3

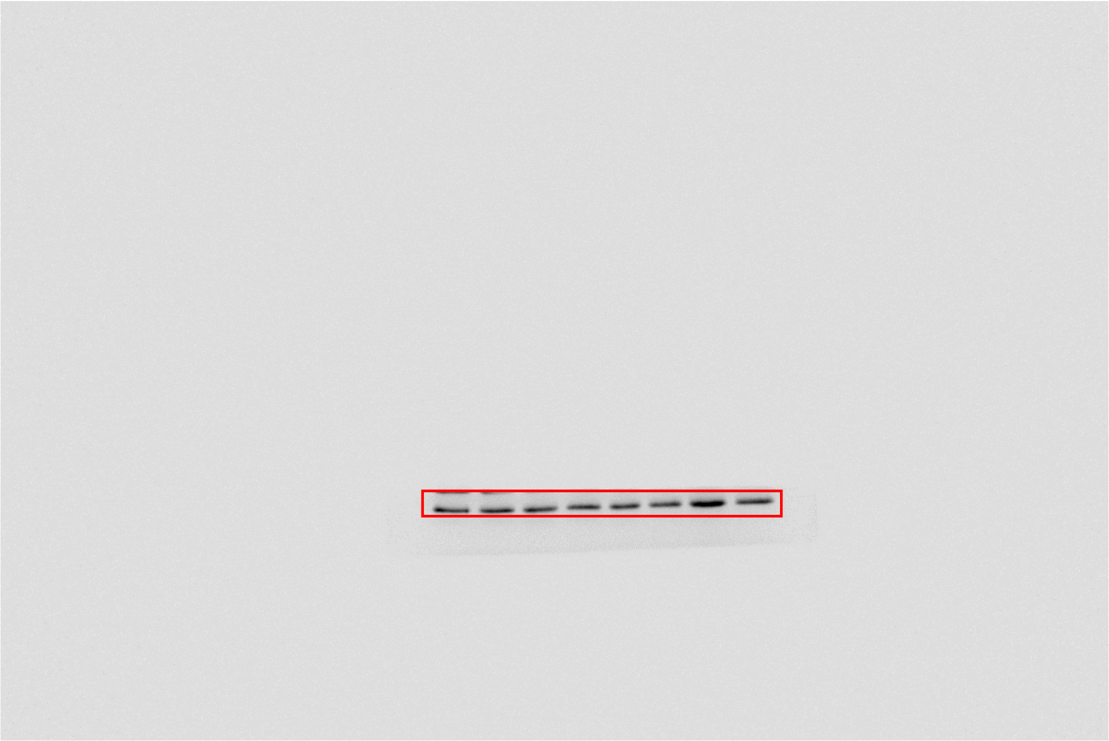

GAPDH

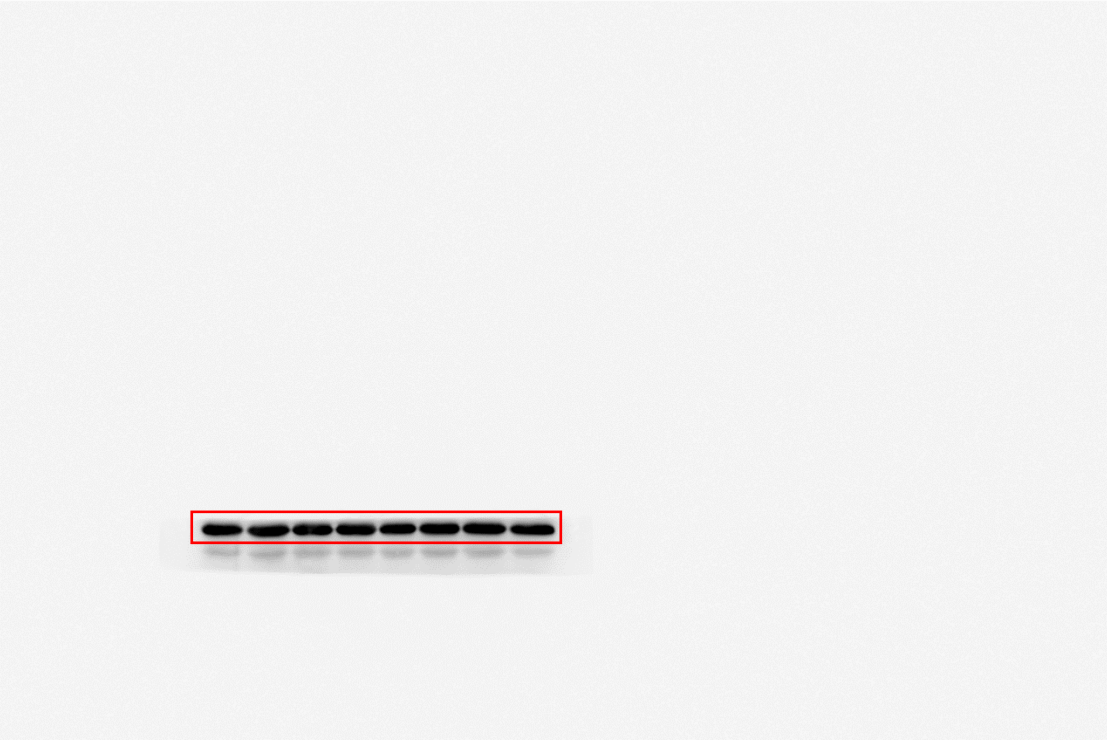

I

Input
Anti-Myc

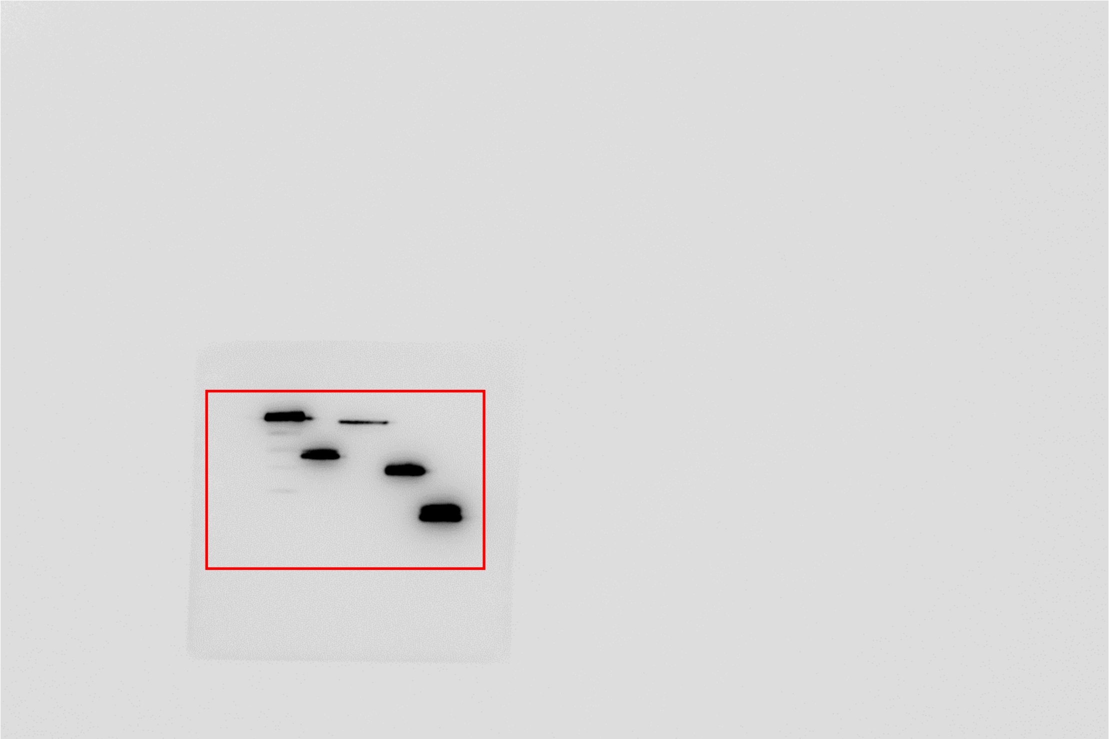

IP Flag
Anti-Myc

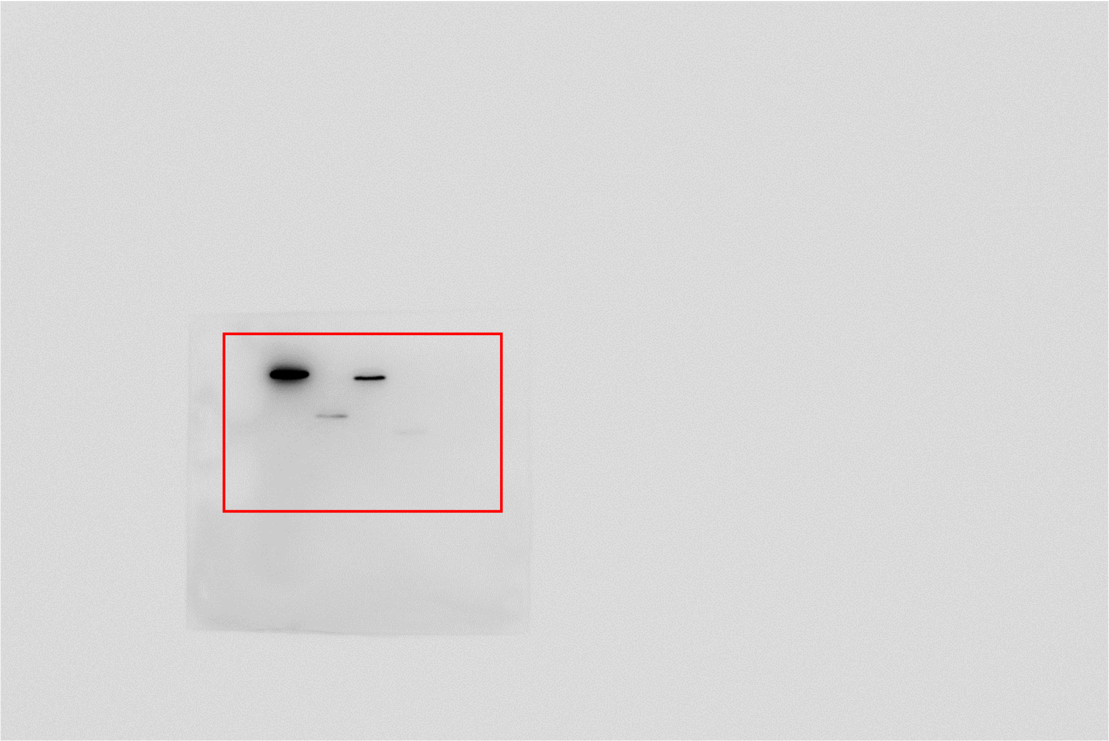

Input
Anti-Flag

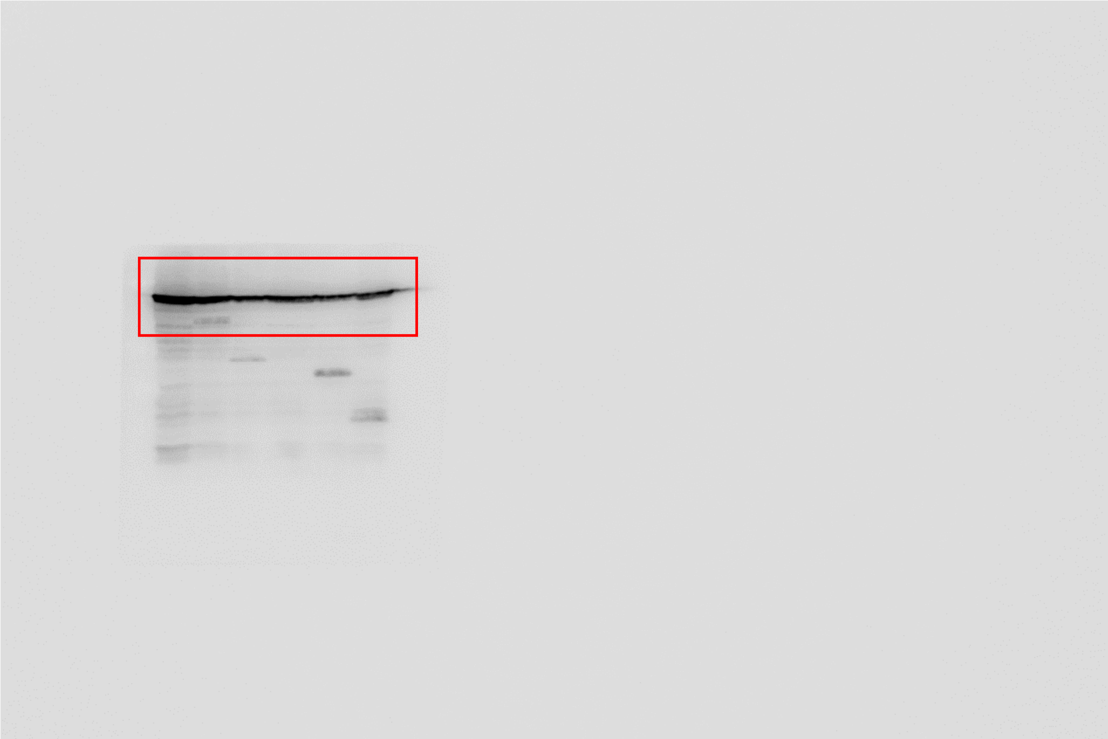

IP Flag
Anti-Flag

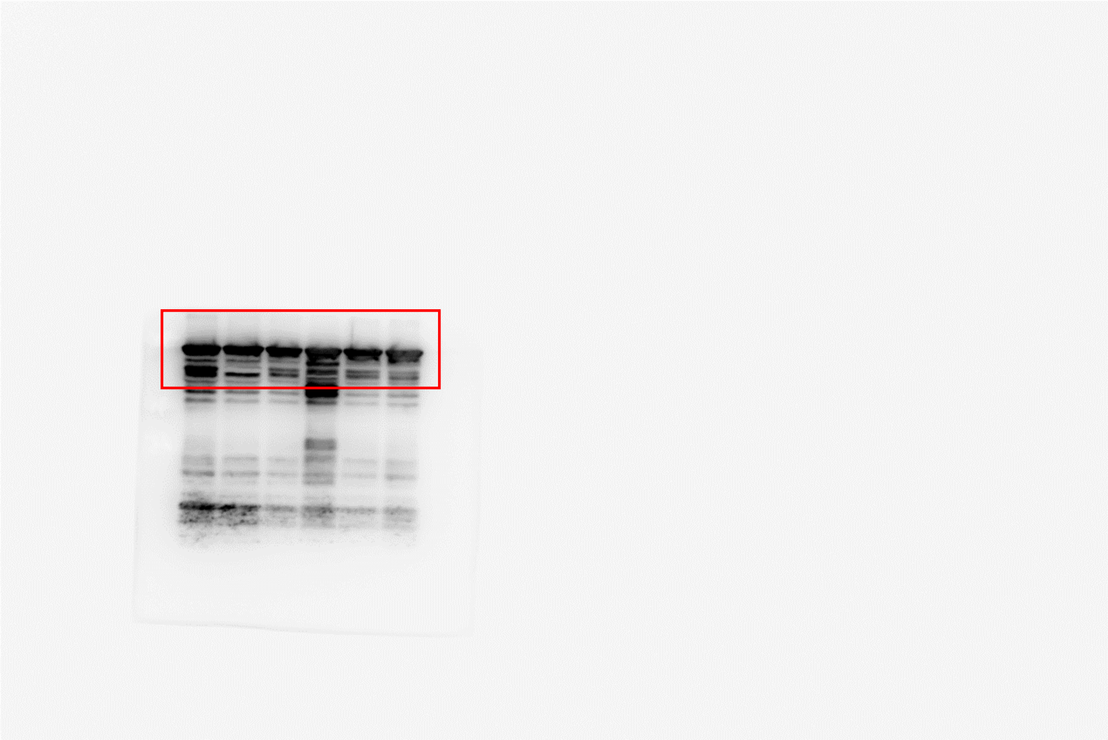

J

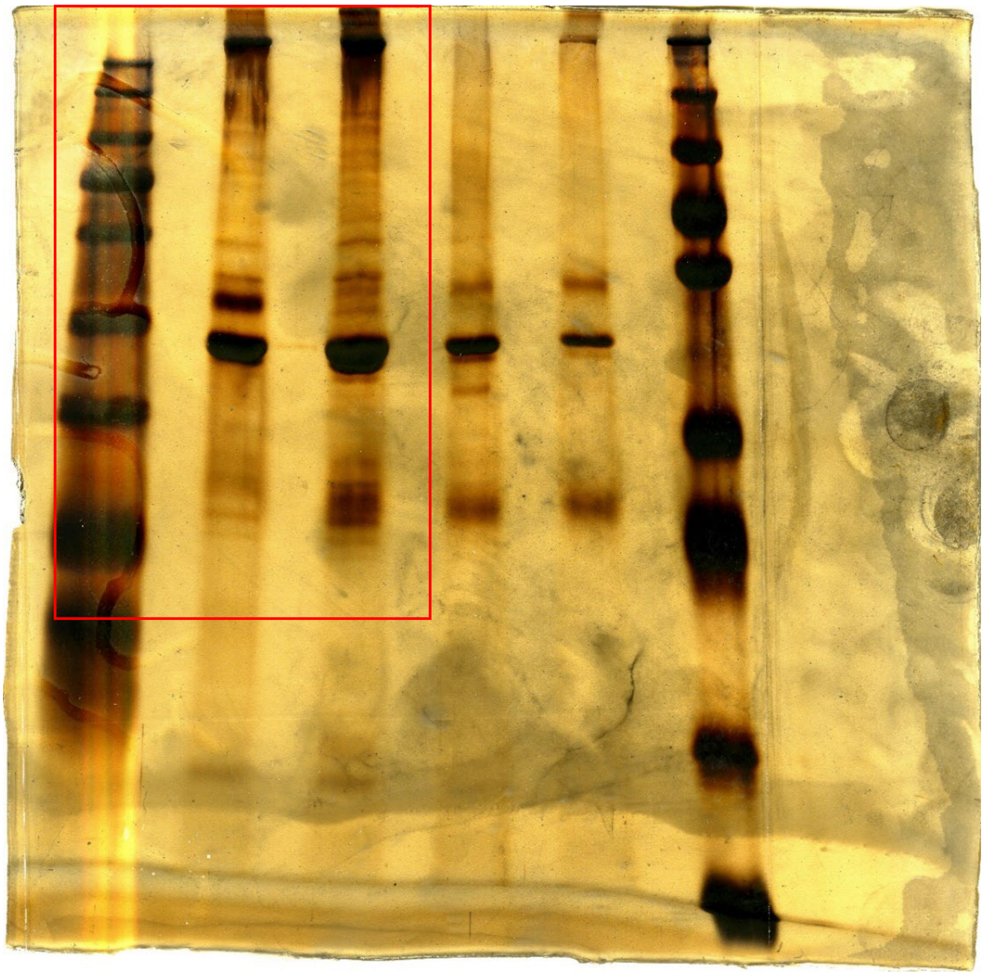

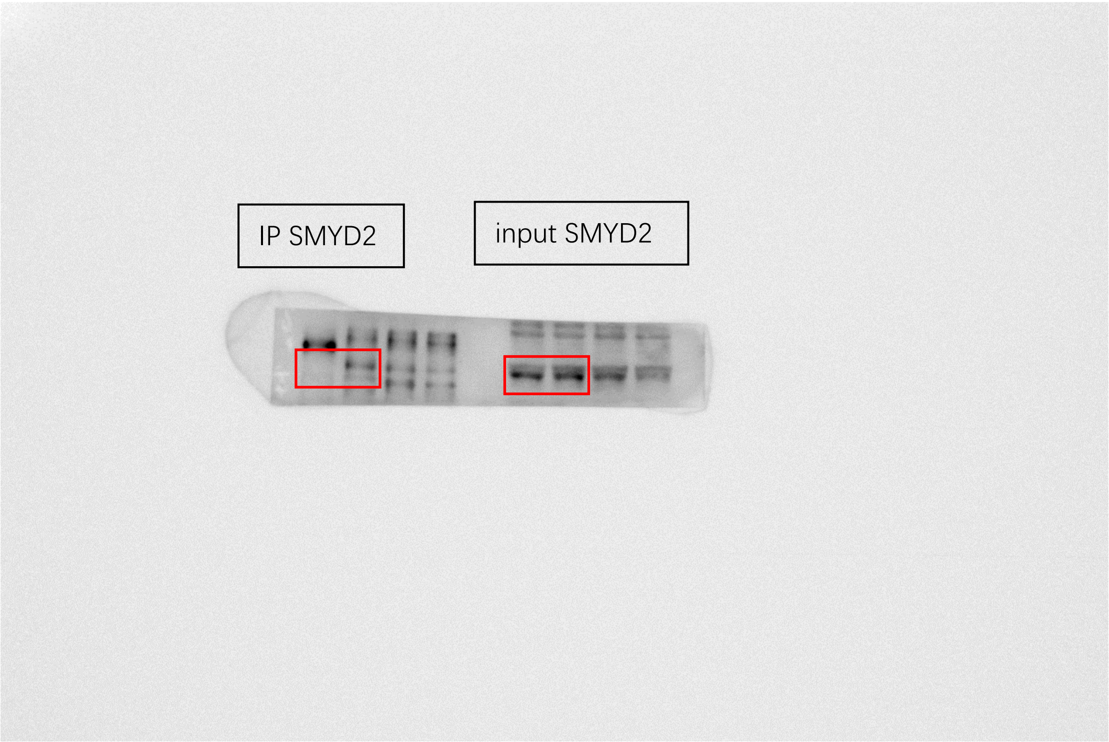

Input
GAPDH

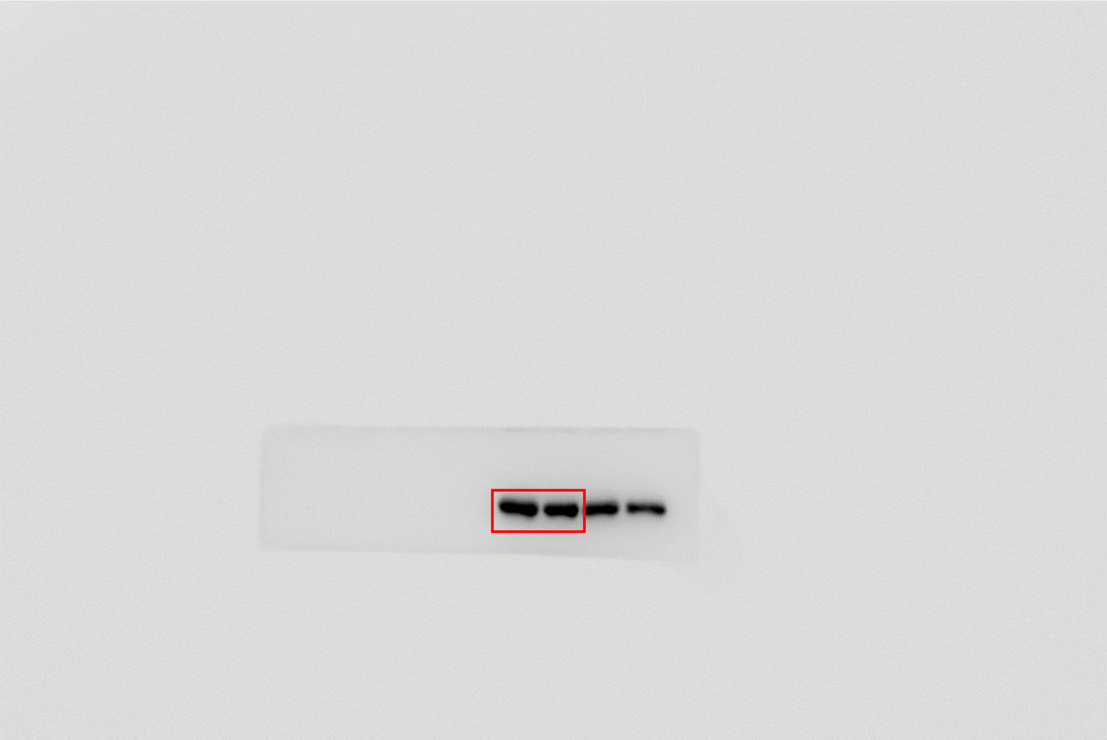

K

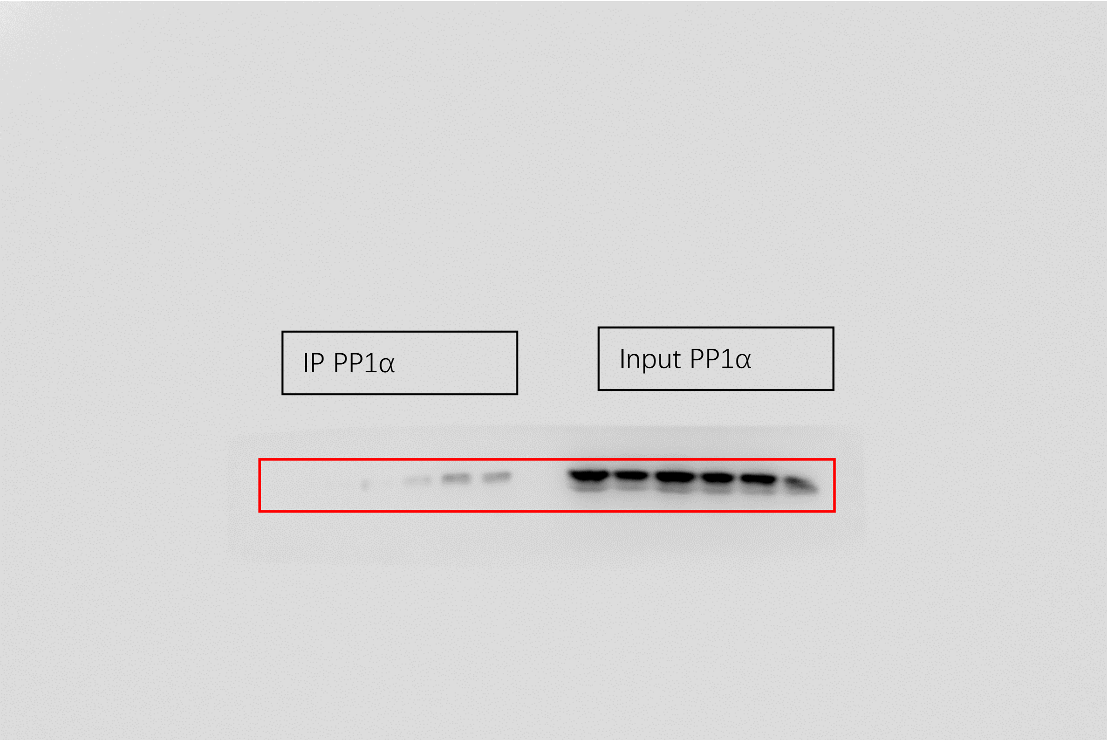

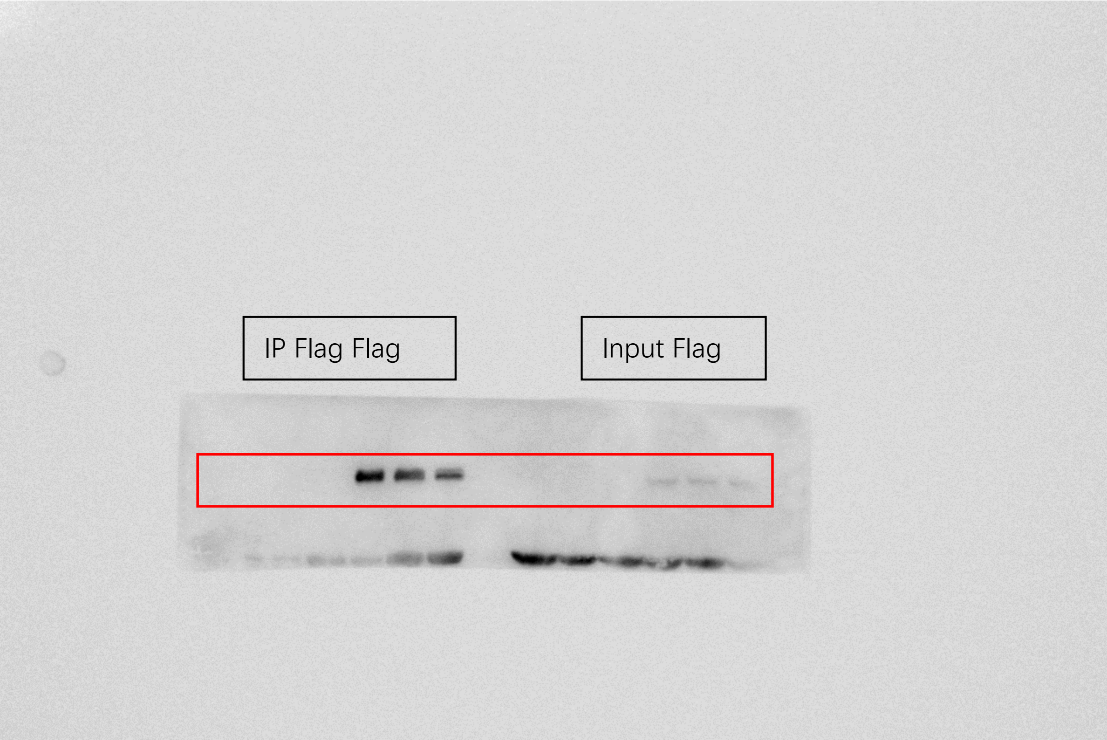

Input GAPDH

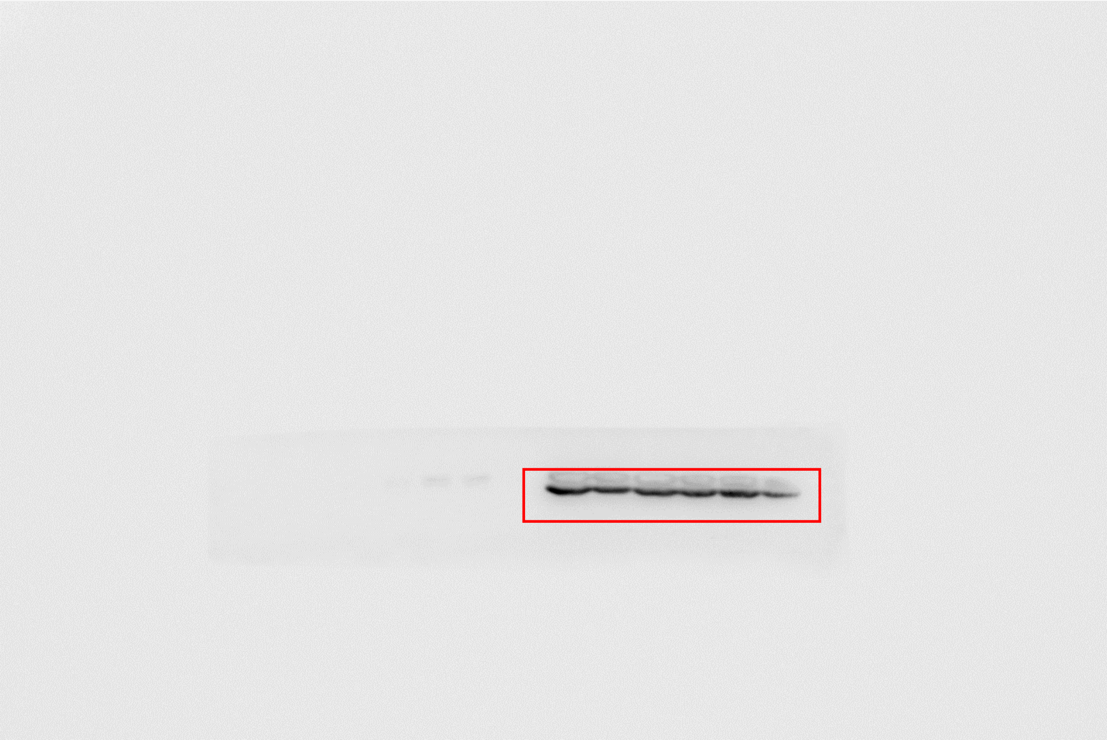

L

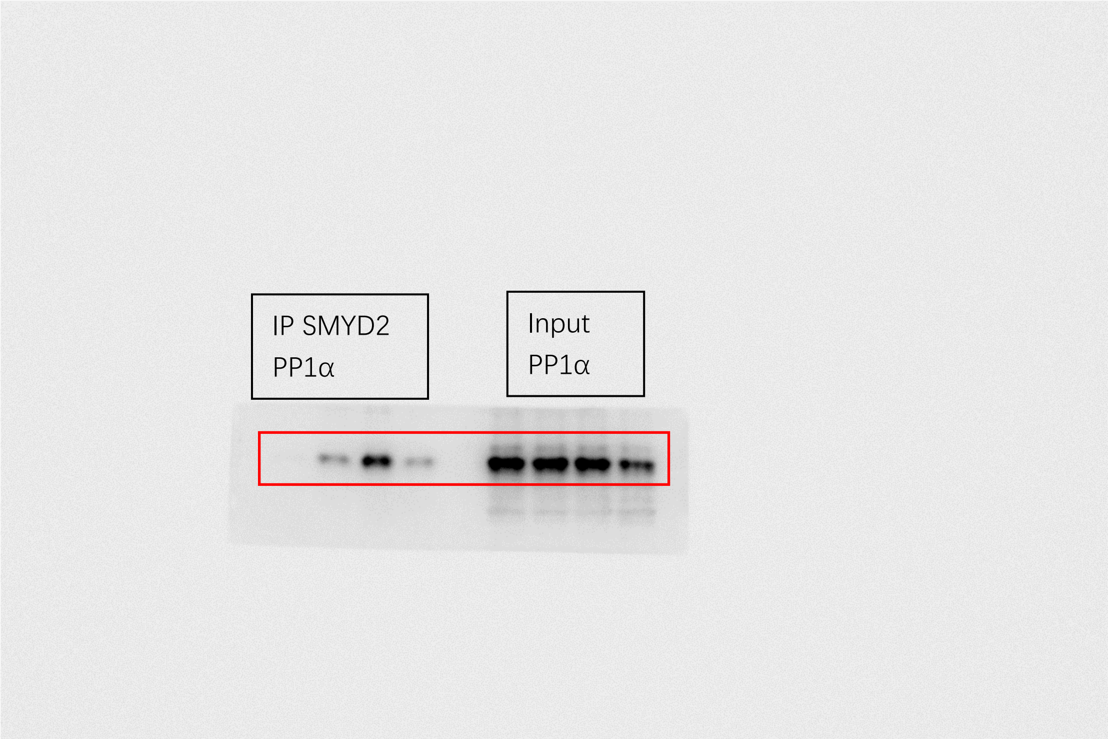

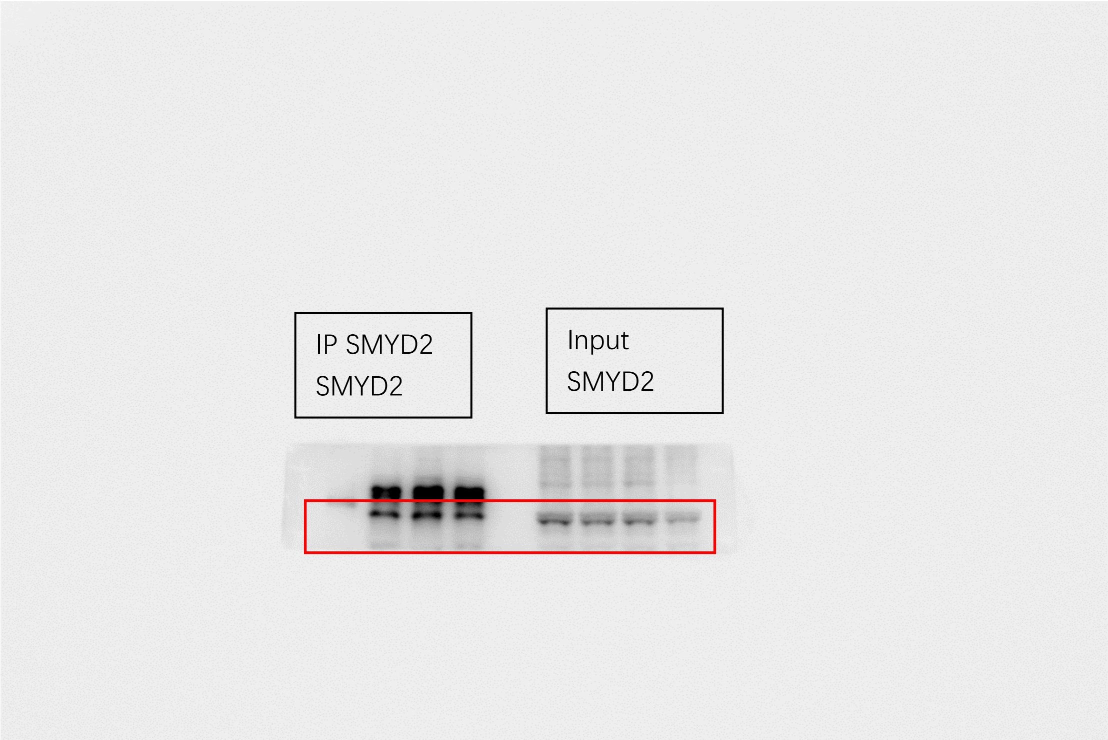

Input
GAPDH

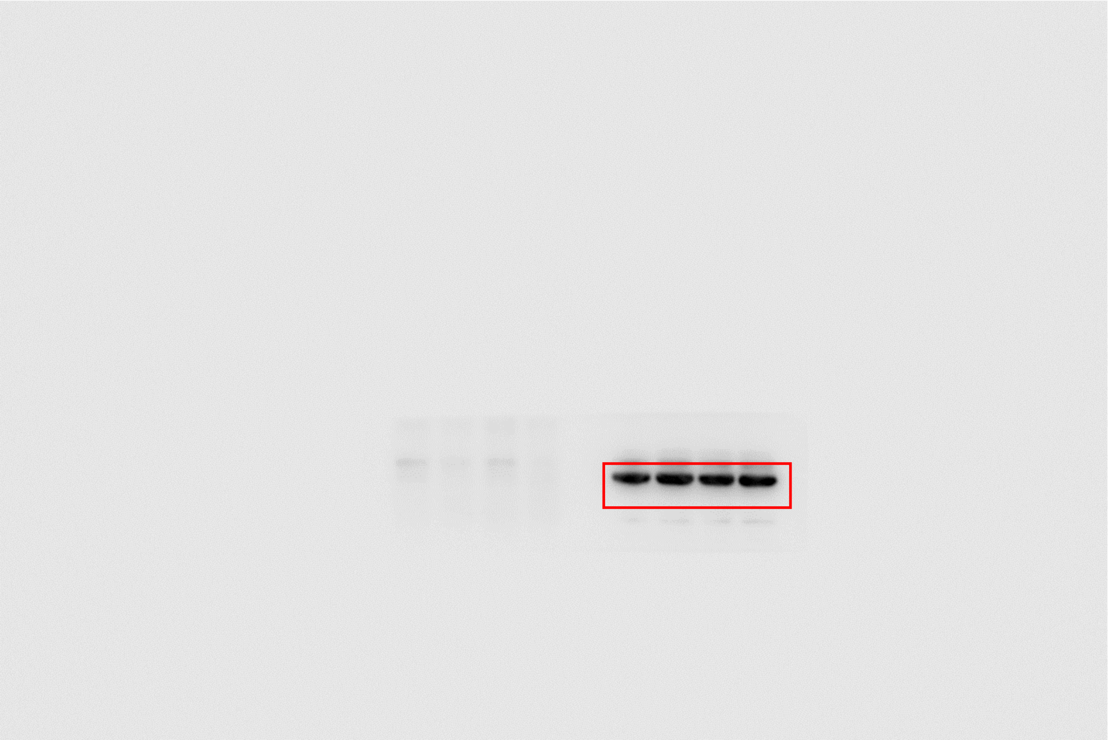

M

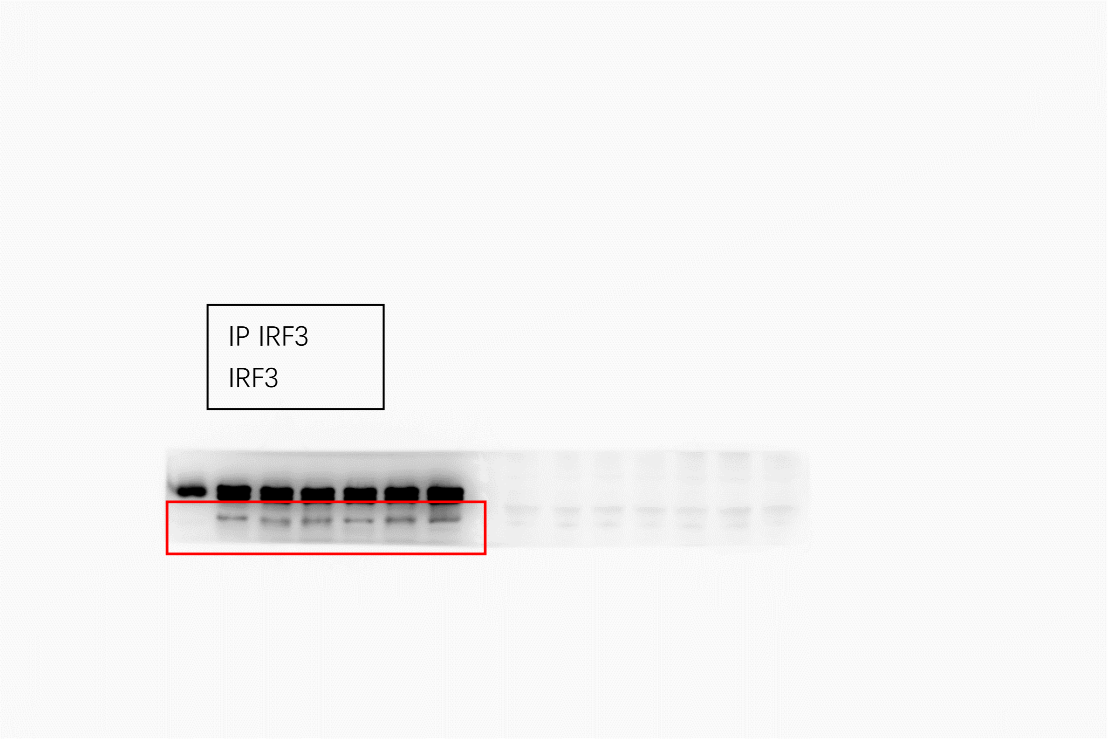

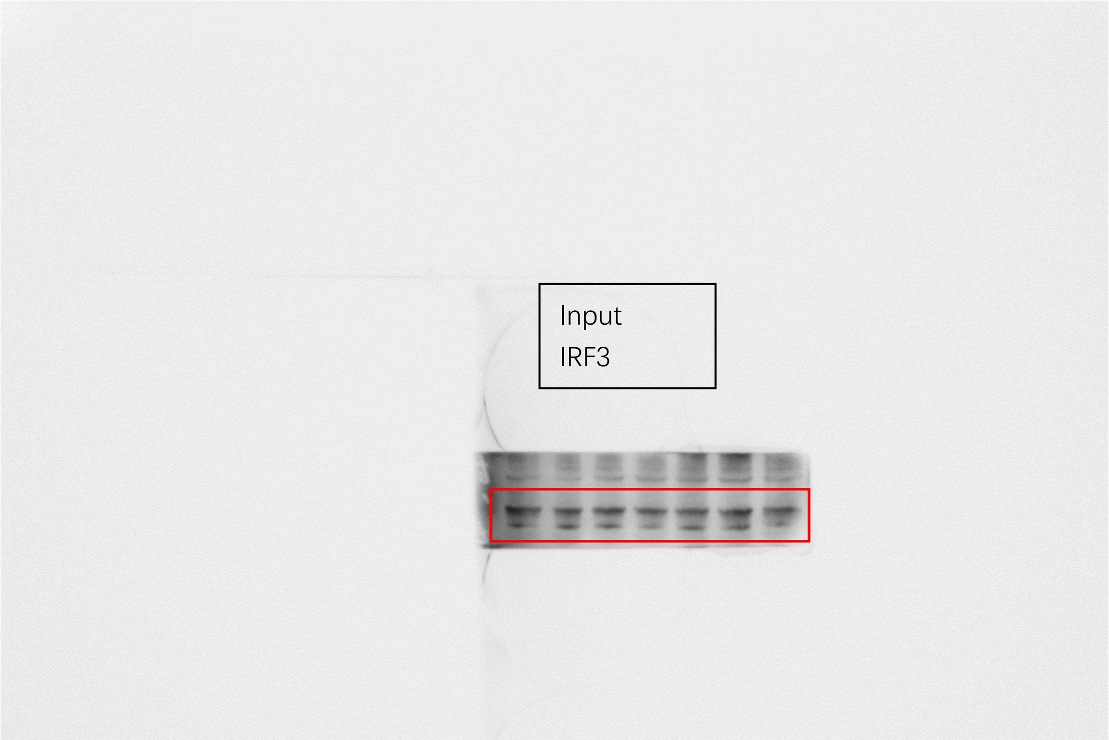
Input
IRF3

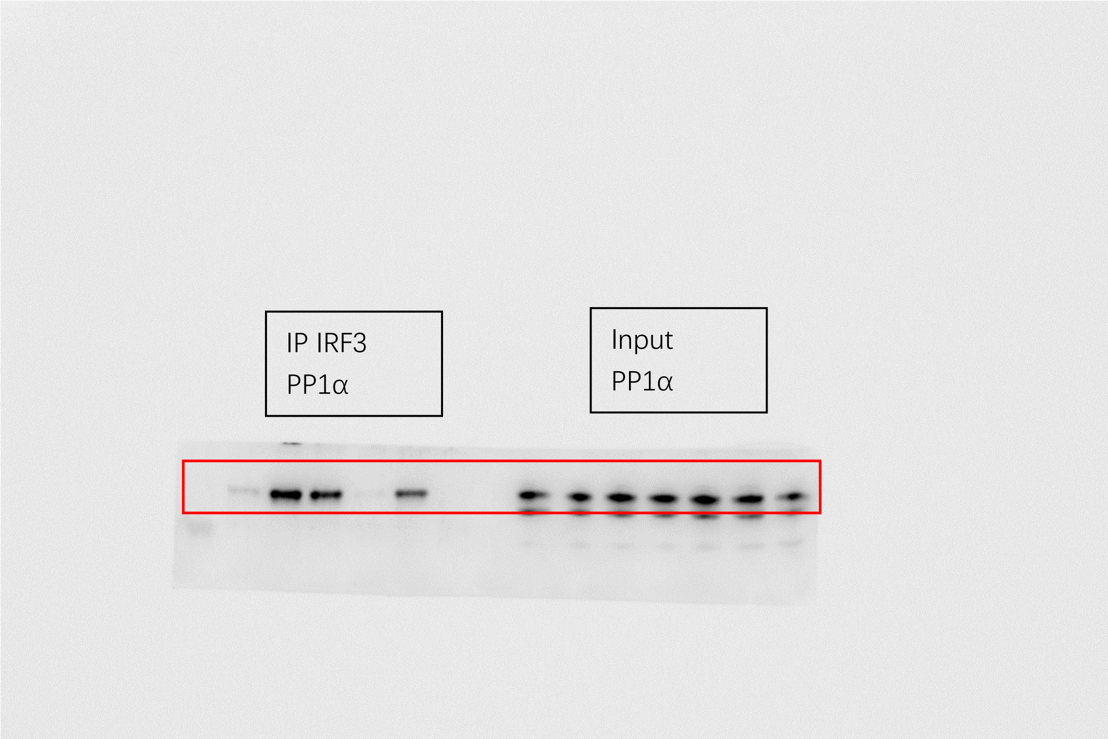

Input
GAPDH

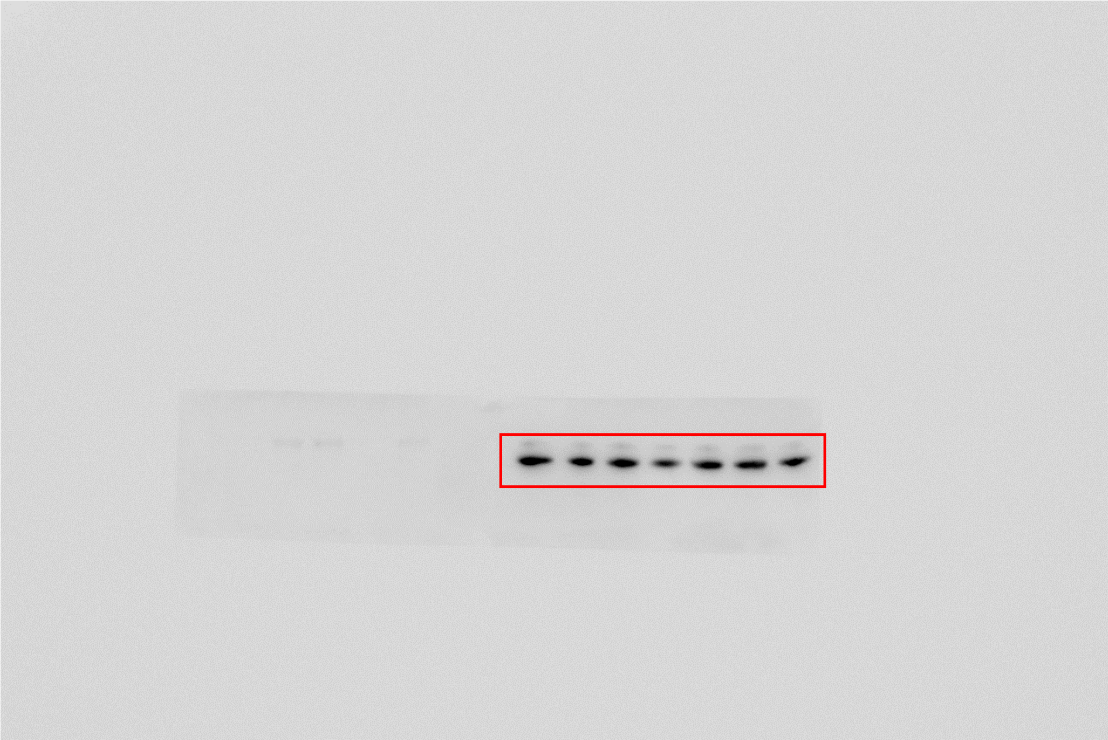

N

IP Flag
Anti-Myc

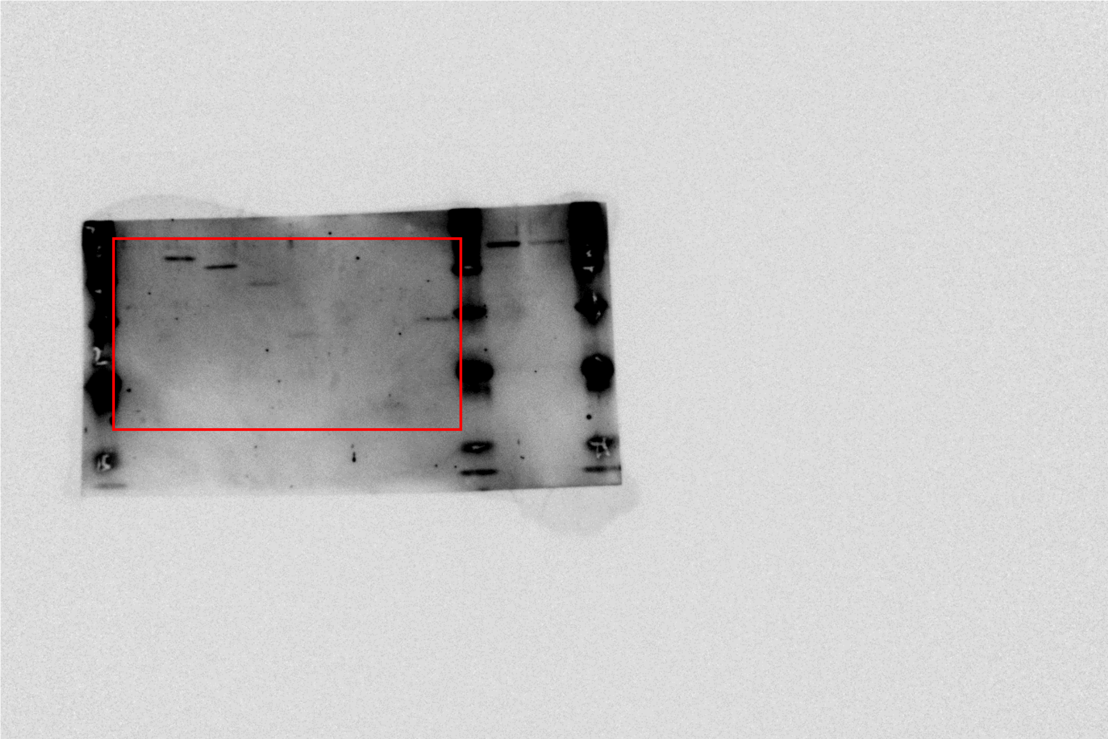

Input
Anti-Myc

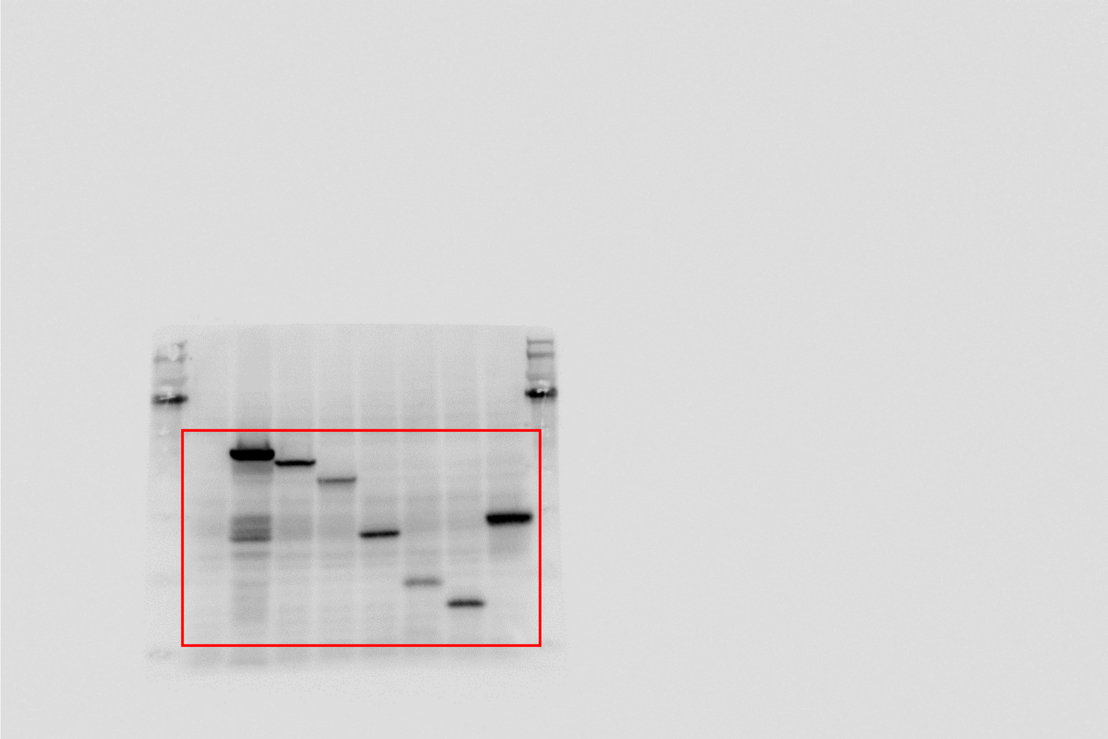

IP Flag
Anti-Flag

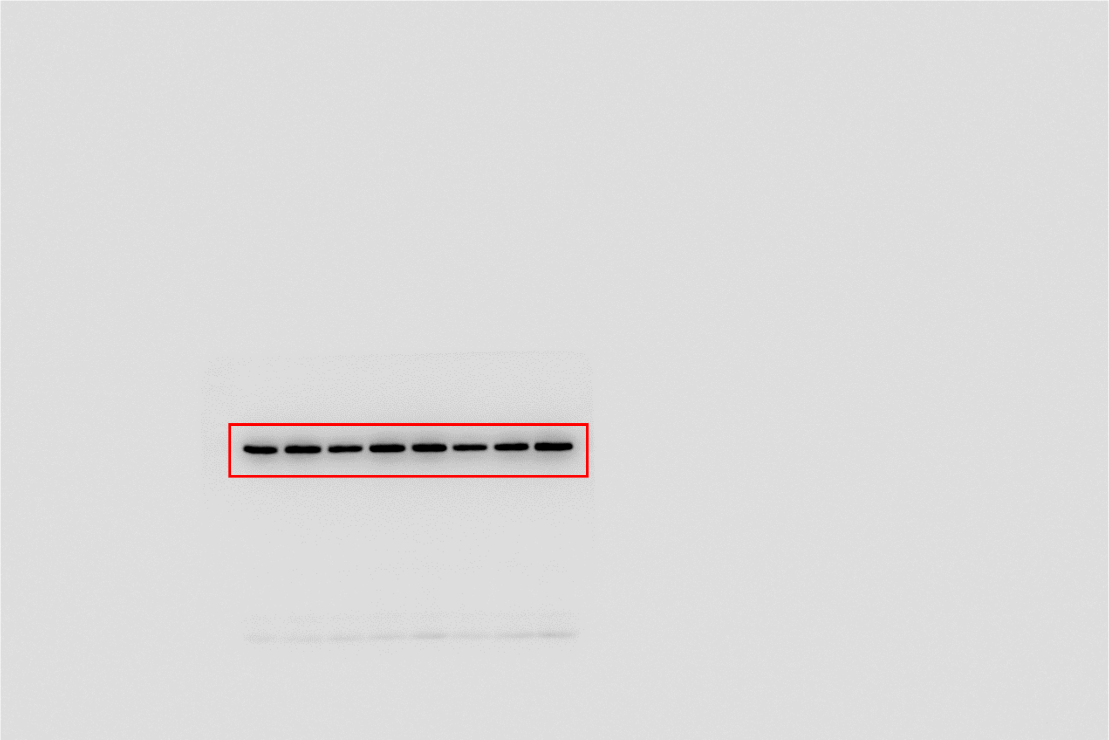

Input
Anti-Flag

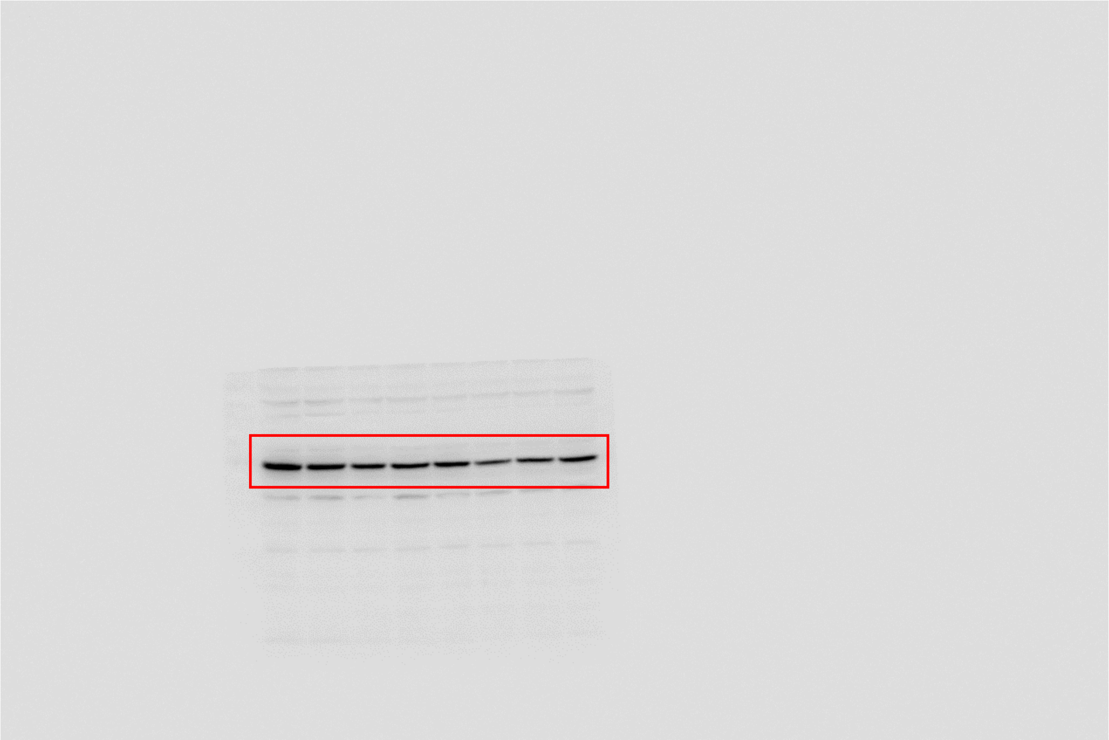

Supplementary Figure 2

D

β-actin

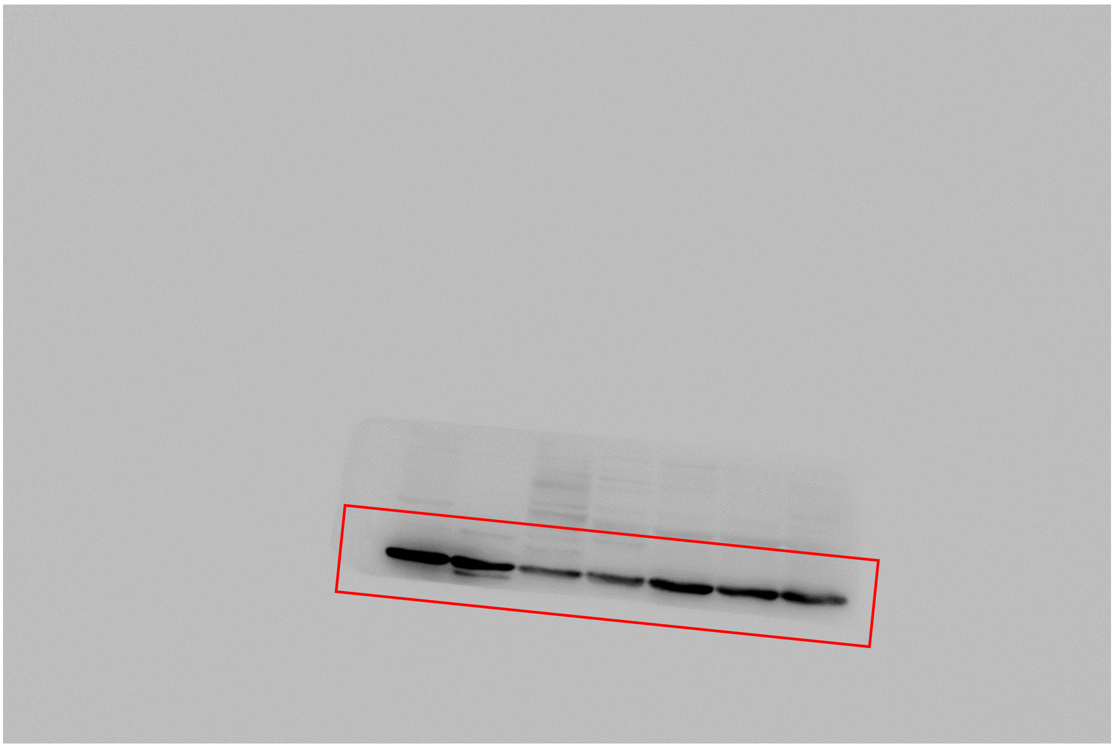

SMYD2

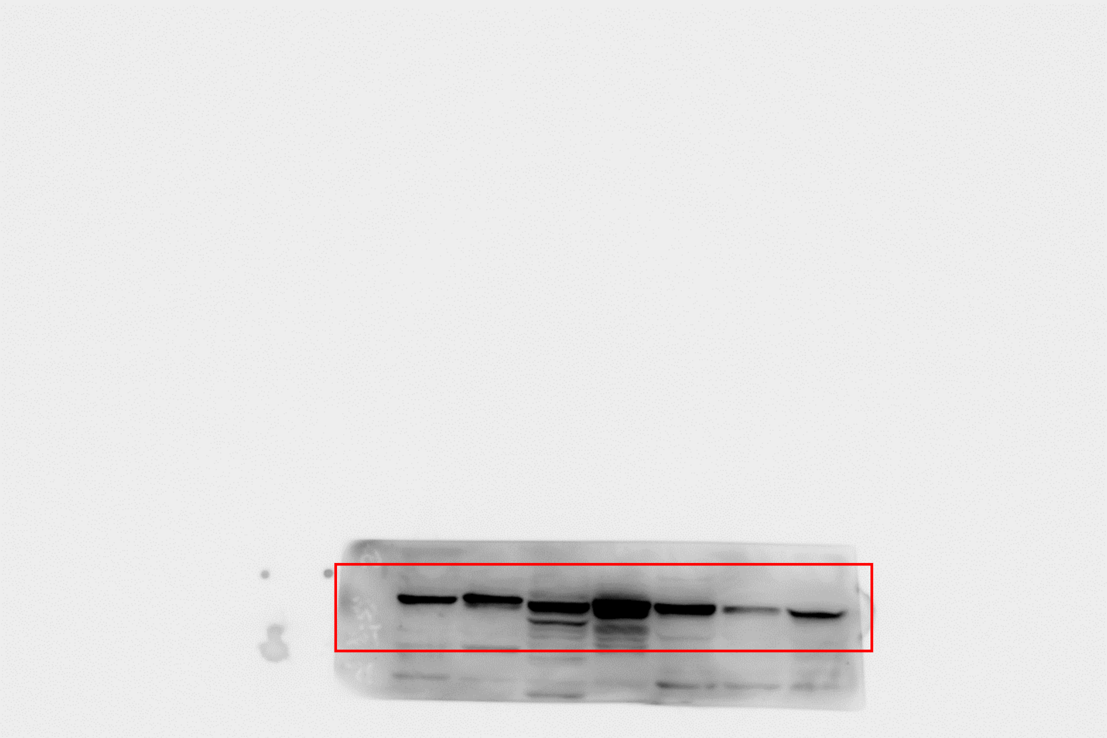

E

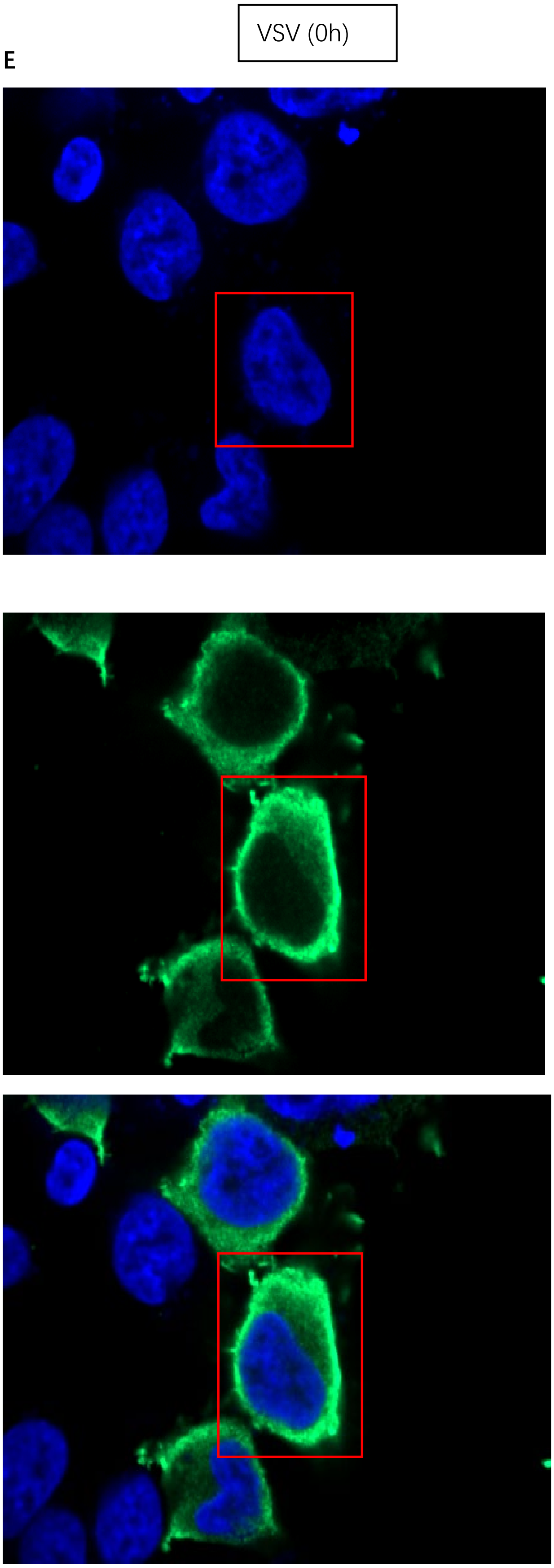

VSV (8h)

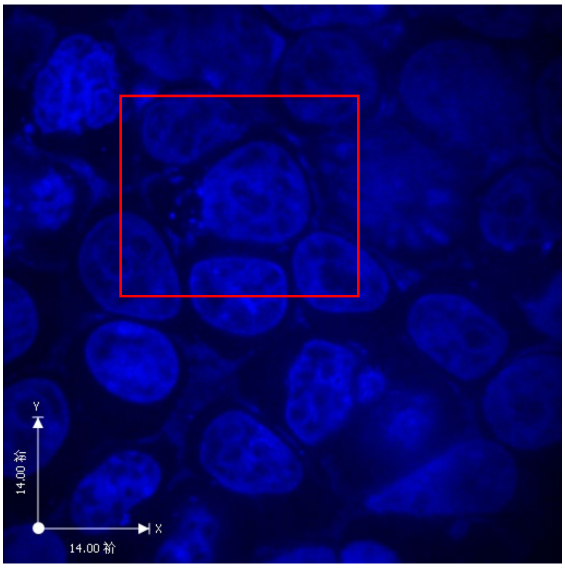

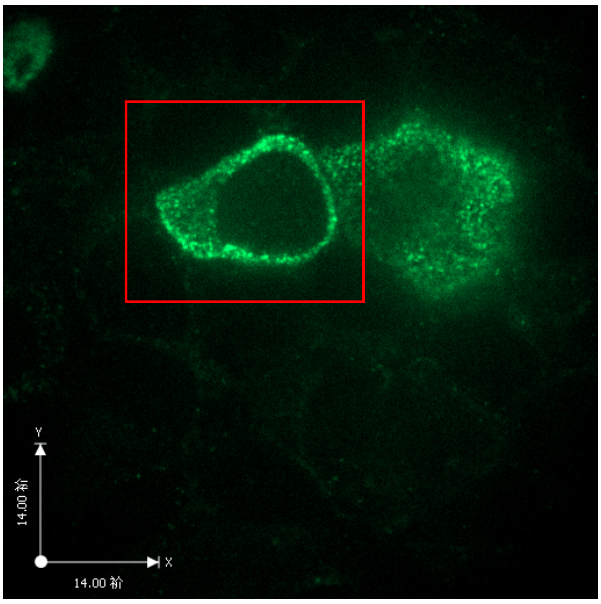

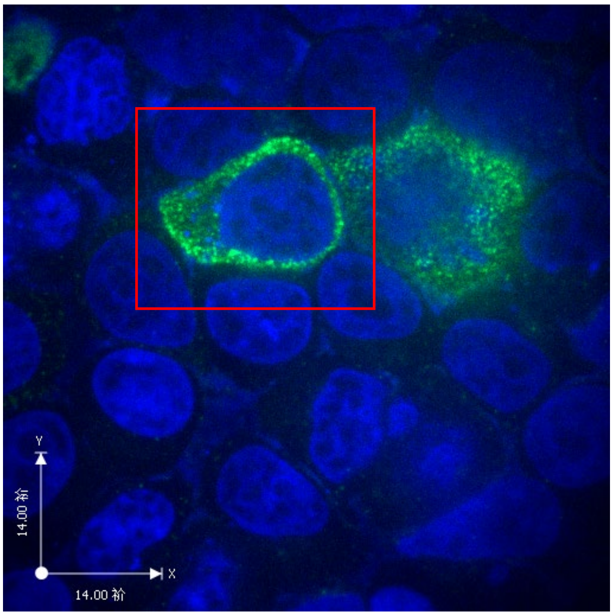

F

VSV (0 h)

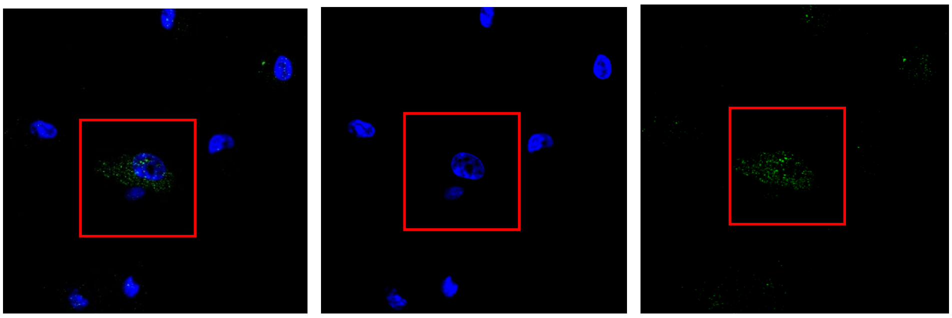

VSV (8 h)

VSV (12 h)

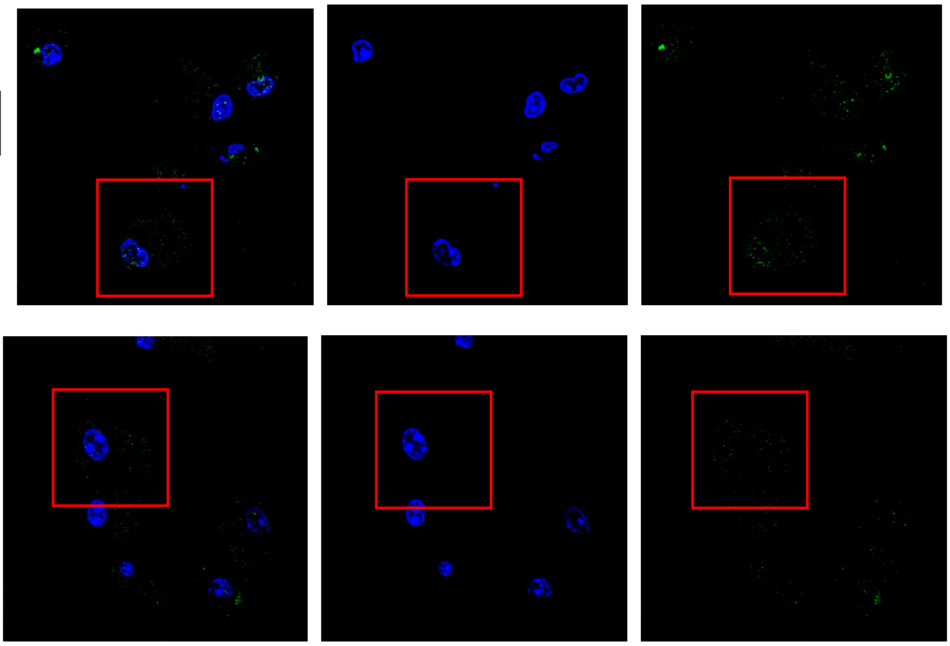

G

β-actin

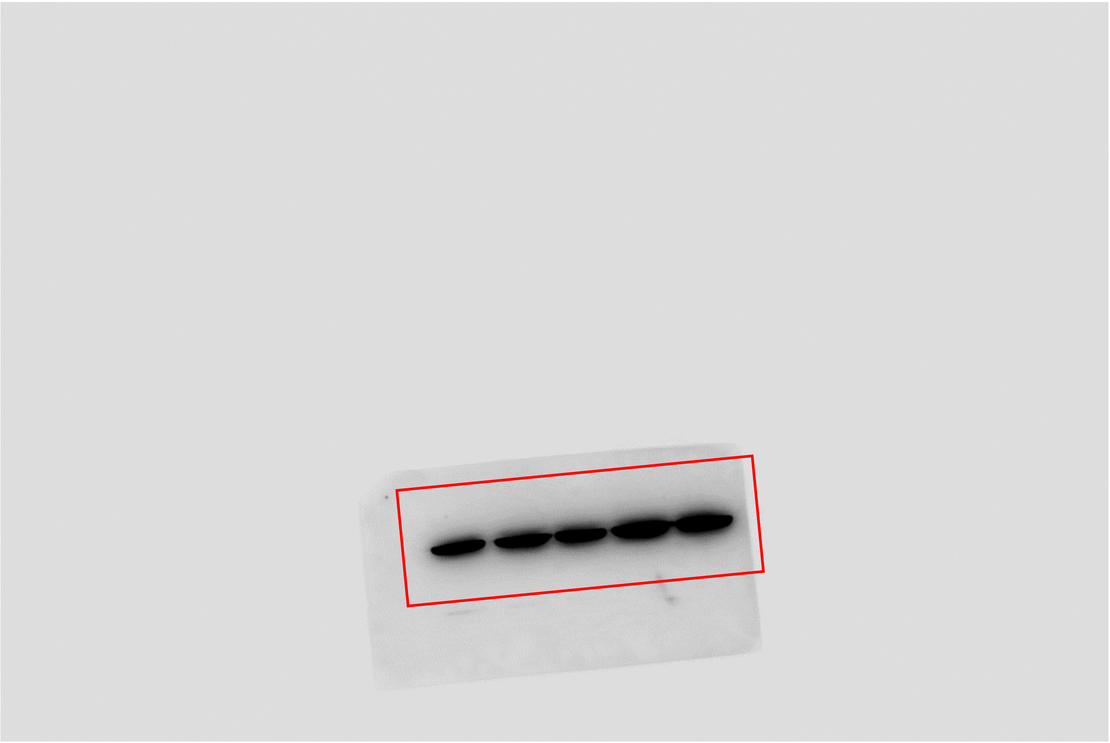

SMYD2

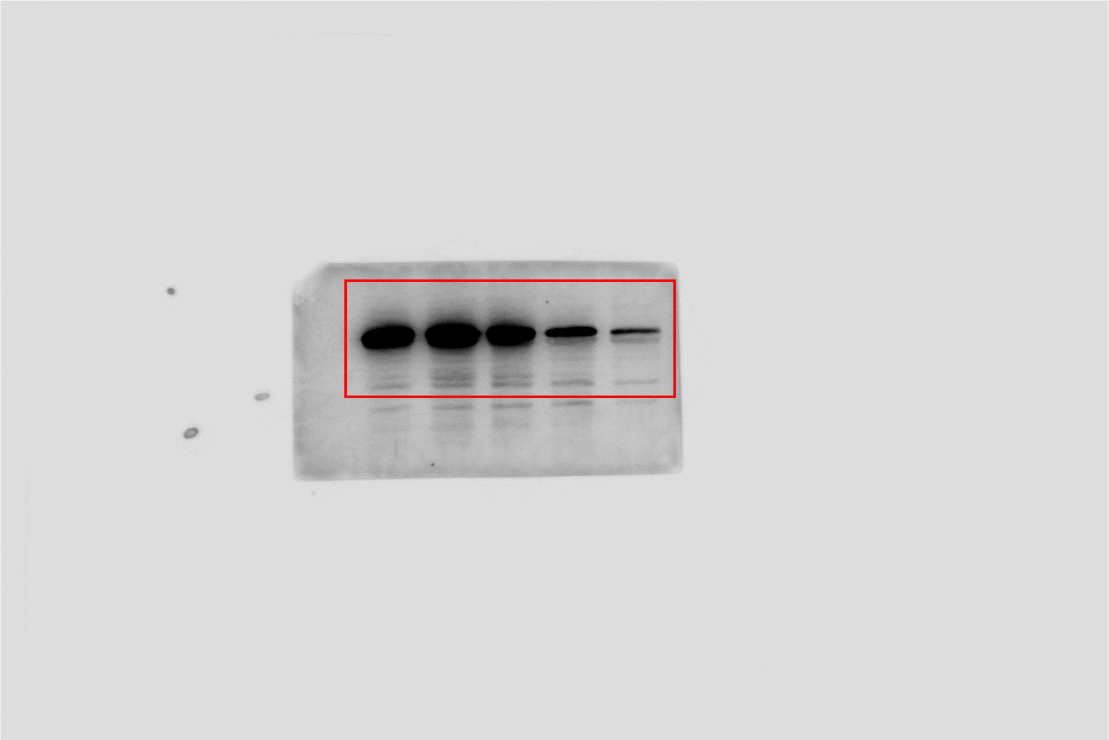

H

SMYD2

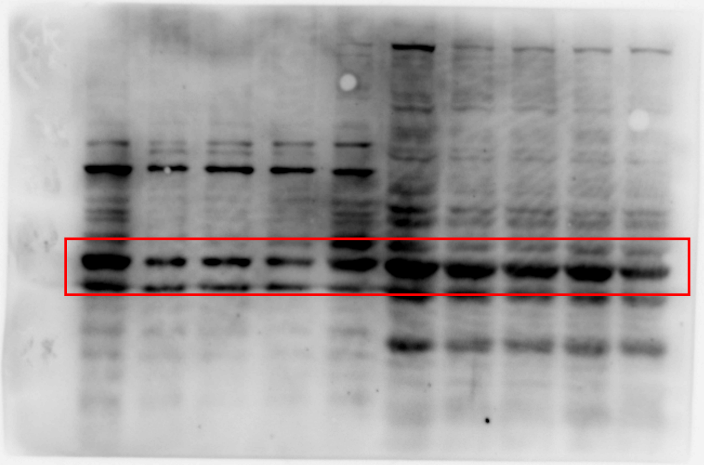

GAPDH

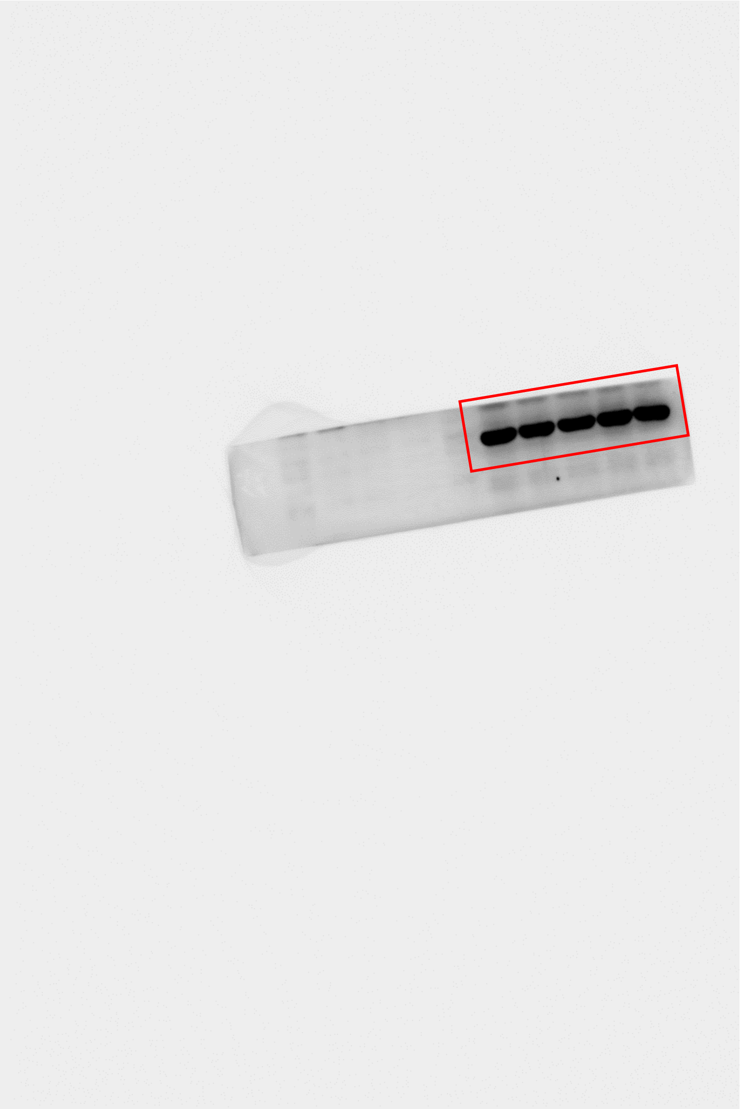

Lamin A/C

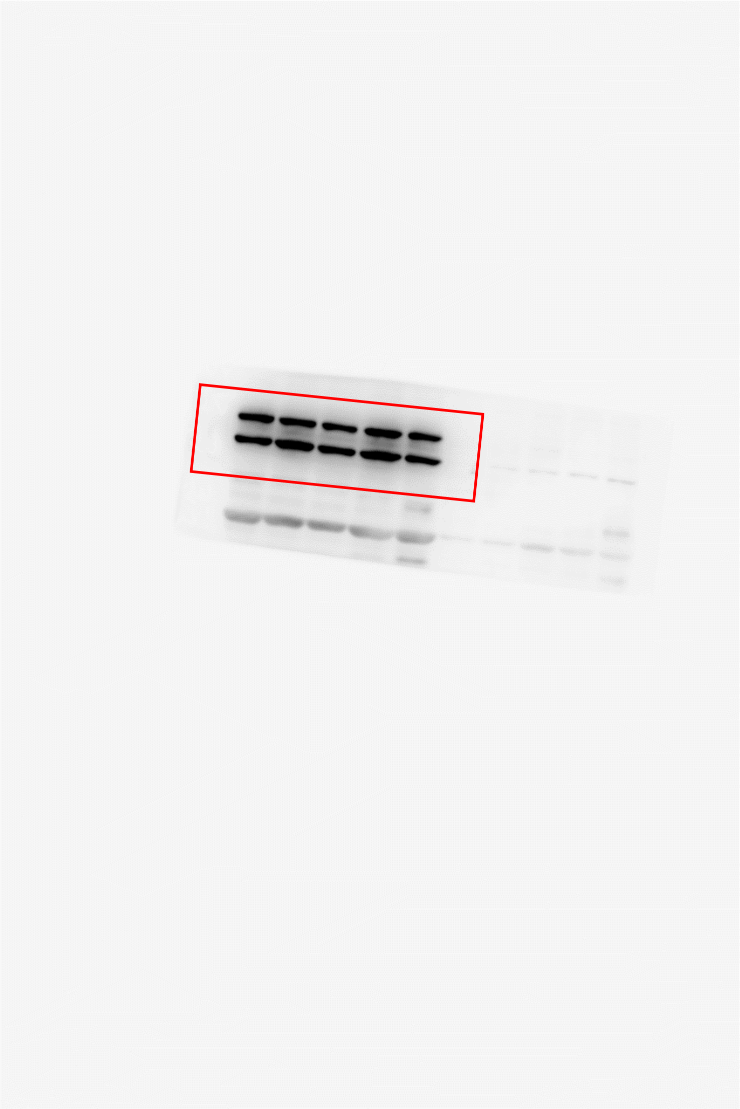

J

SEV
SMYD2

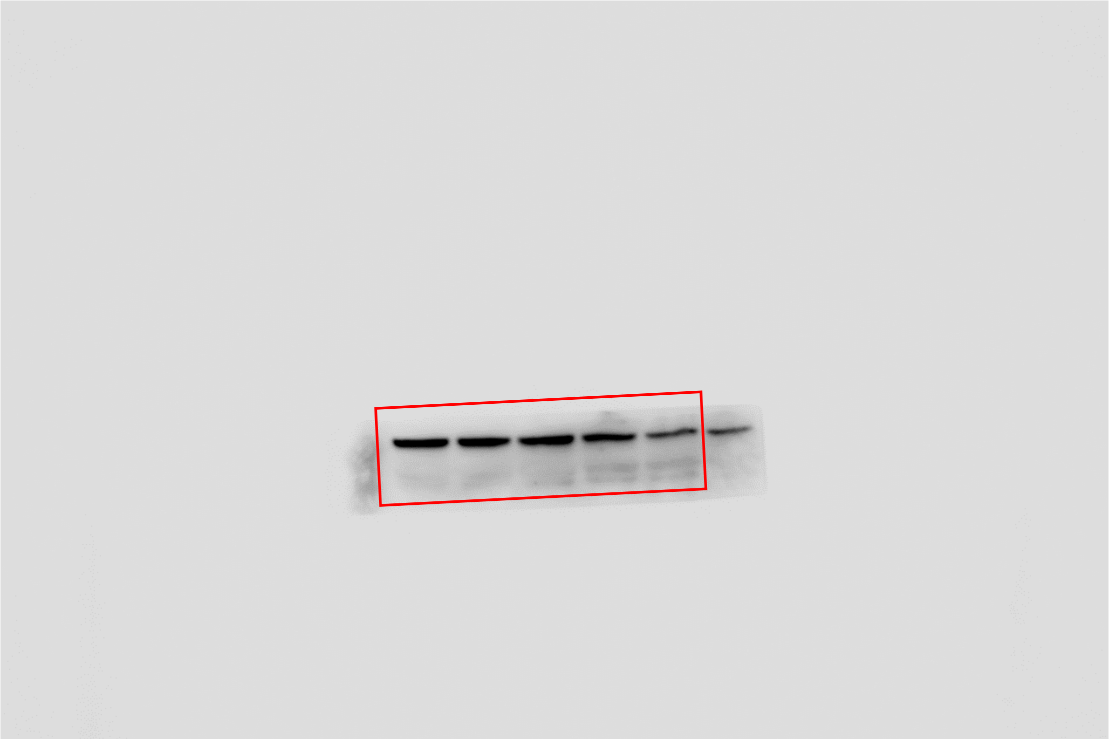

SEV
β-actin

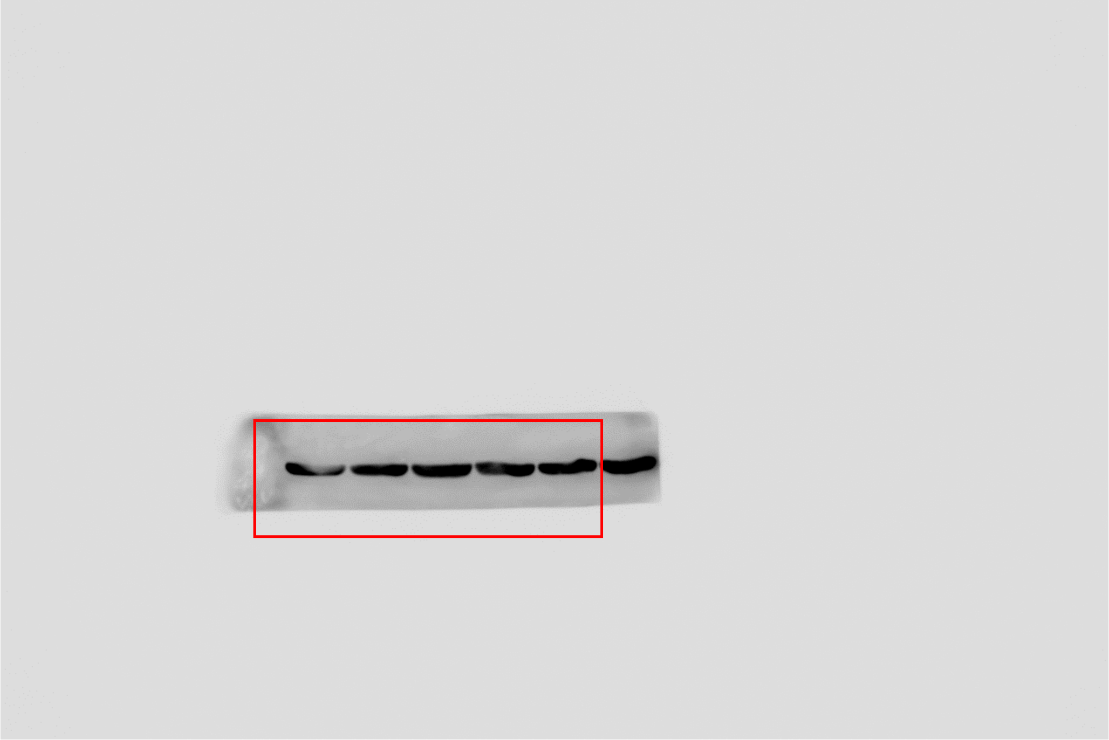

HSV
SMYD2

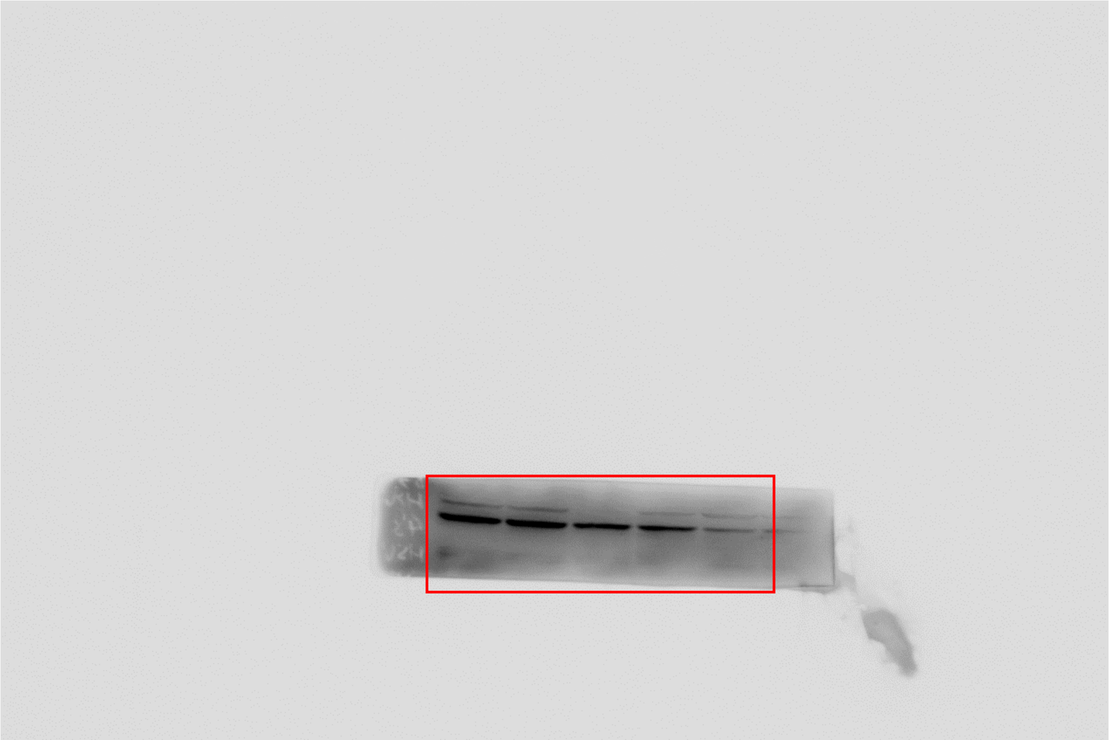

HSV
β-actin

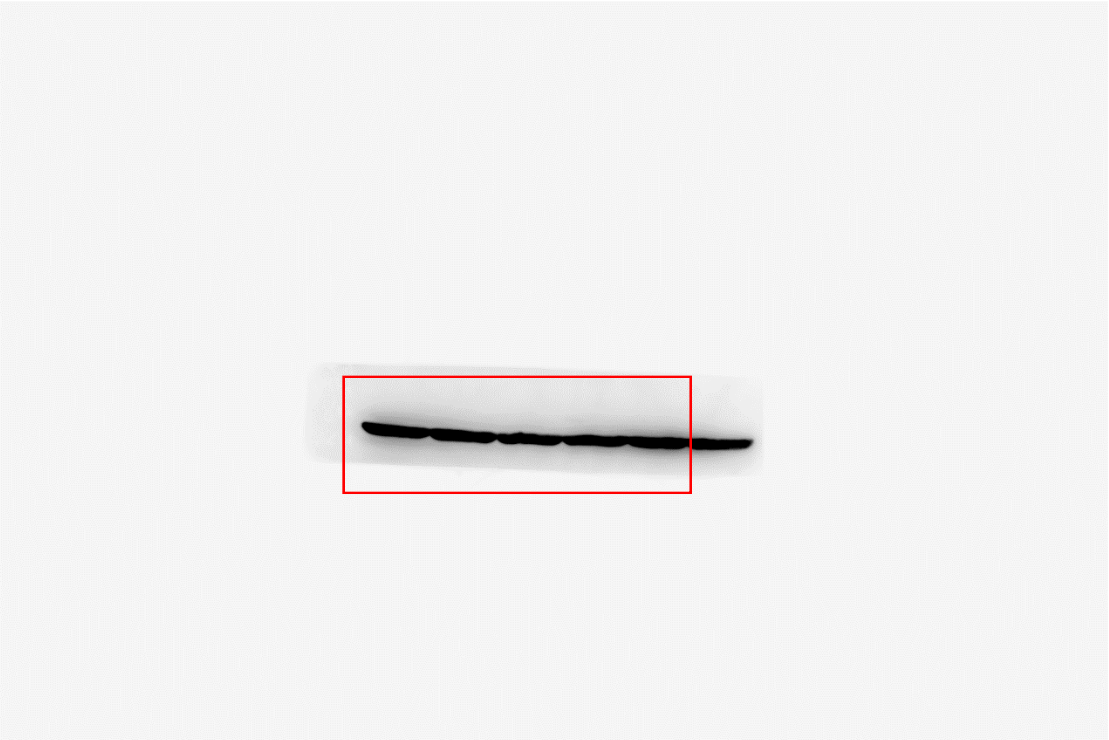

LM
SMYD2

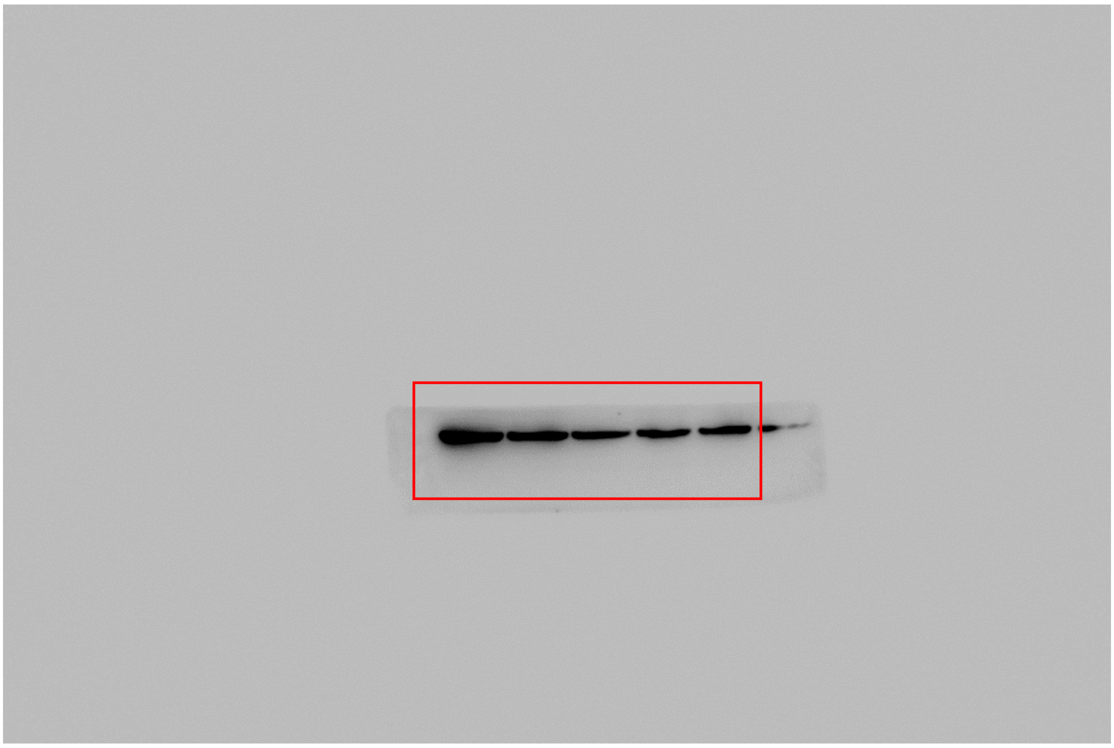

LM
β-actin

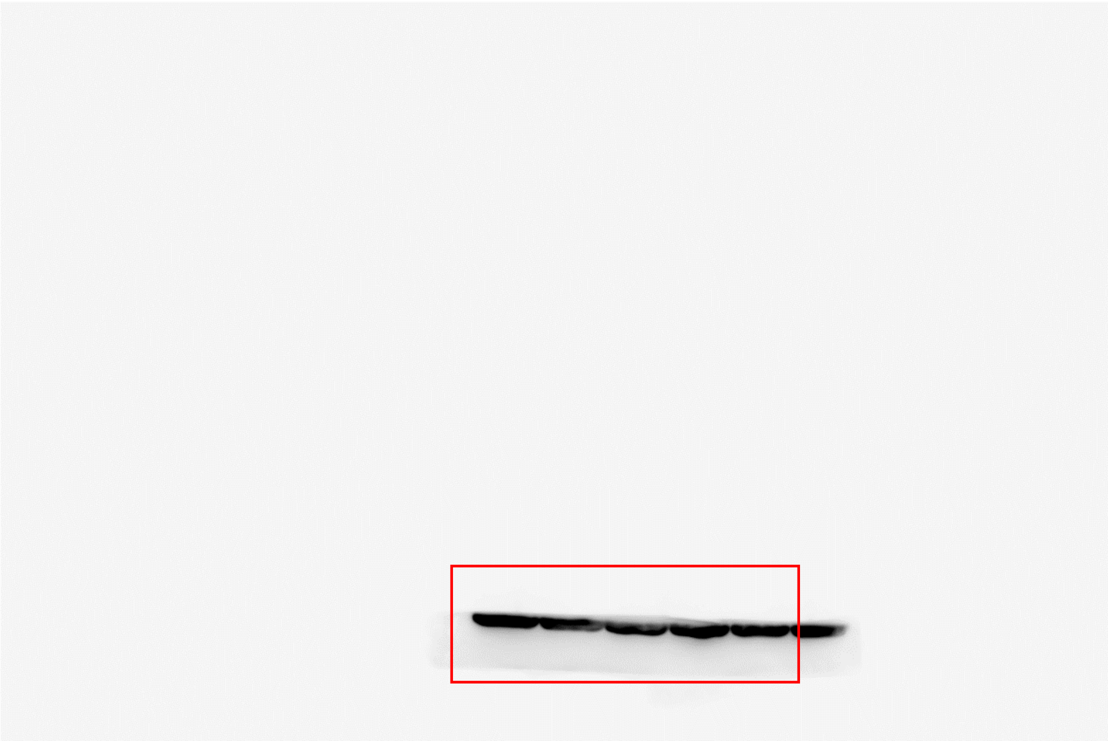

K

IFN-β
β-actin

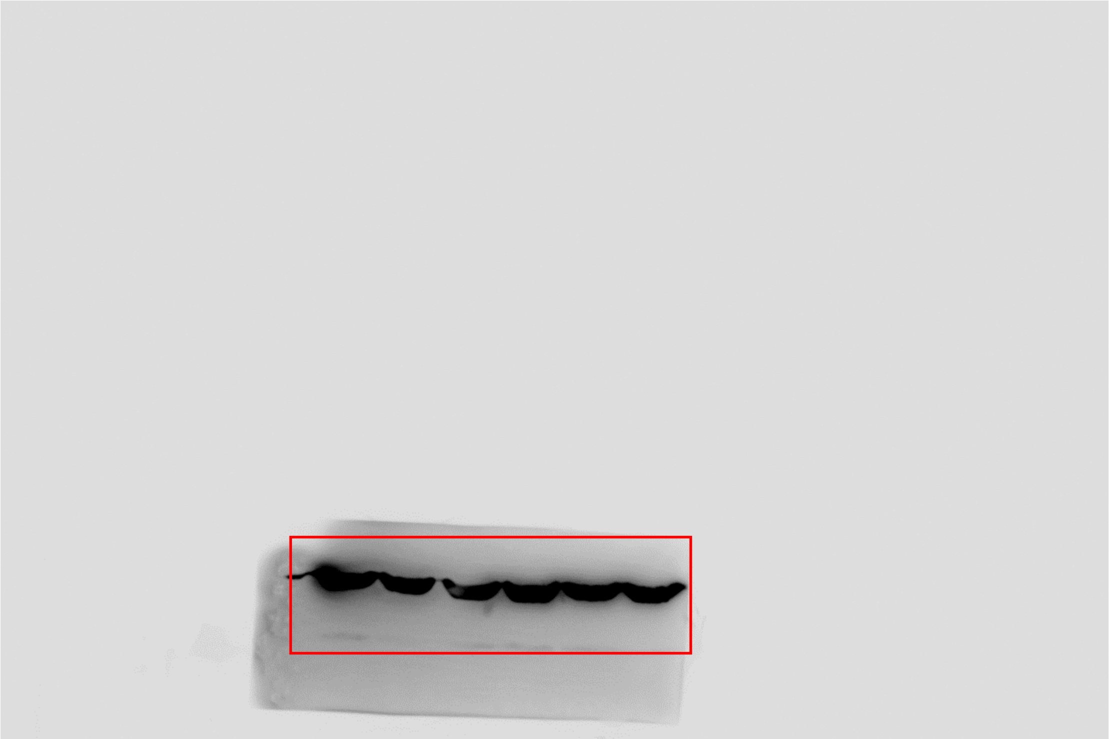

IFN-β
SMYD2

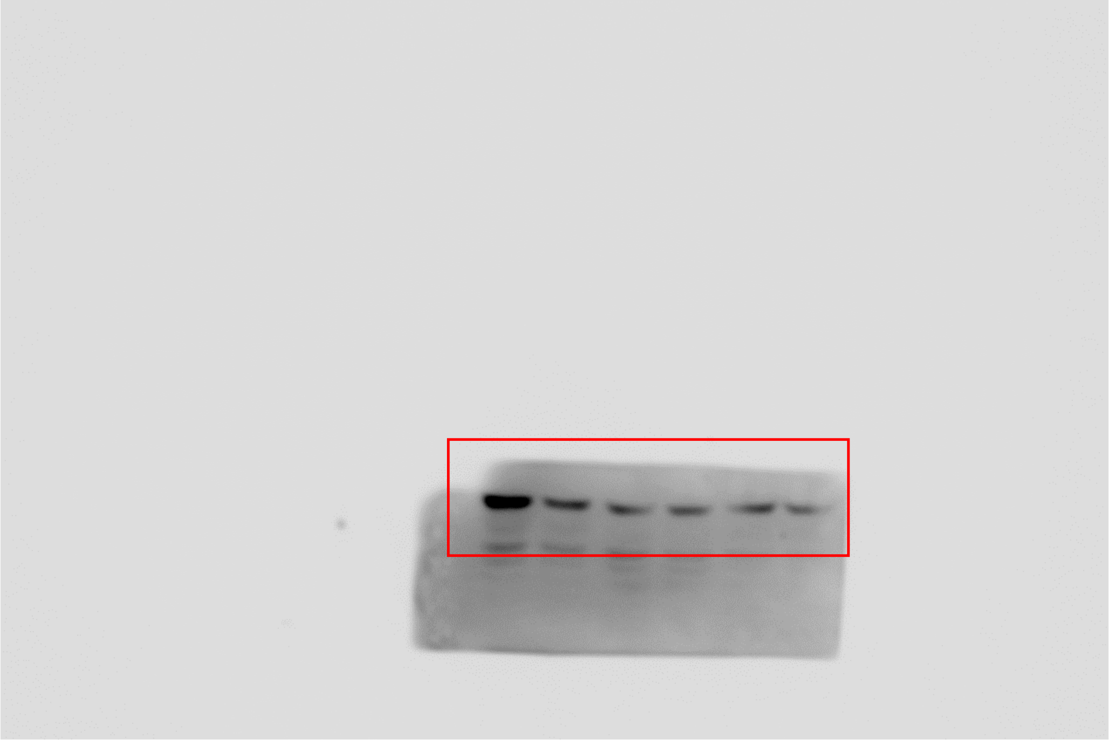

IL-6
SMYD2

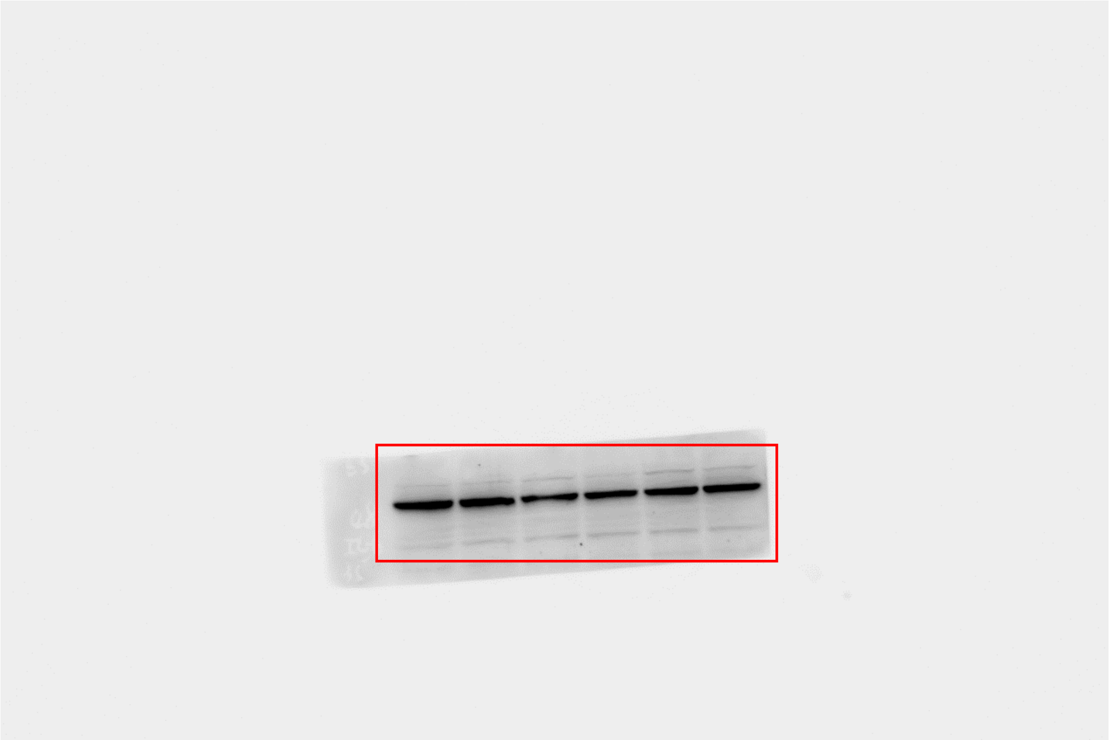

IL-6
β-actin

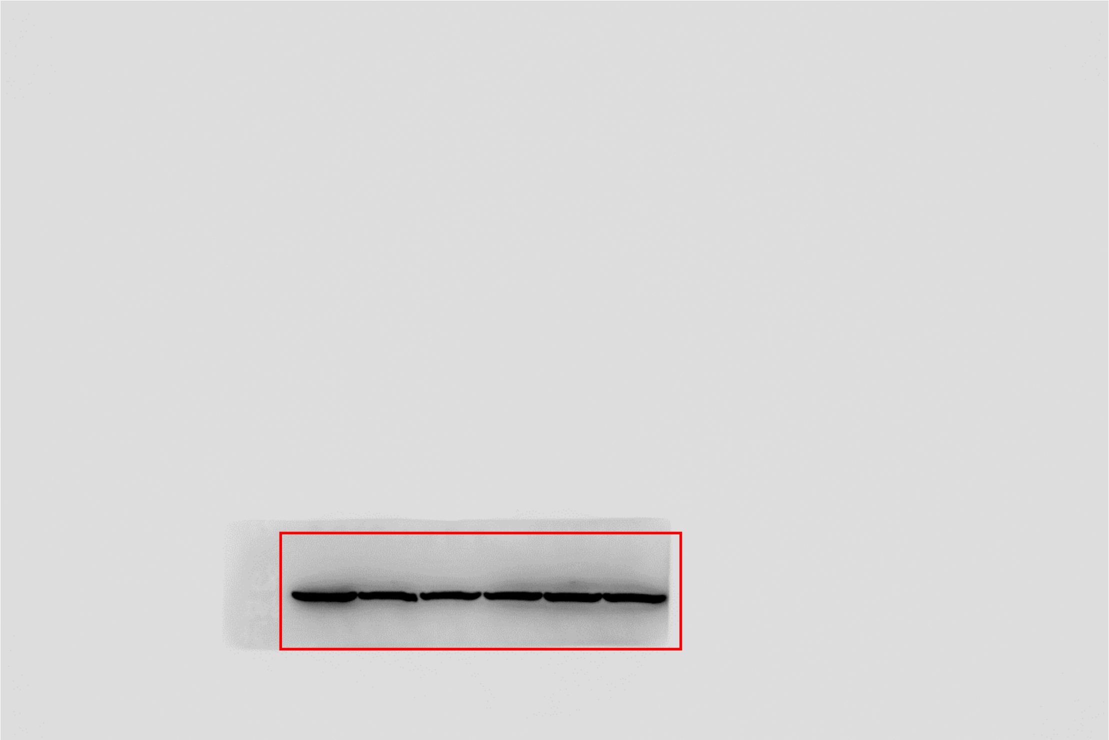

TNF-α
SMYD2

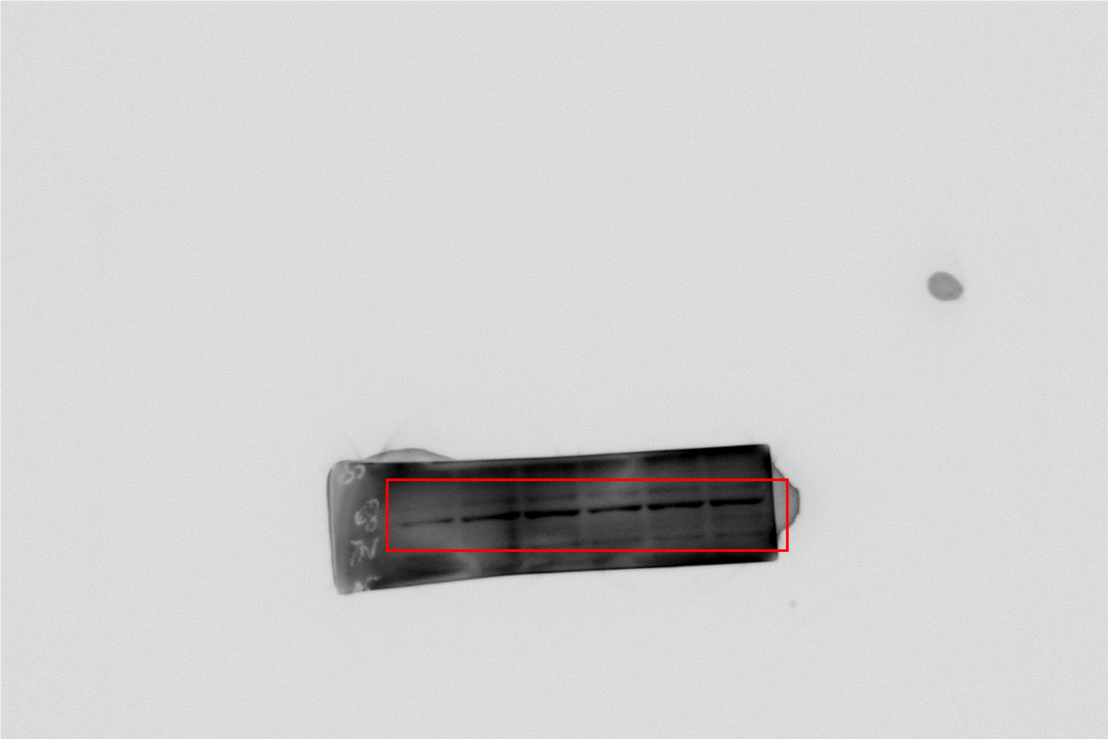

TNF-α
β-actin

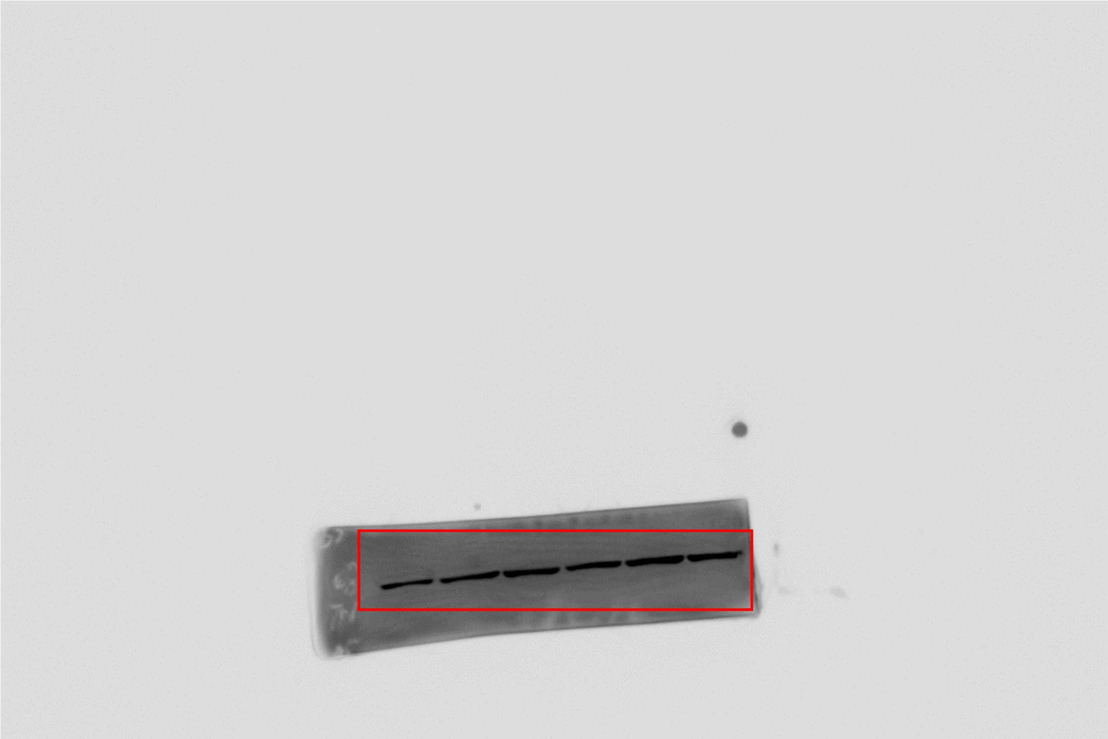

IFN-α
SMYD2

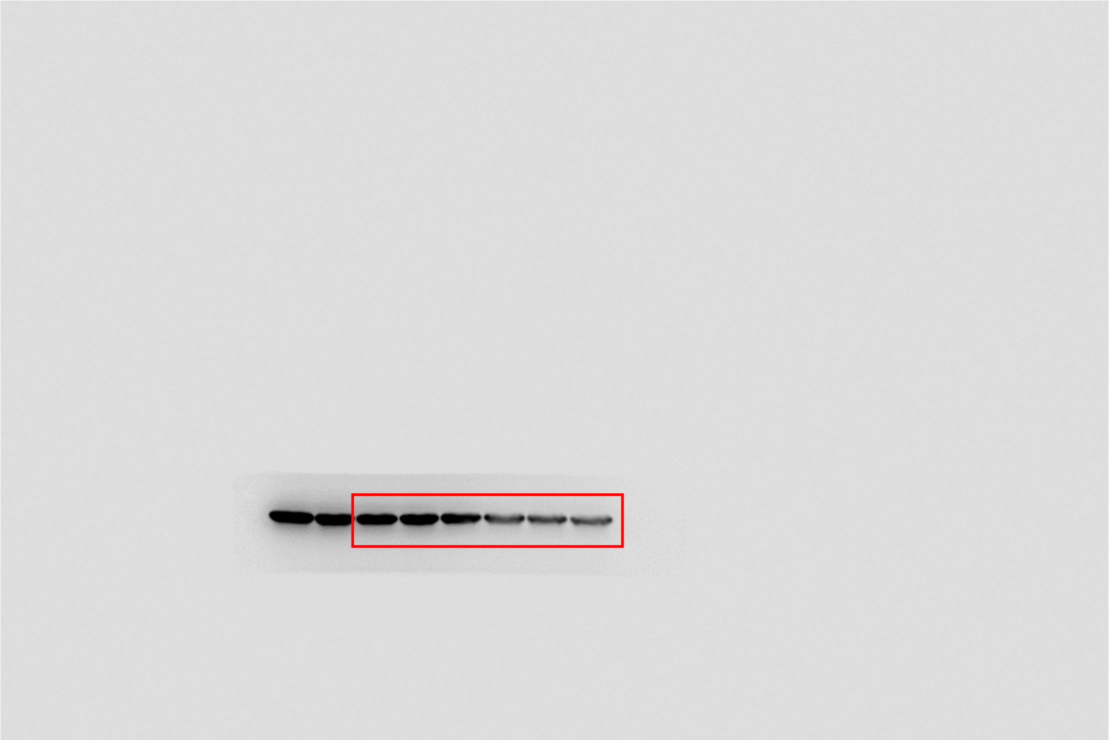

IFN-α
β-actin

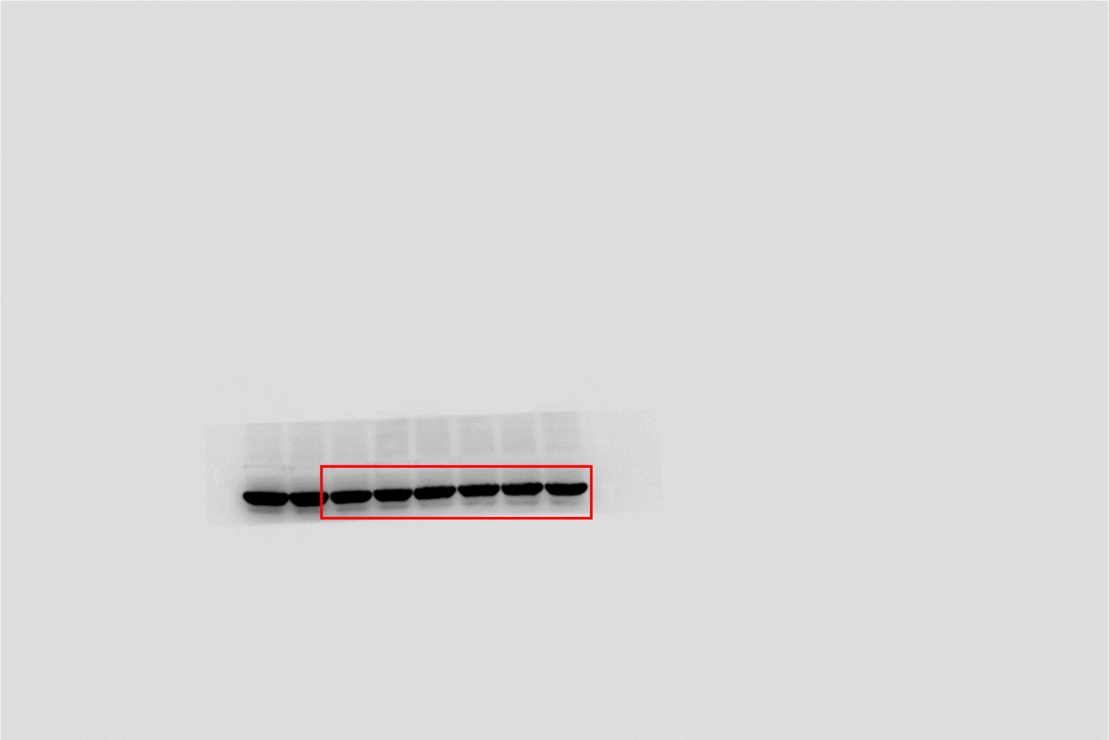

Supplementary Figure 3

B

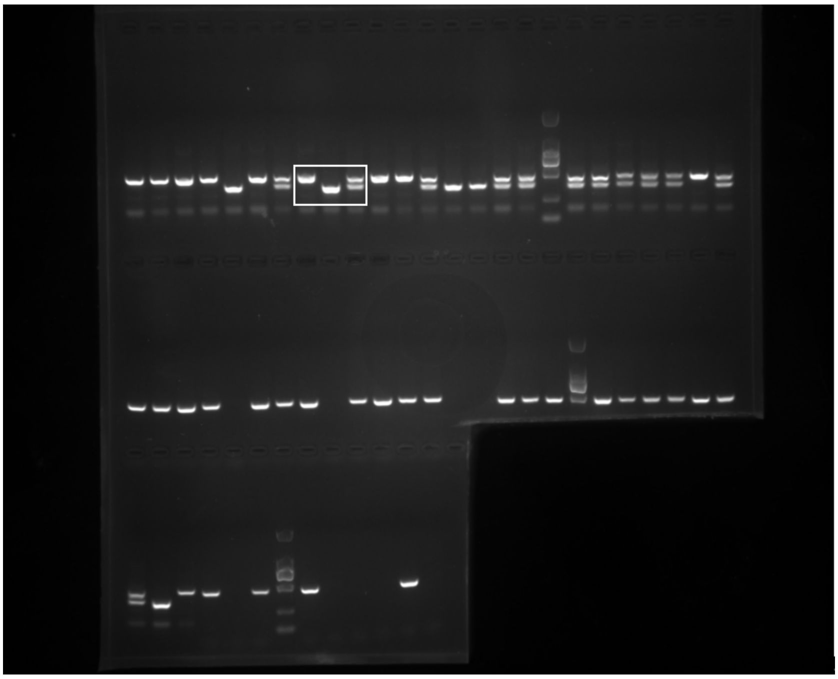

C

Smyd2

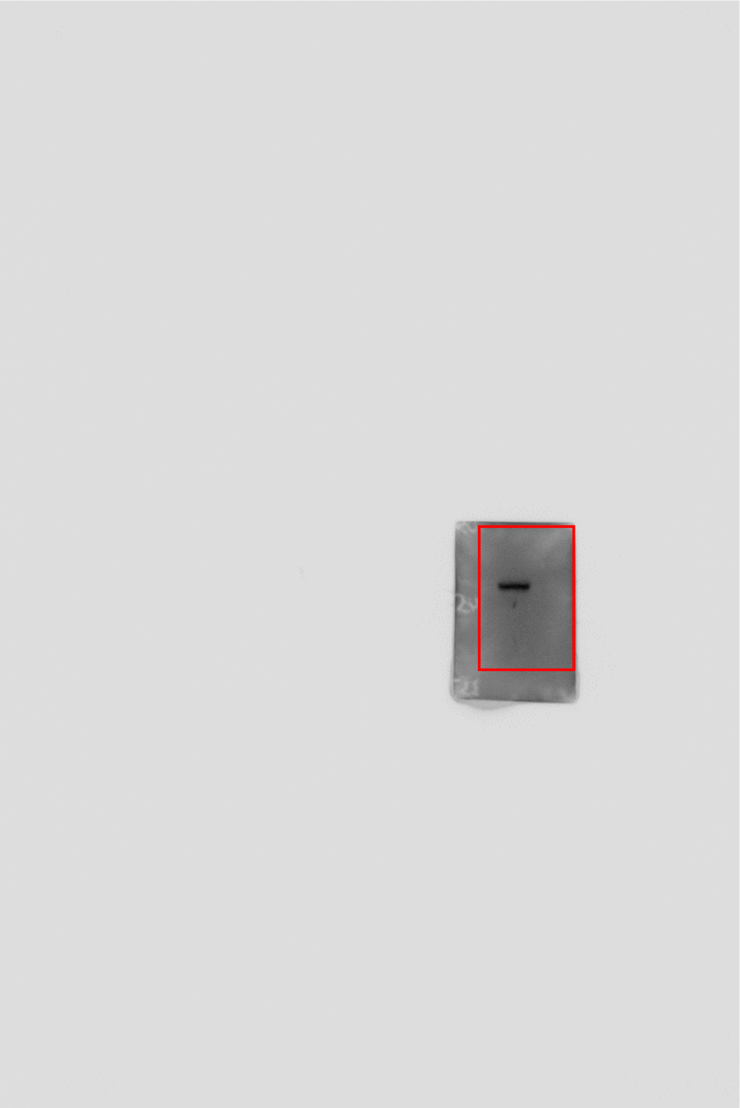

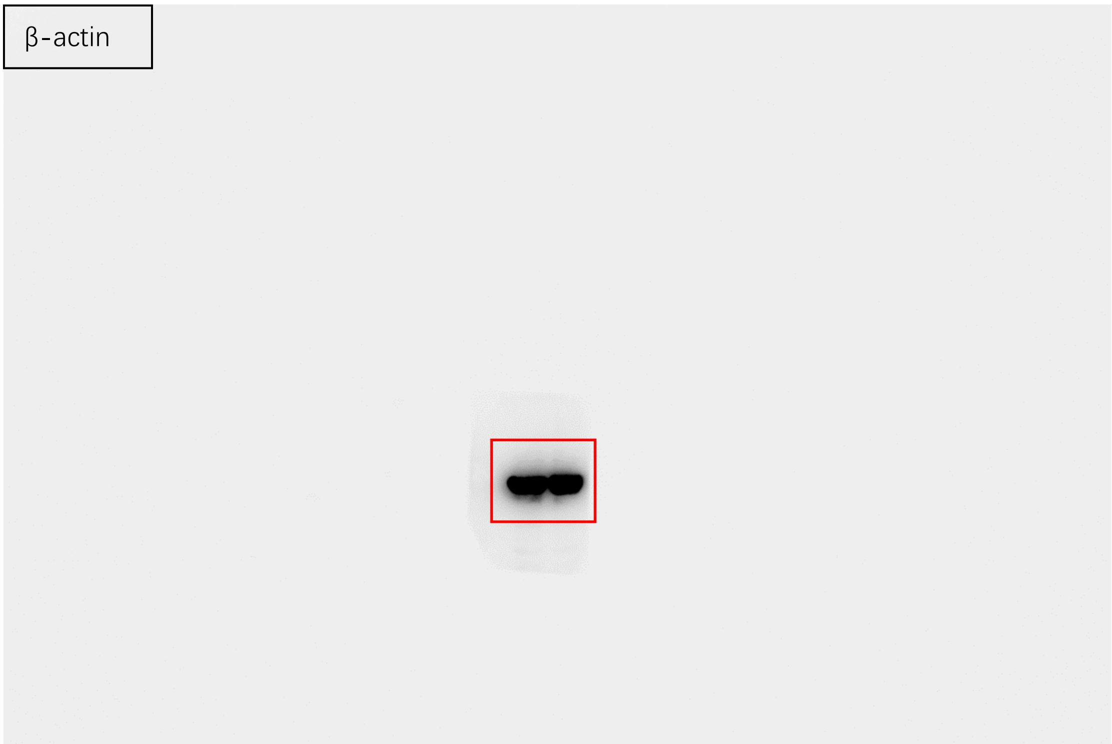

Supplementary Figure 7

A

p-IκB

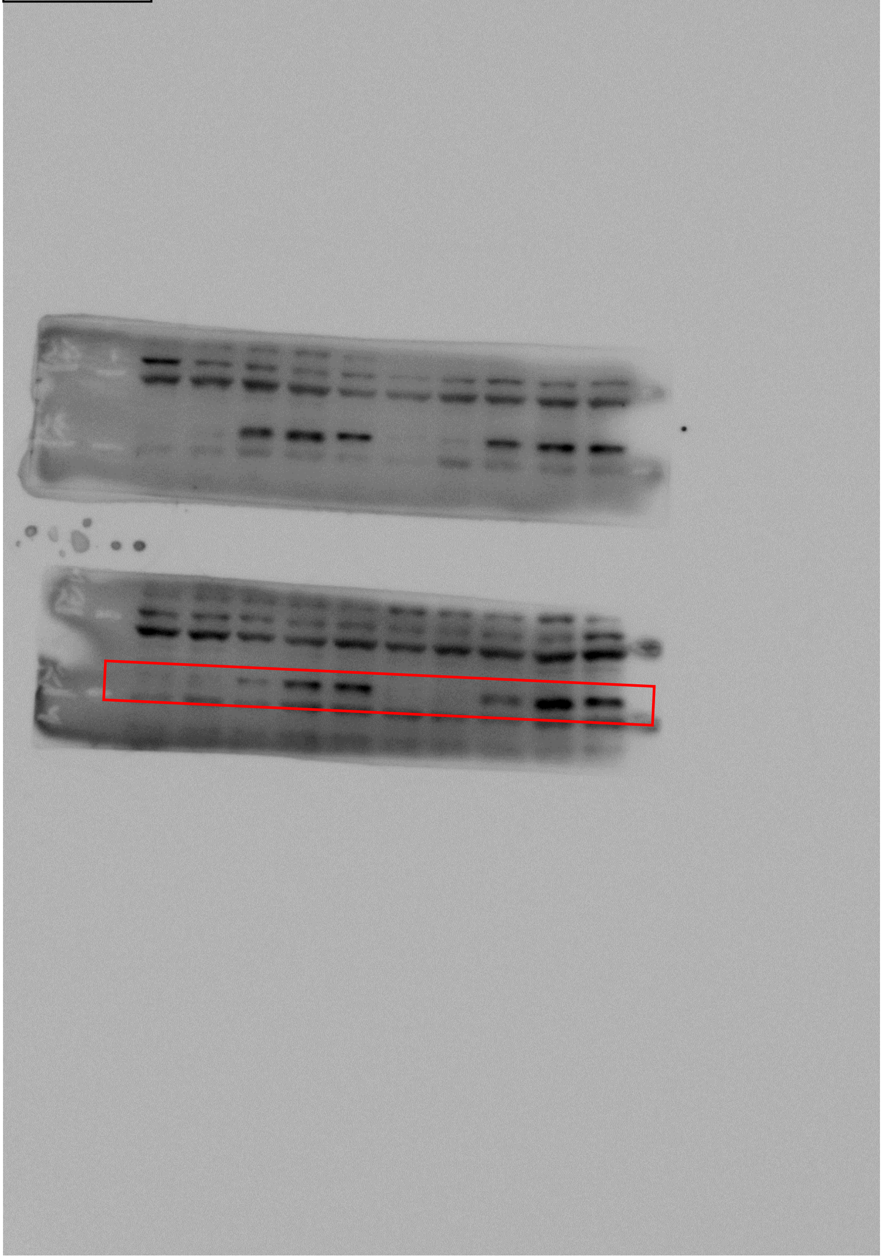

IκB

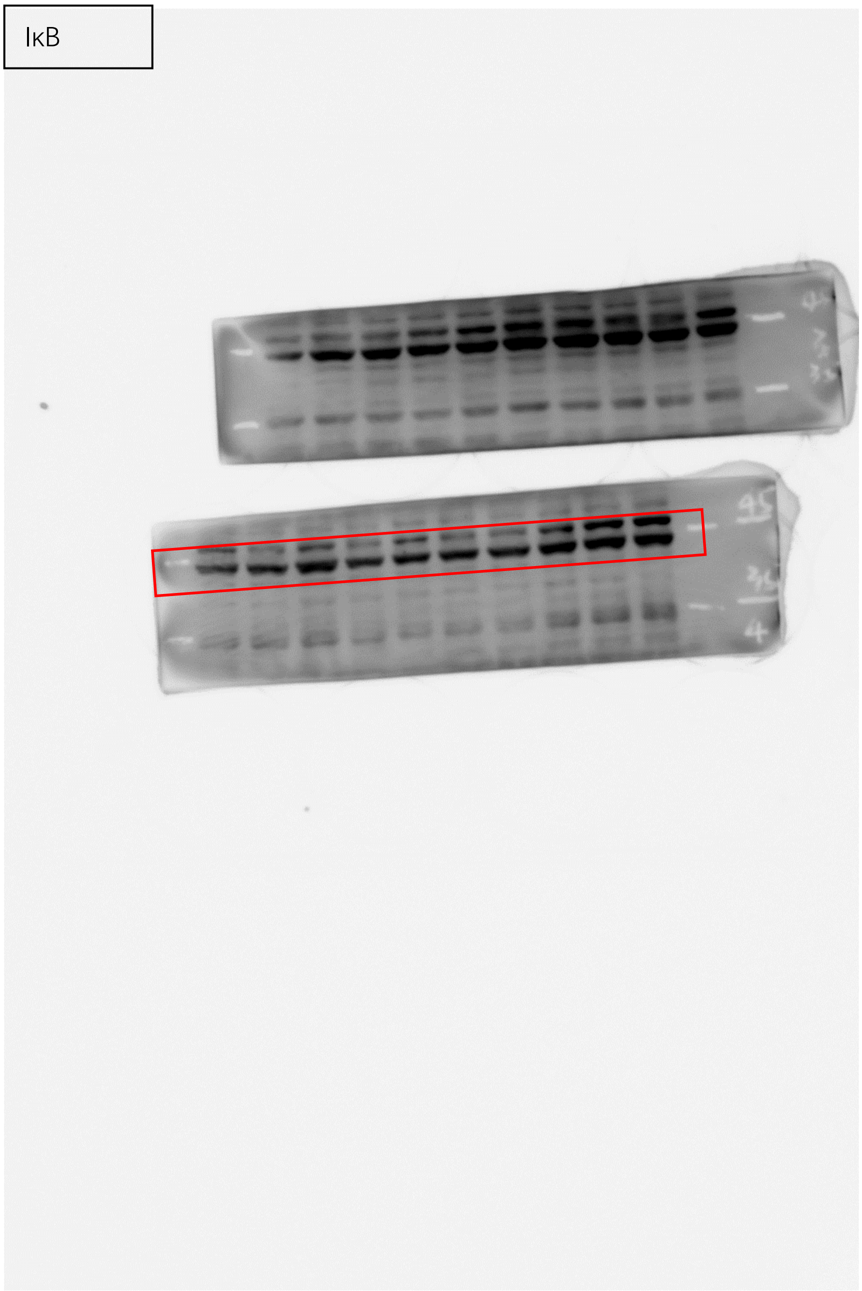

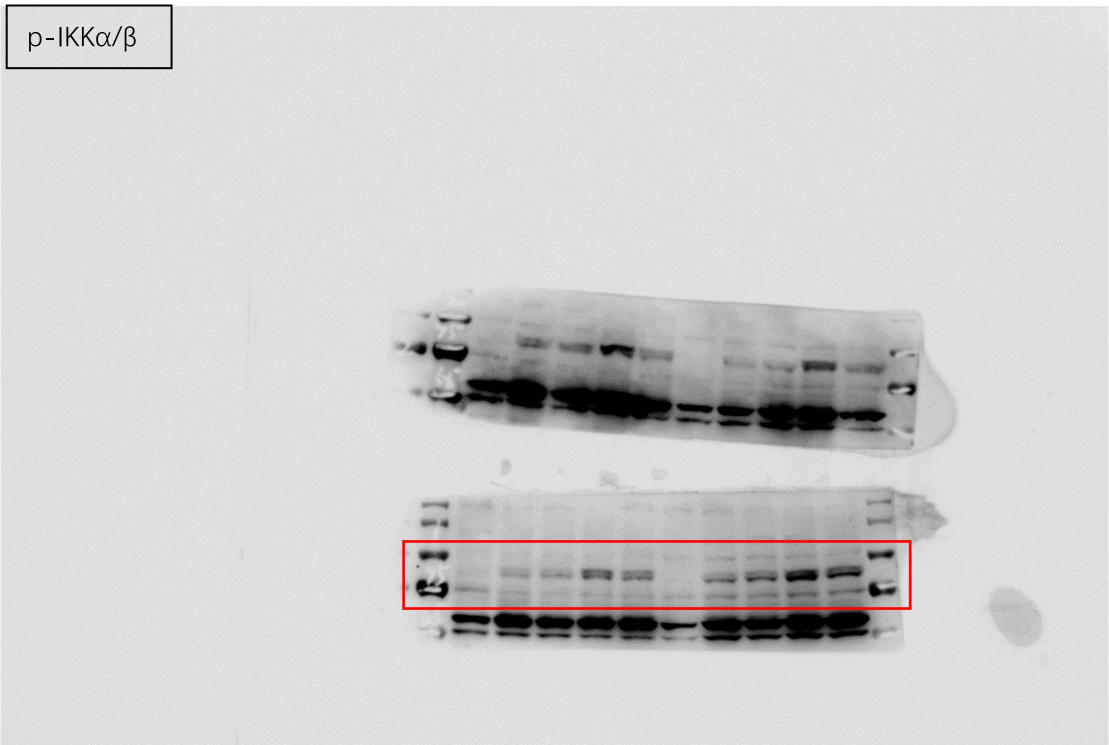
p-IKKα/β

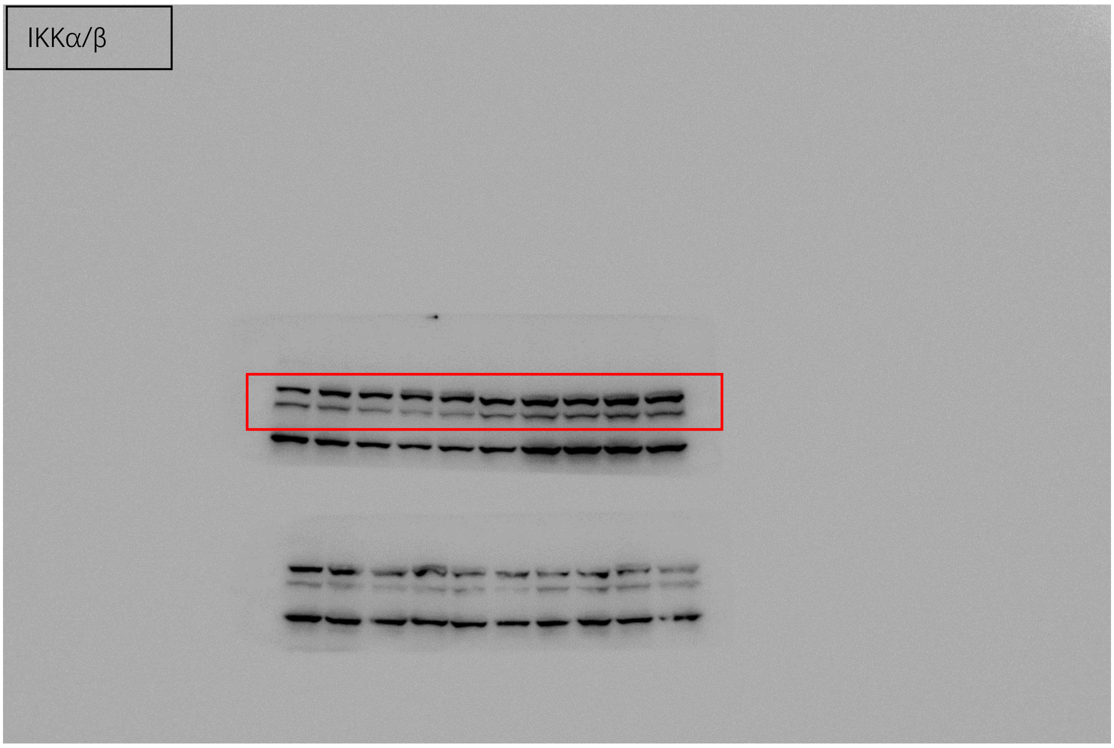
IKKα/β

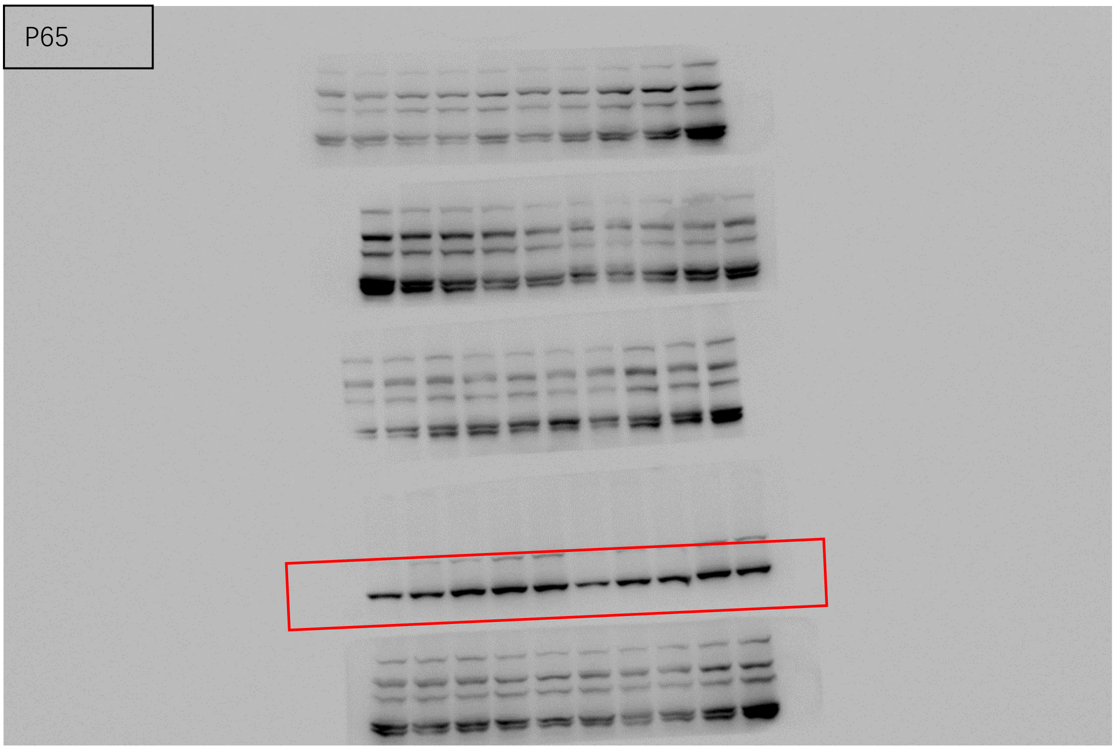
P65

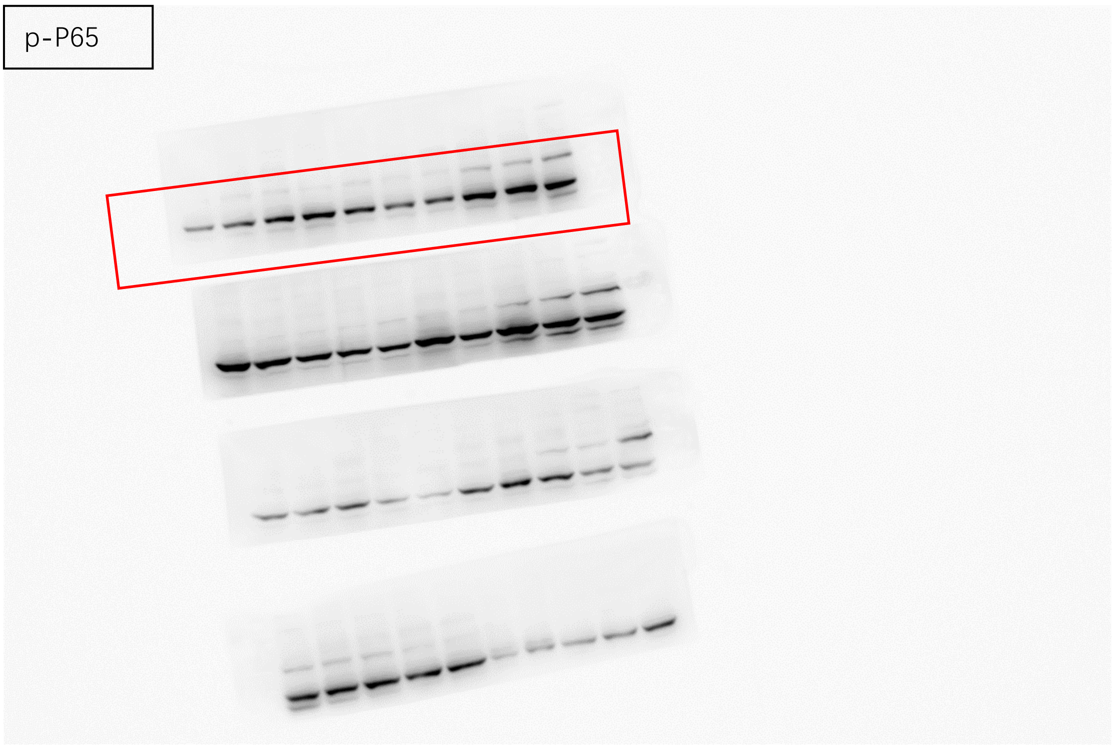
p-P65

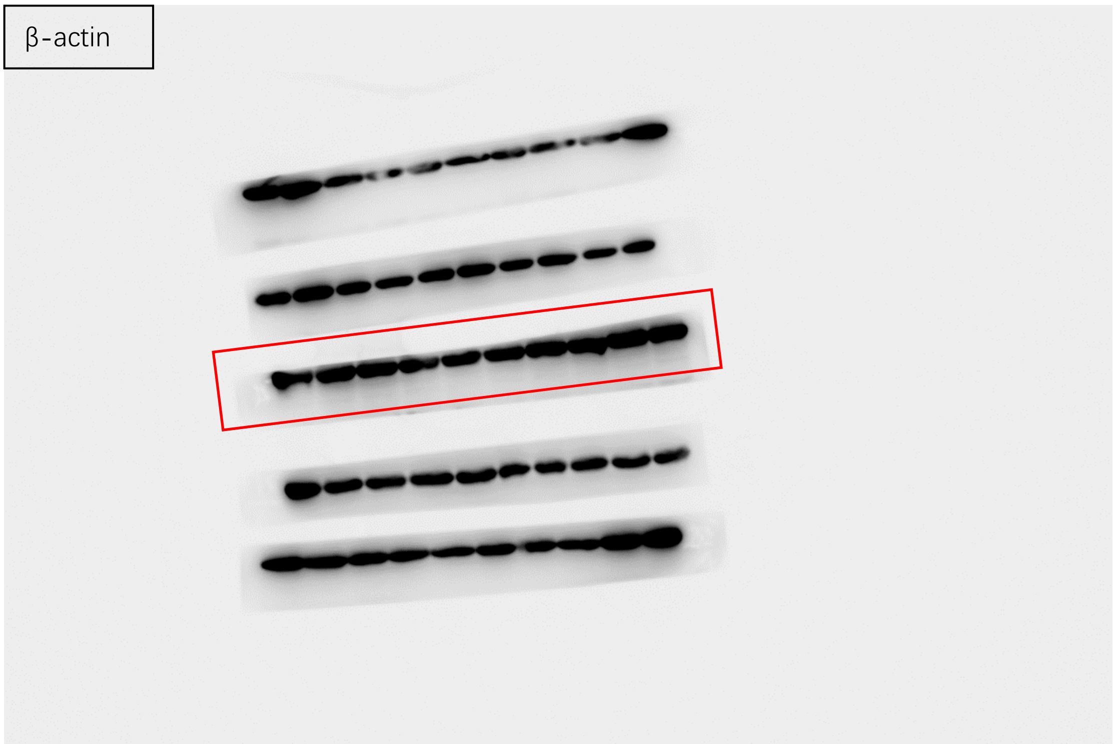
β-actin

E

Ppp1cc

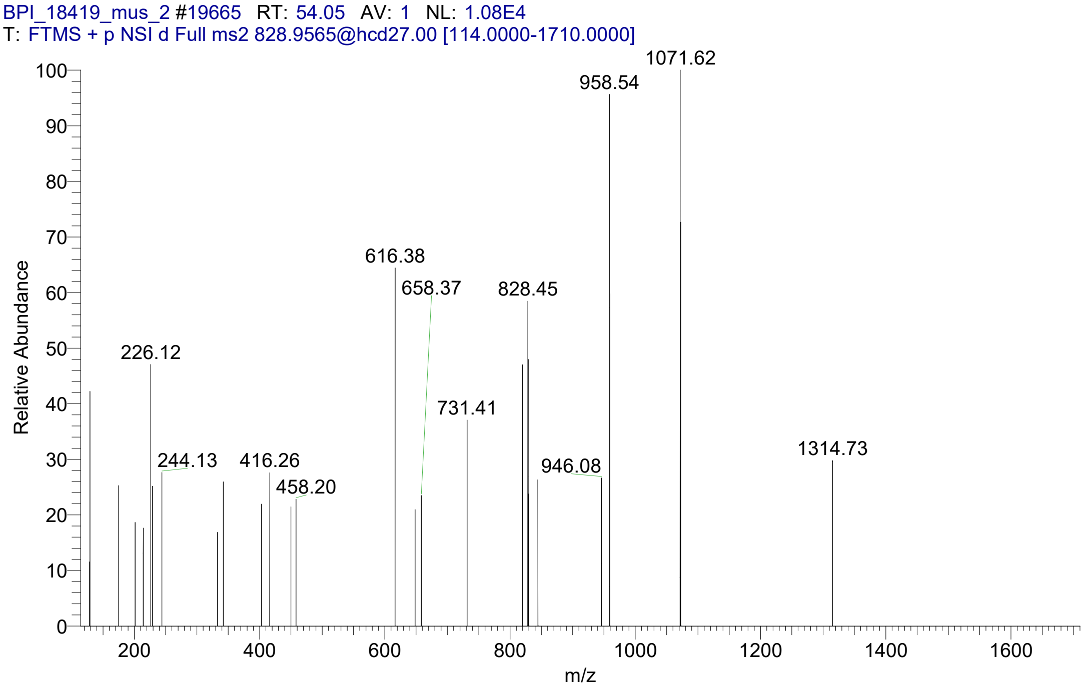
BPI_18419_mus_2 #19665 RT: 54.05 AV: 1 NL: 1.08E4
T: FTMS + p NSI d Full ms2 828.9565@hcd27.00 [114.0000-1710.0000]
Relative Abundance
m/z
1071.62
958.54
616.38
658.37
828.45
226.12
731.41
244.13
416.26
458.20
946.08
1314.73
0
10
20
30
40
50
60
70
80
90
100
200
400
600
800
1000
1200
1400
1600

Ppp1ca

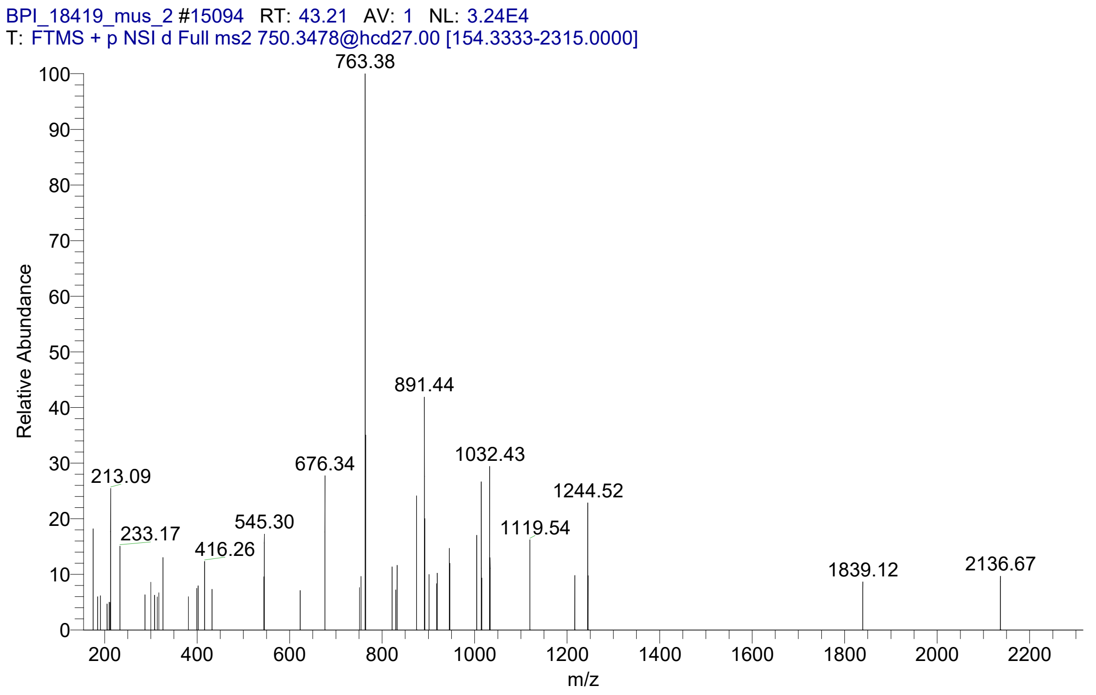

F

PP1α
PP2A

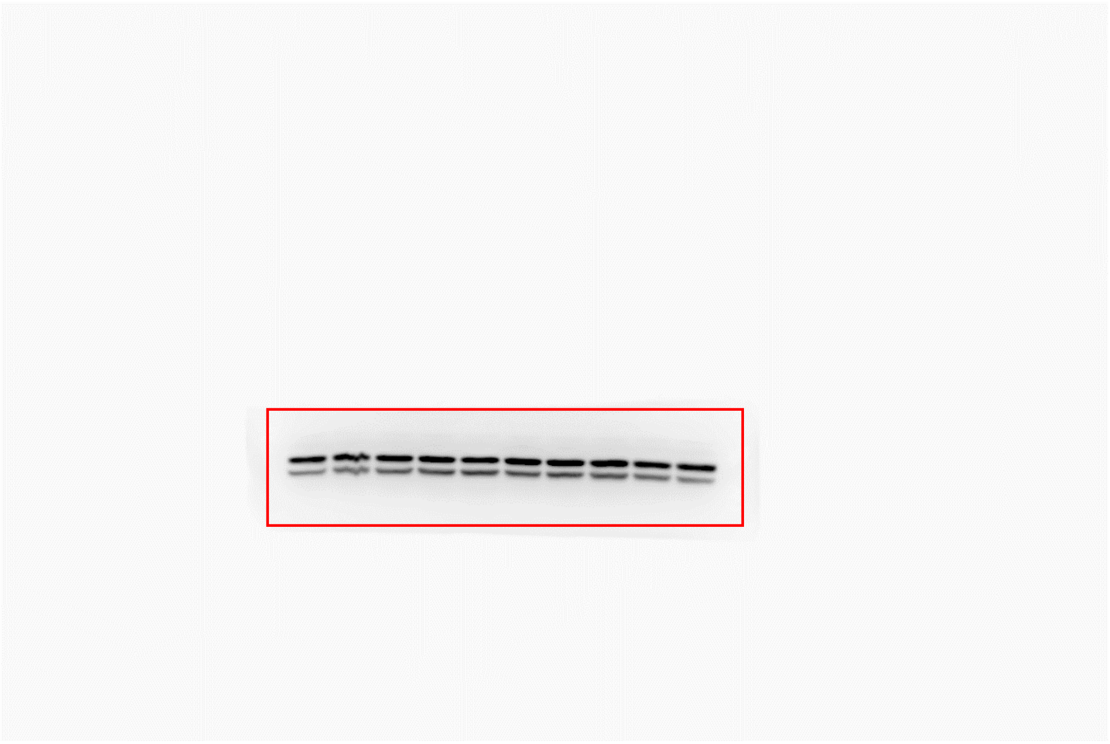

β-actin

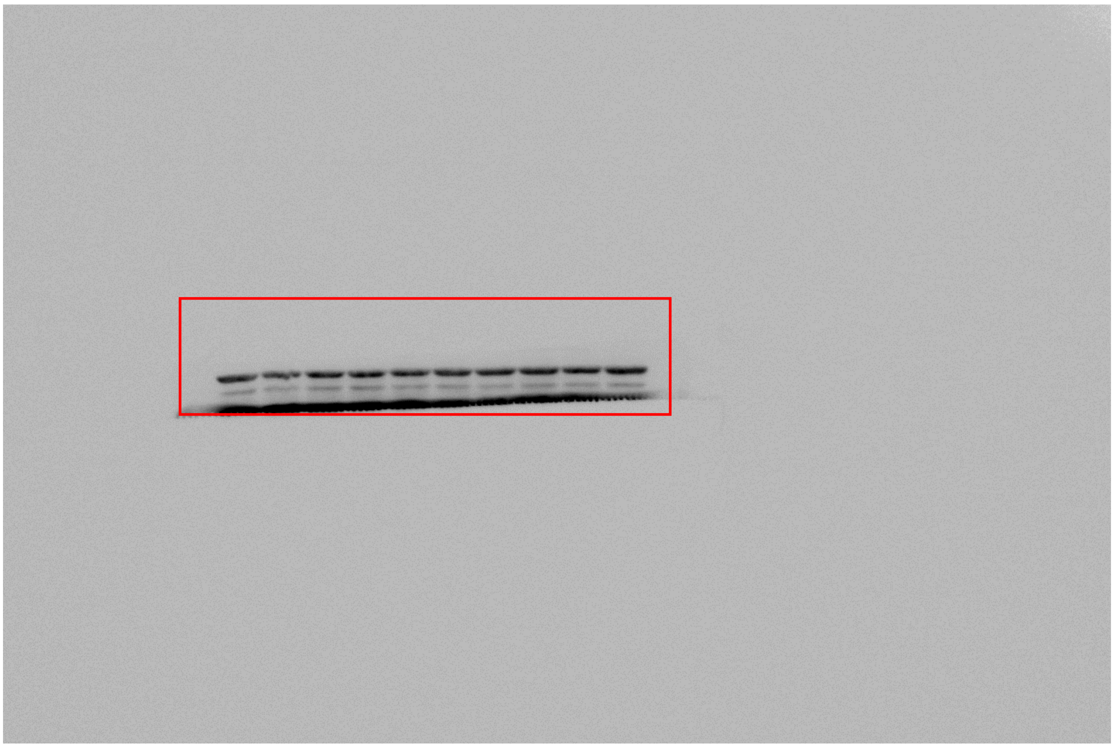

Supplement: Supplementary file 2 — Unprocessed gel and images [file 41419_2023_6118_MOESM2_ESM.pdf]
